# Supplementary material for: Deep MALDI-MS spatial omics guided by quantum cascade laser mid-infrared imaging microscopy
Source: Nat Commun. 2025 May 22;16:4759. doi: 10.1038/s41467-025-59839-3 (PMC12098849; doi:10.1038/s41467-025-59839-3)
Supplement: Supplementary file 1 — Supplementary Information [file 41467_2025_59839_MOESM1_ESM.pdf]

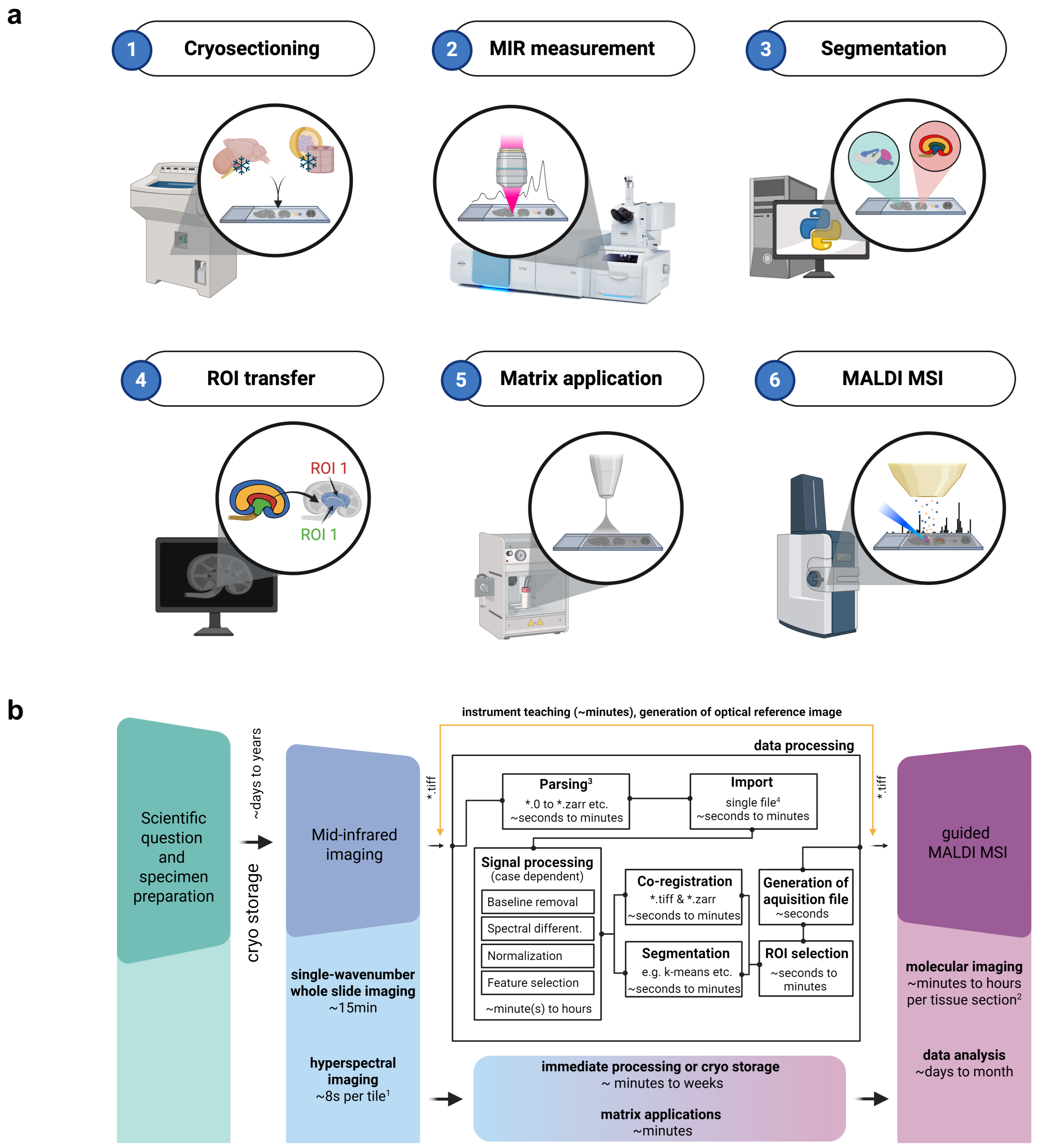

Supplementary Fig. 1. Schematic workflow of quantum cascade laser-based mid-infrared imaging microscopy-guided MALDI MS imaging (QCL-MIR imaging-guided MSI). **a,** General workflow: **1**, Fresh-frozen tissue is cut and mounted onto indium tin oxide (ITO)-coated glass slides and dried in a desiccator. **2**, Mid-infrared (MIR) spectra covering the fingerprint region (950-1800 cm^{-1}) are

rapidly recorded using a quantum cascade laser (QCL)-based MIR imaging microscope in sweep-scan mode. **3**, MIR imaging datasets are segmented utilizing unsupervised methods like k-means clustering on most distinctive vibrational features. **4**, Segments are identified as regions of interest (ROIs) and co-registered with a reference whole-slide, *single wavenumber* MIR image. ROI information is subsequently written into the MSI data acquisition file. **5**, Specimens are spray-coated with MALDI matrix prior to measurement. **6**, Trapped ion mobility spectrometry-time of flight (timsTOF) mass spectrometry imaging restricted to ROIs defined by the QCL-based MIR data. **b,** Overview of the general steps of the workflow including the data flow. [1]The number of tiles per specimen depends on the size of the tissue section and the objective being used. [2]The measurement time on the MS instrument per tissue section depends on the instrument parameters like pixel size etc. [3]This step might be obsolete in the latest version of OPUS. [4]In general, the import of multiple files can be performed, but parallel multi-file processing is not foreseen in the current implementation. Created in BioRender. https://BioRender.com/ np9x3ry

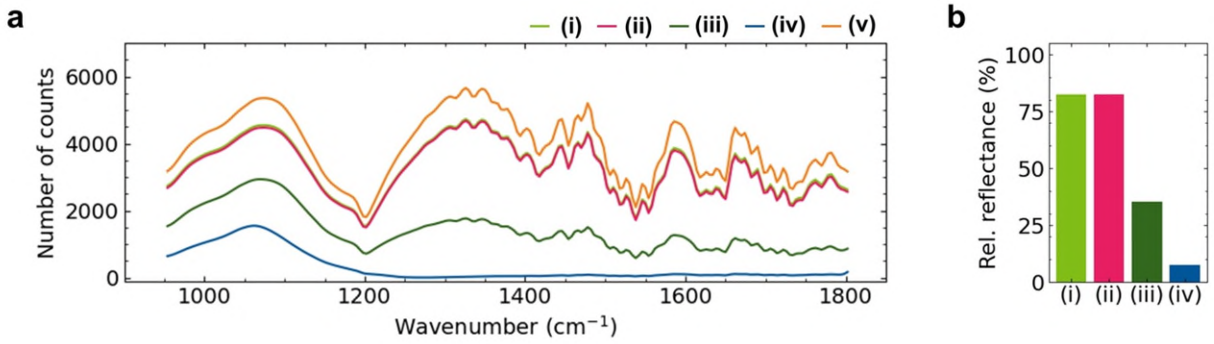

Supplementary Fig. 2. Mid-infrared (MIR) optical properties of various surface-coated glass slides. **a,** The relative reflectance across the mid-infrared fingerprint region was measured as the number of counts on the detector from a single channel measurement on the Hyperion II ILIM (infrared laser imaging) microscope for various ITO-coatings (i), (ii) and (iii), for normal glass slide (iv), and for a gold-coated surface (v). **b,** Spectral responses for ITO-coated slides (relative to gold coating (v)) were averaged over the entire spectra ranging from 950-1800 cm^{-1}. The mean relative reflectance ranged from 82% for both the Bruker MALDI IntelliSlide (i) and the Diamond Coating ITO-coated glass slide (ii) to 35% for the Bruker ITO-coated glass slide (iii) to 7% for the non-coated SuperFrost Plus Adhesion glass slide. As a result, for biomedical specimen analysis with the presented workflow, Bruker MALDI IntelliSlide (i) and Diamond Coating ITO-coated slides (ii) were mainly used, since they allow for transmitted-light microscopy and visual inspection during sample preparation as well as imaging of post-MALDI MSI stained samples. All mass spectrometry-related methods and protocols were optimized for both slide types. Source data is provided as a Source Data file.

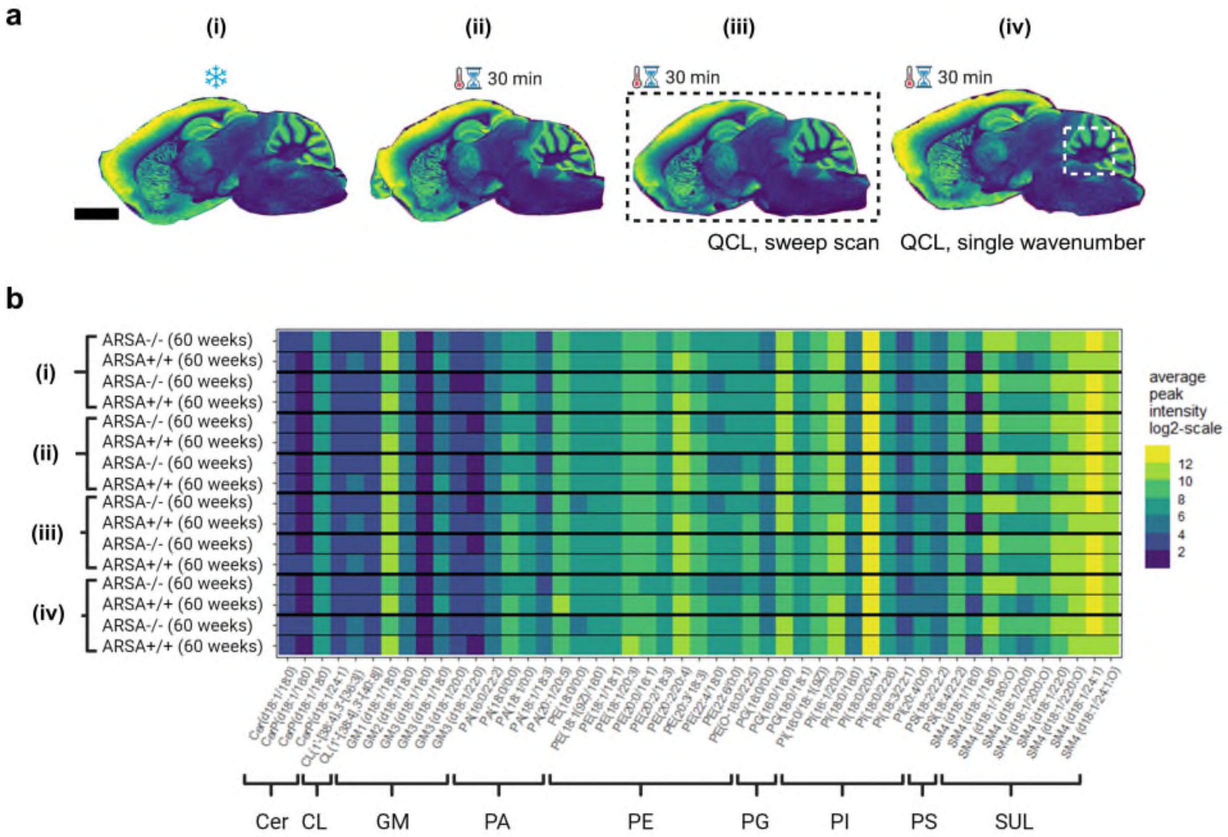

Supplementary Fig. 3. MSI assessment of lipid alterations induced in mouse brain cryo-sections by pre-analytical stress or by laser light. **a,** Four different stress conditions prior to MALDI MSI (timsTOF fleX, operated in qTOF mode) were investigated: **(i)** standard workflow where the samples are stored at -80 °C after sectioning and before matrix deposition, **(ii)** the specimen is kept at room temperature (RT) and standard pressure (SP) for about 30 min, **(iii)** the entire tissue section (dashed black box) is exposed to QCL-MIR imaging for 15 min in sweep scan mode and kept at RT and SP for in total of about 30 min, and **(iv)** where a defined 1.2 mm x 1.2 mm region of the tissue sections (dashed white box) is exposed to infrared light for 15 min at a constant wavenumber of 1656 cm^{-1} (amide I) used for generation of the reference image. Scale bar, 3 mm. **b,** Average peak intensity in negative ion mode MALDI MSI for several lipid classes across an *m/z* range of 600-1700. For each of the stress conditions **(i)** to **(iv)** in **a**, the procedure was repeated for n=4 different 60-week-old mice, two wild-type (ARSA+/+) mice and two arylsulfatase A-deficient (ARSA-/-) mice. Lipid assignment was done by *m/z*-based annotation in Metaspace (www.metaspace2020.eu). No indication of environment- or laser-induced lipid alterations was observed in the MSI data. Differences in peak intensity were within the expected range, considering biological variability and known batch effects in MALDI MSI[4]. Abbreviations: Cer: Ceramides, CL: cardiolipin, GM: ganglioside, PA: phosphatidic acid, PE: phosphatidylethanolamine, PG: phosphatidylglycerol, PI: phosphatidylinositol, PS: phosphatidylserine and SUL: sulfatide. Source data is provided as a Source Data file.

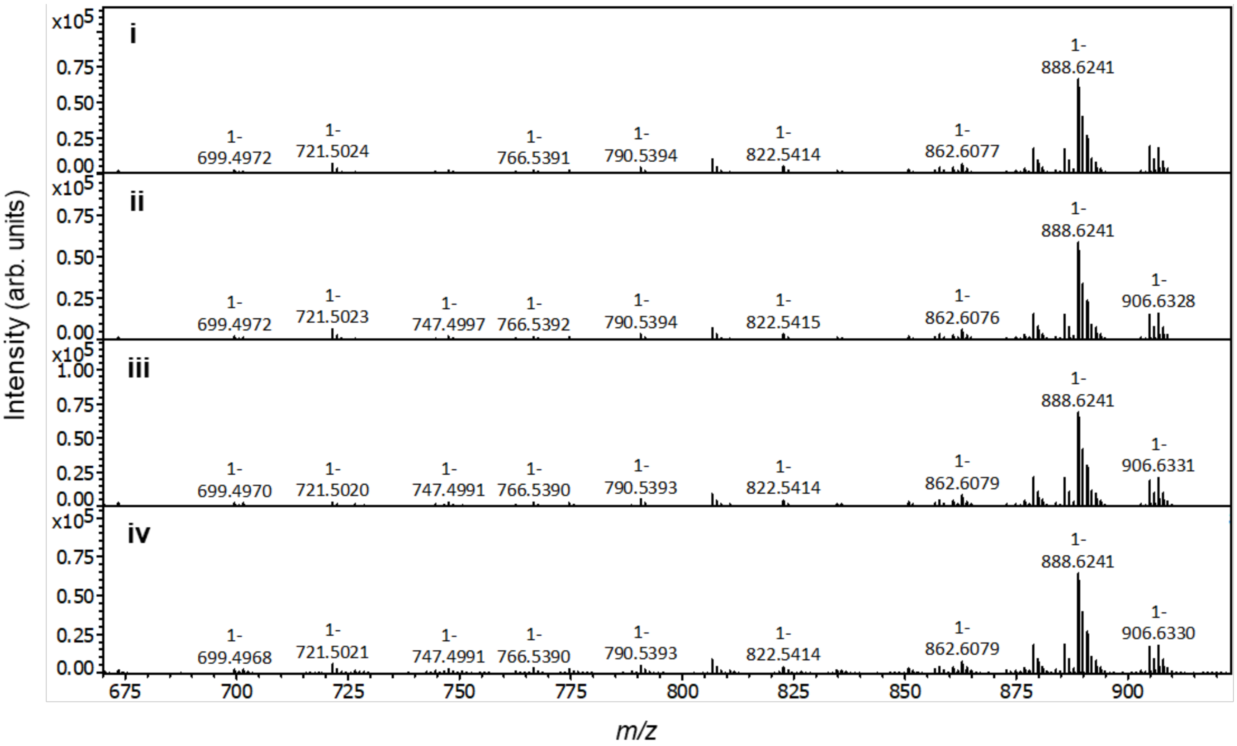

Supplementary Fig. 4. Lipid MS profiles of stressed brain tissue sections. Representative MSI (timsTOF flex, operated in qTOF mode) average spectra (*m/z* 675-920) for brain slices of 60 weeks-old ARSA-/- mice following treatment under conditions presented in **Supplementary Fig. 3**. **(i)** standard workflow where the samples are stored at -80 °C after sectioning and before matrix deposition, **(ii)** the specimen is kept at room temperature (RT) and standard pressure (SP) for about 30 min, **(iii)** the entire tissue section (dashed black box) is exposed to MIR radiation for 15 min in sweep scan mode and kept at RT and SP for in total of about 30 min, and **(iv)** where a defined 1.2 mm x 1.2 mm region of the tissue sections (dashed white box) is exposed to MIR radiation for 15 min at a constant wavenumber of 1656 cm^{-1}. Intensity scale is identical for **(i)-(iv)**. No lipid alterations were observed. Source data is provided as a Source Data file.

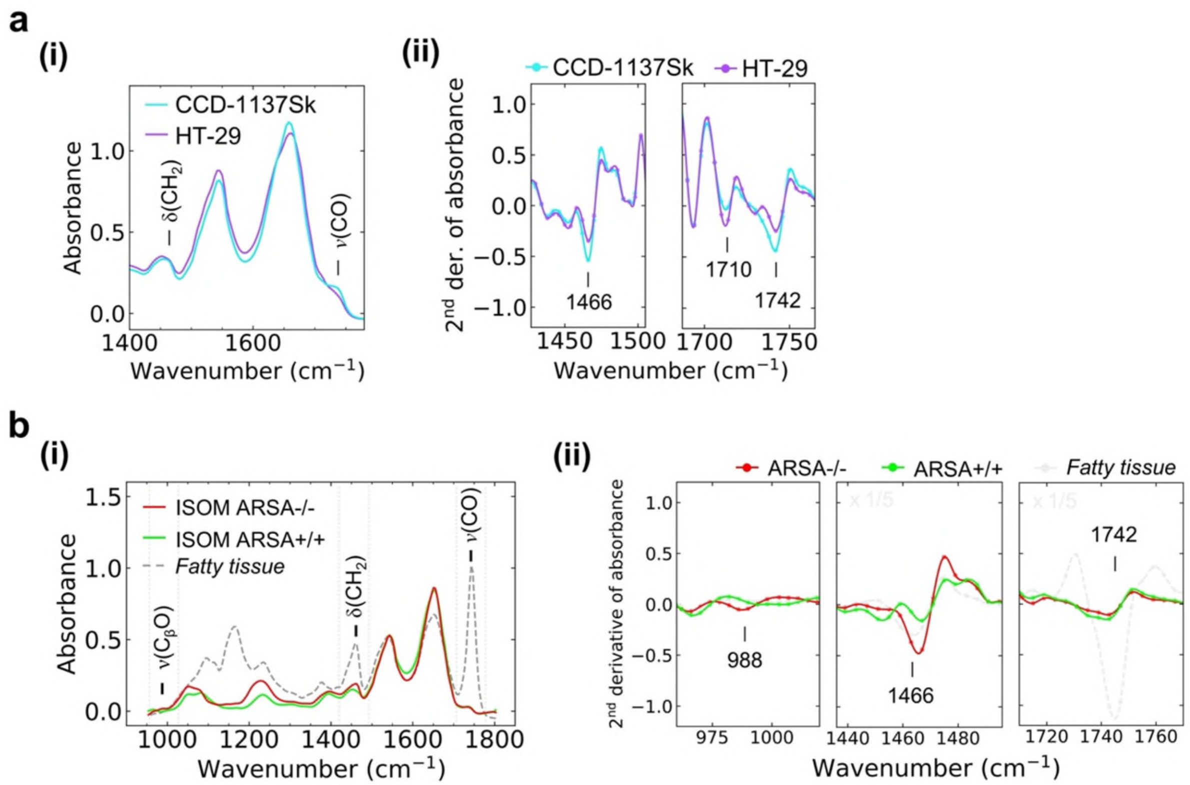

Supplementary Fig. 5. Mid-infrared spectral data for biculture spheroids and ARSA-/- mouse kidney. **a, (i)** Mean cell type-specific QCL-based MIR absorbance spectra (Hyperion II ILIM, 20x objective) for CCD-1137Sk fibroblasts and HT-29 colon cancer cells in spheroids. **(ii)** Lipid-associated bands at 1466 cm^{-1} and 1740 cm^{-1} are reduced in HT-29 cells. 2nd derivative of the mean absorbance spectra for 1466 cm^{-1} and 1740 cm^{-1} demonstrates that the transition (1710 cm^{-1}) between the amide I and ester bands is discriminative for the two cell lines. Data points (dots) and cubic interpolation (line) are shown. **b**, Mean absorbance spectra **(i)** and 2nd derivative of absorbance **(ii)** (Hyperion II ILIM, 3.5x objective) of the ISOM and IMP region as in **Fig. 1g and h**. In addition, the grey dotted line corresponds to the absorbance spectra of a tissue region of high fat content present within the kidneys showing partial overlap with discriminant features of the sulfatide fingerprint, e.g. at 1466 cm^{-1}. Dashed vertical lines represent the spectra regions highlighted for the 2nd derivative data. In **(ii)**, data points (dots) and cubic interpolation (line) are shown. The significance of the observed sulfatide accumulation in the kidney is further highlighted in the (semi-)quantitative analysis provided in **Supplementary Fig. 13c and 13d**, where the distributions of the signal intensities are presented in Box-plots or histograms. Note: Assuming that a given spectral band can be described by a Gaussian, there is a linear relationship between the amplitude of the absorption band and the amplitude of the trough of the 2nd derivative of the absorption. Thus, the significance can be considered as similar for both data representations. Source data is provided as a Source Data file.

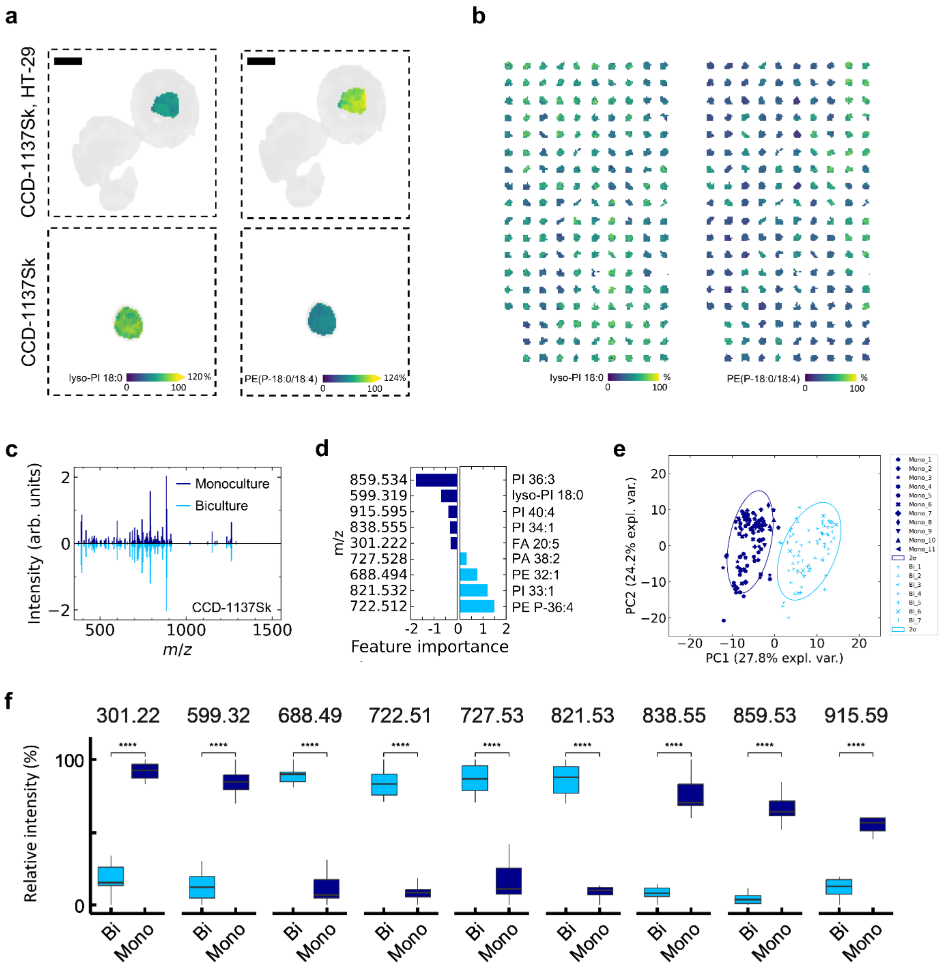

Supplementary Fig. 6. Profiling of fibroblasts in mono- and biculture spheroids. **a**, QCL-based MIR imaging microscopy-guided TIMS-MSI-derived ion images (timsTOF fleX) for *m/z* 599.321 (lyso-PI 18:0[M-H]$^-$) and *m/z* 722.514 (PE P-36:4[M-H]$^-$; both within a ±10 ppm mass window) in monoculture fibroblast (MCF) CCD-1137Sk spheroids and the core fibroblast region of a biculture spheroid (BCF) containing of CCD-1137Sk and HT-29 colon cancer cells. Both *m/z* values are part of a discriminative feature list for monoculture versus biculture fibroblasts. Scale bar, 200 µm. **b**, Overview of ion images for *m/z* 599.321 (lyso-PI 18:0[M-H]$^-$) and *m/z* 722.514 (PE P-36:4[M-H]$^-$) from CCD-1137Sk cells from n=7 wells for BCF and n=11 wells for MCF. **c**, Butterfly plot of the mean intensity from in total 105 MCF- and 72 BCF spheroid sections**. d,** Machine learning-based feature extraction (LASSO (Least Absolute Shrinkage and Selection Operator) regression). Feature importance reveals discriminative *m/z* values between mono- and biculture fibroblasts. To avoid possible elimination of isobaric peaks, we did not implement an isotopic filtering algorithm. **e,** Principal component analysis (PCA) of MSI data distinguishes MCF (dark blue) and BCF (cyan). Spheroid sections from individual spheroids grown in n=11 wells for MCF and n=7 wells for BCF are marked with individual symbols. **f,** Boxplots of relative signal intensities for the extracted *m/z* features. ****Benjamini-Hochberg-adjusted p-value of < 0.001. Source data is provided as a Source Data file.

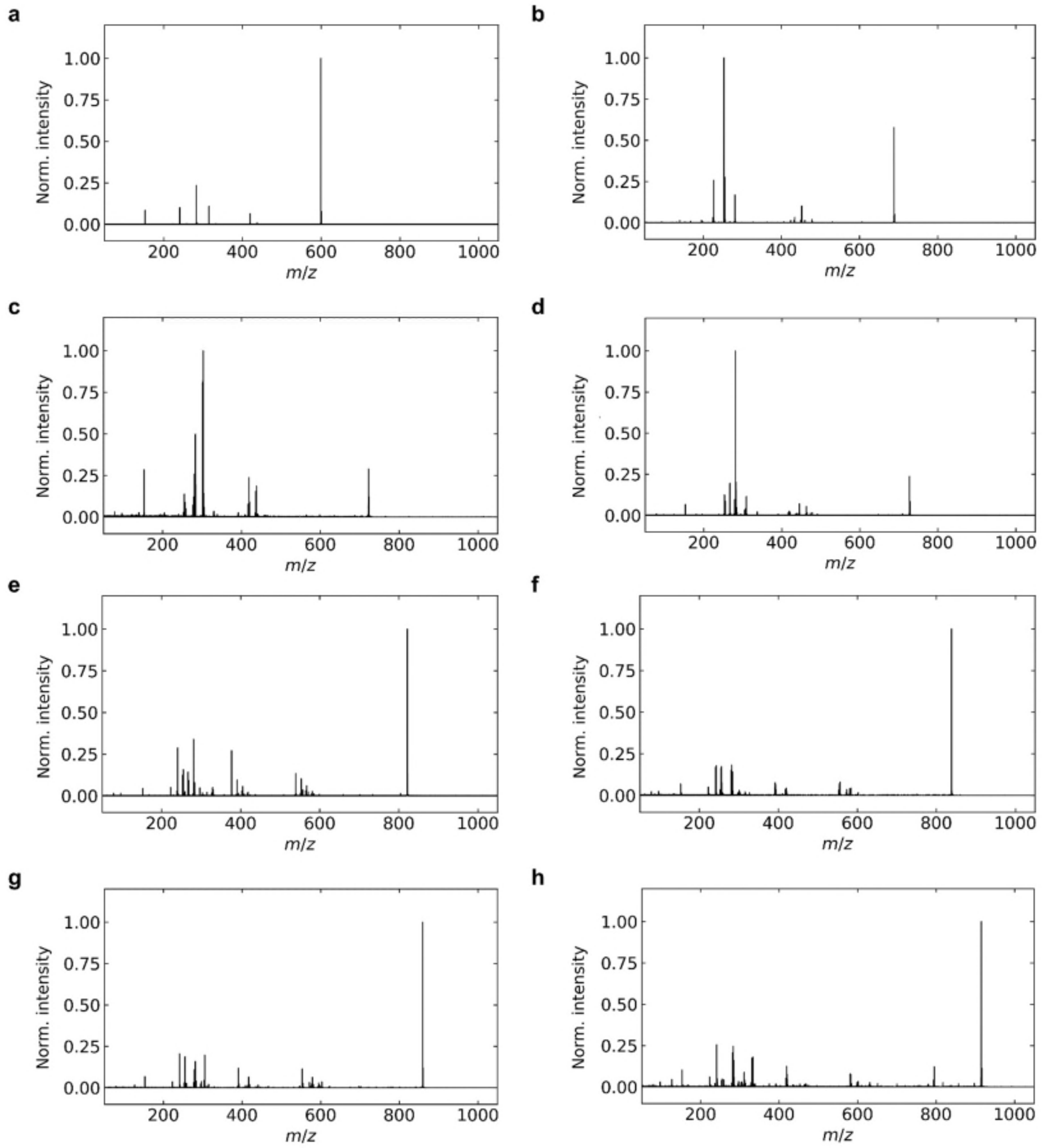

Supplementary Fig. 7. QCL-based MIR imaging microscopy-guided imaging parallel reaction monitoring with parallel accumulation serial fragmentation (iprm-PASEF)-MS^2 spectra of *m/z* features discriminating between MCF and BCF. All MS^2 spectra were recorded on a timsTOF fleX. **a,** *m/z* 599.317 (lyso-PI 18:0$[M-H]^-$) isolated at $1/K_0$ = 1.171 Vs/cm² and fragmented with -40.0 eV. **b,** *m/z* 688.489 (PE 32:1$[M-H]^-$) at $1/K_0$ = 1.281 Vs/cm², fragmented -40.0 eV. **c,** *m/z* 722.511 (PE P-36:4$[M-H]^-$) at $1/K_0$ = 1.317 Vs/cm², fragmented -43.4 eV. **d,** *m/z* 727.527 (PA 38:2$[M-H]^-$) at $1/K_0$ = 1.330 Vs/cm², fragmented -44.5 eV. **e,** *m/z* 821.517 (PI 33:1$[M-H]^-$) at $1/K_0$ = 1.416 Vs/cm² and fragmented with -53.5 eV. **f,** *m/z* 838.541 (PI 34:1$[M-H]^-$, $^{13}C_3$) at $1/K_0$ = 1.427 Vs/cm², fragmented -54.5 eV. **g,** *m/z* 859.530(PI 36:3$[M-H]^-$) at $1/K_0$ = 1.438 Vs/cm², fragmented -55.3 eV. **h,** *m/z* 915.593 (PI 40:4$[M-H]^-$, $^{13}C_2$) at $1/K_0$ = 1.494 Vs/cm², fragmented -59.1 eV. Detailed fragment ion identifications can be found in **Supplementary Tables 2-9**. Source data is provided as a Source Data file.

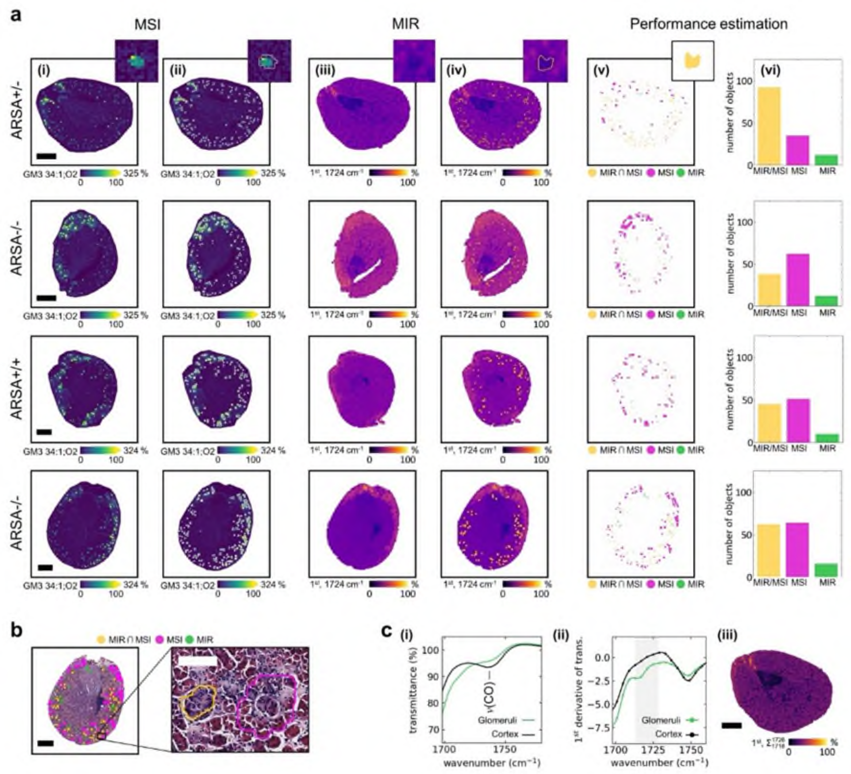

Supplementary Fig. 8. Focused segmentation of glomeruli-containing ROIs in kidney. **a,** Comparison of glomeruli-containing kidney regions in ARSA+/+ (60 weeks), ARSA+/- (12 weeks) and ARSA-/- mice (12 and 60 weeks) by TIMS-MSI (timsTOF fleX) and QCL-based MIR imaging microscopy (Hyperion II ILIM, 3.5x objective): **(i)**, ion image of *m/z* 1151.709 (GM3 34:1;O2[M-H]$^-$), ±10 ppm mass window; **(ii)**, Molecular probabilistic mapping (MPM) hotspot[5] for ganglioside GM3 34:1;O2 to aid probabilistic MSI segmentation of glomeruli; **(iii)**, Mid-infrared image at 1724 cm^{-1} (selected from full spectrum of fingerprint region; 1st derivative of transmittance, Hyperion II, 3.5x objective); **(iv)**, assignment of ROIs by QCL-MIR imaging-based detection of glomeruli; **(v)**, Comparison of probabilistic MSI-MPM-based (magenta) and QCL-MIR imaging-based (green; or both modalities: yellow) segmentation of glomeruli-containing kidney regions. Parameters for QCL-MIR imaging-based identification were optimized to yield high ratios between the numbers of objects identified in both modalities vs. QCL-based MIR imaging alone (see method section). Note that identification of glomeruli-containing ROIs by QCL-MIR imaging can be hampered by high fat content in tissue causing a dominant peak at 1740 cm^{-1} (C=O vibrational band) (**Supplementary Fig. 5**) that limits spectral assignment. 40-80 glomeruli per tissue section were recognized by both modalities. Scale bars, 1 mm. **b**, Hematoxylin and eosin (H&E)-stained histological image of the ARSA-/- (60 weeks) section from **a**. Example regions identified as glomeruli-containing by MSI (magenta) and by both modalities (yellow) are superimposed. Scale bars, 1 mm. **c**, Mean transmittance **(i)** (Hyperion II ILIM, 3.5x objective) and its 1st derivative **(ii)** at around 1720 cm^{-1}. Data points (dots) and cubic interpolation (line) are shown. The grey area highlights a distinct spectral region used to discriminate the glomerular from cortex region, as indicated by the mean MIR image **(iii)**. Scale bars, 1 mm. Source data is provided as a Source Data file.

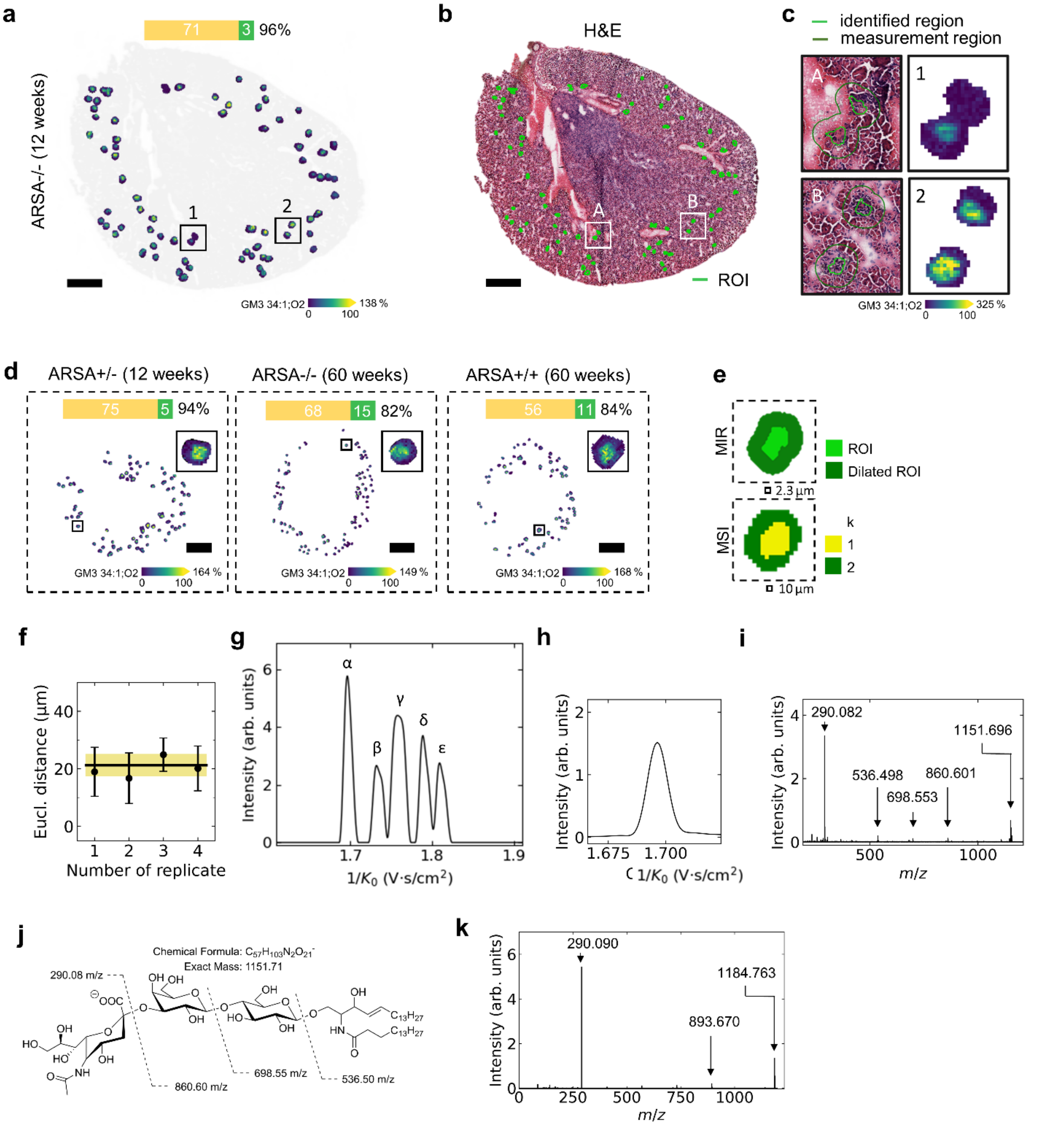

Supplementary Fig. 9. QCL-based MIR imaging microscopy-guided MSI of glomeruli-containing regions in kidney. **a,** QCL-MIR imaging-guided TIMS-MSI (timsTOF fleX) data for *m/z* 1151.708 (GM3 34:1;O2[M-H]⁻, ±10 ppm mass window), and overlay on H&E-stained histological image (**b**). Number of QCL-MIR imaging-segmented regions where characteristic glomeruli gangliosides[6] were identified by MSI (yellow part of the bar chart) and the number of regions below the threshold set for MSI signal intensity (green part), namely 20% of the maximum signal intensity of *m/z* 1151.708 (GM3 34:1;O2[M-H]⁻). Scale bars, 600 µm. **c,** Magnified examples from **a** and **b**. **d,** Example data for tissue

sections from n=3 different mice (wild-type, heterozygotes, and knock-out). Scale bars, 1 mm. **e,** Comparison of dilated (dark green) QCL-MIR imaging ROI (light green; top) and MSI-feature-based clustering (k=2; bottom). **f**, Weighted mean Euclidean distance and internal error[7] (21±4 μm) between centers-of-gravity of QCL-MIR imaging-defined ROI and glomeruli cluster (MSI; yellow) for n=4 different technical replicates. The uncertainties for each data point are expressed as standard deviation. **g**, Extracted ion mobilograms (EIM) for five gangliosides α: GM3 34:1;O2[M-H]$^-$, β: GM3 36:1;O2[M-H]$^-$, γ: GM3 38:1;O2[M-H]$^-$, δ: GM3 40:1;O2[M-H]$^-$, ε: GM3 42:1;O2[M-H]$^-$ identified by subsequent iprm-PASEF MS^2 analysis. **h**, EIM for GM3 34:1;O2[M-H]$^-$ (343.8 Å^2). **i**, iprm-PASEF MS^2 spectrum of *m/z* 1151.696 (GM3 34:1;O2[M-H]$^-$). **j**, Chemical structure of GM3 34:1;O2[M-H]$^-$ including fragment assignment of **i**. **k**, iprm-PASEF MS^2 spectrum of *m/z* 1184.763 (GM3-d5 36:1;O2[M-H$^-$) obtained from a commercially available reference standard compound. Similar fragment ions for the sialic acid moiety at *m/z* 290 is observed, other fragment ions differ by 33 Da, corresponding to the mass difference compared to **i**. Source data is provided as a Source Data file.

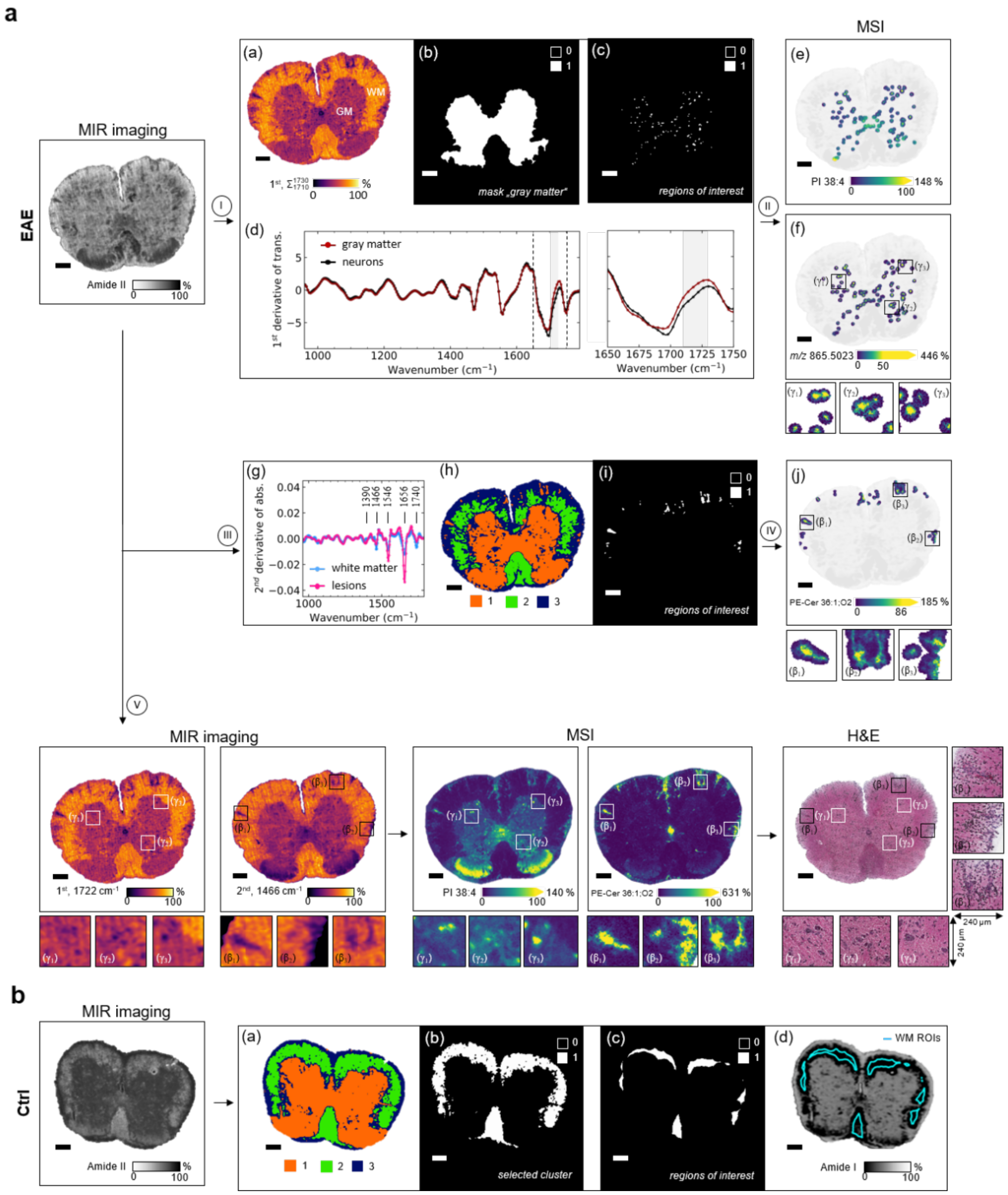

Supplementary Fig. 10. Multimodal assessment of multiple-sclerosis-like mouse spinal cord sections and modelled ion images for the QCL-MIR imaging-guided MSI workflow. **a**, Spinal cords from mice displaying experimental autoimmune encephalomyelitis (EAE), an experimental model of human multiple sclerosis, were investigated by QCL-MIR imaging-guided MSI. Upper part: **(I)** Workflow used for (II) QCL-MIR imaging-guided MSI of <u>single neurons</u> (as identified by corresponding H&E staining): (a) mean MIR ion image at 1740 cm^{-1} (Hyperion II ILIM, 15x objective, 1st derivative of trans-mittance), (b) binary mask of spinal cord gray matter, (c) QCL-MIR imaging-defined dilated single neuron-ROIs used for the MSI data acquisition, (d) 1st derivative spectra for 10 selected neurons and the gray matter region. Data points (dots) and cubic interpolation (line) are shown. Highlighted region is indicated by dashed vertical lines. The grey area highlights a distinct spectral region used to generate the mean MIR image. **(II)** (e) and (f) ion images of *m/z* 885.549 (PI 38:4$[M\text{-}H]^-$) and *m/z* 865.5023 (putatively PG 44:12),

±10 ppm mass window. **(III)** Workflow used for (IV) QCL-MIR imaging-guided MSI of multiple sclerosis-like lesions. (g) Comparison of 2nd derivative spectra between lesions and white matter region. Data points (dots) and cubic interpolation (line) are shown, respectively. Spectral band used for segmentation (h) are highlighted. (i) ROIs are selected based on the segmented data and used to guide the MSI data acquisition, **(IV)** (j) ion image of *m/z* 687.5458 (PE-Cer 36:1;O2[M-H]$^-$, ±10 ppm mass window). **(V)** Comparison of the spinal cord morphology for different imaging modalities recorded on the same tissue section. Scale bars, 240 µm. **b**, Workflow used for QCL-MIR imaging-guided MSI of spinal cord control sections from mice. (a) Segmentation is performed as in **a** (g, h) followed by region selection (b) and erosion (c). ROIs are displayed on a MIR image at amide I wavenumber. Scale bars, 240 µm. Source data is provided as a Source Data file.

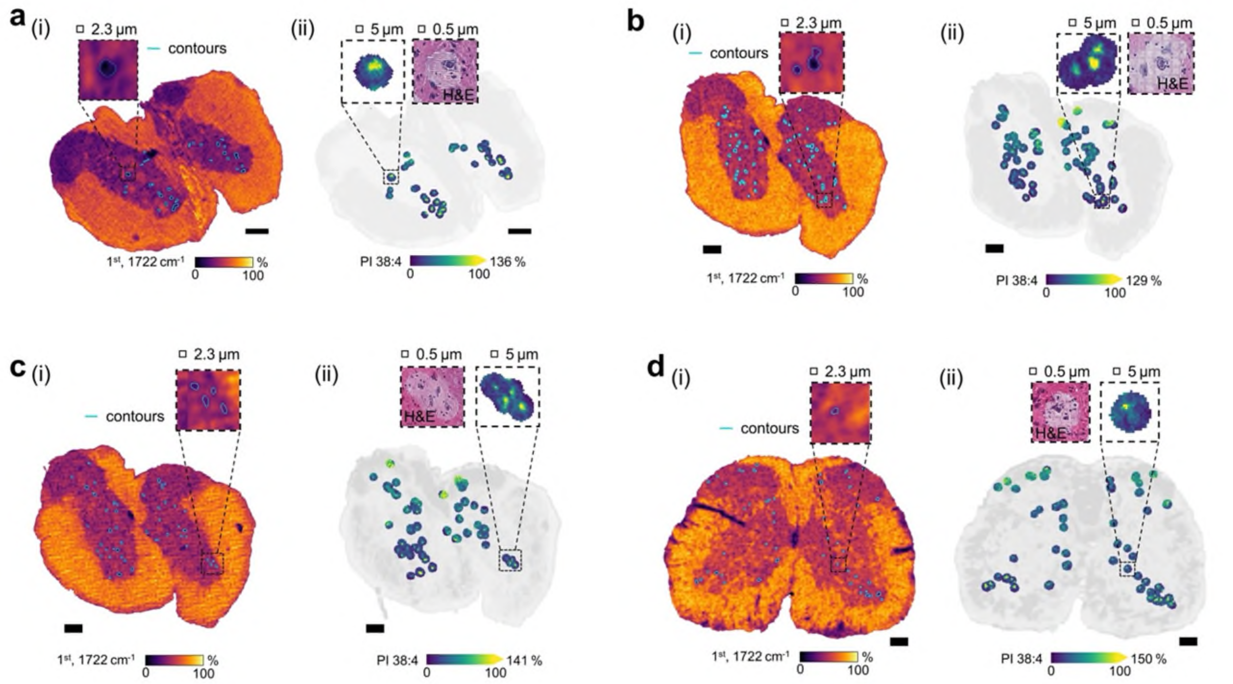

Supplementary Fig. 11. QCL-based MIR imaging microscopy-guided MSI of single neuron-regions in spinal cord tissue sections. **a-d,** (i) Four examples of MIR images at 1722 cm^{-1} with highlighted region of neuron signals (H&E-confirmed; selected from full spectrum of fingerprint region; 1st derivative of transmittance signal, Hyperion II ILIM, 15x objective, binning 2x2), (iii) QCL-MIR imaging-guided TIMS-MSI (timsTOF fleX) data for *m/z* 885.549 (PI 38:4[M-H]$^-$, ±10 ppm mass window). ROIs are indicated by cyan contours in the MIR image. Highlighted are post-MALDI MSI H&E-stained histological image with ablated measurement areas. Scale bars, 200 µm.

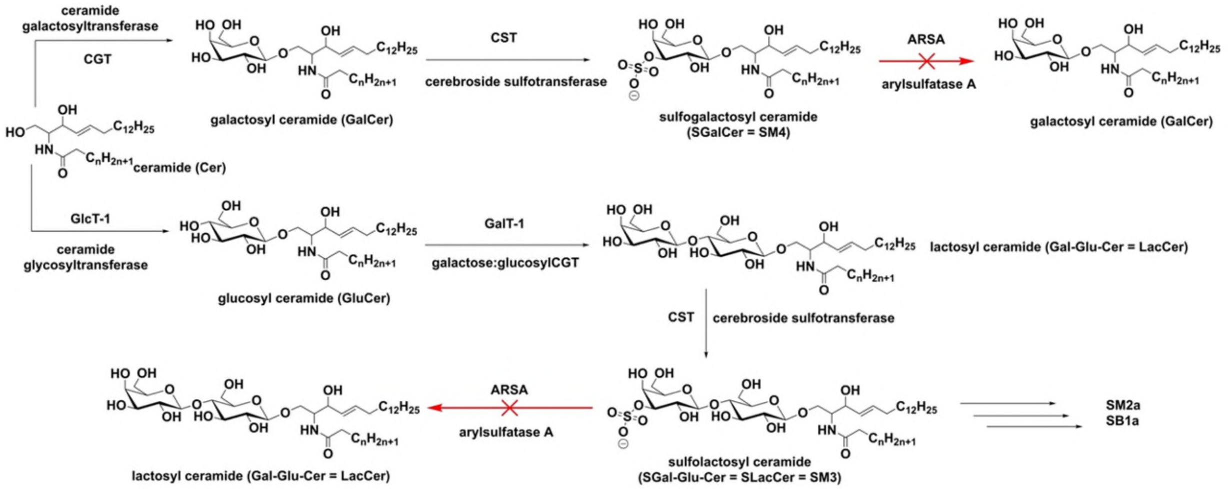

Supplementary Fig. 12. Biosynthesis and degradation of sulfo-glycosphingolipids in the ARSA-/- mouse model of human metachromatic leukodystrophy (MLD). Sulfatide biosynthesis utilizes ceramides for initial enzymatic β-glycosidic linkage of a hexose (galactose [Gal] or glucose [Glu]). Subsequent steps involve either the coupling of another hexose (Gal) to obtain lactosyl ceramides (LacCer) or of a sulfate group in the 3*O*-position of Gal to obtain sulfogalactosyl ceramides (SGalCer = **SM4**). For LacCer the sulfate group is coupled to the 3*O*-position of the terminal Gal, leading to sulfolactosyl ceramides (SLacCer = **SM3**). Complex sulfatides like SM2a or SB1a are generated from SM3. Due to the ARSA deficiency in the mouse model, hydrolytic removal of the sulfate group is blocked for SM4 and SM3, thus causing the accumulation of these lipids in multiple organs including kidney and brain.

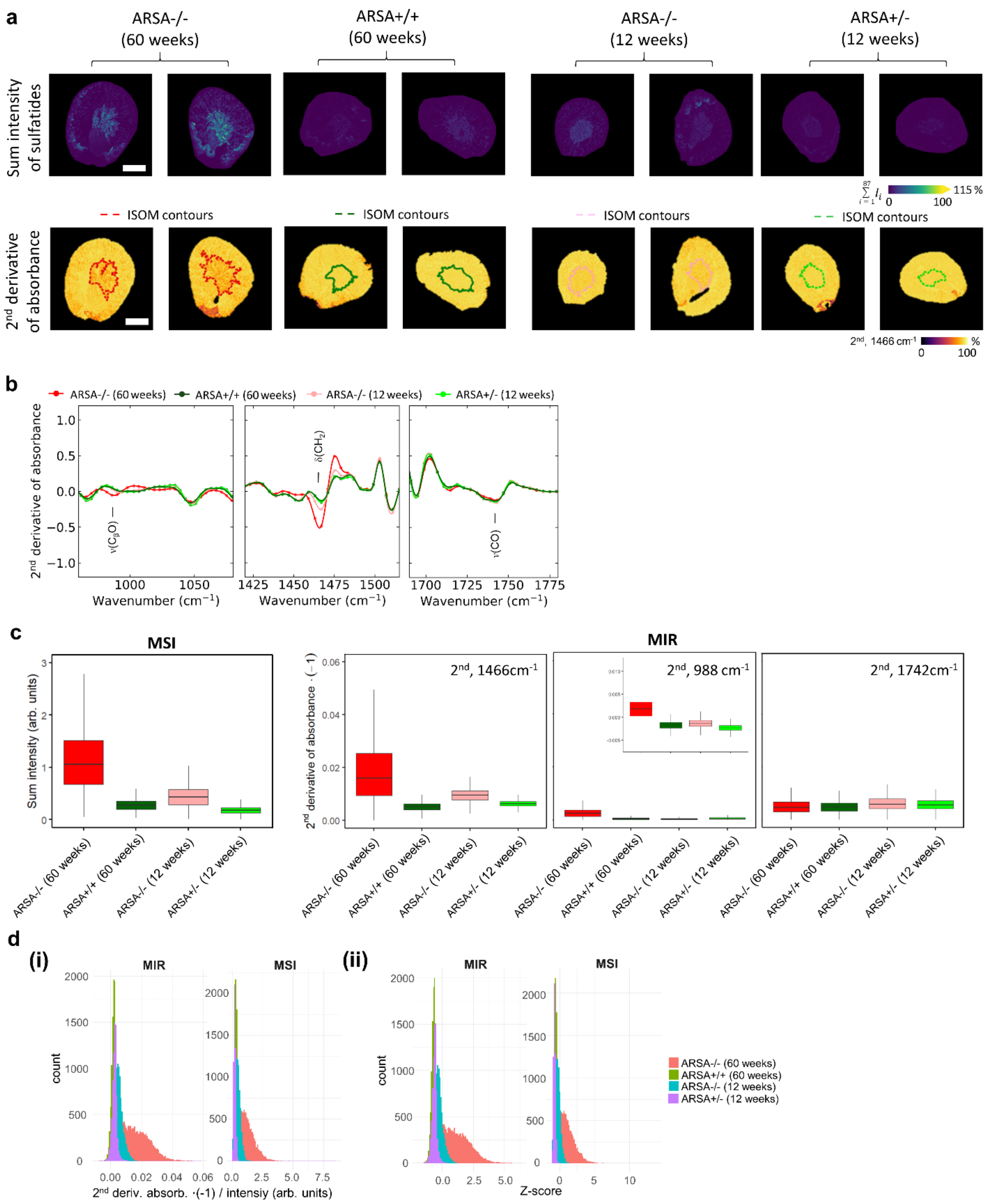

Supplementary Fig. 13. (Semi-)quantitative analysis of sulfatide accumulation in the ISOM by MSI and QCL-based MIR imaging microscopy. **a,** Sum intensity distribution of 87 sulfatides[8] (internal standard normalized) obtained by MSI (timsTOF fleX, operated in qTOF mode) for n=2 ARSA-/- (60 weeks) vs. n=2 ARSA+/+ (60 weeks) and n=2 ARSA-/- (12 weeks) vs. n=2 ARSA+/- (12 weeks)

mice (top). QCL-MIR imaging at the lipid associated band at 1466 cm^{-1} (CH_2 vibration) (bottom). Contours of kidney inner stripe of outer medulla (ISOM) ROI determined by clustering of MSI data (colored solid lines) are highlighted. **b**, Corresponding mean MIR spectra of the 2nd derivative of absorbance. Data points (dots) and cubic interpolation (line) are shown. **c**, Box-plots (n=2) of sum intensity of individual MSI and 2nd derivative of absorbance (linear to the concentration of molecular species[9]) of QCL-MIR imaging data of the ISOM region. QCL-MIR imaging data is presented for the glycolipid-specific spectral band at 988 cm^{-1}, as well as the in general lipid associated but not sulfatide specific bands at 1466 cm^{-1} and 1742 cm^{-1}. Both modalities show consistently (semi-)quantitative accumulation of sulfatides in the ISOM region. As expected, no difference between the different conditions of ARSA is observed for the C=O vibrations. Boxplots indicate median (middle line), 25th and 75th percentile (box) and whiskers (1.5 times the interquartile range). **d**, Corresponding histograms of the data presented in **c (i)** as well as Z-score normalized data for both modalities **(ii)**. Source data is provided as a Source Data file.

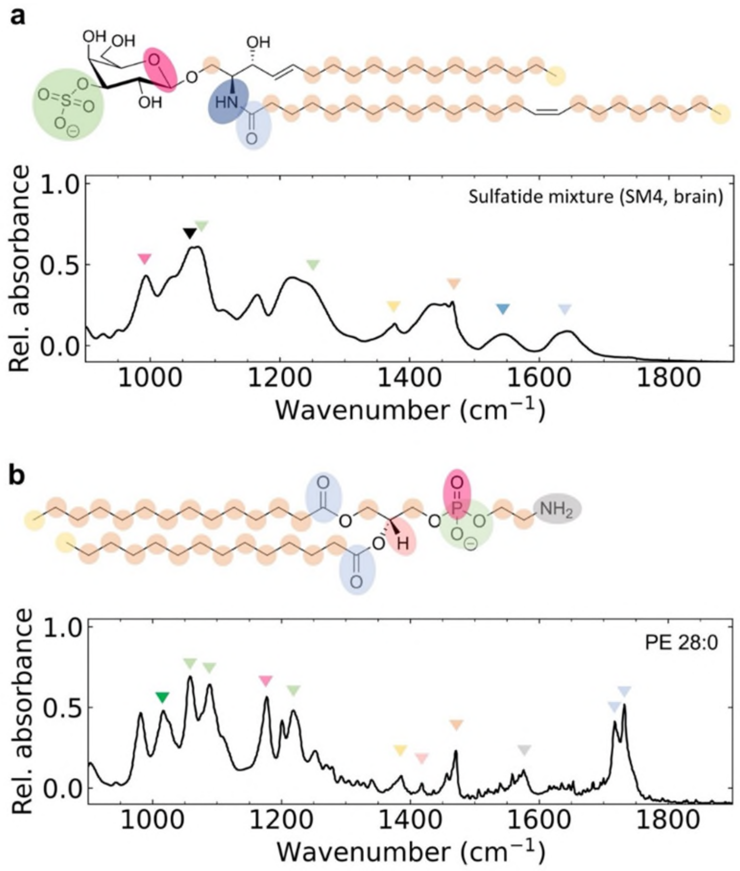

Supplementary Fig. 14. Attenuated total reflectance (ATR) Fourier transform-infrared (FT-IR) fingerprints of a sulfatide mixture and a phosphatidylethanolamine (PE 14:0/14:0). **a**, Molecular features in the FT-IR spectrum of the sulfatide mixture at 1642 cm^{-1} (light blue triangle) and 1541 cm^{-1} (blue triangle) can be attributed to the amide I and amide II band, respectively. Features at 1070 cm^{-1} and 1243 cm^{-1} can be associated to the symmetric and asymmetric stretching vibrations (ν) of the SO_3^- group[10]. A sphingolipid-specific feature present at around 1055 cm^{-1} (black triangle) be associated to a COH stretching vibration[10]. Another prominent feature can be assigned to the C_β-O vibration of the 3-sulfo-galactosyl head group at 988 cm^{-1}. The sulfatide mixture consists to 36% of SM4 24:1, 28% of SM4 24:0, 10% of SM4 22:0, 3% of SM4 20:0, 5% of SM4 18:0 and 18% of other configurations. **b**, Phosphate-associated features can be found at 1015 cm^{-1} (COP stretching vibration), 1088 cm^{-1} (PO_2^- symmetric stretching vibration), 1177 cm^{-1} (PO stretching vibration) and 1227 cm^{-1} (PO_2^- asymmetric stretching vibration). Bending vibrations of CH and NH_2 are located at around 1417 cm^{-1} and 1577 cm^{-1}, respectively. In addition, ester-associated features (stretching vibrations of C=O around 1740 cm^{-1}) are highlighted. Common to both lipid species (phospholipids and sphingolipids) are structures located at 1375 cm^{-1} and 1466 cm^{-1}, which can be assigned to the bending mode (δ) of the CH_2 and CH_3 groups. Data was obtained on a Hyperion II ILIM system in imaging ATR FT-IR mode (20x objective). The spectrum of the sulfatide mixture was normalized relative to the known lipid-associated feature at 2915 cm^{-1} of PE 28:0 attributed to the asymmetric stretching vibration of CH_2. Source data is provided as a Source Data file.

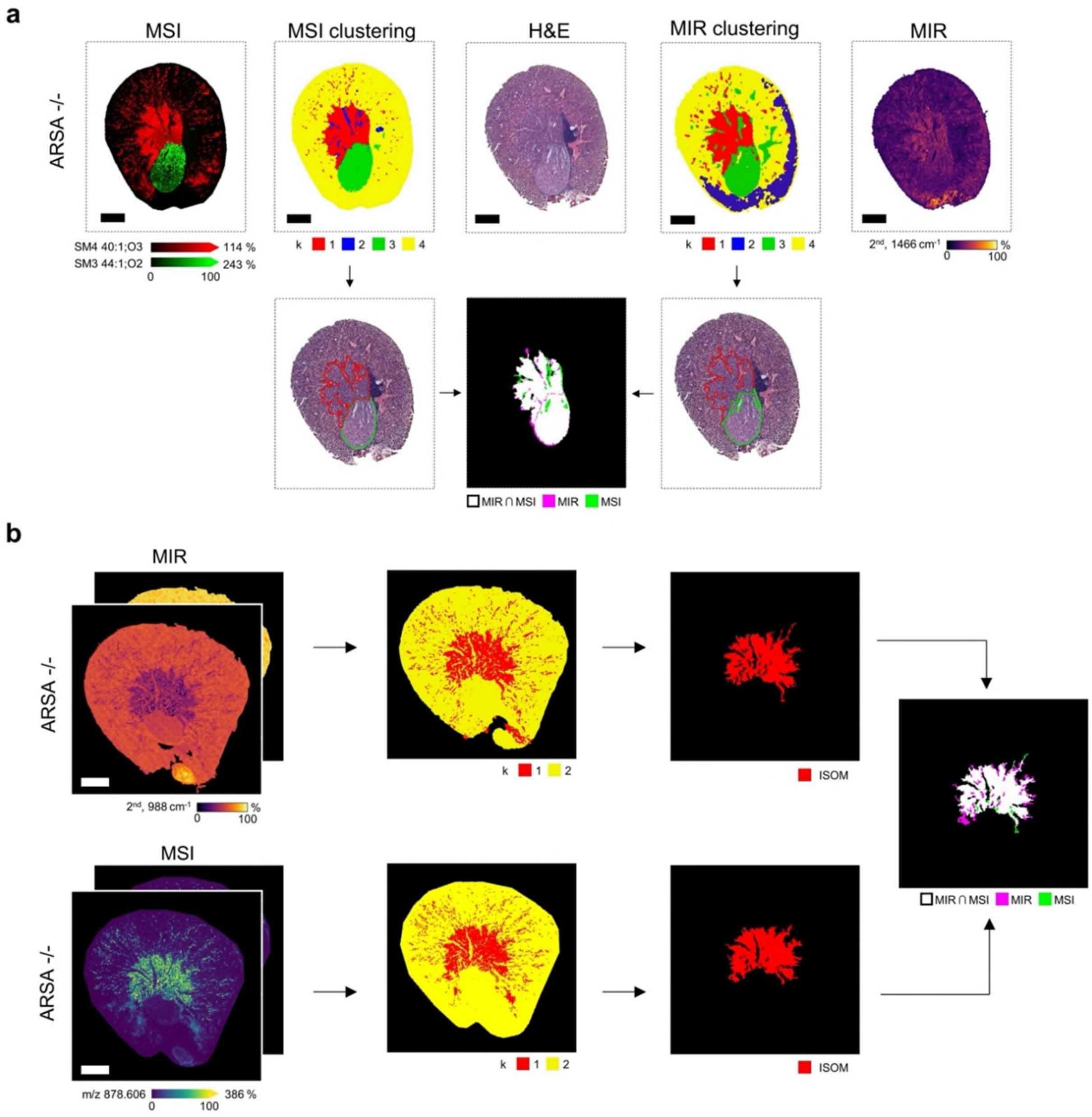

Supplementary Fig. 15. Comparable kidney segmentation with MSI and QCL-based MIR imaging microscopy. **a,** Top row, left: QCL-MIR imaging microscopy-guided TIMS-MSI-derived ion images (timsTOF fleX, both represented within a ±10 ppm mass window) for *m/z* 878.602 (SM4 40:1;O3[M-H]$^-$) and 1080.732 (SM3 44:1;O2[M-H]$^-$) for an ARSA-/- kidney (60 weeks) section; MSI image segmentation based on 60 selected *m/z* features obtained by bisecting k-means clustering (k=4); top row, middle: H&E-stained tissue section, QCL-MIR clustering-based on 5 selected features with k=4; top row, right: MIR image of single lipid-associated band at ν=1466 cm^{-1} (2nd derivative of absorbance, Hyperion II ILIM, 3.5x objective). The blue region in the MIR imaging clustering may indicate tissue with a high fat content. Bottom row: Visualization of the regions of interest (ROIs) for the kidney's inner stripe of the outer medulla (ISOM) and for the kidney inner medulla/papilla (IMP), as defined by MSI or MIR imaging, superimposed on the

H&E-stained image. A comparison of the respective IMP and ISOM regions defined by MSI and QCL-MIR imaging microscopy yielded a Dice-Sorensen coefficient[11] of 93%. Scale bars, 1 mm. **b,** Sulfatide distributions in ARSA-/- mouse kidney (12 weeks old). Top row, left: High-resolution MIR images (2nd derivative data, Hyperion II ILIM, 15x objective) for the lipid-associated bands at 990 cm^{-1} (C_{β}-O vibration of the 3-sulfogalactosyl head group) and 1466 cm^{-1} (CH_2 bending vibration)[10]; middle: corresponding clusters for k-means clustering with k=2; top row, right: selected ISOM segment/ROI. Bottom row, left: Ion images (timsTOF fleX, qTOF mode) acquired with 20 µm lateral step-size and clustering for *m/z* 878.606 (SM4 40:1;O3$[M-H]^-$) and *m/z* 880.612 (SM4 40:0;O3$[M-H]^-$), ±10 ppm mass window). Comparison of the respective ISOM regions based on MSI and QCL-MIR imaging yielded a Dice-Sorensen coefficient of 87%. Scale bars, 1 mm.

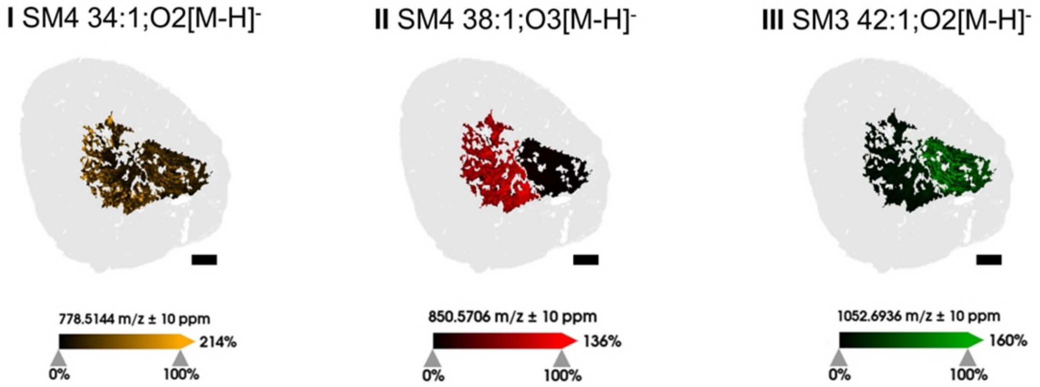

Supplementary Fig. 16. QCL-MIR imaging microscopy-guided TIMS-MSI-derived ion images of the kidney's ISOM and IMP. Ion images (timsTOF fleX) are given for three molecules and conditions: **I** *m/z* 778.5145 (SM4 34:1;O2$[M-H]^-$), showing a similar ion intensity in both regions, **II** *m/z* 850.5720 (SM4 38:1;O3$[M-H]^-$), showing much stronger ion intensity in ISOM, and **III** *m/z* 1052.6925 (SM3 42:1;O2$[M-H]^-$) showing much stronger ion intensity in IMP. All ion images are presented within a mass window of ±10 ppm. Scale bars, 500 µm.

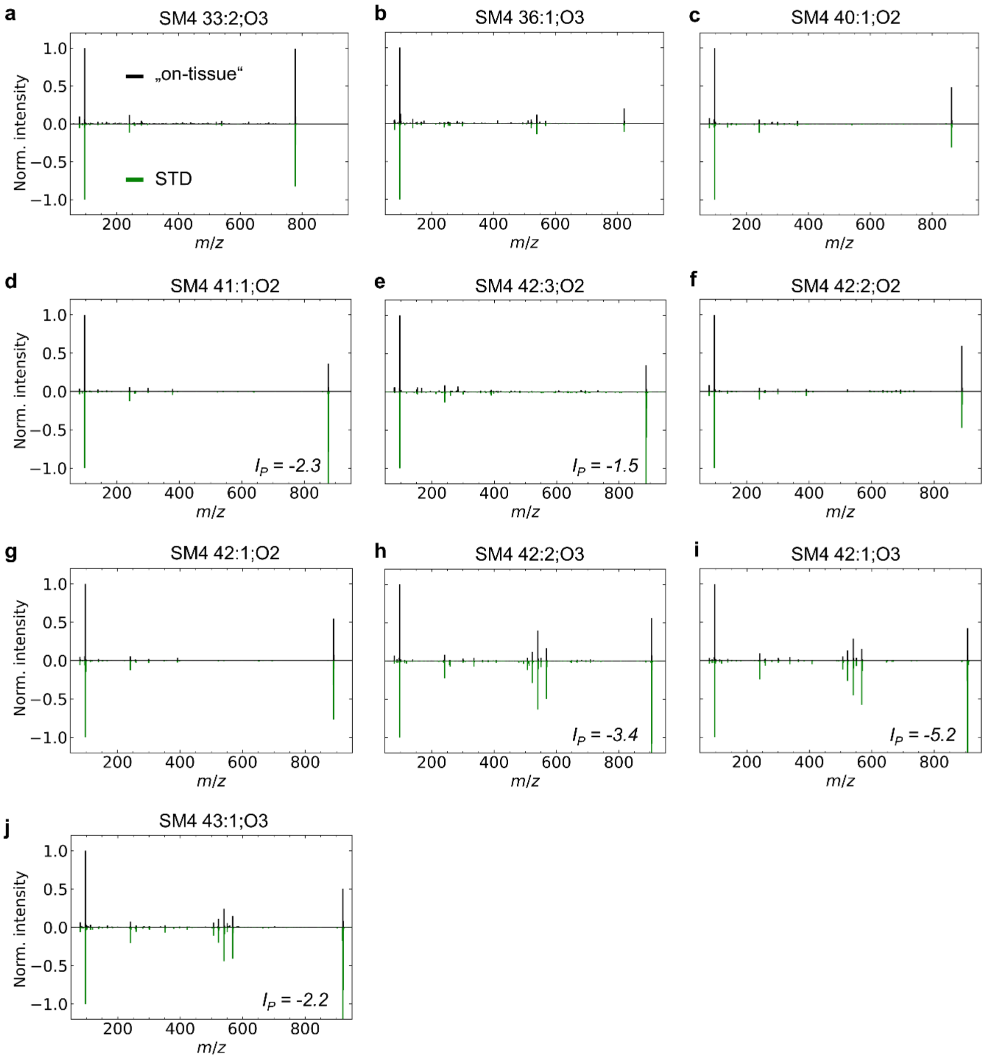

Supplementary Fig. 17. Comparison of iprm-PASEF MS^2 spectra of ten sulfatides obtained from on-tissue analysis (black) vs. commercial standard (porcine) brain sulfatides mixture. All MS^2 spectra were recorded on a timsTOF fleX and normalized to the peak of the HO$_4$S$^-$ fragment at *m/z* 96.96. **a,** *m/z* 778.48 (SM4 33:2;O3[M-H]$^-$) isolated at 1/K_0 = 1.391 Vs/cm² and fragmented with -83.0 eV. **b,** *m/z* 822.54 (SM4 36:1;O3[M-H]$^-$) at 1/K_0 = 1.453 Vs/cm², fragmented -93.5 eV. **c,** *m/z* 862.61 (SM4 40:1;O2[M-H]$^-$) at 1/K_0 = 1.494 Vs/cm², fragmented -96.1 eV. **d,** *m/z* 876.62 (SM4 41:1;O2[M-H]$^-$) at 1/K_0 = 1.476 Vs/cm², fragmented -96.3 eV. **e,** *m/z* 886.62 (SM4 42:3;O2 [M-H]$^-$) at 1/K_0 = 1.515 Vs/cm² and fragmented with -96.7 eV. **f,** *m/z* 888.62 (SM4 42:2;O2[M-H]$^-$) at 1/K_0 = 1.517 Vs/cm², fragmented -96.9 eV. **g,** *m/z* 890.64 (SM4 42:1;O2[M-H]$^-$) at 1/K_0 = 1.525 Vs/cm², fragmented -97.1 eV. **h,** *m/z* 904.62 (SM4 42:2;O3[M-H]$^-$) at 1/K_0 = 1.523 Vs/cm², fragmented -97.1 eV, **i,** *m/z* 906.62 (SM4 42:1;O3[M-H]$^-$) at 1/K_0 = 1.531 Vs/cm², fragmented -97.3 eV, and **j,** *m/z* 920.65 (SM4 43:1;O3[M-H]$^-$) at 1/K_0 = 1.547 Vs/cm², fragmented -97.8 eV. Detailed fragment ion identifications can be found in **Suppl. Figs 20** and **21**. Intensities of the precursor are denoted as I_P. The observed fragments from *on-tissue* and standard mixture (Avanti Polar Lipids) analysis match closely. Source data is provided as a Source Data file.

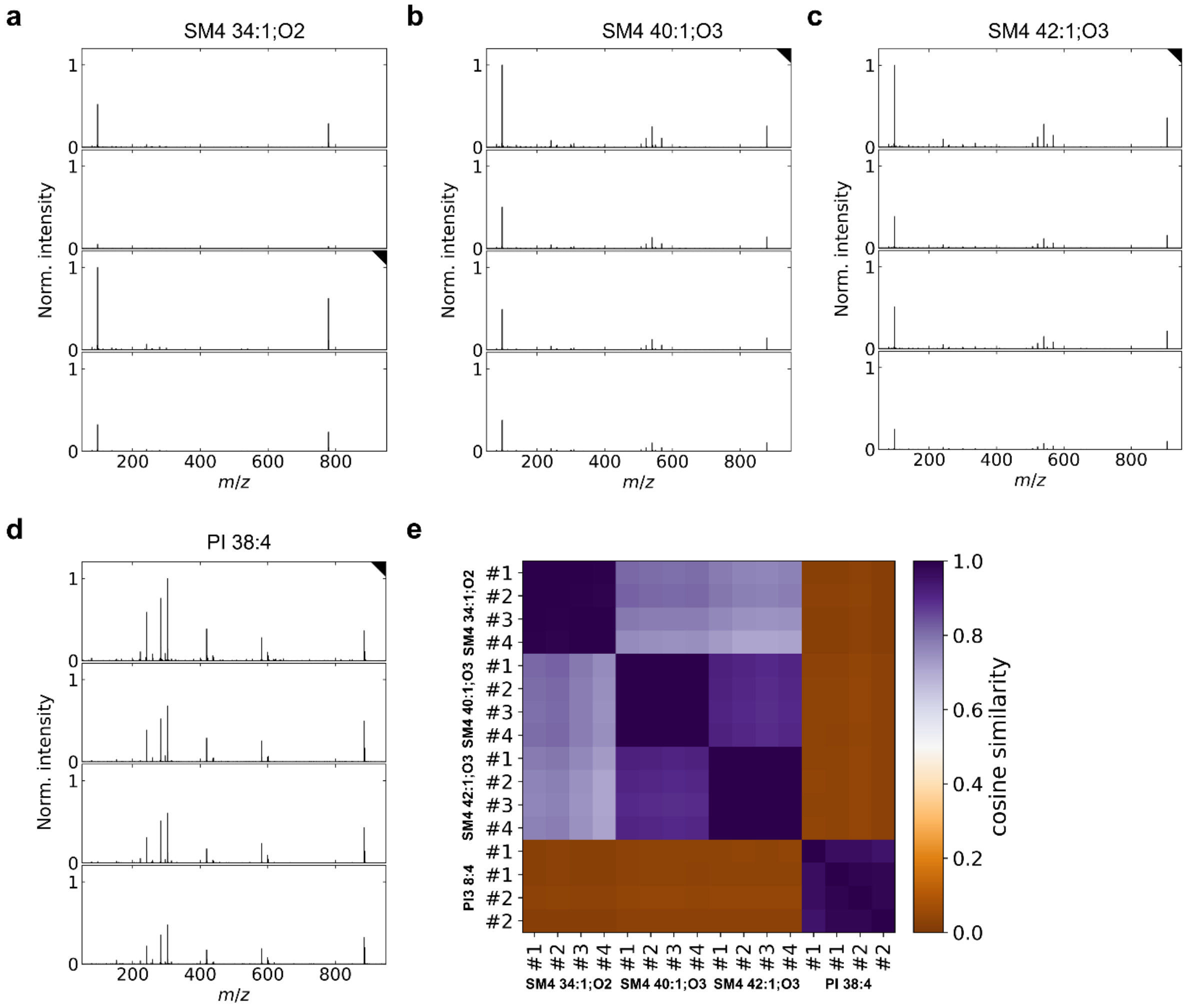

Supplementary Fig. 18. Evaluation of reproducibility for iprm-PASEF MS^2 spectra for n=4 biological replicates. All MS^2 spectra were recorded on a timsTOF fleX. **a,** MS^2 spectra (n=4 biological replicates) for *m/z* 778.51 (SM4 34:1;O2[M-H]$^-$) isolated at $1/K_0$ = 1.390 Vs/cm², fragmented with -84.2 eV. **b,** MS^2 spectra (n=4 biological replicates) for *m/z* 878.60 (SM4 40:1;O3[M-H]$^-$) at $1/K_0$ = 1.475 Vs/cm², fragmented -96.0 eV. **c,** MS^2 spectra for (n=4 biological replicates) for *m/z* 906.62 (SM4 42:1;O3[M-H]$^-$) at $1/K_0$ = 1.531 Vs/cm², fragmented -97.3 eV. **d,** MS^2 spectra for (n=2 biological replicates and n=2 technical replicates) for *m/z* 885.55 (PI 38:4[M-H]$^-$) at $1/K_0$ = 1.476 Vs/cm², fragmented -96.3 eV. Spectra were normalized to the base peak, HO_4S^- for the three sulfatides and FA 20:4 for the PI, of the replicate with the highest signal intensity (marked with black triangle). For data evaluation, MS^2 raw spectra were converted into a continuous representation, with a bin size of 0.05 Da. For detailed structural formulae for sulfatide fragments, see **Suppl. Fig. 20**, for fragments of PI 38:4 see **Suppl. Table 19**. **e,** Heat map of intra- and inter-feature cosine similarity for **a-d** across biological replicates. Highest cosine similarity values across spectra from different biological replicates highlight the reproducibility of the iprm-PASEF MS^2 data. Similarity between the MS^2 spectra of SM4 40:1;O3 and SM4 42:1;O3 is given, since both consist of an α-OH-FA, leading to analogous fragments. Source data is provided as a Source Data file.

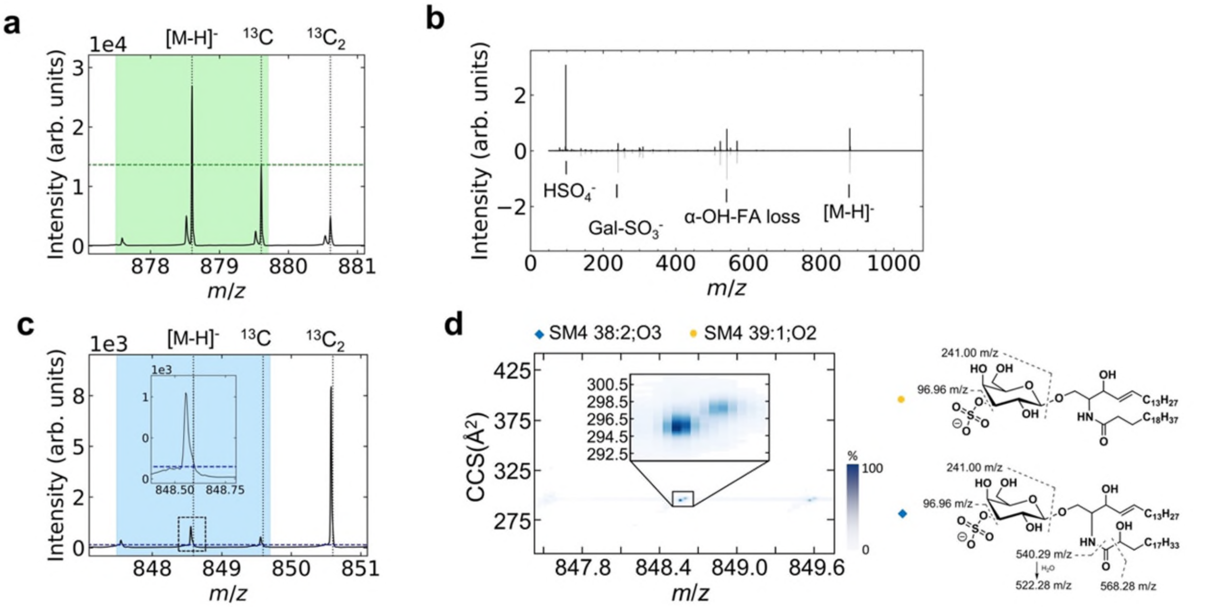

Supplementary Fig. 19. Representative MS1 and MS2 spectra for the sulfatide SM4 40:1;O3[M-H]$^{-}$. **a,** Average MS spectrum (in the range of *m/z* 877-881) obtained by QCL-MIR imaging microscopy-guided MSI (timsTOF fleX) of an ARSA-/- mouse IMP region. The vertical, black dotted lines indicate the theoretical *m/z* values for the monoisotopic mass ([M-H]$^{-}$), the first carbon isotope (^{13}C) and the second carbon isotope ($^{13}C_2$). The green area indicates a typical isolation window of ±1.1 Da. The green horizontal dotted line represents the ion intensity at the position of the theoretical *m/z* value of the first carbon isotope. Within the isolation window, there is no additional *m/z* peak unrelated to SM4 40:1;O3[M-H]$^{-}$ (*m/z* 878.603) with a signal intensity higher than the peak of the first carbon isotope. Hence, the spectrum is considered as non-chimeric. **b,** Butterfly plot of two MS^2 spectra of SM 40:1;O3[M-H]$^{-}$. The black spectrum (top half) was obtained by iprm-PASEF, the grey spectrum (lower half) "conventionally" by MS^2 without prior ion mobility separation ("on-tissue"). Main fragments were assigned as HSO_4^- (*m/z* 96.96), Gal-SO_3^- (*m/z* 241.00), and α-OH fatty acid (α-OH-FA) loss-related. **c**, Example of a chimeric peak: *m/z* 848.557 (SM4 38:2;O3[M-H]$^{-}$) displays an asymmetric peak shape because of non-resolved *m/z* 848.591 (SM4 39:1;O2[M-H]$^{-}$). Precursor isolation window of ±1.1 Da for MS^2 is depicted as light blue area. The blue horizontal dashed line (inset) refers to the intensity value at ^{13}C of [M-H]$^{-}$. **d**, Ion mobility heat map with inset highlighting two SM4 isoforms of *m/z* 848.557, 295.4 Å² (SM4 38:2;O3[M-H]$^{-}$; left peak) and *m/z* 848.591, 297.6 Å² (SM4 39:1;O2[M-H]$^{-}$; right peak). Chemical structures and elucidated fragments for SM4 38:2;O3[M-H]$^{-}$ and SM4 39:1;O2[M-H]$^{-}$ are also shown. Source data is provided as a Source Data file.

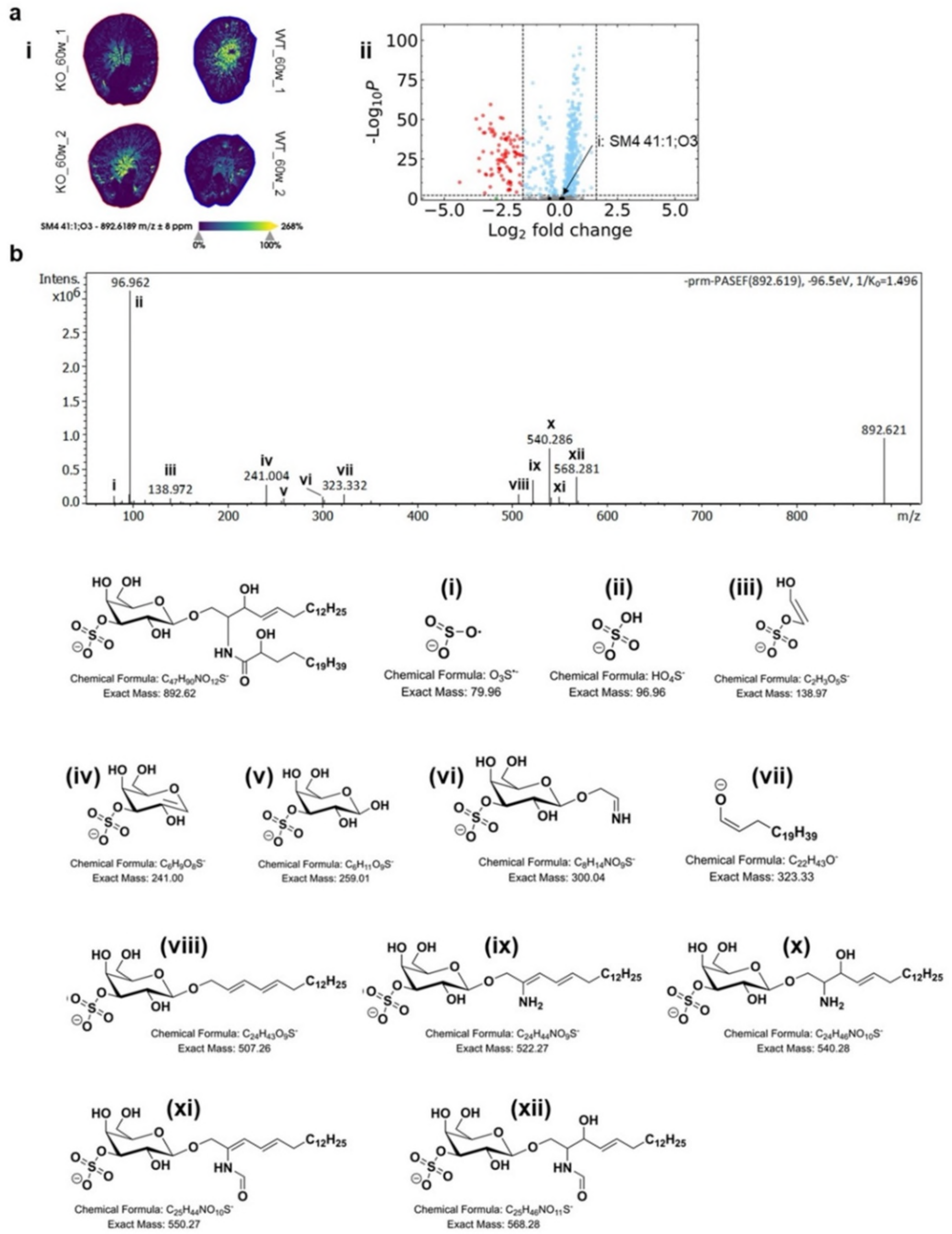

Supplementary Fig. 20. Unequivocal identification of odd-chain sulfatides by QCL-MIR imaging-guided MALDI-TIMS-MSI with iprm-PASEF. **a, (i)** Ion images (timsTOF flex, qTOF mode) of *m/z* 892.619 (SM4 41:1;O3 [M-H]$^-$) for ARSA-/- (KO, left) and ARSA+/+ (WT, right) kidney tissue sections (n=2 biological replicates each). **(ii)** MSI-derived volcano scatter plot suggests the non-significance for the accumulation of odd-chain sulfatides (black dots), in contrast to significant features (red dots). Statistical significance was tested by two-sided standard t-test for n=2 biological replicates. P-values are Benjamini–Hochberg-corrected. **b,** iprm-PASEF-derived MS^2 spectrum of *m/z* 892.621 (SM4 41:1;O3[M-H]$^-$), isolated at $1/K_0$ = 1.496 Vs/cm² and fragmented at -96.5 eV. The structures of the identified fragments are shown below. Source data is provided as a Source Data file.

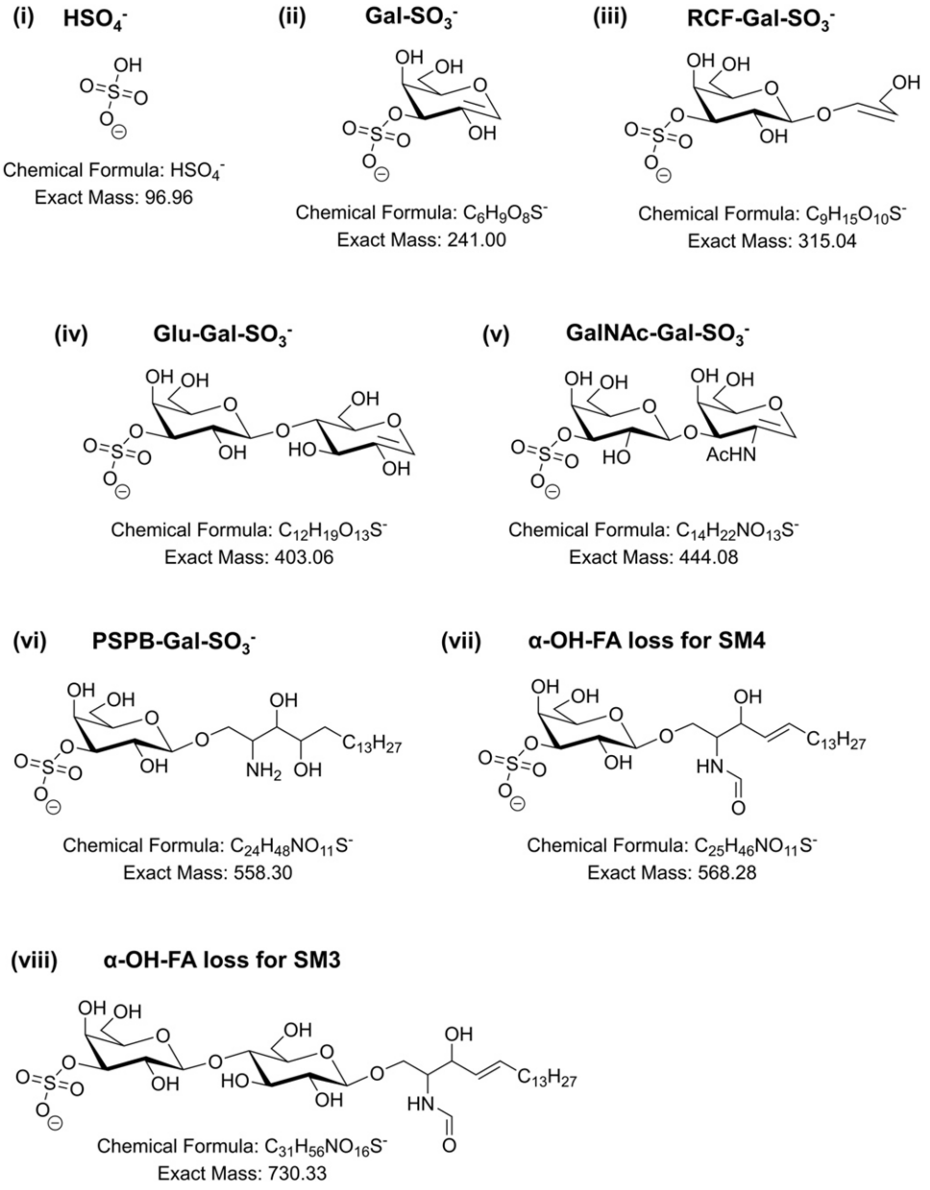

Supplementary Fig. 21. Characteristic sulfatide fragments. Chemical structures of sulfatide fragments: **(i)**, cleavage of the sulfate moiety leads to fragment HSO_4^- at *m/z* 96.96. **(ii)**, cleavage of the Gal-SO_3^- moiety yields fragment at *m/z* 241.00. **(iii)**, ring-cross-fragmentation (RCF) within the glucose part of SM3 leads to fragment at *m/z* 315.04. **(iv)**, cleavage of the entire Glu-Gal-SO_3 moiety forms the fragment at *m/z* 403.06. **(v)**, for SM1b and SB1a, cleavage of GalNAc-Gal-SO_3^- leads to the fragment *m/z* 444.08. **(vi)**, neutral loss of the entire α-OH-FA moiety results in fragment *m/z* 558.30 for sulfatides consisting of a phytosphingoid base (PSPB). **(vii)** and **(viii)**, neutral loss of the α-OH-FA leads to fragments at *m/z* 568.28 and *m/z* 730.33 for SM3 and SM4, respectively.

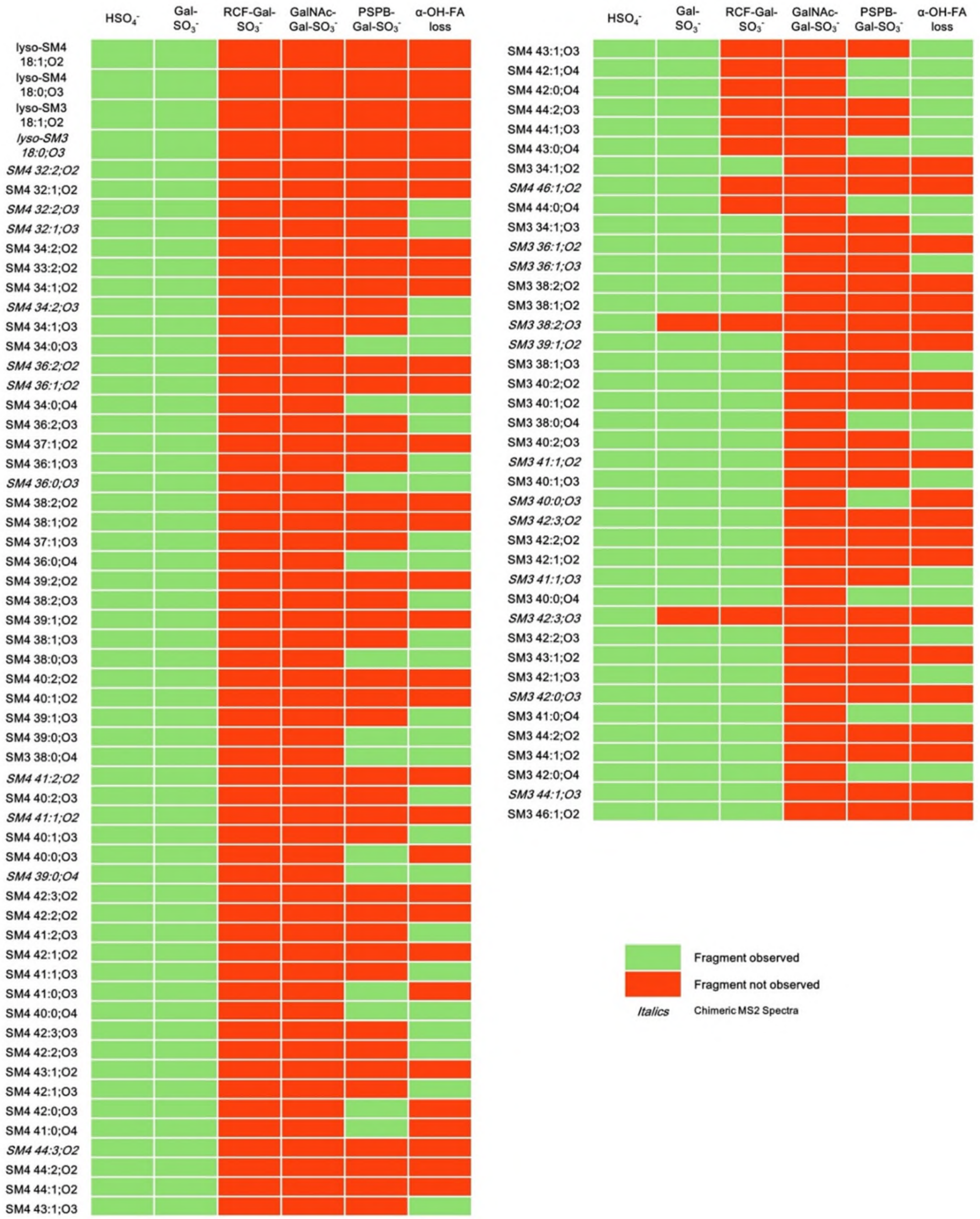

Supplementary Fig. 22. Overview of sulfatide fragments for sulfatide classes SM4 and SM3 observed by QCL-MIR imaging-guided TIMS-MSI and iprm-PASEF analysis. Observed molecules are classified as non-chimeric and chimeric (italic).

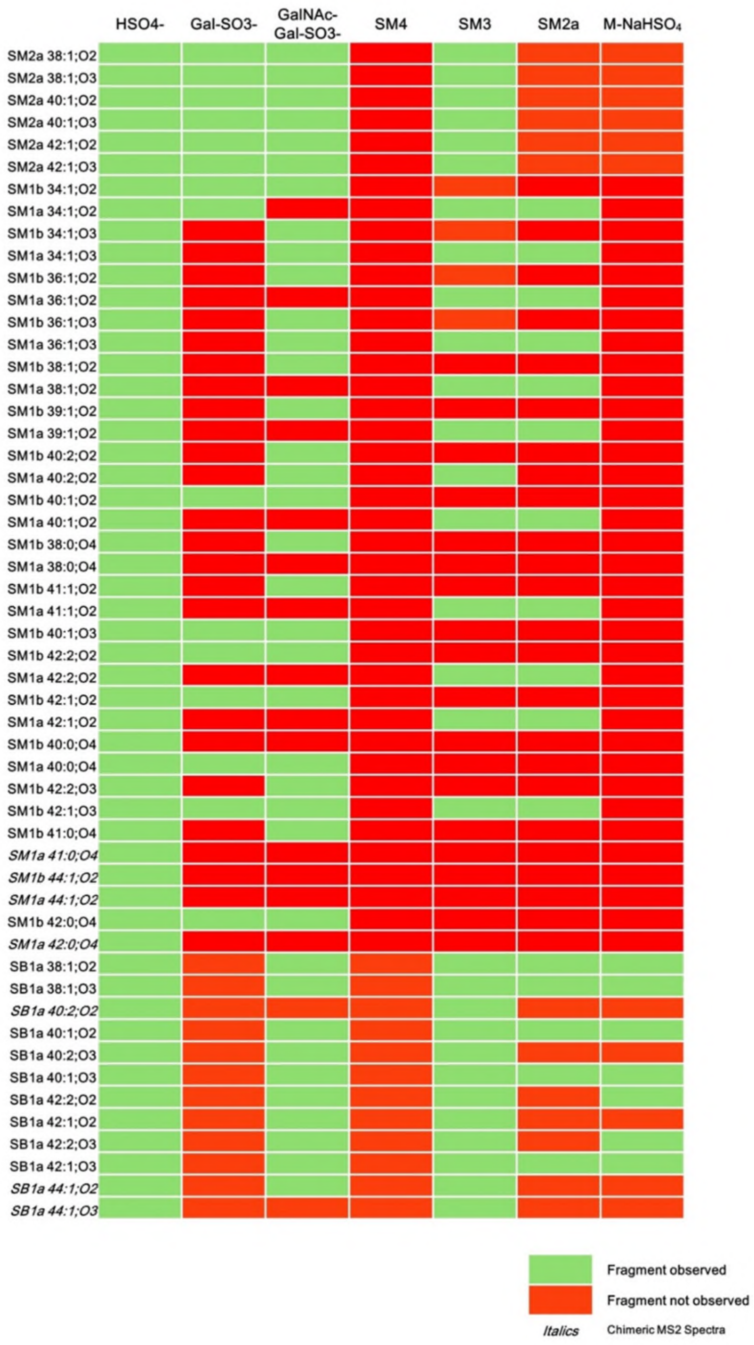

Supplementary Fig. 23. Overview of sulfatide fragments for sulfatide species SM2a, SM1a, SM1b and SB1a observed in QCL-MIR imaging-guided TIMS-MSI and iprm-PASEF analysis. Observed molecules are classified as non-chimeric and chimeric (italic). Note: SB1a are identified as $[M+Na-2H]^-$ adducts.

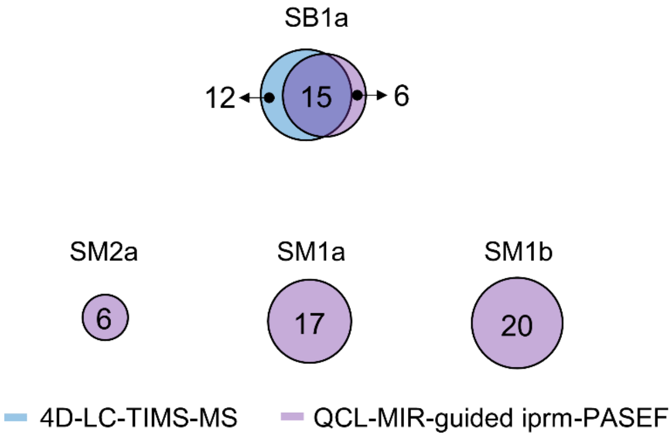

Supplementary Fig. 24. Venn diagram of sulfatide subclasses identified by 4D-LC-TIMS-MS (blue) and MALDI-TIMS-MSI (purple). Comparison of sulfatide identifications for the subclasses SM2a, SB1a, SM1a and SM1b. Note that SB1a isoforms are detected as SB1a$[M-2H]^{2-}$ in LC-TIMS-MS and as SB1a$[M+Na-2H]^{-}$ in MSI. SM1a/b isoforms were only found in TIMS-MSI, as their mass and mobility ranges were not covered in the LC-TIMS-MS setup. Note that the total number of sulfatides is difficult to compare, since the MSI settings were optimized for detection of SM1/SB1 and SM2 sulfatides, which were not detectable with settings used in 4D-LC-TIMS-MS.

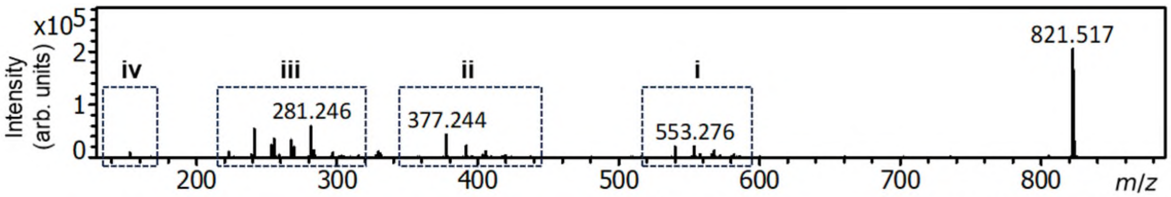

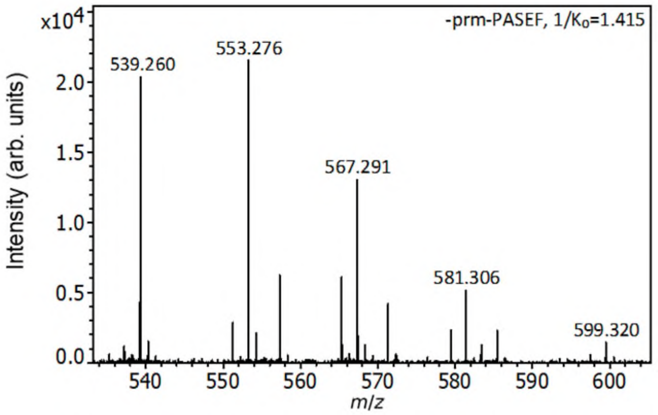

m/z measured	Fragment ion	Sum formula
**821.517**	[M-H]$^-$	$C_{42}H_{78}O_{13}P^-$
**599.320**	[M-15:1ketene-H]$^-$	$C_{27}H_{52}O_{12}P^-$
**597.303**	[M-15:0ketene-H]$^-$	$C_{27}H_{50}O_{12}P^-$
**585.306**	[M-16:1ketene-H]$^-$	$C_{26}H_{50}O_{12}P^-$
**583.286**	[M-16:0ketene-H]$^-$	$C_{26}H_{48}O_{12}P^-$
**581.306**	[M-FA15:1-H]$^-$	$C_{27}H_{50}O_{11}P^-$
**579.292**	[M-FA15:0-H]$^-$	$C_{27}H_{48}O_{11}P^-$
**571.285**	[M-17:1ketene-H]$^-$	$C_{25}H_{48}O_{12}P^-$
**567.291**	[M-FA16:1-H]$^-$	$C_{26}H_{48}O_{11}P^-$
**565.280**	[M-FA16:0-H]$^-$	$C_{26}H_{46}O_{11}P^-$
**557.309**	[M-18:1ketene-H]$^-$	$C_{24}H_{46}O_{12}P^-$
**553.276**	[M-FA17:1-H]$^-$	$C_{25}H_{46}O_{11}P^-$
**551.261**	[M-FA17:0-H]$^-$	$C_{25}H_{44}O_{11}P^-$
**539.260**	[M-FA18:1-H]$^-$	$C_{24}H_{44}O_{11}P^-$
**537.243**	[M-FA18:0-H]$^-$	$C_{24}H_{42}O_{11}P^-$

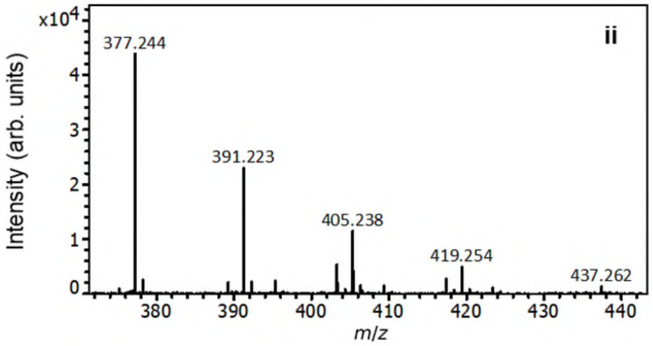

m/z measured	Fragment ion	Sum formula
**437.262**	[M-15:1ketene-inositol-H]$^-$	$C_{21}H_{42}O_7P^-$
**423.248**	[M-16:1ketene-inositol-H]$^-$	$C_{20}H_{40}O_7P^-$
**419.254**	[M-15:1FA-inositol-H]$^-$	$C_{21}H_{40}O_6P^-$
**417.237**	[M-15:0FA-inositol-H]$^-$	$C_{21}H_{38}O_6P^-$
**409.234**	[M-17:1ketene-inositol-H]$^-$	$C_{19}H_{38}O_7P^-$
**405.238**	[M-16:1FA-inositol-H]$^-$	$C_{20}H_{38}O_6P^-$
**403.225**	[M-16:0FA-inositol-H]$^-$	$C_{20}H_{36}O_6P^-$
**395.253**	[M-18:1ketene-inositol-H]$^-$	$C_{18}H_{36}O_7P^-$
**391.223**	[M-17:1FA-inositol-H]$^-$	$C_{19}H_{36}O_6P^-$
**389.208**	[M-17:0FA-inositol-H]$^-$	$C_{19}H_{34}O_6P^-$
**377.244**	[M-18:1FA-inositol-H]$^-$	$C_{18}H_{34}O_6P^-$
**375.230**	[M-18:0FA-inositol-H]$^-$	$C_{18}H_{32}O_6P^-$

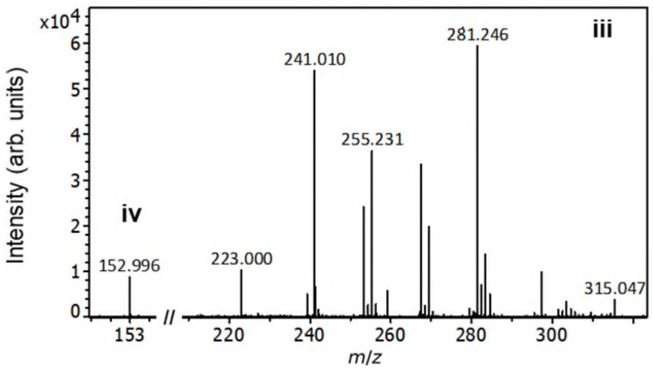

m/z measured	Fragment ion	Sum formula
**315.047**	[M-FA-ketene-H]$^-$	$C_9H_{16}O_{10}P^-$
**297.036**	[M-FA-FA-H]$^-$	$C_9H_{14}O_9P^-$
**283.261**	[FA18:0-H]$^-$	$C_{18}H_{35}O_2^-$
**281.246**	[FA18:1-H]$^-$	$C_{18}H_{33}O_2^-$
**269.246**	[FA17:0-H]$^-$	$C_{17}H_{33}O_2^-$
**267.230**	[FA17:1-H]$^-$	$C_{17}H_{31}O_2^-$
**259.090**	[inositolPO_3H_2-H]$^-$	$C_6H_{12}O_9P^-$
**255.231**	[FA16:0-H]$^-$	$C_{16}H_{31}O_2^-$
**253.215**	[FA16:1-H]$^-$	$C_{16}H_{29}O_2^-$
**241.216**	[FA15:0-H]$^-$	$C_{15}H_{29}O_2^-$
**241.010**	[inositolPO_3H_2-H_2O-H]$^-$	$C_6H_{10}O_8P^-$
**239.199**	[FA15:1-H]$^-$	$C_{15}H_{27}O_2^-$
**223.000**	[inositolPO_3H-2H_2O-H]$^-$	$C_6H_8O_7P^-$
**152.996**	[glycerolPO_3H_2-H]$^-$	$C_3H_6O_5P^-$

Supplementary Fig. 25. Unequivocal identification of odd-chain PI 33:1[M-H]- (m/z 821.517) by QCL-MIR imaging-guided MALDI-TIMS-MSI with iprm-PASEF. Isolated at $1/K_0$ = 1.416 Vs/cm² and fragmented with -53.5 eV. Inlet **i** represents a detailed view for *m/z* 530–610, **ii** for *m/z* 370–445, **iii** for*m/z* 210–310, and **iv** for *m/z* 151-154. The detailed descriptions of the elucidated fragment ions are displayed in tables together with chemical sum formulae. Steric effects made the neutral loss (NL) of the first FA more favorable at the *sn*-2 position[12]. Highest intensities were observed for the NL of FA18:1 and FA17:1. As a consequence, PI 15:0/18:1 and PI 16:0/17:1 were identified as the predominant isomeric structures[12]. Source data is provided as a Source Data file.

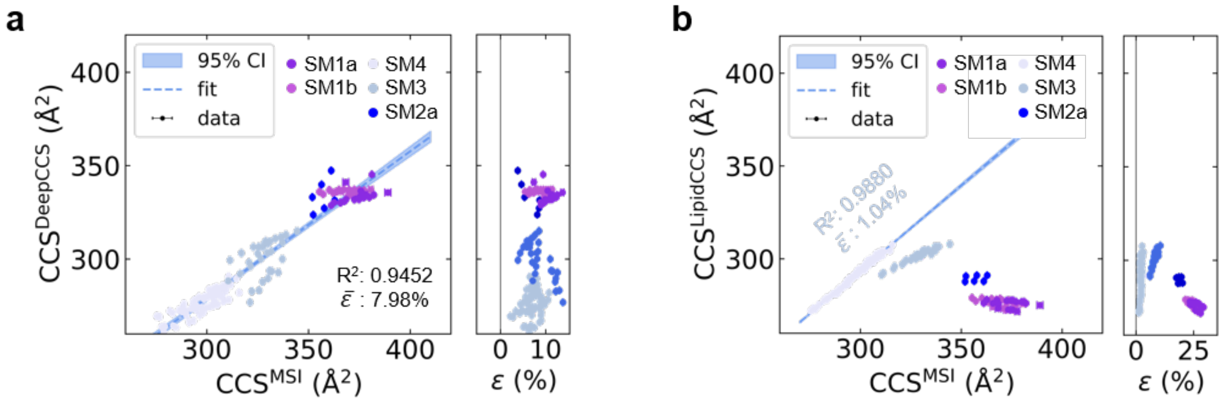

Supplementary Fig. 26. Correlation of experimentally deduced CCS values (QCL-MIR imaging microscopy-guided TIMS-MSI data) and CCS values predicted by IT tools: **a**, DeepCCS and **b**, LipidCCS. For all sulfatide CCS values (mean value for n=4 biological replicates) identified by TIMS-MSI (timsTOF fleX) in this study, AllCCS2 yielded the most accurate prediction, i.e., mean relative deviation $\bar{\varepsilon}$ of about 1.4% (**Fig. 4c**). For individual sulfatide classes it can be increased, e.g., $\bar{\varepsilon}$ = 2.48% for SM2a(1;O2). The accuracy of the predicted values for LipidCCS for the SM4 class is also on the level of 1%, but inaccurate for all other sulfatide classes. In contrast, $\bar{\varepsilon}$ is about 8.0% for CCS values predicted by DeepCCS. Relative deviation ε reveals uncorrelated dependencies for the relative deviation per subclass of the predicted CCS values using AllCCS2 against CCS values obtained by TIMS-MSI. Experimental CCS and *m/z* values are given as the mean across n=4 biological replicates and their uncertainties are expressed as standard deviation (see **Supplementary Table 13**). Source data is provided as a Source Data file.

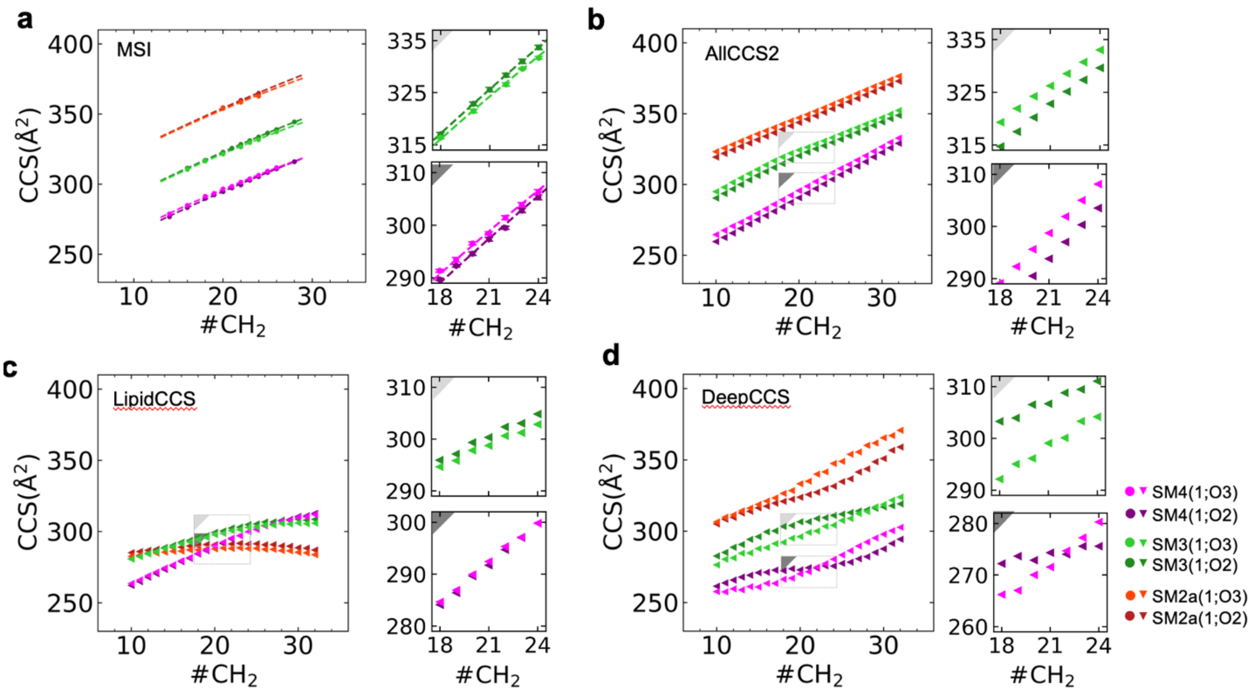

Supplementary Fig. 27. Predicted CCS values as a function of *N*-acyl-linked fatty acid chain length highlighting the structural trends for SM4(1;O2), SM4(1;O3), SM3(1;O2) and SM3(1;O3): **a**, MSI, b, AllCCS2, **c**, DeepCCS and **d**, LipidCCS. The different prediction tools, **b** AllCCS2, **c** DeepCCS, and **d** LipidCCS yield ambiguous relative structural relationships compared to experimental MSI data presented in **a**, **Fig. 3ad,** and **Suppl. Fig. 29** for LC-TIMS-MS data. Experimental CCS and *m/z* values are given as the mean across n=4 biological replicates and their uncertainties are expressed as standard deviation (see **Supplementary Table 13**). Source data is provided as a Source Data file.

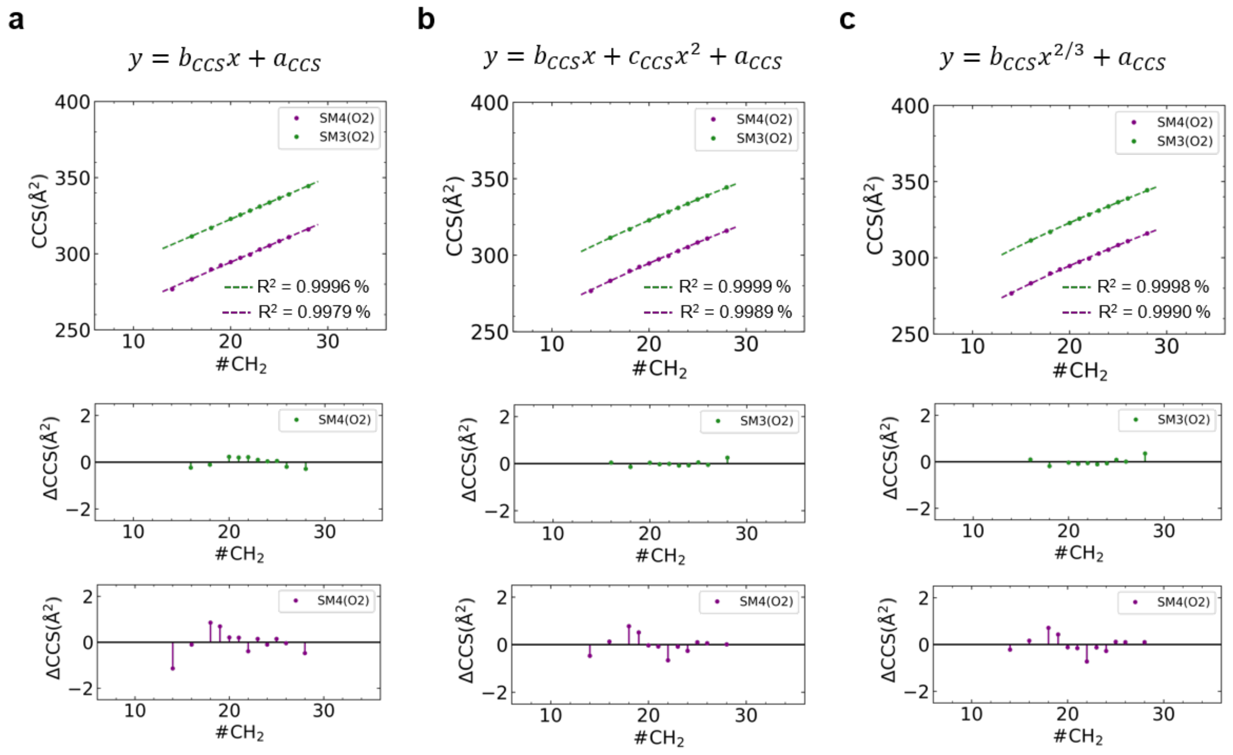

Supplementary Fig. 28. Modelling experimental CCS values as a function of the chain length of the *N*-acyl-linked fatty acid (FA). Linear data fit (**a**), 2nd order polynomial fit (**b**), and $y = b_{CCS}x^{2/3} + a_{CCS}$ (**c**), a fit commonly used to describe CCS values of polymers[13]. A global least square fitting procedure was used with fixed parameters, c_{CCS} and/or b_{CCS} each. Residuals are shown, indicating best agreement between the data and the 2nd order polynomial fit (**b**). Source data is provided as a Source Data file.

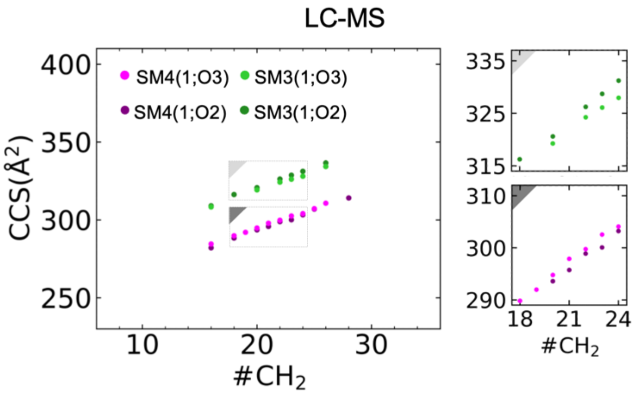

Supplementary Fig. 29. Experimental (TIMS-MSI and LC-MS) and predicted CCS values as a function of *N*-acyl-linked fatty acid chain length. Experimental LC-TIMS-MS-derived CCS values (n=4 biological replicates) for the subclasses SM4 18+n:1;O3 (magenta), SM4 18+n:1;O2 (purple), SM3 18+n:1;O3 (light green), SM3 18+n:1;O2 (dark green). n denotes the chain length alteration. Source data is provided as a Source Data file.

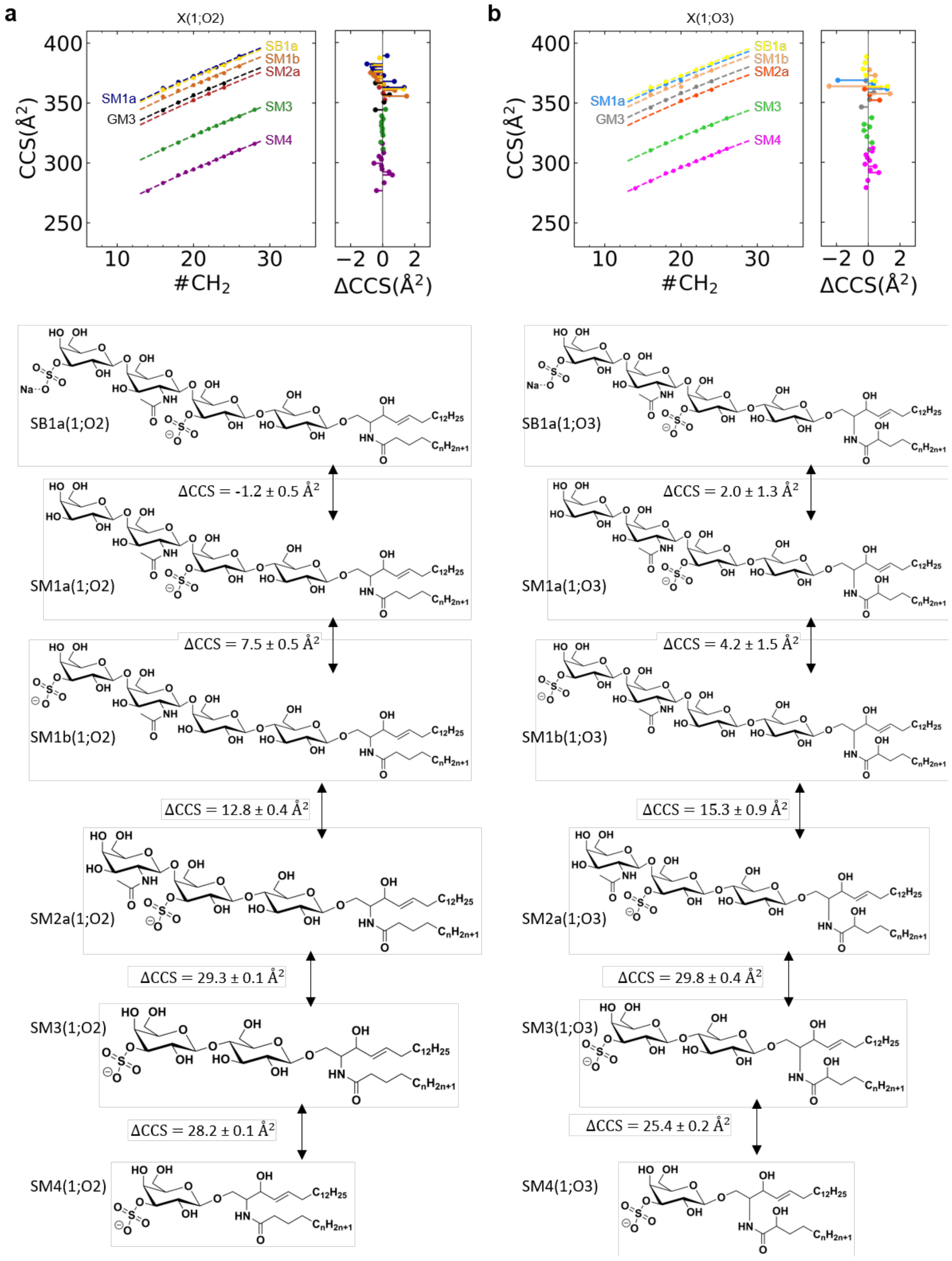

Supplementary Fig. 30. Experimental CCS values for X(1;O2) (a) and X(1;O3) (b) sulfatide species. 2nd order polynomial fit with constrained parameters, c_{CCS} and b_{CCS} (**Supplementary Fig. 28**), was used to evaluate the relative contribution of the degree of glycosylation in the sulfated head group and the α-hydroxylation of the N-acyl FA. Residuals are also presented. Chemical structures for the corresponding sulfatide subclasses and inter-subclass-differences in CCS values (mean value for n=4 biological replicates) are shown. Source data is provided as a Source Data file.

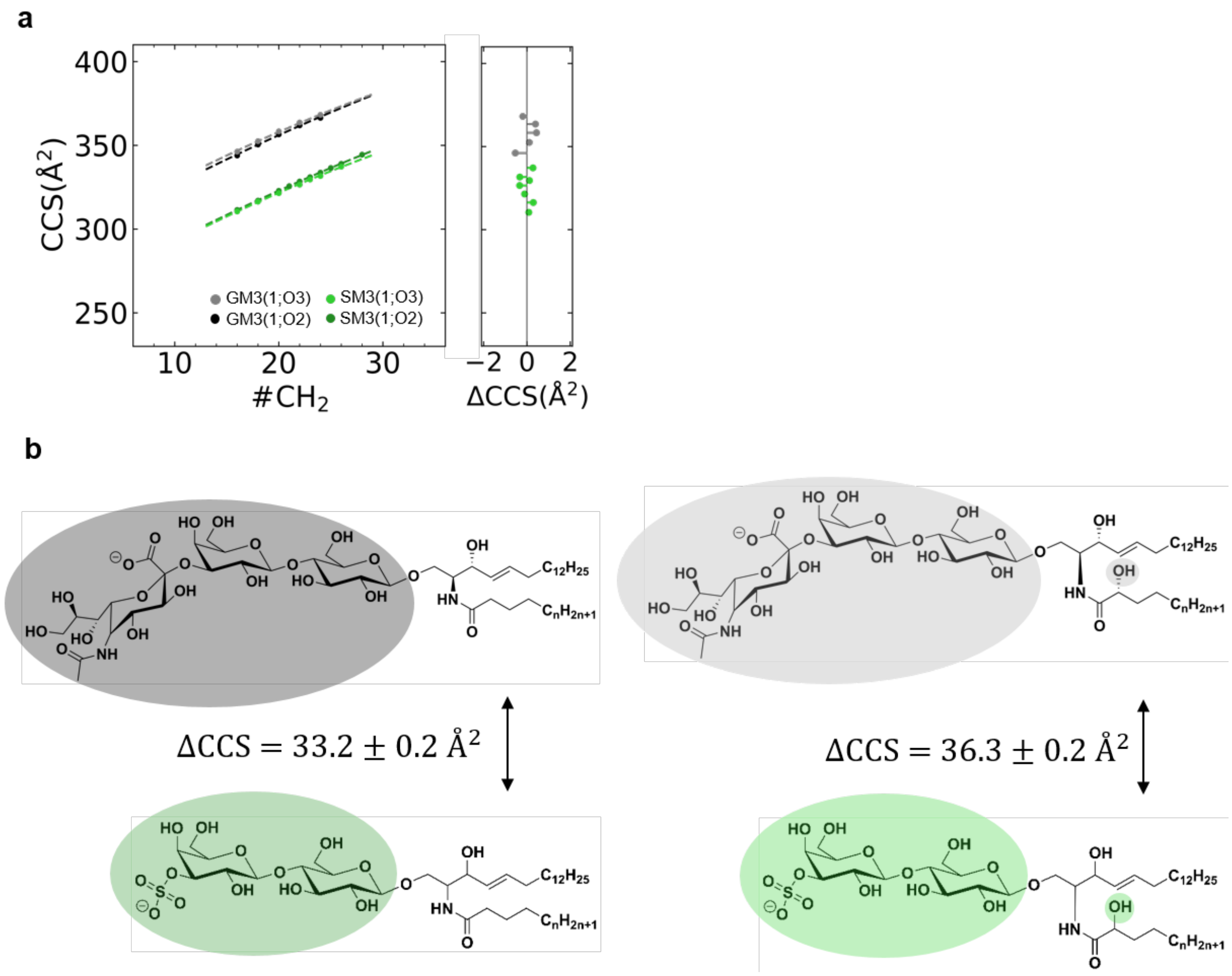

Supplementary Fig. 31. Structure-CCS-relationship for SM3 and GM3 subclasses. **a**, Experimental CCS values (mean value for n=4 biological replicates) for SM3(1;O2), SM3(1;O3), GM3(1;O2) and GM3(1;O3) species. **b**, Chemical structures and results for the differences of the relative CCS curves associated with the contribution of the sulfate group (green) or the sialic acid group (gray) for SM3(O2) vs. GM3(O2) (left), and SM3(O3) vs. GM3(O3) (right) are presented. The difference is increased for SM3(O3) vs. GM3(O3), suggesting a possible influence of the α-OH group. Source data is provided as a Source Data file.

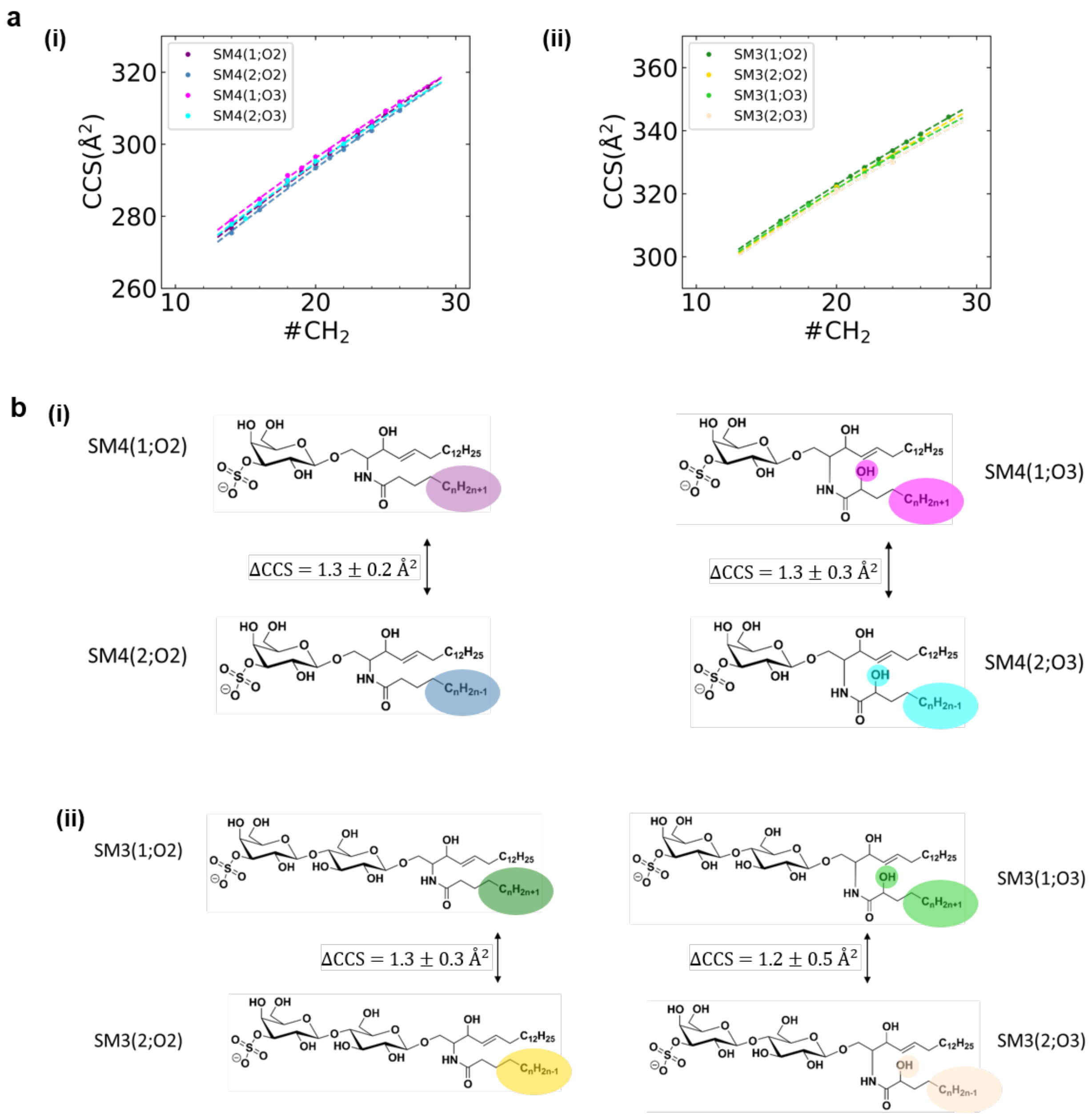

Supplementary Fig. 32. Evolution of the relative CCS values between selected SM3 and SM4 subclasses incorporating either a saturated FA (C_nH_{2n+1}), or a mono-unsaturated FA (C_nH_{2n-1}). **a,** Comparison of CCS values (mean value for n=4 biological replicates) for sulfatide series SM4(O2/O3) **(i)**, and SM3(O2/O3) **(ii)**, depending on the degree of unsaturation in their *N*-acyl-linked FAs. Data is modelled by a 2nd order polynomial fit with fixed parameters of the linear and quadratic term of the polynomial fit. **b**, The differences in CCS values associated to the contribution of the degree of unsaturation in the *N*-acyl-linked FA for SM4(O2/O3) **(i)**, and SM3(O2/O3) **(ii)** are shown. The mean relative difference was 1.3±0.2 Å^2. Source data is provided as a Source Data file.

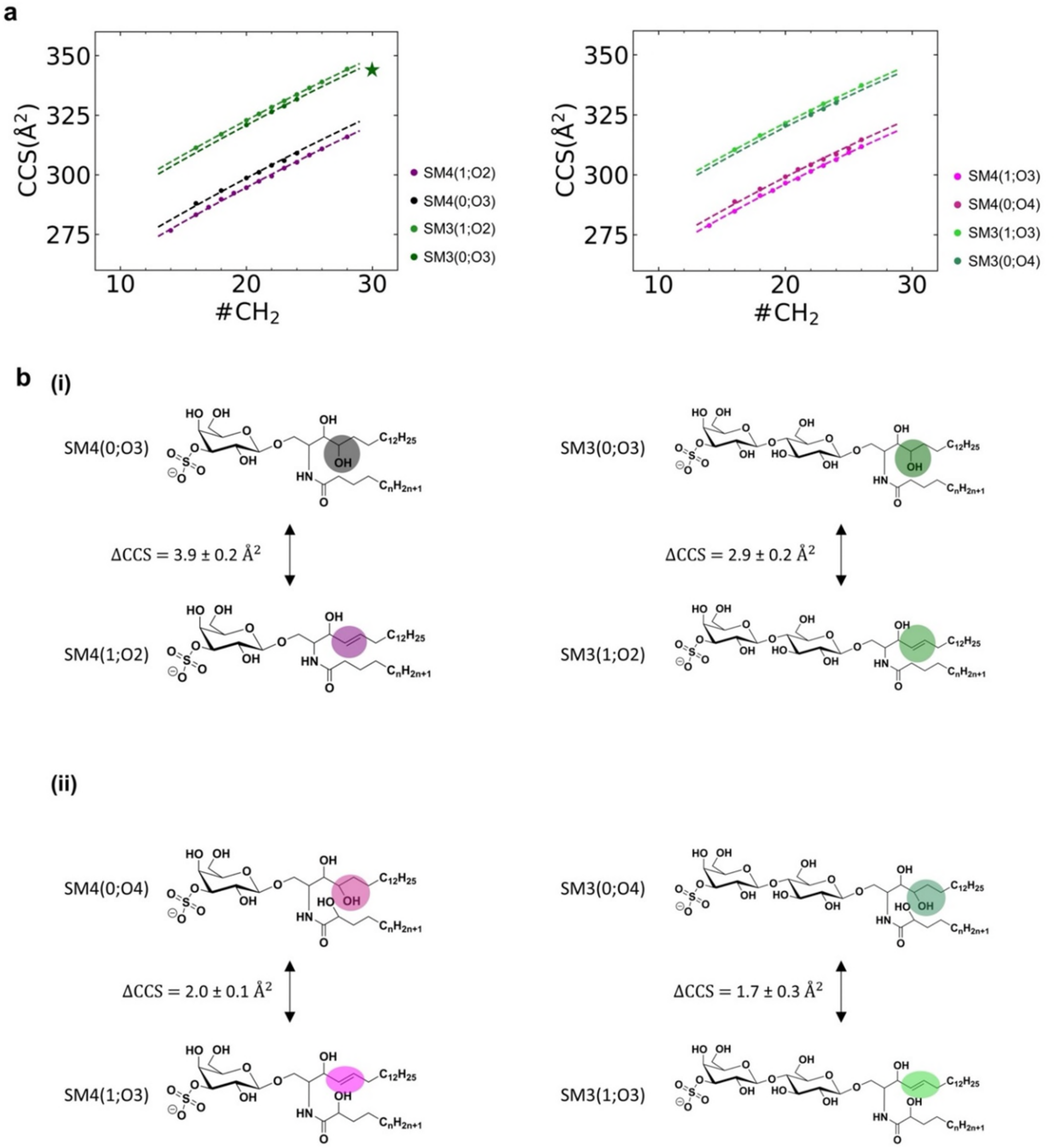

Supplementary Fig. 33. Evolution of the relative CCS values between selected SM3 and SM4 subclasses. **a,** Comparison of CCS values (mean value for n=4 biological replicates) for sulfatide series SM4/SM3(1;O2) against SM4/ SM3(0;O3) (left), and for sulfatide series SM4/SM3(1;O3) against SM4/SM3(0;O4) (right). For SM3(0;O3), the LC-MS based CCS data (green star) was taken, and a corrected offset according to results in **Fig. 3b** was considered. Data is modelled by a 2nd order polynomial fit with fixed amplitudes of the linear and quadratic term of the polynomial fit. **b,** Chemical structures and results for the differences of the relative CCS curves to demonstrate the influence of a phytosphingoid backbone. Source data is provided as a Source Data file.

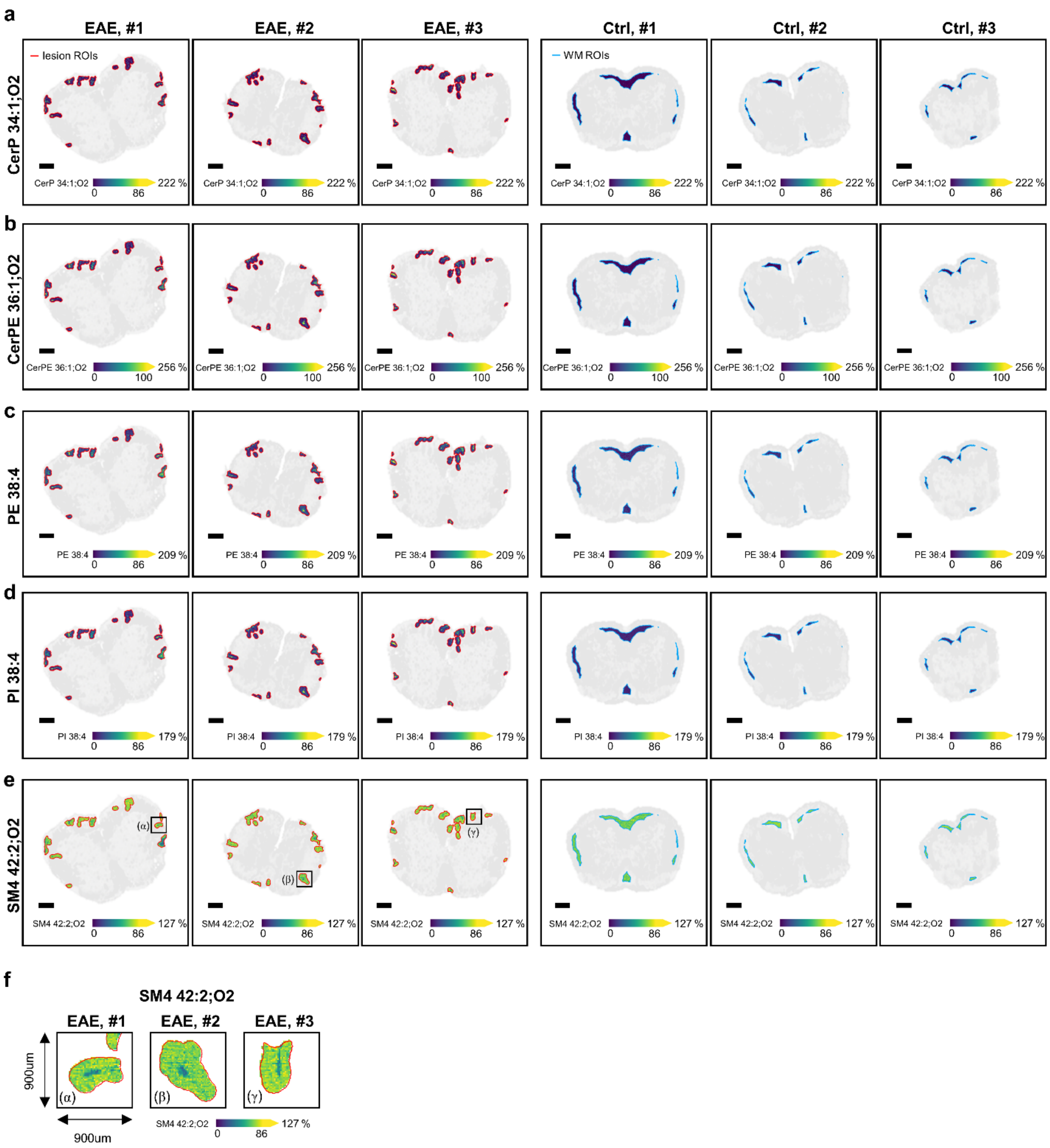

Supplementary Fig. 34. Comparison of lesions in EAE mouse model against healthy control tissue for n=3 biological replicates each. **a,** Ion images for *m/z* 616.472 (CerP 34:1;O2[M-H]$^-$). **b,** ion images for *m/z* 687.543 (CerPE 36:1;O2[M-H]$^-$). **c,** ion images for *m/z* 766.548 (PE 38:4[M-H]$^-$). **d,** ion images for *m/z* 885.549 (PI 38:4[M-H]$^-$). **e,** ion images for *m/z* 888.624 (SM4 42:2;O2[M-H]$^-$). **f,** enlarged view on three different lesions for *m/z* 888.624 (SM4 42:2;O2[M-H]$^-$) to highlight the early onset of demyelination characterized by the reduced signal intensity in the lesion area. Scale bar, 300 µm in **a-e**.

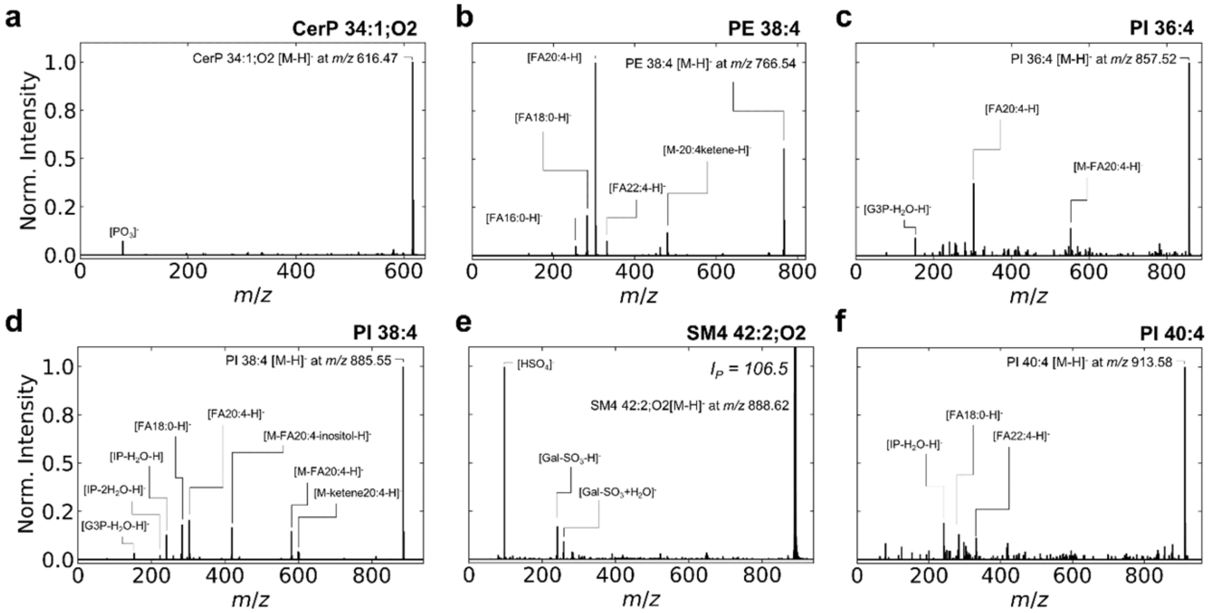

Supplementary Fig. 35. QCL-based MIR imaging microscopy-guided imaging parallel reaction monitoring with parallel accumulation serial fragmentation (iprm-PASEF)-MS^2 spectra of *m/z* features in EAE lesion areas. All MS^2 spectra were recorded on a timsTOF fleX. **a,** *m/z* 616.47 (CerP 34:1;O2$[M-H]^-$) isolated at $1/K_0$ = 1.246 Vs/cm², fragmented with -40.0 eV. **b,** *m/z* 766.54 (PE 38:4$[M-H]^-$) at $1/K_0$ = 1.350 Vs/cm², fragmented -41.5 eV. **c,** *m/z* 857.52 (PI 36:4$[M-H]^-$) at $1/K_0$ = 1.427 Vs/cm², fragmented -43.4 eV. **d,** *m/z* 885.55 (PI 38:4$[M-H]^-$) at $1/K_0$ = 1.457 Vs/cm², fragmented -55.5 eV. **e,** *m/z* 888.62 (SM4 42:2;O2$[M-H]^-$) at $1/K_0$ = 1.499 Vs/cm², fragmented -60.0 eV. **f,** *m/z* 913.58 (PI 40:4$[M-H]^-$) at $1/K_0$ = 1.485 Vs/cm², fragmented -56.4 eV. Detailed fragment ion identifications are marked in the spectra and can be found in **Supplementary Tables 15-21**. Intensity of the precursor is denoted as I_P. Source data is provided as a Source Data file.

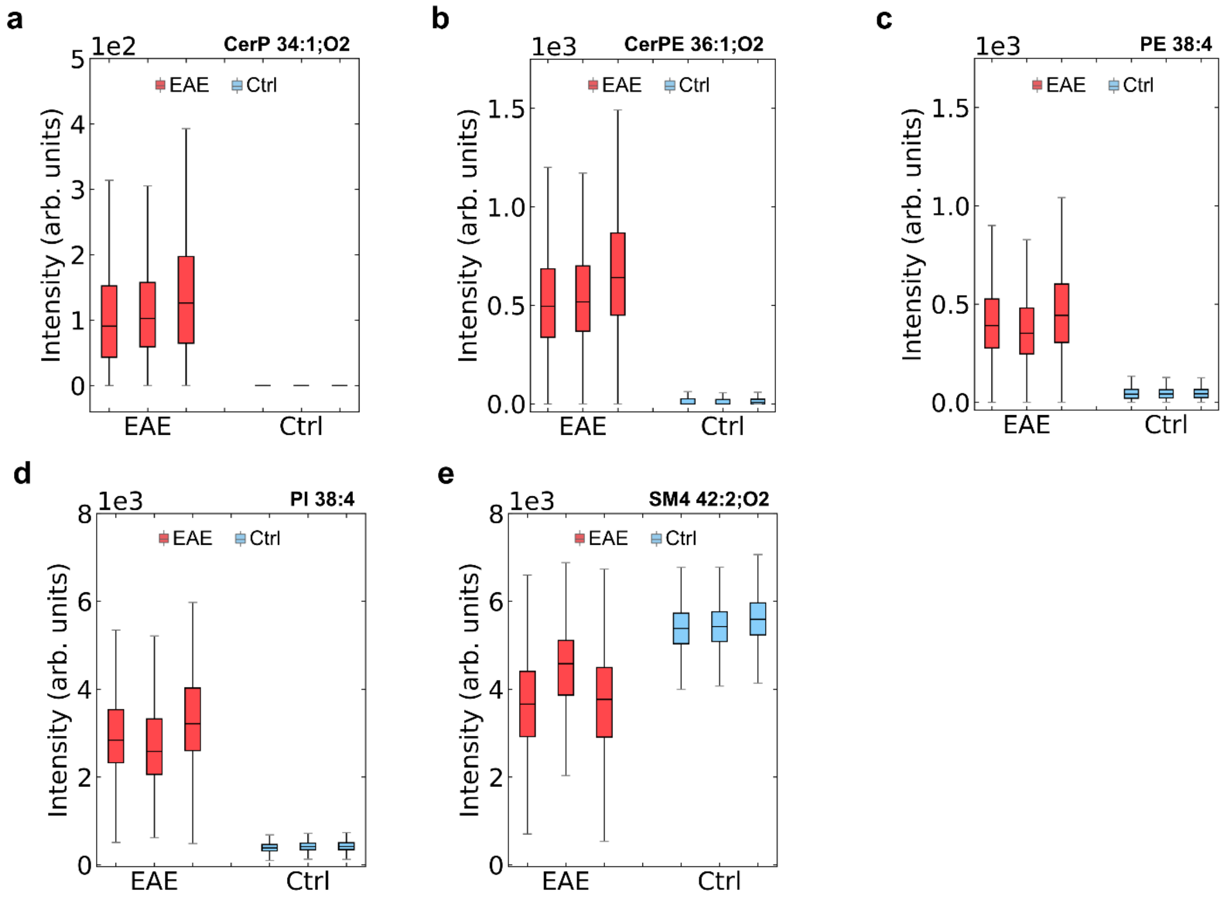

Supplementary Fig. 36. Statistical analysis highlighting the role of proinflammatory lipids in dynamic lipid remodeling in EAE mouse spinal cord lesions compared against white matter area of healthy control mice. EAE lesion areas were obtained by k=2 clustering of the lesion ROIs of the QCL-MIR imaging-guided MSI data and compared against white matter areas of healthy control tissue. Intensity boxplots for n=3 biological replicates including n=3 technical replicates measured on consecutive sections. Two-sided t-statistics yield Benjamini-Hochberg corrected p-values of 0.0016 (**a**, CerP 34:1;O2), 0.0013 (**b**, CerPE 36:1;O2), 0.0015 (**c**, PE 38:4), 0.0011 (**d**, PI 38:4), 0.0136 (**e**, SM4 42:2;O2). Boxplots indicate median (middle line), 25th and 75th percentile (box) and whiskers (1.5 times the interquartile range). Source data is provided as a Source Data file.

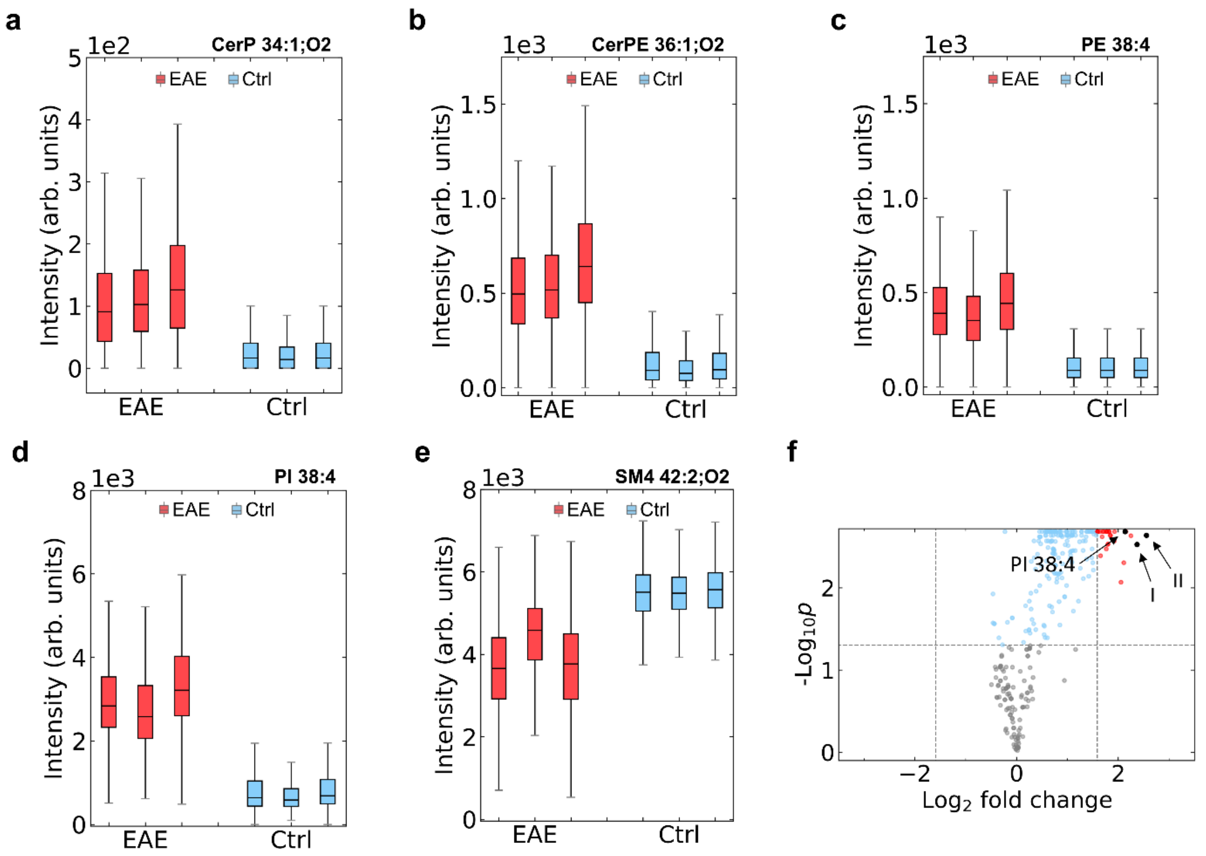

Supplementary Fig. 37. Statistical analysis highlighting the role of proinflammatory lipids in dynamic lipid remodeling in EAE mouse spinal cord lesions compared against adjacent normal white matter area. EAE lesion areas and control areas were obtained by k=2 clustering of the lesion ROIs of the QCL-MIR imaging-guided MSI data. **a-e**, Intensity boxplots for n=3 biological replicates including n=3 technical replicates measured on consecutive sections. T-statistics yield Benjamini-Hochberg corrected p-values are 0.0030 (CerP 34:1;O2), 0.0023 (CerPE 36:1;O2), 0.0023 (PE 38:4), 0.0021 (PI 38:4), 0.0118 (SM4 42:2;O2). Boxplots indicate median (middle line), 25th and 75th percentile (box) and whiskers (1.5 times the interquartile range). **f**, Volcano scatter plot reveals significantly enriched *m/z* features (red dots; including **I** CerP 34:1;O2**, II** CerPE 36:1;O2**,** and PI 38:4 as black dots, similar to **Fig. 4c**) in EAE lesions. Statistical significance was tested by two-sided standard t-test for n=3 biological replicates. P-values are Benjamini–Hochberg-corrected. Source data is provided as a Source Data file.

Supplementary Tables

Supplementary Table 1. Overview of significant features (as determined by lasso method) in MCF and BCF. Experimental CCS and *m/z* values are presented as mean for n=18 technical replicates and their uncertainties are given as standard deviation in parentheses. All ions detected as $[M-H]^-$.

Name	Chemical sum formula	*m/z* theoretical	1/K0 (V·s/cm²)	CCS (Å²)	*m/z* timsTOF	ppm timsTOF
FA 20:5	C20H30O2	301.217304	0.863(6)	181(1)	301.219(4)	7.00
lyso-PI 18:0	C27H53O12P	599.320187	1.176(7)	241(1)	599.320(1)	-0.66
PE 32:1	C37H72NO8P	688.492278	1.289(7)	263(1)	688.494(1)	2.73
PE (P-36:4)	C41H74NO7P	722.513014	1.330(7)	271(1)	722.512(1)	-0.94
PA 38:2	C41H77O8P	727.528330	1.336(7)	273(1)	727.529(1)	0.58
PI 33:1	C42H79O13P	821.518553	1.427(7)	291(1)	821.516(2)	2.74
PI 34:1	C43H81O13P	835.534203	1.430(7)	291(1)	835.534(1)	-0.74
PI 36:3	C45H81O13P	859.534203	1.444(7)	294(1)	859.533(1)	-1.16
PI 40:4	C49H87O13P	913.581153	1.497(7)	304(1)	913.581(1)	-0.50

Supplementary Table 2. Identified fragment ions of lyso-PI 18:0$[M-H]^-$ (*m/z* 599.317). Isolated at $1/K_0$ = 1.171 Vs/cm² and fragmented with -40.0 eV. Fragment patterns were modelled according to Hsu and Turk, 2009[12].

m/z measured	Fragment ion	Sum formula
599.317	$[M-H]^-$	$C_{27}H_{52}O_{12}P^-$
419.254	$[M\text{-inositol-}H]^-$	$C_{21}H_{40}O_6P^-$
315.047	$[M\text{-FA18:0-}H]^-$	$C_9H_{16}O_{10}P^-$
283.262	$[FA18:0\text{-}H]^-$	$C_{18}H_{35}O_2^-$
241.010	$[inositolPO_3H_2\text{-}H]^-$	$C_6H_{10}O_8P^-$
152.994	$[glycerolPO_3H_2\text{-}H]^-$	$C_3H_6O_5P^-$

Supplementary Table 3. Identified fragment ions of PE 32:1[M-H]$^-$ (*m/z* 688.489). Isolated at 1/K_0 = 1.281 Vs/cm² and fragmented with -40.0 eV. PE 16:0/16:1 was identified as the predominant isomeric structure[12].

m/z measured	Fragment ion	Sum formula
688.489	[M-H]$^-$	$C_{37}H_{71}NO_8P^-$
478.292	[M-18:1ketene-H]$^-$	$C_{23}H_{45}NO_7P^-$
460.266	[M-FA18:1-H]$^-$	$C_{23}H_{43}NO_6P^-$
452.276	[M-16:1ketene-H]$^-$	$C_{21}H_{43}NO_7P^-$
450.261	[M-16:0ketene-H]$^-$	$C_{21}H_{41}NO_7P^-$
434.266	[M-FA16:1-H]$^-$	$C_{21}H_{41}NO_6P^-$
432.250	[M-FA16:0-H]$^-$	$C_{21}H_{39}NO_6P^-$
424.245	[M-14:0ketene-H]$^-$	$C_{19}H_{39}NO_7P^-$
281.246	[FA18:1-H]$^-$	$C_{18}H_{33}O_2^-$
255.231	[FA16:0-H]$^-$	$C_{16}H_{31}O_2^-$
253.215	[FA16:1-H]$^-$	$C_{16}H_{29}O_2^-$
227.199	[FA14:0-H]$^-$	$C_{14}H_{27}O_2^-$

Supplementary Table 4. Identified fragment ions of PE P-36:4[M-H]$^-$ (*m/z* 722.511). Isolated at 1/K_0 = 1.317 Vs/cm² and fragmented with -43.4 eV. PE P-18:0/18:4 was identified as the predominant isomeric structure[12].

m/z measured	Fragment ion	Sum formula
722.511	[M-H]$^-$	$C_{41}H_{73}NO_7P^-$
437.264	[M-20:4ketene-H]$^-$	$C_{21}H_{42}NO_5P^-$
419.254	[M-FA20:4-H]$^-$	$C_{21}H_{39}NO_4P^-$
303.230	[FA20:4-H]$^-$	$C_{20}H_{31}O_2^-$
301.214	[FA20:5-H]$^-$	$C_{20}H_{29}O_2^-$
283.263	[FA18:0-H]$^-$	$C_{18}H_{35}O_2^-$
281.247	[FA18:1-H]$^-$	$C_{18}H_{33}O_2^-$
255.232	[FA16:0-H]$^-$	$C_{16}H_{31}O_2^-$
152.994	[$MePO_3EtNH_2$-H]-	$C_3H_8NO_4P^-$

Supplementary Table 5. Identified fragment ions of PA 38:2[M-H]$^-$ (*m/z* 727.523). Isolated at $1/K_0$ = 1.330 Vs/cm² and fragmented with -44.5 eV. PA 20:1/18:1 was identified as the predominant isomeric structure[12].

***m/z* measured**	**Fragment ion**	**Sum formula**
727.523	[M-H]$^-$	$C_{41}H_{76}O_8P^-$
463.281	[M-18:1ketene-H]$^-$	$C_{23}H_{44}NO_7P^-$
445.269	[M-FA18:1-H]$^-$	$C_{23}H_{42}NO_6P^-$
419.254	[M-FA20:2-H]$^-$	$C_{21}H_{40}NO_6P^-$
337.307	[FA22:1-H]$^-$	$C_{22}H_{41}O_2^-$
309.278	[FA20:1-H]$^-$	$C_{20}H_{37}O_2^-$
307.258	[FA20:2-H]$^-$	$C_{20}H_{35}O_2^-$
281.246	[FA18:1-H]$^-$	$C_{18}H_{33}O_2^-$
253.216	[FA16:1-H]$^-$	$C_{16}H_{29}O_2^-$
152.994	[glycerolPO$_3$H$_2$-H]$^-$	$C_3H_6O_5P^-$

Supplementary Table 6. Identified fragment ions of PI 33:1[M-H]$^-$ (*m/z* 821.517). Isolated at $1/K_0$ = 1.416 Vs/cm² and fragmented with -53.5 eV. PI 15:0/18:1 and PI 16:0/17:1 were identified as the predominant isomeric structures[12].

m/z measured	Fragment ion	Sum formula
821.517	[M-H]$^-$	$C_{42}H_{78}O_{13}P^-$
599.320	[M-15:1ketene-H]$^-$	$C_{27}H_{52}O_{12}P^-$
597.303	[M-15:0ketene-H]$^-$	$C_{27}H_{50}O_{12}P^-$
585.306	[M-16:1ketene-H]$^-$	$C_{26}H_{50}O_{12}P^-$
583.286	[M-16:0ketene-H]$^-$	$C_{26}H_{48}O_{12}P^-$
581.306	[M-FA15:1-H]$^-$	$C_{27}H_{50}O_{11}P^-$
579.292	[M-FA15:0-H]$^-$	$C_{27}H_{48}O_{11}P^-$
571.285	[M-17:1ketene-H]$^-$	$C_{25}H_{48}O_{12}P^-$
567.290	[M-FA16:1-H]$^-$	$C_{26}H_{48}O_{11}P^-$
565.280	[M-FA16:0-H]$^-$	$C_{26}H_{46}O_{11}P^-$
557.309	[M-18:1ketene-H]$^-$	$C_{24}H_{46}O_{12}P^-$
553.277	[M-FA17:1-H]$^-$	$C_{25}H_{46}O_{11}P^-$
551.260	[M-FA17:0-H]$^-$	$C_{25}H_{44}O_{11}P^-$
539.261	[M-FA18:1-H]$^-$	$C_{24}H_{44}O_{11}P^-$
537.243	[M-FA18:0-H]$^-$	$C_{24}H_{42}O_{11}P^-$
437.262	[M-15:1ketene-inositol-H]$^-$	$C_{21}H_{42}O_7P^-$
423.248	[M-16:1ketene-inositol-H]$^-$	$C_{20}H_{40}O_7P^-$
419.254	[M-FA15:1-inositol-H]$^-$	$C_{21}H_{40}O_6P^-$
417.237	[M-FA15:0-inositol-H]$^-$	$C_{21}H_{38}O_6P^-$
409.234	[M-17:1ketene-inositol-H]$^-$	$C_{19}H_{38}O_7P^-$
405.238	[M-FA16:1-inositol-H]$^-$	$C_{20}H_{38}O_6P^-$
403.225	[M-FA16:0-inositol-H]$^-$	$C_{20}H_{36}O_6P^-$
395.253	[M-18:1ketene-inositol-H]$^-$	$C_{18}H_{36}O_7P^-$
391.223	[M-FA17:1-inositol-H]$^-$	$C_{19}H_{36}O_6P^-$
389.208	[M-FA17:0-inositol-H]$^-$	$C_{19}H_{34}O_6P^-$
377.244	[M-FA18:1-inositol-H]$^-$	$C_{18}H_{34}O_6P^-$
375.230	[M-FA18:0-inositol-H]$^-$	$C_{18}H_{32}O_6P^-$
315.048	[M-FA-ketene-H]$^-$	$C_9H_{16}O_{10}P^-$
297.036	[M-FA-FA-H]$^-$	$C_9H_{14}O_9P^-$
283.261	[FA18:0-H]$^-$	$C_{18}H_{35}O_2^-$
281.246	[FA18:1-H]$^-$	$C_{18}H_{33}O_2^-$
269.246	[FA17:0-H]$^-$	$C_{17}H_{33}O_2^-$
267.230	[FA17:1-H]$^-$	$C_{17}H_{31}O_2^-$
259.090	[inositolPO$_3$H$_2$-H]$^-$	$C_6H_{12}O_9P^-$
255.231	[FA16:0-H]$^-$	$C_{16}H_{31}O_2^-$
253.215	[FA16:1-H]$^-$	$C_{16}H_{29}O_2^-$
241.216	[FA15:0-H]$^-$	$C_{15}H_{29}O_2^-$
241.010	[inositolPO$_3$H$_2$-H$_2$O-H]$^-$	$C_6H_{10}O_8P^-$
239.199	[FA15:1-H]$^-$	$C_{15}H_{27}O_2^-$
223.000	[inositolPO$_3$H-2H$_2$O-H]$^-$	$C_6H_8O_7P^-$
152.996	[glycerolPO$_3$H$_2$-H]$^-$	$C_3H_6O_5P^-$

Supplementary Table 7. Identified fragment ions of PI 34:1[M-H]$^-$ (3rd carbon isotope ($^{13}C_3$), *m/z* 838.541). Isolated at 1/K_0 = 1.427 Vs/cm² and fragmented with -54.5 eV.

m/z measured	Fragment ion	Sum formula
838.541	$[M(^{13}C_3)\text{-}H]^-$	$C_{42}H_{78}O_{13}P^-$
598.317–602.331	$[M(^{13}C_{3\text{-}n})$-16:1(0)ketene-H$]^-$	$^{13}C_{3\text{-}n}C_{24+n}H_{52(50)}O_{12}P^-$
579.291–584.315	$[M(^{13}C_{3\text{-}n})$-FA16:1(0) -H$]^-$	$^{13}C_{3\text{-}n}C_{24+n}H_{50(48)}O_{11}P^-$
571.292–575.300	$[M(^{13}C_{3\text{-}n})$-18:1(0)ketene-H$]^-$	$^{13}C_{3\text{-}n}C_{22+n}H_{48(46)}O_{12}P^-$
552.263–557.321	$[M(^{13}C_{3\text{-}n})$-FA18:1(0)-H$]^-$	$^{13}C_{3\text{-}n}C_{22+n}H_{46(44)}O_{11}P^-$
417.234–422.260	$[M(^{13}C_{3\text{-}n})$-FA16:1(0)-inositol-H$]^-$	$^{13}C_{3\text{-}n}C_{18+n}H_{40(38)}O_6P^-$
389.207–393.232	$[M(^{13}C_{3\text{-}n})$-FA18:1(0)-inositol-H$]^-$	$^{13}C_{3\text{-}n}C_{16+n}H_{36(34)}O_6P^-$
315.045–318.056	[M-FA-ketene-H]$^-$	$^{13}C_{3\text{-}n}C_6H_{16}O_{10}P^-$
297.031–300.039	[M-FA-FA-H]$^-$	$^{13}C_{3\text{-}n}C_6H_{14}O_9P^-$
281.247–286.280	$[(^{13}C_{3\text{-}n})$18:1(0)FA-H$]^-$	$^{13}C_{3\text{-}n}C_{15}H_{34(36)}O_2^-$
259.022–262.036	$[\text{inositolPO}_3H_2\text{-}H]^-$	$^{13}C_{3\text{-}n}C_3H_{12}O_9P^-$
253.213–258.242	$[(^{13}C_{3\text{-}n})$16:1(0)FA-H$]^-$	$^{13}C_{3\text{-}n}C_{13}H_{30(32)}O_2^-$
241.009–244.016	$[\text{inositolPO}_3H_2\text{-}H_2O\text{-}H]^-$	$^{13}C_{3\text{-}n}C_3H_{10}O_8P^-$
223.000–226.006	$[\text{inositolPO}_3H\text{-}2H_2O\text{-}H]^-$	$^{13}C_{3\text{-}n}C_3H_8O_7P^-$
152.994–155.000	$[\text{glycerolPO}_3H_2\text{-}H]^-$	$^{13}C_{3\text{-}n}C_3H_6O_5P^-$

Supplementary Table 8. Identified fragment ions of PI 36:3[M-H]- (*m/z* 859.530). Isolated at 1/K_0 = 1.438 Vs/cm² and fragmented with -55.3 eV. PI 16:0/20:3 was identified as the predominant isomeric structure[12].

m/z measured	Fragment ion	Sum formula
859.530	[M-H]$^-$	$C_{42}H_{78}O_{13}P^-$
621.303	[M-16:0ketene-H]$^-$	$C_{29}H_{50}O_{12}P^-$
603.290	[M-FA16:0-H]$^-$	$C_{29}H_{48}O_{11}P^-$
599.319	[M-18:3ketene-H]$^-$	$C_{27}H_{52}O_{12}P^-$
597.302	[M-18:2ketene-H]$^-$	$C_{27}H_{50}O_{12}P^-$
595.299	[M-18:1ketene-H]$^-$	$C_{27}H_{48}O_{12}P^-$
593.265	[M-18:0ketene-H]$^-$	$C_{27}H_{46}O_{12}P^-$
581.306	[M-FA18:3-H]$^-$	$C_{27}H_{50}O_{11}P^-$
579.291	[M-FA18:2-H]$^-$	$C_{27}H_{48}O_{11}P^-$
577.277	[M-FA18:1-H]$^-$	$C_{27}H_{46}O_{11}P^-$
575.258	[M-FA18:0-H]$^-$	$C_{27}H_{44}O_{11}P^-$
571.293	[M-20:3ketene-H]$^-$	$C_{25}H_{48}O_{12}P^-$
553.276	[M-FA20:3-H]$^-$	$C_{26}H_{46}O_{11}P^-$
441.236	[M-FA16:0-inositol-H]$^-$	$C_{23}H_{38}O_6P^-$
419.256	[M-FA18:3-inositol-H]$^-$	$C_{21}H_{40}O_6P^-$
417.240	[M-FA18:2-inositol-H]$^-$	$C_{21}H_{38}O_6P^-$
415.224	[M-FA18:1-inositol-H]$^-$	$C_{21}H_{36}O_6P^-$
413.209	[M-FA18:0-inositol-H]$^-$	$C_{23}H_{34}O_6P^-$
391.230	[M-FA20:3-inositol-H]$^-$	$C_{19}H_{36}O_6P^-$
315.050	[M-FA-ketene-H]$^-$	$C_9H_{16}O_{10}P^-$

305.246	[FA20:3-H]$^-$	$C_{20}H_{33}O_2^-$
297.037	[M-FA-FA-H]$^-$	$C_9H_{14}O_9P^-$
283.262	[FA18:0-H]$^-$	$C_{18}H_{35}O_2^-$
281.247	[FA18:1-H]$^-$	$C_{18}H_{33}O_2^-$
279.231	[FA18:2-H]$^-$	$C_{18}H_{31}O_2^-$
277.214	[FA18:3-H]$^-$	$C_{18}H_{29}O_2^-$
259.022	[inositolPO$_3$H$_2$-H]$^-$	$C_6H_{12}O_9P^-$
255.231	[FA16:0-H]$^-$	$C_{16}H_{31}O_2^-$
241.010	[inositolPO$_3$H$_2$-H$_2$O-H]$^-$	$C_6H_{10}O_8P^-$
223.000	[inositolPO$_3$H-2H$_2$O-H]$^-$	$C_6H_8O_7P^-$
152.994	[glycerolPO$_3$H$_2$-H]$^-$	$C_3H_6O_5P^-$

Supplementary Table 9. Identified fragment ions of PI 40:4[M-H]$^-$ (2nd carbon isotope ($^{13}C_2$), *m/z* 915.593). Isolated at 1/K_0 = 1.494 Vs/cm² and fragmented with -59.1 eV. PI 18:1/22:3 and PI 18:0/22:4 were identified as the predominant isomeric structures[12].

m/z **measured**	**Fragment ion**	**Sum formula**
915.593	[M($^{13}C_2$)-H]$^-$	$C_{42}H_{78}O_{13}P^-$
628.282–631.315	[M($^{13}C_{2-n}$)-FA18:1(0)-H]$^-$<br>[M($^{13}C_{2-n}$)-20:4ketene-H]$^-$	$^{13}C_{2-n}C_{29+n}H_{50(48)}O_{11}P^-$<br>$^{13}C_{2-n}C_{27+n}H_{56}O_{12}P^-$
609.343–611.347	[M($^{13}C_{2-n}$)-FA20:4-H]$^-$	$^{13}C_{2-n}C_{27+n}H_{54}O_{11}P^-$
597.300–603.348	[M($^{13}C_{2-n}$)-22:4(3)ketene-H]$^-$<br>[M($^{13}C_{2-n}$)-20:4FA-H]$^-$	$^{13}C_{2-n}C_{25+n}H_{52(50)}O_{12}P^-$<br>$^{13}C_{2-n}C_{27+n}H_{46}O_{11}P^-$
579.290–585.320	[M($^{13}C_{2-n}$)-22:4(3)FA-H]$^-$	$^{13}C_{2-n}C_{25+n}H_{50(48)}O_{11}P^-$
467.247–471.268	[M($^{13}C_{2-n}$)-FA18:1(0)-inositol-H]$^-$	$^{13}C_{2-n}C_{23+n}H_{42(40)}O_6P^-$
417.238–422.257	[M($^{13}C_{2-n}$)-F22:4(3)-inositol-H]$^-$	$^{13}C_{2-n}C_{19+n}H_{40(38)}O_6P^-$
333.272	[FA22:3-H]$^-$	$C_{22}H_{37}O_2^-$
331.258	[FA22:4-H]$^-$	$C_{20}H_{35}O_2^-$
315.045	[M-FA-ketene-H]$^-$	$C_9H_{16}O_{10}P^-$
311.304	[FA20:0-H]$^-$	$C_{20}H_{39}O_2^-$
305.249	[FA20:3-H]$^-$	$C_{20}H_{33}O_2^-$
303.231	[FA20:4-H]$^-$	$C_{20}H_{31}O_2^-$
297.036	[M-FA-FA-H]$^-$	$C_9H_{14}O_9P^-$
283.263	[FA18:0-H]$^-$	$C_{18}H_{35}O_2^-$
281.246	[FA18:1-H]$^-$	$C_{18}H_{33}O_2^-$
259.020	[inositolPO$_3$H$_2$-H]$^-$	$C_6H_{12}O_9P^-$
255.230	[FA16:0-H]$^-$	$C_{16}H_{31}O_2^-$
253.215	[FA16:1-H]$^-$	$C_{16}H_{29}O_2^-$
241.009	[inositolPO$_3$H$_2$-H$_2$O-H]$^-$	$C_6H_{10}O_8P^-$
222.999	[inositolPO$_3$H-2H$_2$O-H]$^-$	$C_6H_8O_7P^-$
152.995	[glycerolPO$_3$H$_2$-H]$^-$	$C_3H_6O_5P^-$

Supplementary Table 10. Overview of GM3-series gangliosides identified in ARSA-/- and ARSA+/+ kidney. Experimental CCS and *m/z* values are presented as mean across n=4 biological replicates and their uncertainties are given as standard deviation in parentheses. All ions detected as $[M-H]^-$.

Name	Chemical sum formula	*m/z* theoretical	1/K0 (V·s/cm²)	CCS (Å²)	m/z timsTOF	ppm timsTOF
GM3 34:1;O2	C57H104N2O21	1151.705882	1.697(1)	343.9(3)	1151.708(1)	2.01
GM3 34:1;O3	C57H104N2O22	1167.700797	1.708(1)	346.2(3)	1167.701(1)	0.20
GM3 36:1;O2	C59H108N2O21	1179.737182	1.730(1)	350.4(2)	1179.737(1)	0.18
GM3 36:1;O3	C59H108N2O22	1195.732097	1.740(3)	352.5(6)	1195.733(3)	0.36
GM3 38:1;O2	C61H112N2O21	1207.768482	1.760(2)	356.5(3)	1207.770(1)	1.69
GM3 38:1;O3	C61H112N2O22	1223.763397	1.769(2)	358.3(3)	1223.764(1)	0.41
GM3 40:1;O2	C63H116N2O21	1235.799782	1.787(2)	361.9(4)	1235.800(3)	-0.03
GM3 40:1;O3	C63H116N2O22	1251.794697	1.796(2)	363.6(4)	1251.798(1)	2.66
GM3 42:1;O2	C65H120N2O21	1263.831082	1.810(2)	366.4(4)	1263.831(3)	-0.22
GM3 42:1;O3	C65H120N2O22	1279.825997	1.819(1)	368.1(3)	1279.829(1)	1.94

Supplementary Table 11. List of all (kidney) sulfatides reported in ARSA-/- mice based on low-resolution MALDI-TOF-MSI (Marsching et al., 2011)[8].

Lipid class	Name	Chemical sum formula	*m/z* theoretical
SM4	SM4 32:1;O2	C38H73NO11S	750.483160
SM4	SM4 32:2;O3	C38H71NO12S	764.462422
SM4	SM4 32:1;O3	C38H73NO12S	766.478070
SM4	SM4 34:2;O2	C40H75NO11S	776.498807
SM4	SM4 34:1;O2	C40H77NO11S	778.514457
SM4	SM4 34:2;O3	C40H75NO12S	792.493722
SM4	SM4 34:1;O3	C40H77NO12S	794.509372
SM4	SM4 34:0;O3	C40H79NO12S	796.525022
SM4	SM4 36:2;O2	C42H79NO11S	804.530107
SM4	SM4 36:1;O2	C42H81NO11S	806.545757
SM4	SM4 34:0;O4	C40H79NO13S	812.519936
SM4	SM4 36:2;O3	C42H79NO12S	820.525022
SM4	SM4 36:1;O3	C42H81NO12S	822.540672
SM4	SM4 38:2;O2	C44H83NO11S	832.561407
SM4	SM4 38:1;O2	C44H85NO11S	834.577057
SM4	SM4 37:1;O3	C43H83NO12S	836.556322
SM4	SM4 38:2;O3	C44H83NO12S	848.556322
SM4	SM4 39:1;O2	C45H87NO11S	848.592707
SM4	SM4 38:1;O3	C44H85NO12S	850,571972
SM4	SM4 40:2;O2	C46H87NO11S	860.592707
SM4	SM4 40:1;O2	C46H89NO11S	862.608357
SM4	SM4 39:1;O3	C45H87NO12S	864.587622
SM4	SM4 38:0;O4	C44H87NO13S	868.582536
SM4	SM4 41:2;O2	C47H89NO11S	874.608357

SM4	SM4 40:2;O3	C46H87NO12S	876.587622
SM4	SM4 41:1;O2	C47H91NO11S	876.624007
SM4	SM4 40:1;O3	C46H89NO12S	878.603272
SM4	SM4 42:3;O2	C48H89NO11S	886.608357
SM4	SM4 42:2;O2	C48H91NO11S	888.624007
SM4	SM4 41:2;O3	C47H89NO12S	890.603272
SM4	SM4 42:1;O2	C48H93NO11S	890.639657
SM4	SM4 41:1;O3	C47H91NO12S	892.618922
SM4	SM4 40:0;O4	C46H91NO13S	896.613837
SM4	SM4 42:3;O3	C48H89NO12S	902.603272
SM4	SM4 42:2;O3	C48H91NO12S	904.618922
SM4	SM4 43:1;O2	C49H95NO11S	904.655307
SM4	SM4 42:1;O3	C48H93NO12S	906.634572
SM4	SM4 44:2;O2	C50H95NO11S	916.655307
SM4	SM4 44:1;O2	C50H97NO11S	918.670957
SM4	SM4 43:1;O3	C49H95NO12S	920.650222
SM4	SM4 42:1;O4	C48H93NO13S	922.629487
SM4	SM4 42:0;O4	C48H95NO13S	924.645137
SM4	SM4 44:1;O3	C50H97NO12S	934.665872
SM3	SM3 34:1;O2	C46H87N1O16S1	940.567281
SM3	SM3 34:1;O3	C46H87N1O17S1	956.562196
SM3	SM3 36:1;O2	C48H91N1O16S1	968.598581
SM3	SM3 38:1;O2	C50H95N1O16S1	996.629881
SM3	SM3 38:1;O3	C50H95N1O17S1	1012.624796
SM3	SM3 38:0;O3	C50H97N1O17S1	1014.640446
SM3	SM3 40:2;O2	C52H97N1O16S1	1022.645531
SM3	SM3 40:1;O2	C52H99N1O16S1	1024.661181
SM3	SM3 40:2;O3	C52H97N1O17S1	1038.640446
SM3	SM3 41:1;O2	C53H101N1O16S1	1038.676831
SM3	SM3 40:1;O3	C52H99N1O17S1	1040.656096
SM3	SM3 40:0;O3	C52H101N1O17S1	1042.671746
SM3	SM3 42:3;O2	C54H99N1O16S1	1048.661181
SM3	SM3 42:2;O2	C54H101N1O16S1	1050.676831
SM3	SM3 42:1;O2	C54H103N1O16S1	1052.692481
SM3	SM3 41:1;O3	C53H101N1O17S1	1054.671746
SM3	SM3 41:0;O3	C53H103N1O17S1	1056.687396
SM3	SM3 40:0;O4	C52H101N1O18S1	1058.666661
SM3	SM3 42:3;O3	C54H99N1O17S1	1064.656096
SM3	SM3 42:2;O3	C54H101N1O17S1	1066.671746
SM3	SM3 43:1;O2	C55H105N1O16S1	1066.708131
SM3	SM3 42:1;O3	C54H103N1O17S1	1068.687396
SM3	SM3 42:0;O3	C54H105N1O17S1	1070.703046
SM3	SM3 44:1;O2	C56H107N1O16S1	1080.723781
SM3	SM3 42:1;O4	C54H103N1O18S1	1084.682311
SM3	SM3 42:0;O4	C54H105N1O18S1	1086.697961
SB1a	SB1a 34:1;O2	C60H110N2O29S2	1305.699477
SB1a	SB1a 34:1;O3	C60H110N2O30S2	1321.694392

SB1a	SB1a 36:1;O2	C62H114N2O29S2	1333.730777
SB1a	SB1a 38:1;O2	C64H118N2O29S2	1361.762077
SB1a	SB1a 38:0;O2	C64H120N2O29S2	1363.777727
SB1a	SB1a 39:1;O2	C65H120N2O29S2	1375.777727
SB1a	SB1a 38:1;O3	C64H118N2O29S2	1377.756991
SB1a	SB1a 38:0;O3	C64H120N2O30S2	1379.772641
SB1a	SB1a 40:2;O2	C66H120N2O29S2	1387.777727
SB1a	SB1a 40:1;O2	C66H122N2O29S2	1389.793377
SB1a	SB1a 39:1;O3	C65H120N2O30S2	1391.772641
SB1a	SB1a 41:1;O2	C67H124N2O29S2	1403.809027
SB1a	SB1a 40:1;O3	C66H122N2O30S2	1405.788292
SB1a	SB1a 40:0;O3	C66H124N2O30S2	1407.803941
SB1a	SB1a 42:2;O2	C68H124N2O29S2	1415.809027
SB1a	SB1a 42:1;O2	C68H126N2O29S2	1417.824677
SB1a	SB1a 41:1;O3	C67H124N2O30S2	1419.803941
SB1a	SB1a 42:2;O3	C68H124N2O30S2	1431.803942
SB1a	SB1a 42:1;O3	C68H126N2O30S2	1433.819592
SB1a	SB1a 42:0;O3	C68H128N2O30S2	1435.835241

Supplementary Table 12. Overview of sulfatides identified in ARSA-/- kidney by LC-ESI-TIMS-TOF-MS. Internal standard marked with asterisk. SM4 and SM3 were detected as $[M-H]^-$, and SB1a were detected as $[M-2H]^{2-}$. Internal standard marked with asterisk.

Name	Chemical sum formula	*m/z* theoretical	CCS (Å²)	t_R	*m/z* timsTOF	ppm timsTOF
SM4 32:1;O3	C38H73NO12S	766.478072	280.3	8.74	766.477	-1.41
SM4 34:2;O2	C40H75NO11S	776.498807	284.2	9.39	776.498	-0.52
SM4 34:1;O2	C40H77NO11S	778.514457	285.5	10.17	778.515	0.43
SM4 34:2;O3	C40H75NO12S	792.493722	285.4	9.10	792.493	-1.04
*SM4 35:1;O2	C41H79NO11S	792.530107	288.8	10.70	792.530	0.10
SM4 34:1;O3	C40H77NO12S	794.509372	286.8	9.89	794.509	0.01
SM4 34:0;O3	C40H79NO12S	796.525022	289.4	9.66	796.525	-0.17
SM4 36:2;O2	C42H79NO11S	804.530107	290.4	10.52	804.530	-0.20
SM4 36:1;O2	C42H81NO11S	806.545757	291.7	11.18	806.544	-1.74
SM4 36:0;O2	C42H83NO11S	808.561407	292.3	11.49	808.562	0.39
SM4 36:2;O3	C42H79NO12S	820.525022	291.6	10.24	820.525	0.19
SM4 36:1;O3	C42H81NO12S	822.540672	292.3	10.91	822.541	0.16
SM4 38:2;O2	C44H83NO11S	832.561407	295.9	11.45	832.562	0.11
SM4 38:1;O2	C44H85NO11S	834.577057	296.5	12.07	834.575	-1.93
SM4 37:1;O3	C43H83NO12S	836.556322	296.4	11.42	836.555	-1.87
SM4 36:0;O4	C42H83NO13S	840.551237	296.2	10.57	840.552	0.65
SM4 38:2;O3	C44H83NO12S	848.556322	297.4	11.20	848.556	-0.38
SM4 39:1;O2	C45H87NO11S	848.592707	298.9	12.54	848.590	-3.11
SM4 38:1;O3	C44H85NO12S	850.571972	297.9	11.84	850.569	0.97

SM4 40:2;O2	C46H87NO11S	860.592707	300.7	12.40	860.588	-1.87
SM4 40:1;O2	C46H89NO11S	862.608357	301.9	12.97	862.606	-2.73
SM4 39:1;O3	C45H87NO12S	864.587622	300.4	12.33	864.587	-0.92
SM4 38:0;O4	C44H87NO13S	868.582536	301.8	11.51	868.582	-0.15
SM4 41:2;O2	C47H89NO11S	874.608357	301.6	12.56	874.609	0.32
SM4 40:2;O3	C46H87NO12S	876.587622	302.2	12.16	876.587	-0.57
SM4 41:1;O2	C47H91NO11S	876.624007	304.3	13.38	876.623	-1.49
SM4 40:1;O3	C46H89NO12S	878.603272	303.3	12.78	878.604	0.60
SM4 42:3;O2	C48H89NO11S	886.608357	303.7	12.52	886.604	-4.91
SM4 42:2;O2	C48H91NO11S	888.624007	304.4	13.25	888.623	-0.79
SM4 41:2;O3	C47H89NO12S	890.603272	304.6	12.59	890.603	-0.53
SM4 42:1;O2	C48H93NO11S	890.639657	306.6	13.77	890.638	-1.31
SM4 41:1;O3	C47H91NO12S	892.618922	305.2	13.17	892.618	-0.54
SM4 40:0;O4	C46H91NO13S	896.613837	305.8	12.44	896.612	-1,71
SM4 42:3;O3	C48H89NO12S	902.603272	305.4	12.28	902.601	-2.18
SM4 42:2;O3	C48H91NO12S	904.618922	307.1	12.86	904.618	-1.32
SM4 42:2;O3	C48H91NO12S	904.618922	307.9	13.09	904.619	0.09
SM4 43:1;O2	C49H95NO11S	904.655307	310.4	14.12	904.655	-0.74
SM4 42:1;O3	C48H93NO12S	906.634572	308.4	13.57	906.634	-1.07
SM4 44:2;O2	C50H95NO11S	916.655307	311.2	13.82	916.654	-1.32
SM4 44:1;O2	C50H97NO11S	918.670957	312.8	14.45	918.670	-0.65
SM4 43:1;O3	C49H95NO12S	920.650222	310.1	13.95	920.649	-1.64
SM4 42:1;O4	C48H93NO13S	922.629487	308.6	12.52	922.628	-2.04
SM4 42:0;O4	C48H95NO13S	924.645137	309.7	13.30	924.644	-1.34
SM4 44:1;O3	C50H97NO12S	934.665872	314.0	14.29	934.668	1.97
SM4 46:1;O2	C52H101NO11S	946.702258	316.9	15.09	946.704	1.41
SM3 34:1;O2	C46H87N1O16S1	940.567281	313.0	10.11	940.566	-1.17
SM3 34:1;O3	C46H87N1O17S1	956.562196	311.6	9.78	956.562	-0.52
SM3 36:1;O2	C48H91N1O16S1	968.598581	319.0	11.13	968.600	0.97
SM3 38:1;O2	C50H95N1O16S1	996.629881	324.0	12.05	996.629	-0.96
SM3 38:1;O3	C50H95N1O17S1	1012.62480	321.7	11.80	1012.622	-2.96
SM3 40:2;O2	C52H97N1O16S1	1022.64553	327.6	12.19	1022.644	-1.49
SM3 40:1;O2	C52H99N1O16S1	1024.66118	329.8	12.91	1024.660	-1.13
SM3 40:0;O2	C52H101NO16S1	1026.67683	330.7	13.22	1026.675	-1.46
SM3 40:2;O3	C52H97N1O17S1	1038.64045	326.3	11.89	1038.640	-0.49
SM3 41:1;O2	C53H101N1O16S1	1038.67683	332.5	13.33	1038.677	-0.20
SM3 40:1;O3	C52H99N1O17S1	1040.65610	327.7	12.59	1040.654	-2.01
SM3 40:0;O3	C52H101N1O17S1	1042.67175	328.0	12.50	1042.670	-1.82
SM3 42:3;O2	C54H99N1O16S1	1048.66118	332.0	12.44	1048.660	-0.89
SM3 42:2;O2	C54H101N1O16S1	1050.67683	333.5	13.03	1050.675	-1.85
SM3 42:1;O2	C54H103N1O16S1	1052.69248	335.6	13.71	1052.692	-0.27
SM3 41:1;O3	C53H101N1O17S1	1054.67175	330.1	13.08	1054.673	1.05
SM3 42:0;O2	C54H105NO16S1	1054.70813	336.8	13.95	1054.707	-0.66
SM3 41:0;O3	C53H103N1O17S1	1056.68740	330.4	12.94	1056.689	1.04
SM3 40:0;O4	C52H101N1O18S1	1058.66666	326.4	12.29	1058.665	-1,76
SM3 42:2;O3	C54H101N1O17S1	1066.67175	331.4	12.75	1066.669	-2.86
SM3 43:1;O2	C55H105N1O16S1	1066.70813	337.5	14.05	1066.708	0.20
SM3 42:1;O3	C54H103N1O17S1	1068.68740	333.3	13.47	1068.686	-1.71
SM3 42:0;O3	C54H105N1O17S1	1070.70305	333.3	13.34	1070.701	-1.75
SM3 44:1;O2	C56H107N1O16S1	1080.72378	340.4	14.39	1080.722	-1.21
SM3 42:1;O4	C54H103N1O18S1	1084.68231	330.3	12.40	1084.681	-0.93
SM3 42:0;O4	C54H105N1O18S1	1086.69796	331.5	13.14	1086.696	-2.08
SB1a 34:1;O2	C60H110N2O29S2	692.32451	395.1	8.77	692.325	0.15

SB1a 34:1;O3	C60H110N2O30S2	700.32187	396.1	8.50	700.321	-1.19
SB1a 36:1;O2	C62H114N2O29S2	706.34016	400.9	9.85	706.339	-1.07
SB1a 37:1;O2	C63H116N2O29S2	713.34798	402.8	10.36	713.348	-0.48
SB1a 36:1;O3	C62H114N2O30S2	714.33762	401.5	9.60	714.337	-0.71
SB1a 38:1;O2	C64H118N2O29S2	720.35581	405.4	10.83	720.355	-1.06
SB1a 38:0;O2	C64H120N2O29S2	721.36363	405.1	11.15	721.364	0.00
SB1a 38:2;O3	C64H116N2O30S2	727.34544	405.4	9.94	727.345	-0.19
SB1a 39:1;O2	C65H120N2O29S2	727.36363	406.7	11.32	727.362	-1.74
SB1a 38:1;O3	C64H118N2O30S2	728.35327	406.0	10.61	728.353	-0.90
SB1a 38:0;O3	C64H120N2O30S2	729.36109	407.3	10.42	729.360	-0.86
SB1a 40:2;O2	C66H120N2O29S2	733.36363	409.3	10.97	733.363	-0.53
SB1a 40:1;O2	C66H122N2O29S2	734.37146	410.3	11.71	734.371	-0.35
SB1a 39:1;O3	C65H120N2O30S2	735.36109	408.0	11.08	735.362	0.58
SB1a 41:1;O2	C67H124N2O29S2	741.37928	411.9	12.17	741.379	-0.34
SB1a 40:1;O3	C66H122N2O30S2	742.36892	410.2	11.50	742.369	0.60
SB1a 40:0;O3	C66H124N2O30S2	743.37674	411.8	11.35	743.377	-0.04
SB1a 42:2;O2	C68H124N2O29S2	747.37928	414.2	11.77	747.379	-0.53
SB1a 42:1;O2	C68H126N2O29S2	748.38711	414.1	12.57	748.387	-0.59
SB1a 41:1;O3	C67H124N2O30S2	749.37674	413.1	11.94	749.375	-1.85
SB1a 42:3;O3	C68H122N2O30S2	754.36892	413.1	11.03	754.368	-1.31
SB1a 42:2;O3	C68H124N2O30S2	755.37674	415.1	11.61	755.377	-0.09
SB1a 42:1;O3	C68H126N2O30S2	756.38457	415.1	12.38	756.385	-0.09
SB1a 42:0;O3	C68H128N2O30S2	757.39239	416.4	12.21	757.393	0.46
SB1a 44:1;O2	C70H130N2O29S2	762.40276	419.6	13.32	762.402	-1.10
SB1a 42:1;O4	C68H126N2O31S2	764.38202	417.0	11.32	764.383	0.80
SB1a 42:0;O4	C68H128N2O31S2	765.38985	416.7	12.04	765.389	-1.51

Supplementary Table 13. Overview of sulfatides identified in ARSA-/- kidney by MALDI-MSI. Experimental CCS and *m/z* values are presented as mean across n=4 biological replicates and their uncertainties are given as standard deviation in parentheses. Internal standard marked with asterisk. Sulfatides with predominant accumulation in cortex region are marked in *italics*.

Name	Chemical sum formula	Adduct	*m/z* theoretical	CCS (Å²)	*m/z* timsTOF	ppm
SM4 18:1;O2	C24H47NO10S	$[M-H]^-$	540.284791	228.9(3)	540.285(1)	1.13
SM4 18:0;O3	C24H49NO11S	$[M-H]^-$	558.295356	233.7(3)	558.297(1)	2.50
SM4 32:2;O2	C38H71NO11S	$[M-H]^-$	748.467507	275.4(2)	748.468(1)	0.26
SM4 32:1;O2	C38H73NO11S	$[M-H]^-$	750.483157	276.7(2)	750.482(1)	-1.21
SM4 32:2;O3	C38H71NO12S	$[M-H]^-$	764.462422	277.8(3)	764.463(1)	0.89
SM4 32:1;O3	C38H73NO12S	$[M-H]^-$	766.478072	278.8(3)	766.476(1)	-2.70
SM4 34:2;O2	C40H75NO11S	$[M-H]^-$	776.498807	281.8(2)	776.498(1)	-0.72
SM4 33:2;O3	C39H73NO12S	$[M-H]^-$	778.478072	279.3(3)	778.477(1)	-1.44
SM4 34:1;O2	C40H77NO11S	$[M-H]^-$	778.514457	283.2(3)	778.515(1)	0.09
SM4 34:2;O3	C40H75NO12S	$[M-H]^-$	792.493722	283.5(4)	792.493(1)	-1.54
*SM4 35:1;O2	C41H79NO11S	$[M-H]^-$	792.530107	286.4(3)	792.531(1)	0.91
SM4 34:1;O3	C40H77NO12S	$[M-H]^-$	794.509372	284.8(2)	794.509(1)	0.00
SM4 34:0;O3	C40H79NO12S	$[M-H]^-$	796.525022	288.0(4)	796.527(1)	2.61
SM4 36:2;O2	C42H79NO11S	$[M-H]^-$	804.530107	288.6(3)	804.529(1)	-0.82
SM4 36:1;O2	C42H81NO11S	$[M-H]^-$	806.545757	289.7(2)	806.547(1)	1.11
SM4 34:0;O4	C40H79NO13S	$[M-H]^-$	812.519936	288.8(4)	812.520(1)	0.23
SM4 36:2;O3	C42H79NO12S	$[M-H]^-$	820.525022	290.0(5)	820.523(1)	-2.04
SM4 37:1;O2	C43H83NO11S	$[M-H]^-$	820.561407	292.3(3)	820.561(2)	-0.86
SM4 36:1;O3	C42H81NO12S	$[M-H]^-$	822.540672	291.3(3)	822.541(1)	-0.15
SM4 36:0;O3	C42H83NO12S	$[M-H]^-$	824.556322	293.4(3)	824.557(2)	1.06
SM4 38:2;O2	C44H83NO11S	$[M-H]^-$	832.561407	293.4(2)	832.562(1)	0.47
SM4 38:1;O2	C44H85NO11S	$[M-H]^-$	834.577057	294.6(3)	834.577(1)	-0.40
SM4 37:1;O3	C43H83NO12S	$[M-H]^-$	836.556322	293.4(2)	836.555(1)	-1.10
SM4 36:0;O4	C42H83NO13S	$[M-H]^-$	840.551237	294.1(3)	840.551(1)	0.22
SM4 39:2;O2	C45H85NO11S	$[M-H]^-$	846.577057	296.2(2)	846.578(1)	0.60
SM4 38:2;O3	C44H83NO12S	$[M-H]^-$	848.556322	295.3(3)	848.557(1)	0.89
SM4 39:1;O2	C45H87NO11S	$[M-H]^-$	848.592707	297.3(3)	848.591(1)	-1.69
SM4 38:1;O3	C44H85NO12S	$[M-H]^-$	850.571972	296.5(3)	850.572(1)	-0.55
SM4 38:0;O3	C44H87NO12S	$[M-H]^-$	852.587622	298.7(2)	852.587(2)	-1.08
SM4 40:2;O2	C46H87NO11S	$[M-H]^-$	860.592707	298.5(3)	860.594(1)	0.92
SM4 40:1;O2	C46H89NO11S	$[M-H]^-$	862.608357	299.5(3)	862.609(1)	0.28
SM4 39:1;O3	C45H87NO12S	$[M-H]^-$	864.587622	298.4(3)	864.588(1)	0.15
SM4 39:0;O3	C45H89NO12S	$[M-H]^-$	866.603272	301.1(2)	866.604(1)	1.24
SM4 38:0;O4	C44H87NO13S	$[M-H]^-$	868.582536	299.2(3)	868.582(1)	-0.10
SM4 41:2;O2	C47H89NO11S	$[M-H]^-$	874.608357	301.8(3)	874.606(1)	-2.35
SM4 40:2;O3	C46H87NO12S	$[M-H]^-$	876.587622	300.1(3)	876.587(1)	-0.54
SM4 41:1;O2	C47H91NO11S	$[M-H]^-$	876.624007	302.8(4)	876.623(1)	-0.81
SM4 40:1;O3	C46H89NO12S	$[M-H]^-$	878.603272	301.4(4)	878.604(1)	0.57
SM4 40:0;O3	C46H91NO12S	$[M-H]^-$	880.618922	304.0(2)	880.619(1)	0.26
SM4 39:0;O4	C45H89NO13S	$[M-H]^-$	882.598187	302.2(1)	882.599(1)	0.35
SM4 42:3;O2	C48H89NO11S	$[M-H]^-$	886.608357	302.9(5)	886.608(1)	-0.52
SM4 42:2;O2	C48H91NO11S	$[M-H]^-$	888.624007	303.8(4)	888.623(1)	-1.44
SM4 41:2;O3	C47H89NO12S	$[M-H]^-$	890.603272		*890.603(1)*	*-0.53*
SM4 42:1;O2	C48H93NO11S	$[M-H]^-$	890.639657	305.3(4)	890.638(1)	-1.33
SM4 41:1;O3	C47H91NO12S	$[M-H]^-$	892.618922	303.8(4)	892.619(1)	-0.02
SM4 41:0;O3	C47H93NO12S	$[M-H]^-$	894.634572	306.0(4)	894.635(2)	0.34
SM4 40:0;O4	C46H91NO13S	$[M-H]^-$	896.613837	304.2(3)	896.614(1)	0.60
SM4 42:3;O3	C48H89NO12S	$[M-H]^-$	902.603272	303.5(4)	902.603(1)	-0.72
SM4 42:2;O3	C48H91NO12S	$[M-H]^-$	904.618922	304.8(3)	904.619(1)	-0.33
SM4 43:1;O2	C49H95NO11S	$[M-H]^-$	904.655307	308.3(5)	904.654(1)	-1.17
SM4 42:1;O3	C48H93NO12S	$[M-H]^-$	906.634572	306.3(5)	906.635(1)	0.09
SM4 42:0;O3	C48H95NO12S	$[M-H]^-$	908.650222	309.1(1)	908.651(1)	1.30
SM4 41:0;O4	C47H93NO13S	$[M-H]^-$	910.629487	306.5(4)	910.630(1)	0.62
SM4 44:3;O2	C50H93NO11S	$[M-H]^-$	914.639657	307.7(3)	914.640(1)	0.18

SM4 44:2;O2	C50H95NO11S	$[M-H]^-$	916.655307	309.3(5)	916.656(1)	0.65
SM4 44:1;O2	C50H97NO11S	$[M-H]^-$	918.670957	310.9(5)	918.671(1)	0.05
SM4 43:1;O3	C49H95NO12S	$[M-H]^-$	920.650222	309.3(7)	920.650(1)	-0.43
SM4 42:1;O4	C48H93NO13S	$[M-H]^-$	922.629487	307.8(4)	922.629(1)	-0.26
SM4 42:0;O4	C48H95NO13S	$[M-H]^-$	924.645137	308.6(4)	924.644(1)	-0.85
SM4 44:2;O3	C50H95NO12S	$[M-H]^-$	932.650222	310.7(5)	932.651(1)	1.02
SM4 44:1;O3	C50H97NO12S	$[M-H]^-$	934.665872	311.8(5)	934.666(1)	-0.16
SM4 43:0;O4	C49H97NO13S	$[M-H]^-$	938.660787	311.0(5)	938.660(1)	-1.05
SM4 46:1;O2	C52H101NO11S	$[M-H]^-$	946.702258	315.9(3)	946.700(1)	-2.33
SM4 44:0;O4	C50H99NO13S	$[M-H]^-$	952.676438	314.6(1)	952.674(1)	-2.51
SM3 18:1;O2	C30H57N1O15S	$[M-H]^-$	702.337615	259.6(1)	702.337(1)	-1.52
SM3 18:0;O3	C30H59N1O16S	$[M-H]^-$	720.348180	262.3(1)	720.347(1)	-2.19
SM3 34:1;O2	C46H87N1O16S	$[M-H]^-$	940.567281	311.4(4)	940.567(1)	-0.64
SM3 34:1;O3	C46H87N1O17S	$[M-H]^-$	956.562196	310.5(5)	956.562(1)	-0.02
SM3 36:1;O2	C48H91N1O16S	$[M-H]^-$	968.598581	317.0(4)	968.597(1)	-1.61
SM3 36:1;O3	C48H91N1O17S	$[M-H]^-$	984.593496	316.4(2)	984.592(1)	-1.52
SM3 38:2;O2	C50H93N1O16S	$[M-H]^-$	994.614231	322.1(4)	994.612(1)	-2.22
SM3 38:1;O2	C50H95N1O16S	$[M-H]^-$	996.629881	322.8(3)	996.629(1)	-0.48
SM3 38:2;O3	C50H93N1O17S	$[M-H]^-$	1010.609146	321.3(1)	1010.609(1)	-0.34
SM3 39:1;O2	C51H97N1O16S	$[M-H]^-$	1010.645531	325.6(4)	1010.643(1)	-2.11
SM3 38:1;O3	C50H95N1O17S	$[M-H]^-$	1012.624796	321.5(3)	1012.623(1)	-2.07
SM3 38:0;O3	C50H97N1O17S	$[M-H]^-$	1014.640446		*1014.639(1)*	*-1.13*
SM3 40:2;O2	C52H97N1O16S	$[M-H]^-$	1022.645531	327.4(4)	1022.645(1)	-0.84
SM3 40:1;O2	C52H99N1O16S	$[M-H]^-$	1024.661181	328.3(4)	1024.662(1)	1.17
SM3 38:0;O4	C50H97N1O18S	$[M-H]^-$	1030.635360	320.7(2)	1030.637(1)	1.83
SM3 40:2;O3	C52H97N1O17S	$[M-H]^-$	1038.640446	325.7(3)	1038.640(2)	-0.24
SM3 41:1;O2	C53H101N1O16S	$[M-H]^-$	1038.676831	331.0(4)	1038.676(1)	-0.63
SM3 40:1;O3	C52H99N1O17S	$[M-H]^-$	1040.656096	326.5(3)	1040.657(1)	0.82
SM3 40:0;O3	C52H101N1O17S	$[M-H]^-$	1042.671746		*1042.673(1)*	*0.82*
SM3 42:3;O2	C54H99N1O16S	$[M-H]^-$	1048.661181	330.8(3)	1048.660(1)	-1.05
SM3 42:2;O2	C54H101N1O16S	$[M-H]^-$	1050.676831	331.9(4)	1050.676(1)	-1.15
SM3 42:1;O2	C54H103N1O16S	$[M-H]^-$	1052.692481	333.7(4)	1052.693(1)	0.30
SM3 41:1;O3	C53H101N1O17S	$[M-H]^-$	1054.671746	329.6(1)	1054.671(1)	-1.09
SM3 41:0;O3	C53H103N1O17S	$[M-H]^-$	1056.687396		*1056.689(1)*	*1.49*
SM3 40:0;O4	C52H101N1O18S	$[M-H]^-$	1058.666661	325.0(4)	1058.666(2)	-0.34
SM3 42:3;O3	C54H99N1O17S	$[M-H]^-$	1064.656096	335.2(6)	1064.666(3)	9.23
SM3 42:2;O3	C54H101N1O17S	$[M-H]^-$	1066.671746	330.1(4)	1066.672(1)	-0.16
SM3 43:1;O2	C55H105N1O16S	$[M-H]^-$	1066.708131	336.5(5)	1066.709(1)	0.63
SM3 42:1;O3	C54H103N1O17S	$[M-H]^-$	1068.687396	331.7(3)	1068.688(1)	0.89
SM3 42:0;O3	C54H105N1O17S	$[M-H]^-$	1070.703046		*1070.702(1)*	*-0.98*
SM3 41:0;O4	C53H103N1O18S	$[M-H]^-$	1072.682311	327.5(2)	1072.682(1)	-0.20
SM3 44:2;O2	C56H105N1O16S	$[M-H]^-$	1078.708131	337.2(5)	1078.708(1)	-0.05
SM3 44:1;O2	C56H107N1O16S	$[M-H]^-$	1080.723781	339.0(4)	1080.725(1)	1.17
SM3 42:1;O4	C54H103N1O18S	$[M-H]^-$	1084.682311	328.9(1)	1084.681(1)	-1.35
SM3 42:0;O4	C54H105N1O18S	$[M-H]^-$	1086.697961	330.0(3)	1086.698(1)	-0.19
SM3 44:1;O3	C56H107N1O17S	$[M-H]^-$	1096.718696	337.2(4)	1096.718(1)	-0.50
SM3 46:1;O2	C58H111N1O16S	$[M-H]^-$	1108.755082	344.4(6)	1108.757(2)	1.77
SM2a 38:1;O2	C58H108N2O21S	$[M-H]^-$	1199.709253	352.3(1)	1199.709(1)	-0.38
SM2a 38:1;O3	C58H108N2O22S	$[M-H]^-$	1215.704168	351.9(2)	1215.704(1)	-0.14
SM2a 40:1;O2	C60H112N2O21S	$[M-H]^-$	1227.740553	357.7(1)	1227.739(1)	-1.00
SM2a 40:1;O3	C60H112N2O22S	$[M-H]^-$	1243.735468	356.4(1)	1243.735(3)	-0.74
SM2a 42:1;O2	C62H116N2O21S	$[M-H]^-$	1255.771853	362.8(1)	1255.772(1)	0.38
SM2a 42:1;O3	C62H116N2O22S	$[M-H]^-$	1271.766768	361.1(2)	1271.768(4)	0.77
SM1b 34:1;O2	C60H110N2O26S	$[M-H]^-$	1305.699477	355.3(2)	1305.699(5)	-0.49
SM1b 34:1;O3	C60H110N2O27S	$[M-H]^-$	1321.694392	357.1(1)	1321.695(3)	0.79
SM1b 36:1;O2	C62H114N2O26S	$[M-H]^-$	1333.730777	360.2(4)	1333.732(3)	0.83
SM1b 36:1;O3	C62H114N2O27S	$[M-H]^-$	1349.725692	362.1(4)	1349.727(1)	1.17
SM1b 38:1;O2	C64H118N2O26S	$[M-H]^-$	1361.762077	365.0(2)	1361.764(2)	1.51
SM1b 39:1;O2	C65H120N2O26S	$[M-H]^-$	1375.777727	367.4(2)	1375.775(5)	-1.67
SM1b 38:1;O3	C64H118N2O26S	$[M-H]^-$	1377.756991	363.6(3)	1377.753(1)	-2.71
SM1b 40:2;O2	C66H120N2O26S	$[M-H]^-$	1387.777727	369.2(2)	1387.777(1)	-0.36
SM1b 40:1;O2	C66H122N2O26S	$[M-H]^-$	1389.793377	370.0(2)	1389.793(1)	-0.50
SM1b 38:0;O4	C64H120N2O28S	$[M-H]^-$	1395.767556	366(1)	1395.767(1)	-0.69
SM1b 41:1;O2	C67H124N2O26S	$[M-H]^-$	1403.809027	372.5(1)	1403.809(1)	-0.34
SM1b 40:1;O3	C66H122N2O27S	$[M-H]^-$	1405.788292	372.5(2)	1405.792(3)	2.44
SM1b 42:2;O2	C68H124N2O26S	$[M-H]^-$	1415.809027	373.5(2)	1415.812(1)	1.82

SM1b 42:1;O2	C68H126N2O26S	[M-H]$^-$	1417.824677	375.0(2)	1417.824(1)	-0.57
SM1b 40:0;O4	C66H124N2O28S	[M-H]$^-$	1423.798857	370(2)	1423.805(1)	4.21
SM1b 42:2;O3	C68H124N2O27S	[M-H]$^-$	1431.803942	376.1(2)	1431.803(3)	-0.47
SM1b 42:1;O3	C68H126N2O27S	[M-H]$^-$	1433.819592	377.2(3)	1433.821(1)	0.69
SM1b 41:0;O4	C67H126N2O28S	[M-H]$^-$	1437.814507	373(2)	1437.815(1)	0.17
SM1b 44:1;O2	C70H130N2O26S	[M-H]$^-$	1445.855977	381(1)	1445.856(1)	-0.21
SM1b 42:0;O4	C68H128N2O28S	[M-H]$^-$	1451.830157	374(2)	1451.828(1)	-1.40
SM1a 34:1;O2	C60H110N2O26S	[M-H]$^-$	1305.699477	362.6(2)	1305.699(5)	-0.49
SM1a 34:1;O3	C60H110N2O27S	[M-H]$^-$	1321.694392	361.2(1)	1321.695(3)	0.79
SM1a 36:1;O2	C62H114N2O26S	[M-H]$^-$	1333.730777	367.7(2)	1333.732(3)	0.83
SM1a 36:1;O3	C62H114N2O27S	[M-H]$^-$	1349.725692	365.8(3)	1349.727(1)	1.17
SM1a 38:1;O2	C64H118N2O26S	[M-H]$^-$	1361.762077	372.5(2)	1361.764(2)	1.51
SM1a 39:1;O2	C65H120N2O26S	[M-H]$^-$	1375.777727	374.9(3)	1375.775(5)	-1.67
SM1a 38:1;O3	C64H118N2O26S	[M-H]$^-$	1377.756991	368.5(5)	1377.753(1)	-2.71
SM1a 40:2;O2	C66H120N2O26S	[M-H]$^-$	1387.777727	375.7(2)	1387.777(1)	-0.36
SM1a 40:1;O2	C66H122N2O26S	[M-H]$^-$	1389.793377	377.2(2)	1389.793(1)	-0.50
SM1a 38:0;O4	C64H120N2O28S	[M-H]$^-$	1395.767556	368(1)	1395.767(1)	-0.69
SM1a 41:1;O2	C67H124N2O26S	[M-H]$^-$	1403.809027	379.9(2)	1403.809(1)	-0.34
SM1a 42:2;O2	C68H124N2O26S	[M-H]$^-$	1415.809027	381.2(2)	1415.812(4)	1.82
SM1a 42:1;O2	C68H126N2O26S	[M-H]$^-$	1417.824677	382.2(2)	1417.824(1)	-0.57
SM1a 40:0;O4	C66H124N2O28S	[M-H]$^-$	1423.798857	373(1)	1423.805(1)	4.21
SM1a 41:0;O4	C67H126N2O28S	[M-H]$^-$	1437.814507	376(1)	1437.815(1)	0.17
SM1a 44:1;O2	C70H130N2O26S	[M-H]$^-$	1445.855977	389(1)	1445.856(1)	-0.21
SM1a 42:0;O4	C68H128N2O28S	[M-H]$^-$	1451.830157	378(1)	1451.828(1)	-1.40
SB1a 34:1;O2	C60H109N2NaO29S2	[M+Na-2H]$^-$	1407.638235	361.4(2)	1407.638(1)	0.03
SB1a 34:1;O3	C60H109N2NaO30S2	[M+Na-2H]$^-$	1423.633150	363.2(2)	1423.634(1)	0.93
SB1a 36:1;O2	C62H113N2NaO29S2	[M+Na-2H]$^-$	1435.669535	366(1)	1435.672(2)	1.77
SB1a 36:1;O3	C62H113N2NaO30S2	[M+Na-2H]$^-$	1451.664450	367.9(3)	1451.660(1)	-3.41
SB1a 38:1;O2	C64H117N2NaO29S2	[M+Na-2H]$^-$	1463.700836	371.0(1)	1463.700(1)	-0.76
SB1a 38:1;O3	C64H117N2NaO30S2	[M+Na-2H]$^-$	1479.695750	372.7(3)	1479.692(1)	-2.86
SB1a 40:2;O2	C66H119N2NaO29S2	[M+Na-2H]$^-$	1489.716486	375.1(4)	1489.713(1)	-2.31
SB1a 40:1;O2	C66H121N2NaO29S2	[M+Na-2H]$^-$	1491.732136	376.3(1)	1491.734(2)	1.25
SB1a 38:0;O4	C64H119N2NaO31S2	[M+Na-2H]$^-$	1497.706315	376.4(8)	1497.704(1)	-1.41
SB1a 40:2;O3	C66H119N2NaO30S2	[M+Na-2H]$^-$	1505.711400	376.6(3)	1505.715(1)	2.70
SB1a 40:1;O3	C66H121N2NaO30S2	[M+Na-2H]$^-$	1507.727050	377.8(3)	1507.726(1)	-0.65
SB1a 42:2;O2	C68H123N2NaO29S2	[M+Na-2H]$^-$	1517.747786	379.9(1)	1517.750(1)	1.13
SB1a 42:1;O2	C68H125N2NaO29S2	[M+Na-2H]$^-$	1519.763436	381.5(2)	1519.761(1)	-1.69
SB1a 40:0;O4	C66H123N2NaO31S2	[M+Na-2H]$^-$	1525.737615	380.7(2)	1525.730(1)	-4.77
SB1a 42:2;O3	C68H123N2NaO30S2	[M+Na-2H]$^-$	1533.742700	381.7(1)	1533.740(1)	-1.84
SB1a 42:1;O3	C68H125N2NaO30S2	[M+Na-2H]$^-$	1535.758350	383.1(1)	1535.753(4)	-3.76
SB1a 41:0;O4	C67H125N2NaO31S2	[M+Na-2H]$^-$	1539.753265	383.8(8)	1539.754(1)	0.48
SB1a 44:2;O2	C70H127N2NaO29S2	[M+Na-2H]$^-$	1545.779086	384.8(2)	1545.786(2)	4.34
SB1a 44:1;O2	C70H129N2NaO29S2	[M+Na-2H]$^-$	1547.794736	387(1)	1547.797(1)	1.61
SB1a 42:0;O4	C68H127N2NaO31S2	[M+Na-2H]$^-$	1553.768915	387(2)	1553.769(1)	-0.11
SB1a 44:1;O3	C70H129N2NaO30S2	[M+Na-2H]$^-$	1563.789651	388.1(1)	1563.781(8)	-5.64

Supplementary Table 14. Overview of significant features in EAE mouse spinal cord lesions. Experimental CCS and *m/z* values are presented as mean across n=3 biological replicates and their uncertainties are given as standard deviation in parentheses. All ions detected as $[M\text{-}H]^-$.

Name	Chemical sum formula	*m/z* theoretical	CCS (Å²)	*m/z* timsTOF	ppm
CerP 34:1;O2	C34H68NO6P	616.471149	255.3(2)	616.472(1)	1.22
CerPE 36:1;O2	C38H77N2O6P	687.544648	269.7(1)	687.543(1)	-2.25
PE 38:4	C43H78NO8P	766.539229	279.6(1)	766.538(1)	-1.56
PI 36:4	C45H79O13P	857.518553	295.0(1)	857.519(1)	0.33
PI 38:4	C47H83O13P	885.549853	300.9(1)	885.549(1)	-0.70
SM4 42:2;O2	C48H91NO11S	888.624007	304.6(1)	888.624(1)	-0.12
PI 40:4	C49H87O13P	913.581153	306.9(1)	913.581(1)	-0.06

Supplementary Table 15. Identified fragment ions of CerP 34:1$[M\text{-}H]^-$ (*m/z* 616.467). Isolated at $1/K_0$ = 1.246Vs/cm² and fragmented with -40.0 eV.

m/z measured	Fragment ion	Sum formula
616.467	$[M\text{-}H]^-$	$C_{34}H_{67}NO_6P^-$
78.959	metaphosphate	O_3P^-

Supplementary Table 16. Identified fragment ions of CerPE 36:1$[M\text{-}H]^-$ (*m/z* 687.542). Isolated at $1/K_0$ = 1.313 Vs/cm² and fragmented with -40.0 eV. Fragment patterns were modelled according to Hsu and Turk, 2009[12]. CerPE 18:1;O2/18:0 was identified as the predominant isomeric structure.

m/z measured	Fragment ion	Sum formula
687.542	$[M\text{-}H]^-$	$C_{38}H_{76}N_2O_6P^-$
405.274	$[M\text{-}18\text{:}0amide\text{-}H]^-$	$C_{20}H_{40}NO_5P^-$
377.242	$[M\text{-}20\text{:}0amide\text{-}H]^-$	$C_{18}H_{36}NO_5P^-$
168.041	$[phosphorylcholine]^-$	$C_4H_{11}NO_4P^-$

Supplementary Table 17. Identified fragment ions of PE 38:4[M-H]$^-$ (*m/z* 766.535). Isolated at $1/K_0$ = 1.352 Vs/cm² and fragmented with -41.5 eV. Fragment patterns were modelled according to Hsu and Turk, 2009[12]. PE 18:0/20:4 was identified as the predominant isomeric structure[12].

m/z measured	Fragment ion	Sum formula
766.535	[M-H]$^-$	$C_{43}H_{77}NO_8P^-$
480.307	[M-20:4ketene-H]$^-$	$C_{23}H_{47}NO_7P^-$
331.257	[FA22:4-H]$^-$	$C_{22}H_{35}O_2$-
303.230	[FA20:4-H]$^-$	$C_{20}H_{31}O_2$-
283.258	[FA18:0-H]$^-$	$C_{18}H_{35}O_2$-
255.232	[FA16:0-H]$^-$	$C_{16}H_{31}O_2$-

Supplementary Table 18. Identified fragment ions of PI 36:4[M-H]$^-$ (*m/z* 857.519). Isolated at $1/K_0$ = 1.427 Vs/cm² and fragmented with -53.5 eV. Fragment patterns were modelled according to Hsu and Turk, 2009[12]. PI 16:0/20:4 was identified as the predominant isomeric structure[12].

m/z measured	Fragment ion	Sum formula
857.519	[M-H]$^-$	$C_{45}H_{78}O_{13}P^-$
553.276	[M-FA20:4-H]$^-$	$C_{25}H_{46}O_{11}P^-$
303.233	[FA20:4-H]$^-$	$C_{20}H_{31}O_2$-
152.994	[glycerolPO$_3$H$_2$-H]$^-$	$C_3H_6O_5P^-$

Supplementary Table 19. Identified fragment ions of PI 38:4[M-H]$^-$ (*m/z* 885.546). Isolated at $1/K_0$ = 1.457 Vs/cm² and fragmented with -55.5 eV. Fragment patterns were modelled according to Hsu and Turk, 2009[12]. PI 18:0/20:4 was identified as the predominant isomeric structure.

m/z measured	Fragment ion	Sum formula
885.546	[M-H]$^-$	$C_{47}H_{82}O_{13}P^-$
599.320	[M-20:4ketene-H]$^-$	$C_{27}H_{52}O_{12}P^-$
581.307	[M-FA20:4-H]$^-$	$C_{27}H_{50}O_{11}P^-$
419.253	[M-FA20:4-inositol-H]$^-$	$C_{21}H_{40}O_6P^-$
315.045	[M-FA-ketene-H]$^-$	$C_9H_{16}O_{10}P^-$
303.230	[FA20:4-H]$^-$	$C_{20}H_{31}O_2$-
283.263	[FA18:0-H]$^-$	$C_{18}H_{35}O_2$-
259.019	[inositolPO$_3$H$_2$-H]$^-$	$C_6H_{12}O_9P^-$
241.010	[inositolPO$_3$H$_2$-H$_2$O-H]$^-$	$C_6H_{10}O_8P^-$
223.000	[inositolPO$_3$H-2H$_2$O-H]$^-$	$C_6H_8O_7P^-$
152.994	[glycerolPO$_3$H$_2$-H]$^-$	$C_3H_6O_5P^-$

Supplementary Table 20. Identified fragment ions of SM4 42:2;O2[M-H]$^-$ (*m/z* 888.620). Isolated at $1/K_0$ = 1.499 Vs/cm² and fragmented with -60.0 eV.

***m/z* measured**	**Fragment ion**	**Sum formula**
888.620	$[M-H]^-$	$C_{48}H_{90}NO_{11}S^-$
259.011	$[Gal-SO_3+H_2O]^-$	$C_6H_{11}O_9S^-$
241.000	$[Gal-SO_3]^-$	$C_6H_9O_8S^-$
96.958	$[sulfate]^-$	HO_4S^-

Supplementary Table 21. Identified fragment ions of PI 40:4[M-H]$^-$ (*m/z* 913.581). Isolated at $1/K_0$ = 1.485 Vs/cm² and fragmented with -56.4 eV. Fragment patterns were modelled according to Hsu and Turk, 2009[12]. PI 18:0/22:4 was identified as the predominant isomeric structure[12].

***m/z* measured**	**Fragment ion**	**Sum formula**
913.581	$[M-H]^-$	$C_{49}H_{86}O_{13}P^-$
331.263	$[FA22:4-H]^-$	$C_{22}H_{35}O_2^-$
283.263	$[FA18:0-H]^-$	$C_{18}H_{35}O_2^-$
241.010	$[inositolPO_3H_2-H_2O-H]^-$	$C_6H_{10}O_8P^-$

Supplementary Methods

Hematoxylin & Eosin Staining

Matrix removal was performed by submerging the slide in 70 % EtOH for 1 min. For subsequent H&E staining, the SunTissuePrep System (SunChrom, Friedrichsdorf, Germany) was used. The staining procedure consists of the following steps as described previously[1]: 1.5 min hemalum (Mayer's hemalum solution, Sigma-Aldrich), 2 min tap water, 1 min deionized water, 1 min acidic (350 mL ethanol + 150 mL H_2O + 1.5 mL HCl) ethanol (absolute EMPLURA®, Merck; HCL Titripur, Merck), 45 s deionized water, 2 min bluing solution (2 g $NaHCO_3$ + 20 g $MgSO_4$ in 1L H_2O) ($NaHCO_3$, Merck) ($MgSO_4$, VWR Chemicals), 1 min deionized water, 2 min Eosin (Eosin Y-solution 0.5 % aqueous, Merck), 45 s deionized water, 1 min 80 % EtOH, 2 min 96 % EtOH, 2 min 100 % EtOH, and 3 min xylene. The slides were cover-slipped with Eukitt (Sigma-Aldrich) and a glass cover slide (VWR Chemicals) for long-term preservation.

MALDI-TIMS-TOF Mass Spectrometry Imaging

Embedding of spinal cord tissue samples

Spinal cords were embedded using a modified version of a published protocol[2]. Briefly, 7.5 g hydroxypropyl methyl cellulose (HPMC) and 2.5 g polyvinylpyrrolidone (PVP) were added to 100 ml of deionized water and stirred for 1 hour. The mixture was kept at 4 °C overnight to ensure complete dissolution of the polymers. 3 mL aliquots were centrifuged for 5 min at 800 g to remove trapped air. Finally, the embedding medium was stored at -20 °C until use.

Matrix spray-coating

ARSA mouse kidney tissue sections: 10 mg/mL DHAP was dissolved in 70% ACN with 125 mM ammonium sulfate. After sonication, 0.1% TFA and 3 µM of SM4 35:1;O2 (100 µg/mL (= 157.41 µM) in MeOH/chloroform 2:1) as internal standard (IS) were added. Matrix was applied with an M5 TM-Sprayer (HTX Technologies, Chapel Hill, USA). Temperatures of the spray nozzle and tray were 75 °C and 35 °C, respectively. The

spraying parameters were as follows: Spray Nozzle Velocity: 1200 mm/min; Flow Rate: 0.1 mL/min; No. of Passes: 10; Track Spacing: 2 mm; Pattern: HH; Pressure: 10 psi; Gas Low Rate: 2 L/min; Nozzle Height: 40 mm; Drying Time: 0s.

Spheroid sections: were coated with DAN (10 mg/mL) in ACN/water 7:3 (*v/v*). The spraying parameters were as follows: Spray Nozzle Velocity: 1350 mm/min; Flow Rate: 0.07 mL/min; No. of Passes: 6; Track Spacing: 2 mm; Pattern: HH; Pressure: 10 psi; Gas Low Rate: 2 L/min; Nozzle Height: 40 mm; Drying Time: 10s.

Embedded spinal cord tissue sections: DAN (10 mg/mL) in ACN with 60 mM HCl/water 6:4 (*v/v*) was used. The spraying parameters were similar to those for spheroid samples.

Photo-cleavable mass tags: α-CHCA (10 mg/mL) in ACN/water/TFA 7:3:0.01 (*v/v/v*) was used. Spraying parameters: Spray Nozzle Velocity: 1350 mm/min; Flow Rate: 0.1 mL/min; No. of Passes: 8; Pattern: HH; Pressure: 10 psi; Gas Low Rate: 2 L/min; Nozzle Height: 40 mm; Drying Time: 10s.

MALDI MSI data analysis

Volcano plot generation: Volcano plots for EAE mouse model were generated using in house written Python-code. First, .imzML files of individual regions for each biological sample were generated using SCiLS lab (root mean square normalized). In addition, a feature list was generated within SCiLS lab via the feature finding option (T-ReX^2 algorithm, rel. intensity threshold 0.3%) and then exported as .csv file. Subsequently, statistical analysis was performed using the *scipy.stats* package. Hereby, p-values adjusted according to the Benjamini-Hochberg criterion and values for the $\log_2$ fold change were calculated for each *m/z* feature and visualized. For the case study of the EAE mouse model, the calculation was performed for n=3 biological replicates (three EAE mice and three control mice). For the case study of the ARSA mouse model, the calculation was performed for n=2 biological replicates (two ARSA-/- and two ARSA+/+ mice, both 60 weeks old) combined into one dataset. The volcano plot was generated according to Cairns *et al.*[3], using a subset of 3000 pixels, repeated ten times.

LC-TIMS-MS data analysis

Data processing including identification and annotation of the sulfatides was performed using Metaboscape 2021b (Bruker Daltonics). The acquired data from ARSA-/- and ARSA+/+ (ARSA +/- for 12-week-old mice) samples was subsequently uploaded to Metaboscape 2021b in a single bucket table. The processing method parameters included: i) feature detection was performed using an intensity threshold of 100 counts, ii) recursive feature extraction was conducted for features found in at least 1 out of 8 analyses (to ensure features are found in both or either ARSA-/- and ARSA+/+ (ARSA +/- for 12-week-mice) samples), and iii) the feature was only included in the bucket tables if it was found in 1 analysis after recursive feature extraction.

To establish a spectral library and sulfatide database with ion mobility values and retention time (RT), a reference kidney sample was first extracted and analyzed in the same manner as ARSA-/- and ARSA+/+ samples. Due to the lack of sufficient experimental data, including a spectral library for a broad coverage, each fragmentation spectrum was manually interpreted based on expected diagnostic fragment ions for sulfate and glycosidic bond cleavages from this glycolipid class.

To comprehensively cover possible sulfatide precursors in the MS survey and subsequent interrogation and assignment of their fragmentation spectra, the following approaches were utilized: a) Δ*m/z* of possible structural differences in ceramide, such as double bond, chain extension, hydroxylation, and multiple sphingoid bases; and b) Δ*m/z* of glycan chain differences due to monosaccharide units, N-acetyl substitution, and sulfate substitution of monosaccharides. These specific Δ*m/z* values were used to discover and cover both the ceramide heterogeneity and the glycan heterogeneity, as well as to infer ΔRT specific to homologous series. The Kendrick Mass Defect Plot available in Metaboscape was used as a quality control measure in this regard. Accordingly, the MS1 spectra, *m/z* of the precursors, RT, and their MS2 spectra were first manually assigned and curated. Subsequently, the CCS values for these structures were determined and included in the in-house database library. Following this manual annotation of sulfatides, the identified sulfatide species along with their 4D-descriptors (*m/z*, RT, CCS, and MS^2 spectra) were used to establish the Sulfatide Analyte List. This Sulfatide Analyte List was then used for

the automated annotation of sulfatides in all the samples. Tolerances and scoring adjustments for automatically annotating sulfatides using Sulfatides List include m/z between 2 and 5 mDa, RT between 0.1 and 0.5 min, mSigma between 10 and 500, MS/MS score between 500 and 900, and CCS between 0.1% and 2.0%. After manual verification and further curation, we identified 93–95 sulfatide species in two 60-week-old and 44-65 sulfatide species in two 12-week-old ARSA-/- kidney samples.

Supplementary References

1 Rittel, M. F. *et al.* Spatial Omics Imaging of Fresh-Frozen Tissue and Routine FFPE Histopathology of a Single Cancer Needle Core Biopsy: A Freezing Device and Multimodal Workflow. *Cancers (Basel)* **15**, doi:10.3390/cancers15102676 (2023).

2 Dannhorn, A. *et al.* Universal Sample Preparation Unlocking Multimodal Molecular Tissue Imaging. *Anal Chem* **92**, 11080-11088, doi:10.1021/acs.analchem.0c00826 (2020).

3 Cairns, J. L. *et al.* Mass-Guided Single-Cell MALDI Imaging of Low-Mass Metabolites Reveals Cellular Activation Markers. *Adv Sci (Weinh)*, e2410506, doi:10.1002/advs.202410506 (2024).

4 Balluff, B., Hopf, C., Porta Siegel, T., Grabsch, H. I. & Heeren, R. M. A. Batch Effects in MALDI Mass Spectrometry Imaging. *J Am Soc Mass Spectrom* **32**, 628-635, doi:10.1021/jasms.0c00393 (2021).

5 Abu Sammour, D. *et al.* Spatial probabilistic mapping of metabolite ensembles in mass spectrometry imaging. *Nat Commun* **14**, 1823, doi:10.1038/s41467-023-37394-z (2023).

6 Grove, K. J. *et al.* Diabetic nephropathy induces alterations in the glomerular and tubule lipid profiles. *J Lipid Res* **55**, 1375-1385, doi:10.1194/jlr.M049189 (2014).

7 Birge, R. T. The Calculation of Errors by the Method of Least Squares. *Phys. Rev.* **40**, 207, doi:https://doi.org/10.1103/PhysRev.40.207 (1932).

8 Marsching, C., Eckhardt, M., Grone, H. J., Sandhoff, R. & Hopf, C. Imaging of complex sulfatides SM3 and SB1a in mouse kidney using MALDI-TOF/TOF mass spectrometry. *Anal Bioanal Chem* **401**, 53-64, doi:10.1007/s00216-011-4802-0 (2011).

9 Rosas, S. *et al.* Metasurface-Enhanced Mid-Infrared Spectrochemical Imaging of Tissues. *Adv Mater* **35**, e2301208, doi:10.1002/adma.202301208 (2023).

10 Dreissig, I., Machill, S., Salzer, R. & Krafft, C. Quantification of brain lipids by FTIR spectroscopy and partial least squares regression. *Spectrochim Acta A Mol Biomol Spectrosc* **71**, 2069-2075, doi:10.1016/j.saa.2008.08.008 (2009).

11 Dice, L. R. Measures of the Amount of Ecologic Association Between Species. *Ecology* **26**, 297-302, doi:https://doi.org/10.2307/1932409 (1945).

12 Hsu, F. F. & Turk, J. Electrospray ionization with low-energy collisionally activated dissociation tandem mass spectrometry of glycerophospholipids: mechanisms of fragmentation and structural characterization. *J Chromatogr B Analyt Technol Biomed Life Sci* **877**, 2673-2695, doi:10.1016/j.jchromb.2009.02.033 (2009).

13 Duez, Q. *et al.* One Step Further in the Characterization of Synthetic Polymers by Ion Mobility Mass Spectrometry: Evaluating the Contribution of End-groups. *Polymers (Basel)* **11**, doi:10.3390/polym11040688 (2019).
