# Supplementary figures and images for: Deep MALDI-MS spatial omics guided by quantum cascade laser mid-infrared imaging microscopy (part 2 of 2)
Source: Nat Commun. 2025 May 22;16:4759. doi: 10.1038/s41467-025-59839-3 (PMC12098849; doi:10.1038/s41467-025-59839-3)

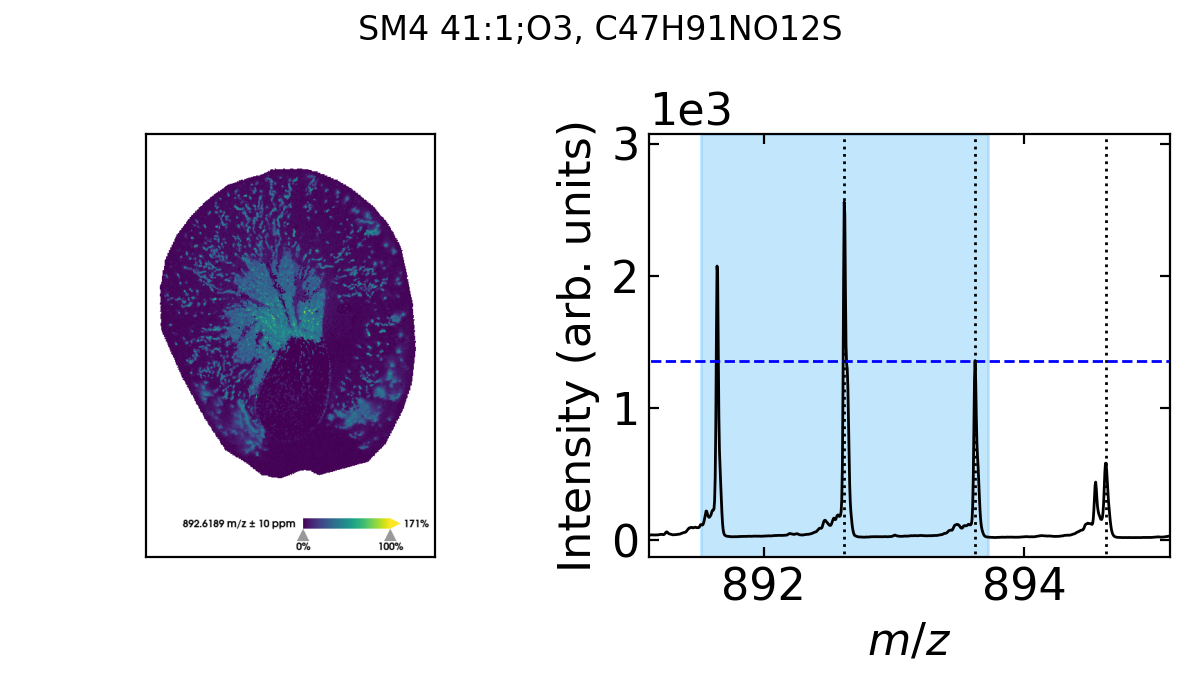

Supplement: Supplementary file 3 — Supplementary Data 1 [file 41467_2025_59839_MOESM3_ESM.zip › Suppl_Dataset_1_REV/qTOF_data1_slide1_python/892.618922_qTOF_60w_1.png]

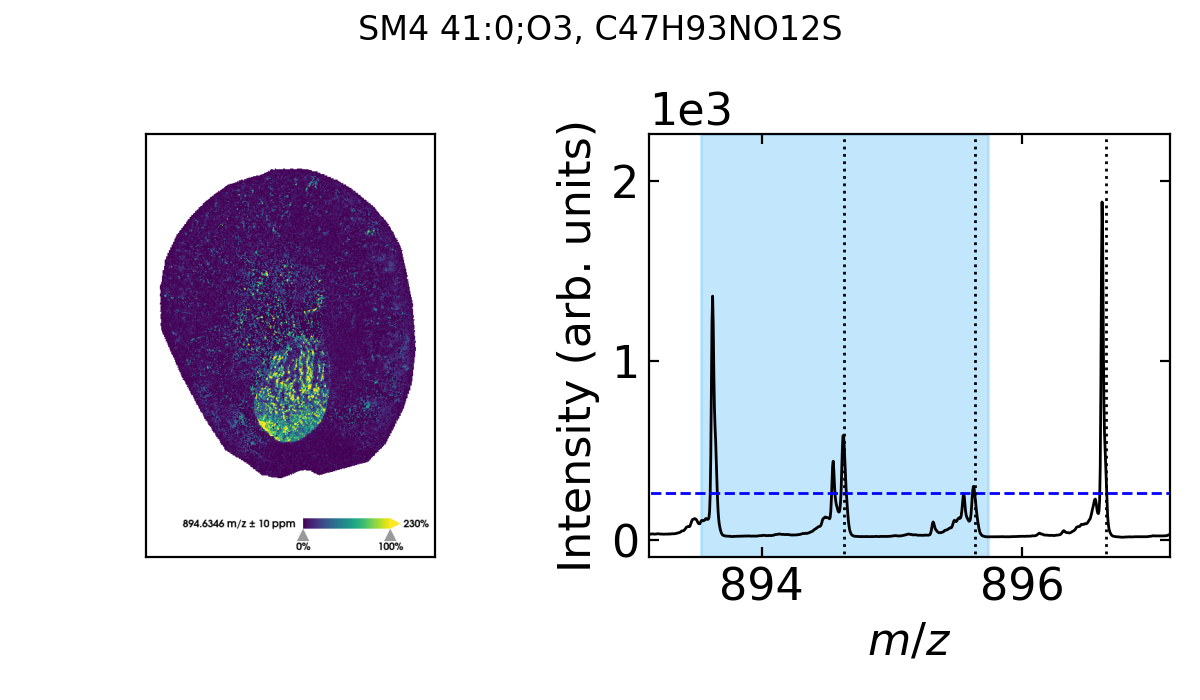

Supplement: Supplementary file 3 — Supplementary Data 1 [file 41467_2025_59839_MOESM3_ESM.zip › Suppl_Dataset_1_REV/qTOF_data1_slide1_python/894.634572_qTOF_60w_1.png]

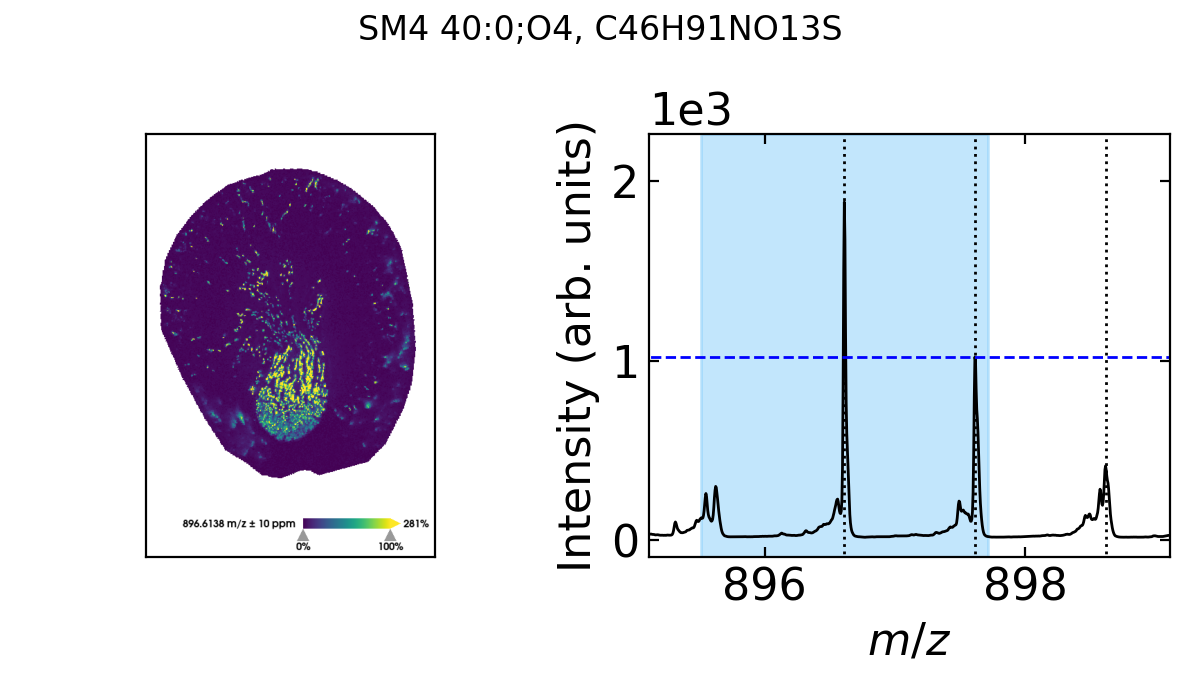

Supplement: Supplementary file 3 — Supplementary Data 1 [file 41467_2025_59839_MOESM3_ESM.zip › Suppl_Dataset_1_REV/qTOF_data1_slide1_python/896.613837_qTOF_60w_1.png]

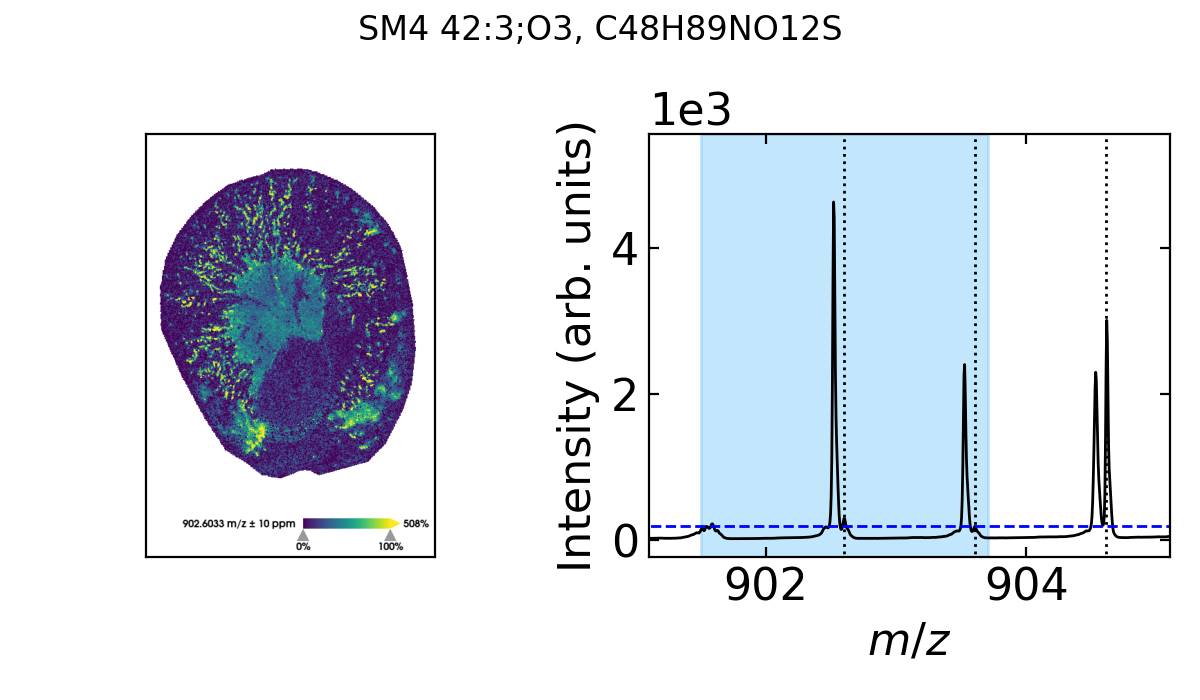

Supplement: Supplementary file 3 — Supplementary Data 1 [file 41467_2025_59839_MOESM3_ESM.zip › Suppl_Dataset_1_REV/qTOF_data1_slide1_python/902.603272_qTOF_60w_1.png]

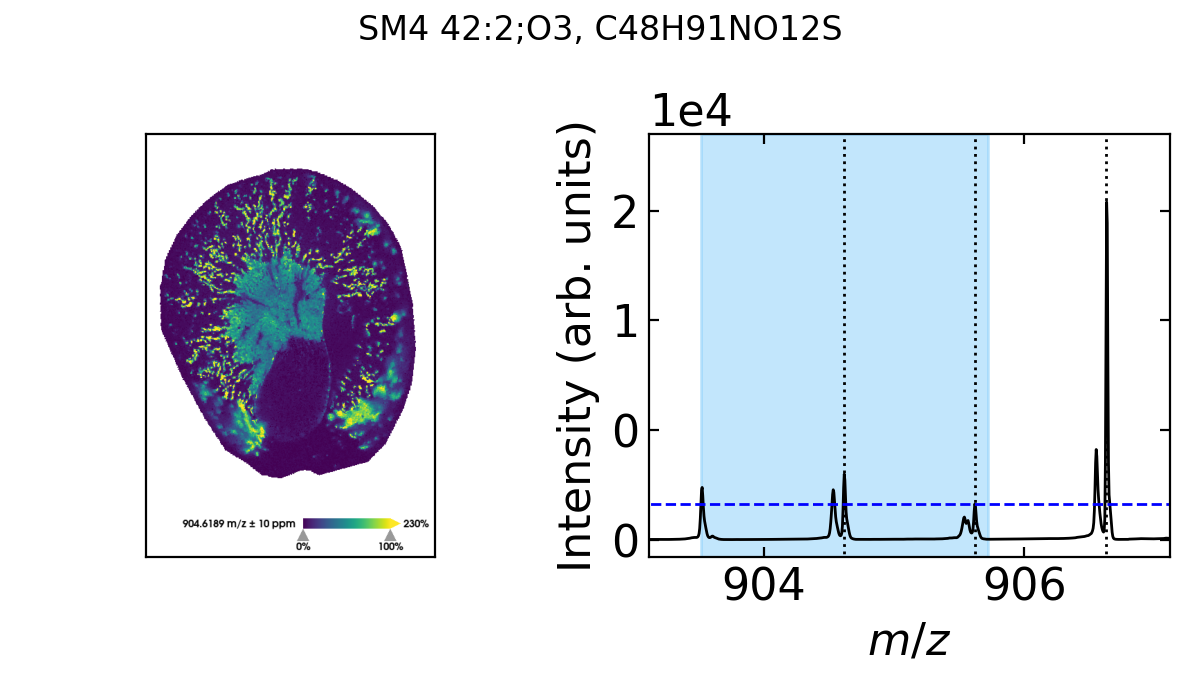

Supplement: Supplementary file 3 — Supplementary Data 1 [file 41467_2025_59839_MOESM3_ESM.zip › Suppl_Dataset_1_REV/qTOF_data1_slide1_python/904.618922_qTOF_60w_1.png]

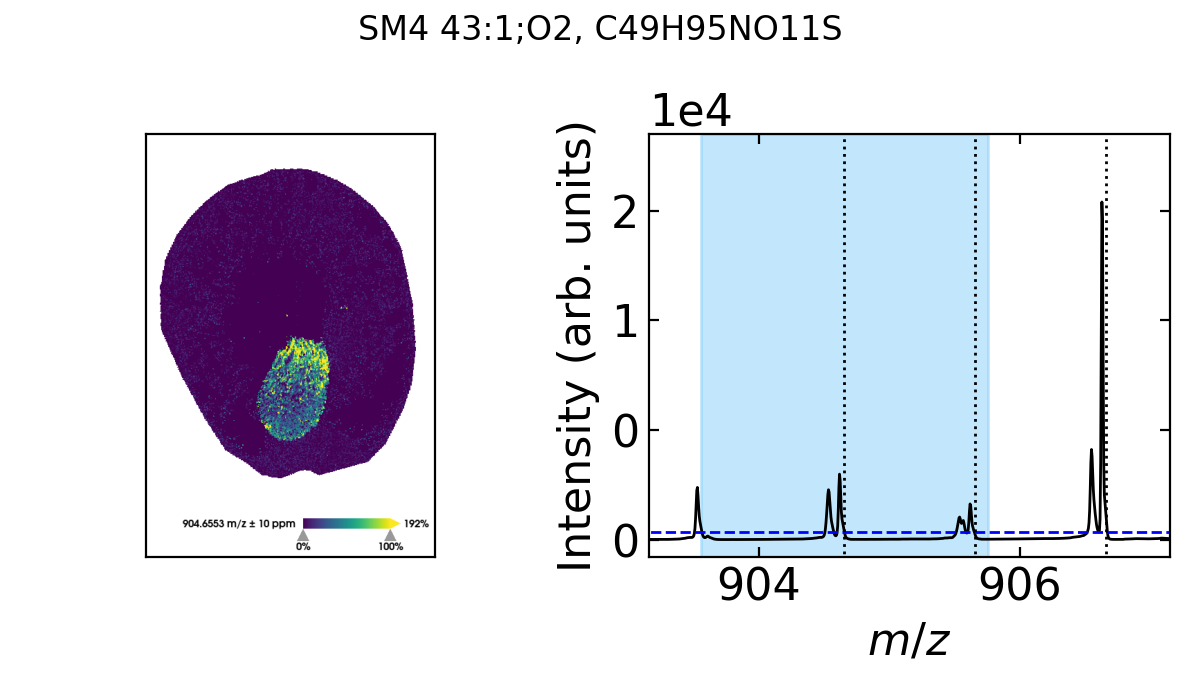

Supplement: Supplementary file 3 — Supplementary Data 1 [file 41467_2025_59839_MOESM3_ESM.zip › Suppl_Dataset_1_REV/qTOF_data1_slide1_python/904.655307_qTOF_60w_1.png]

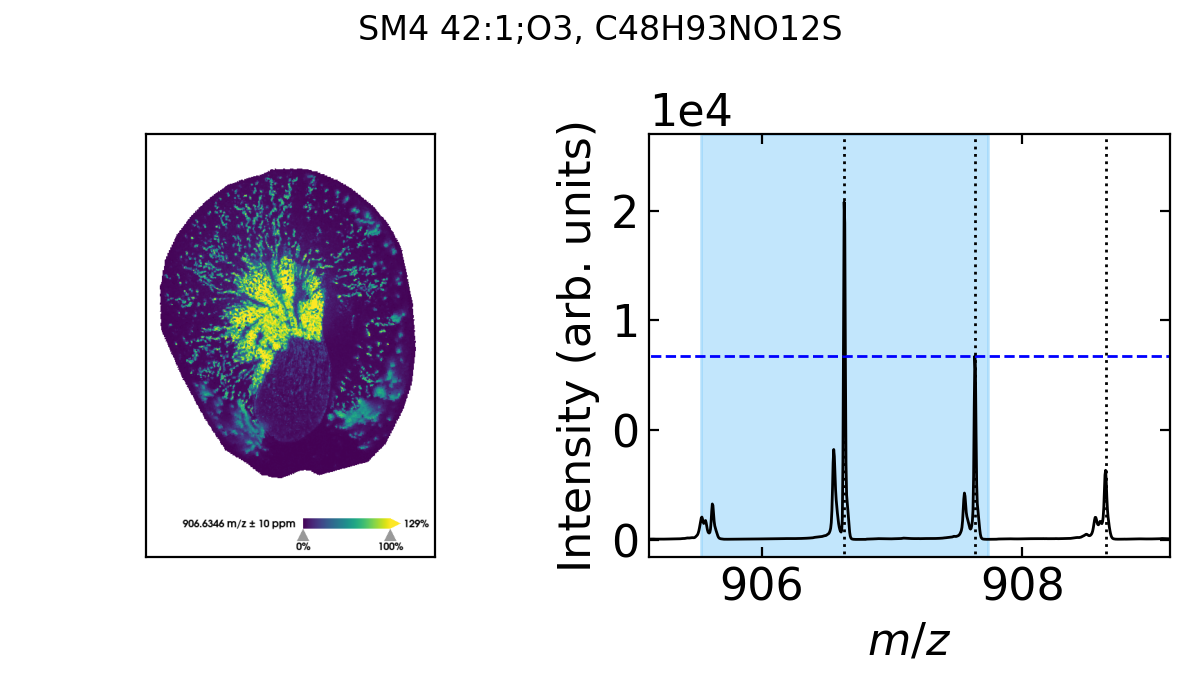

Supplement: Supplementary file 3 — Supplementary Data 1 [file 41467_2025_59839_MOESM3_ESM.zip › Suppl_Dataset_1_REV/qTOF_data1_slide1_python/906.634572_qTOF_60w_1.png]

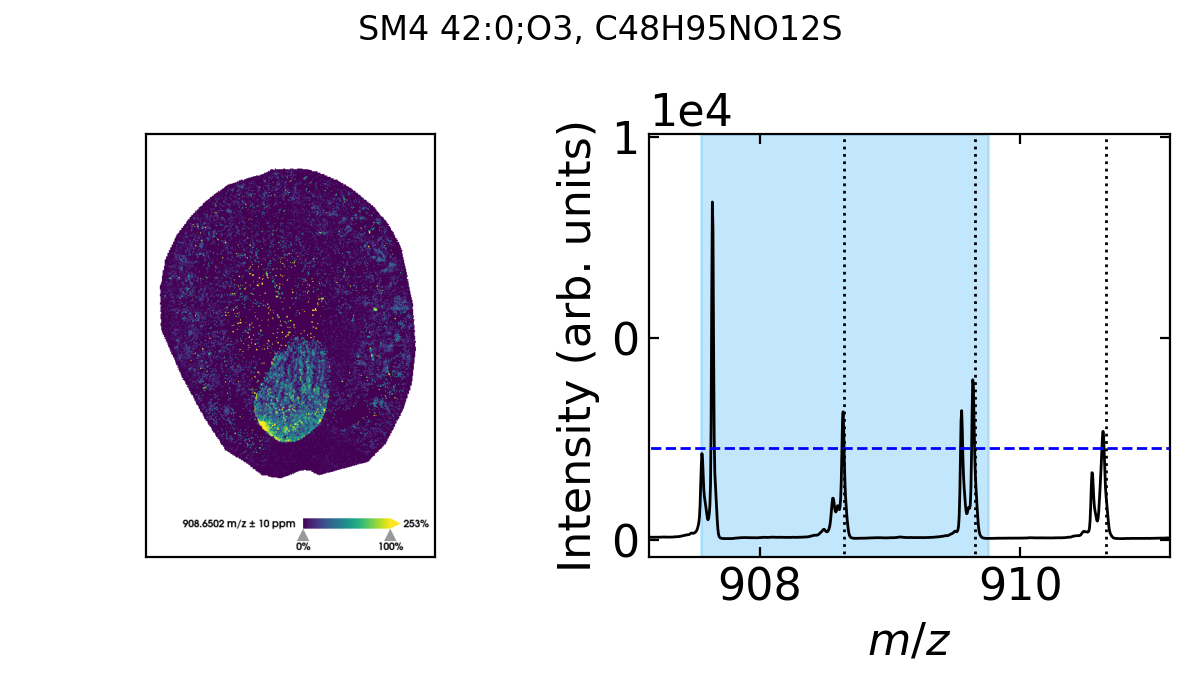

Supplement: Supplementary file 3 — Supplementary Data 1 [file 41467_2025_59839_MOESM3_ESM.zip › Suppl_Dataset_1_REV/qTOF_data1_slide1_python/908.650222_qTOF_60w_1.png]

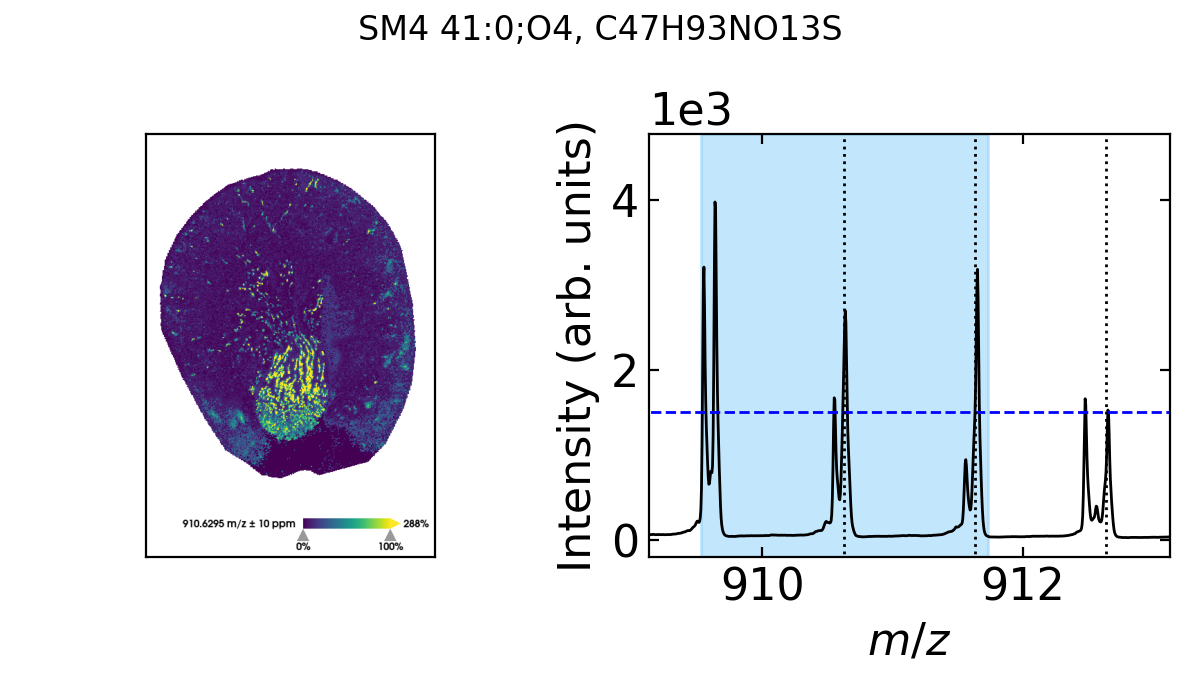

Supplement: Supplementary file 3 — Supplementary Data 1 [file 41467_2025_59839_MOESM3_ESM.zip › Suppl_Dataset_1_REV/qTOF_data1_slide1_python/910.629487_qTOF_60w_1.png]

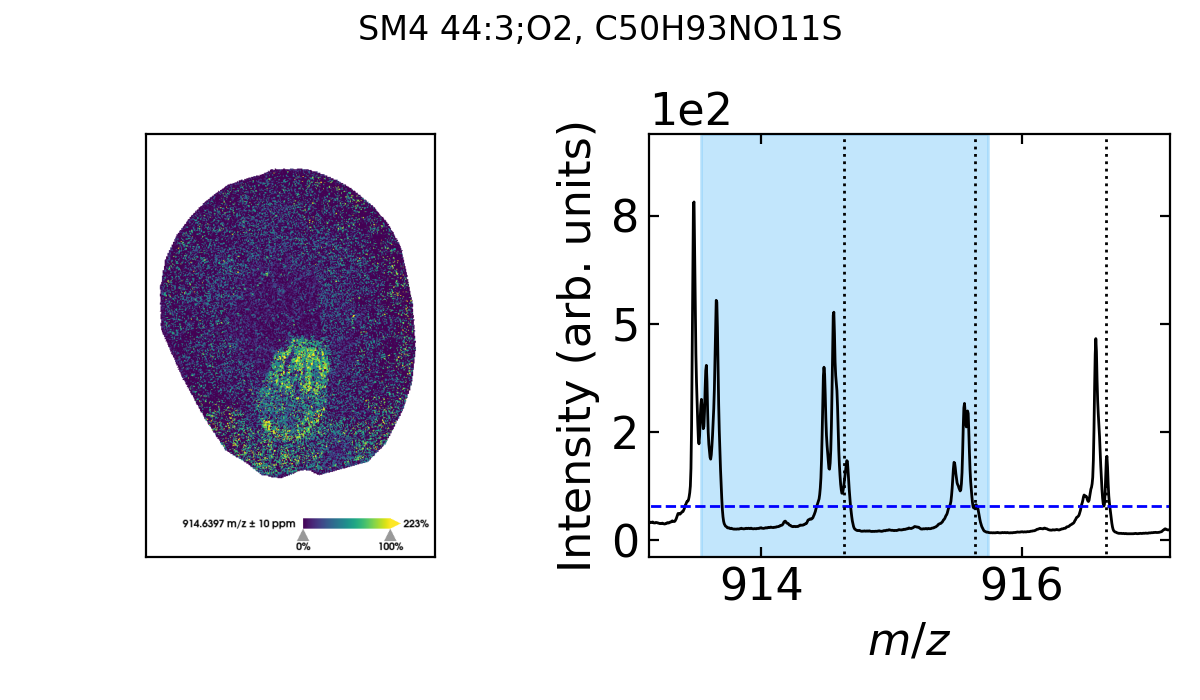

Supplement: Supplementary file 3 — Supplementary Data 1 [file 41467_2025_59839_MOESM3_ESM.zip › Suppl_Dataset_1_REV/qTOF_data1_slide1_python/914.639657_qTOF_60w_1.png]

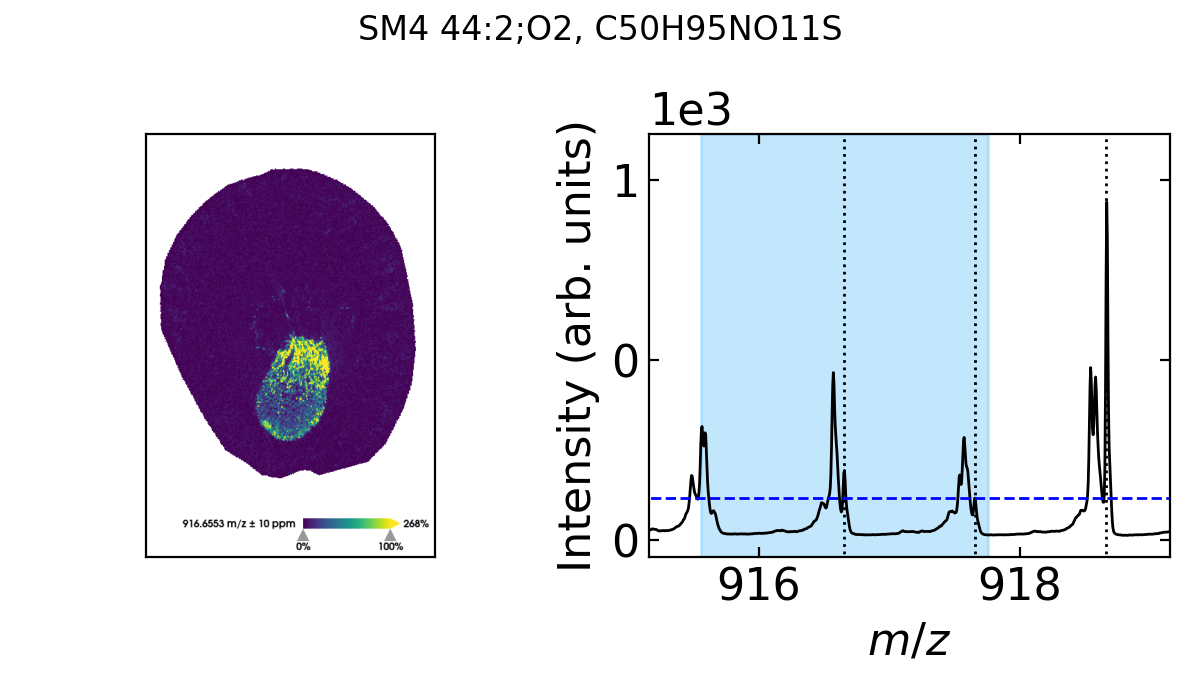

Supplement: Supplementary file 3 — Supplementary Data 1 [file 41467_2025_59839_MOESM3_ESM.zip › Suppl_Dataset_1_REV/qTOF_data1_slide1_python/916.655307_qTOF_60w_1.png]

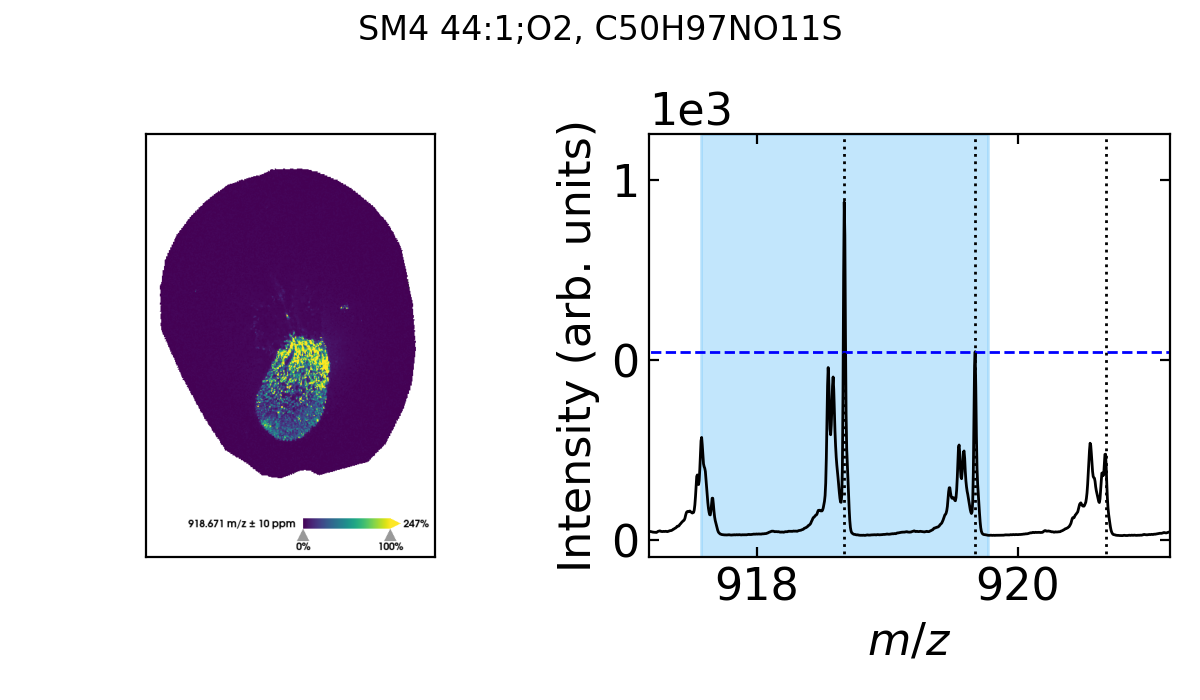

Supplement: Supplementary file 3 — Supplementary Data 1 [file 41467_2025_59839_MOESM3_ESM.zip › Suppl_Dataset_1_REV/qTOF_data1_slide1_python/918.670957_qTOF_60w_1.png]

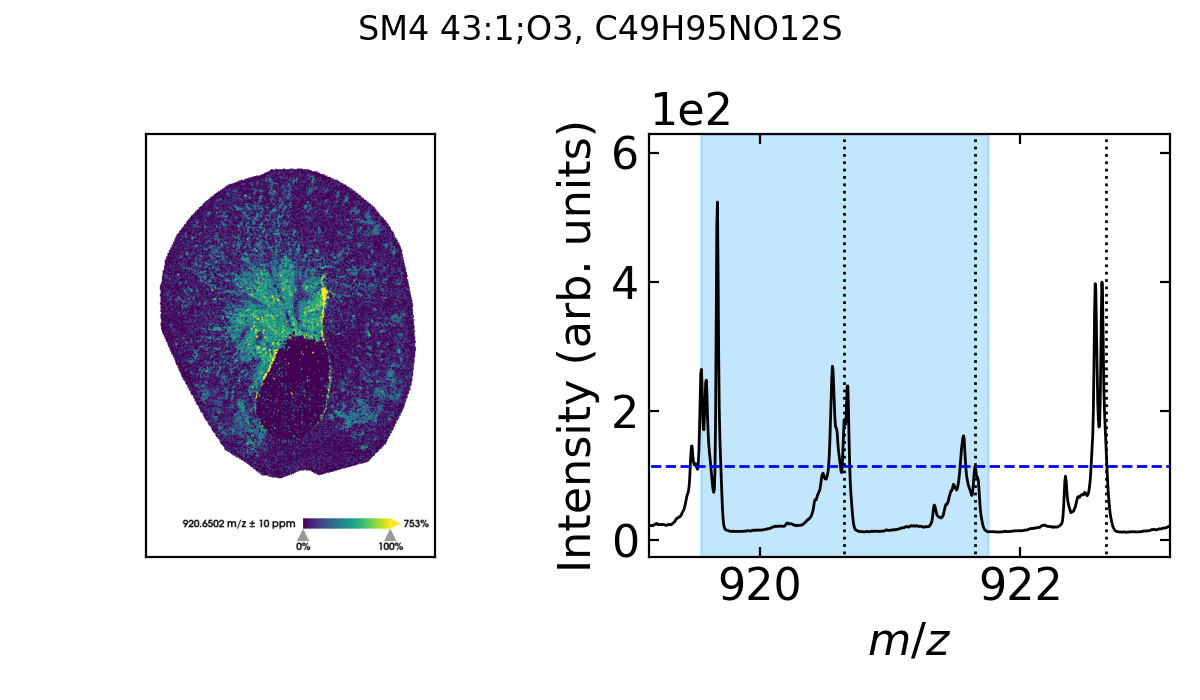

Supplement: Supplementary file 3 — Supplementary Data 1 [file 41467_2025_59839_MOESM3_ESM.zip › Suppl_Dataset_1_REV/qTOF_data1_slide1_python/920.650222_qTOF_60w_1.png]

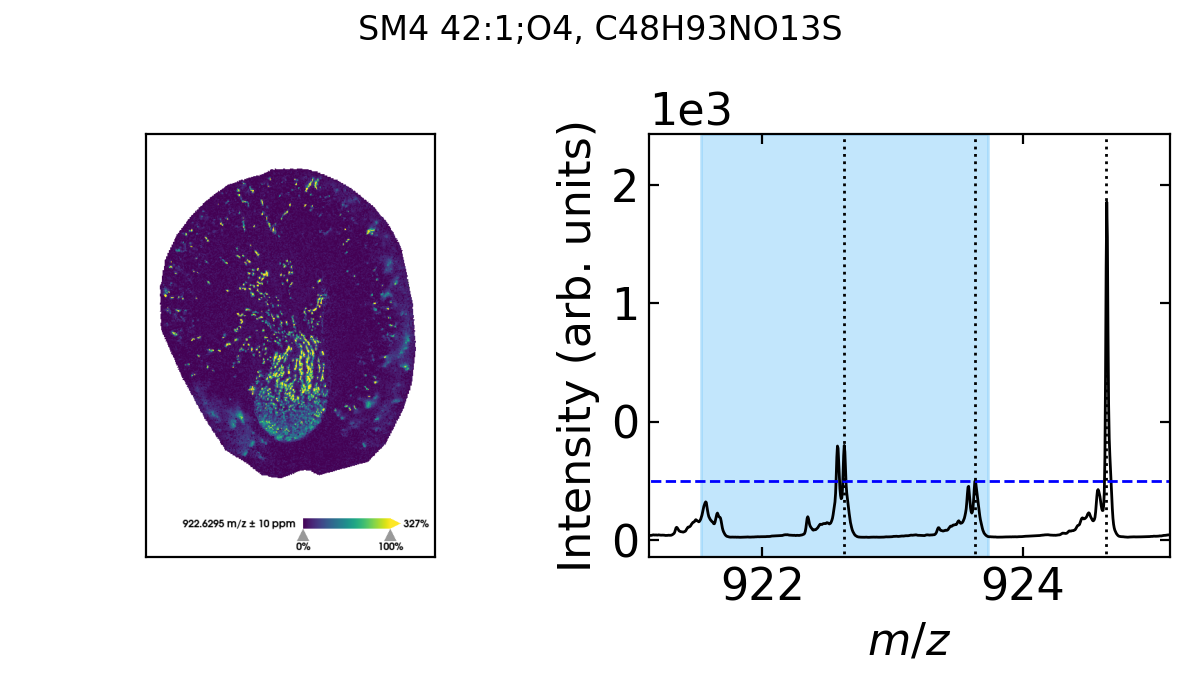

Supplement: Supplementary file 3 — Supplementary Data 1 [file 41467_2025_59839_MOESM3_ESM.zip › Suppl_Dataset_1_REV/qTOF_data1_slide1_python/922.629487_qTOF_60w_1.png]

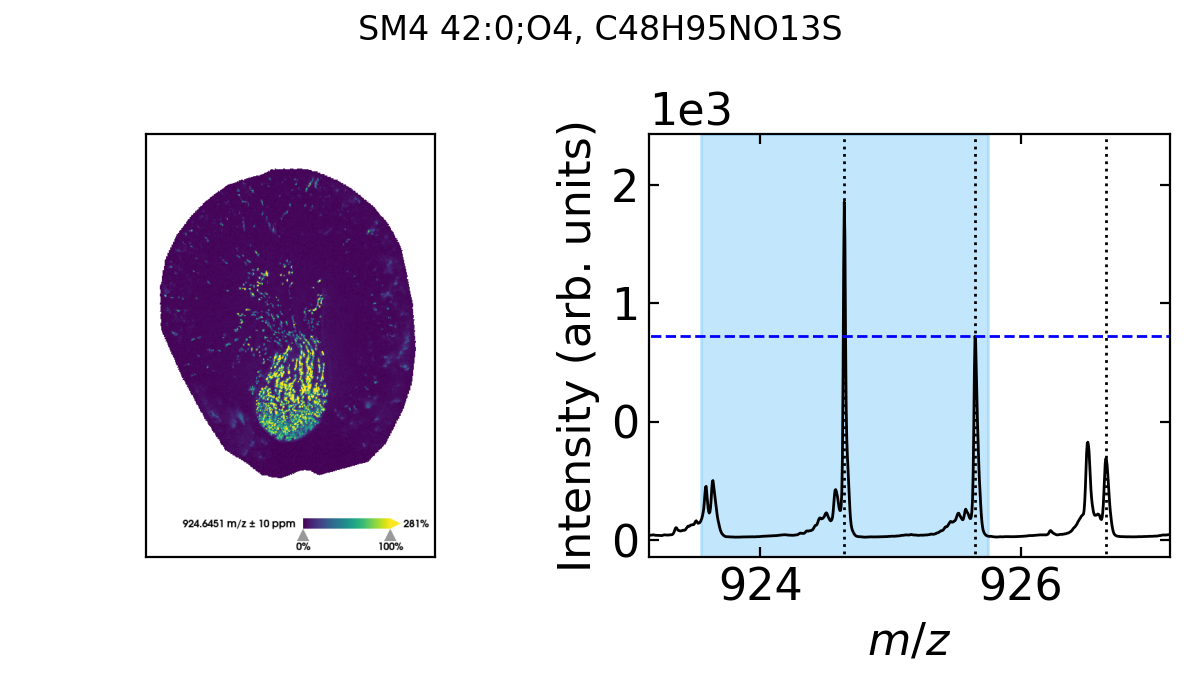

Supplement: Supplementary file 3 — Supplementary Data 1 [file 41467_2025_59839_MOESM3_ESM.zip › Suppl_Dataset_1_REV/qTOF_data1_slide1_python/924.645137_qTOF_60w_1.png]

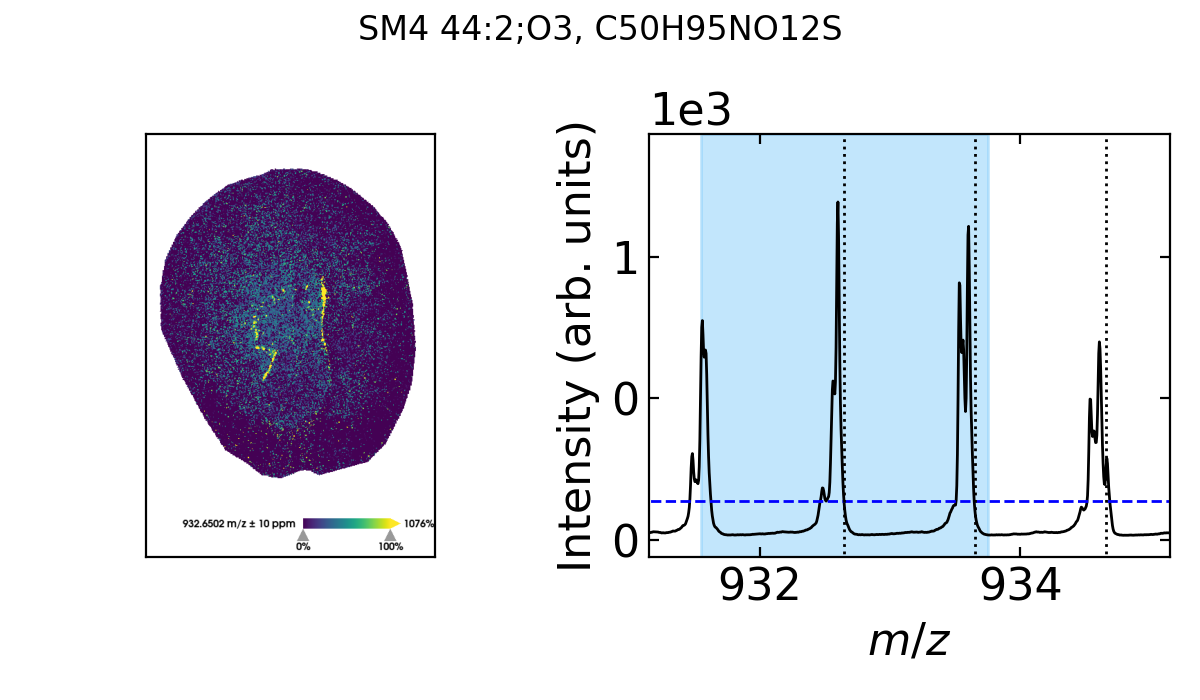

Supplement: Supplementary file 3 — Supplementary Data 1 [file 41467_2025_59839_MOESM3_ESM.zip › Suppl_Dataset_1_REV/qTOF_data1_slide1_python/932.650222_qTOF_60w_1.png]

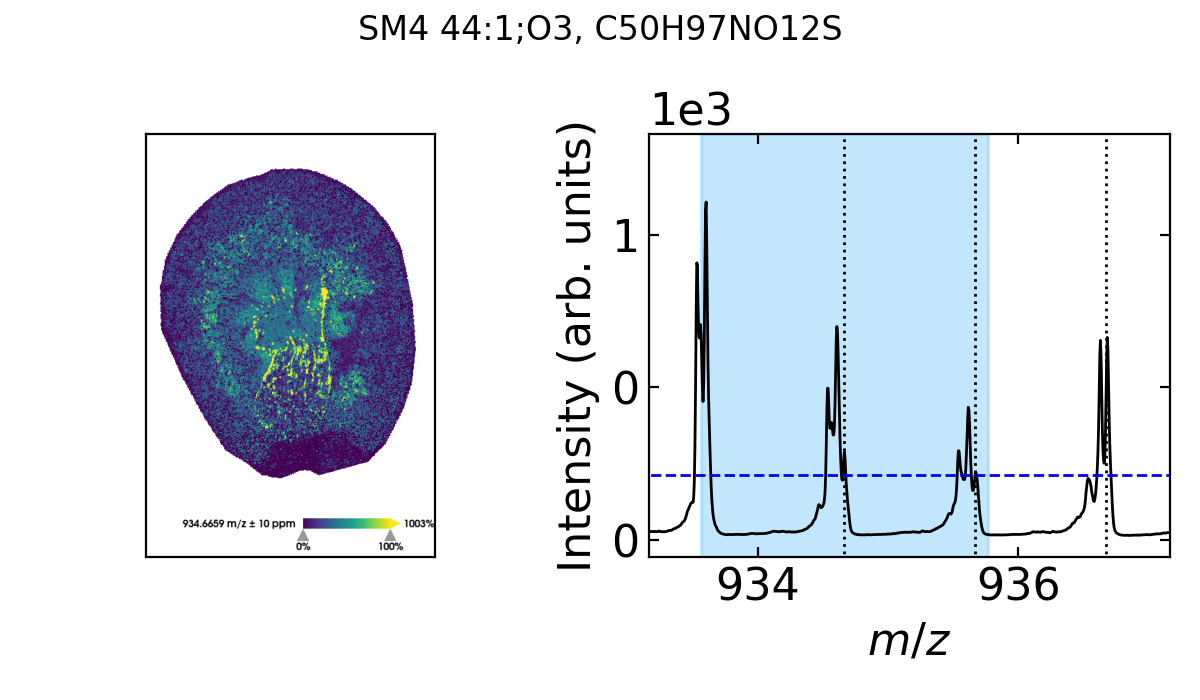

Supplement: Supplementary file 3 — Supplementary Data 1 [file 41467_2025_59839_MOESM3_ESM.zip › Suppl_Dataset_1_REV/qTOF_data1_slide1_python/934.665872_qTOF_60w_1.png]

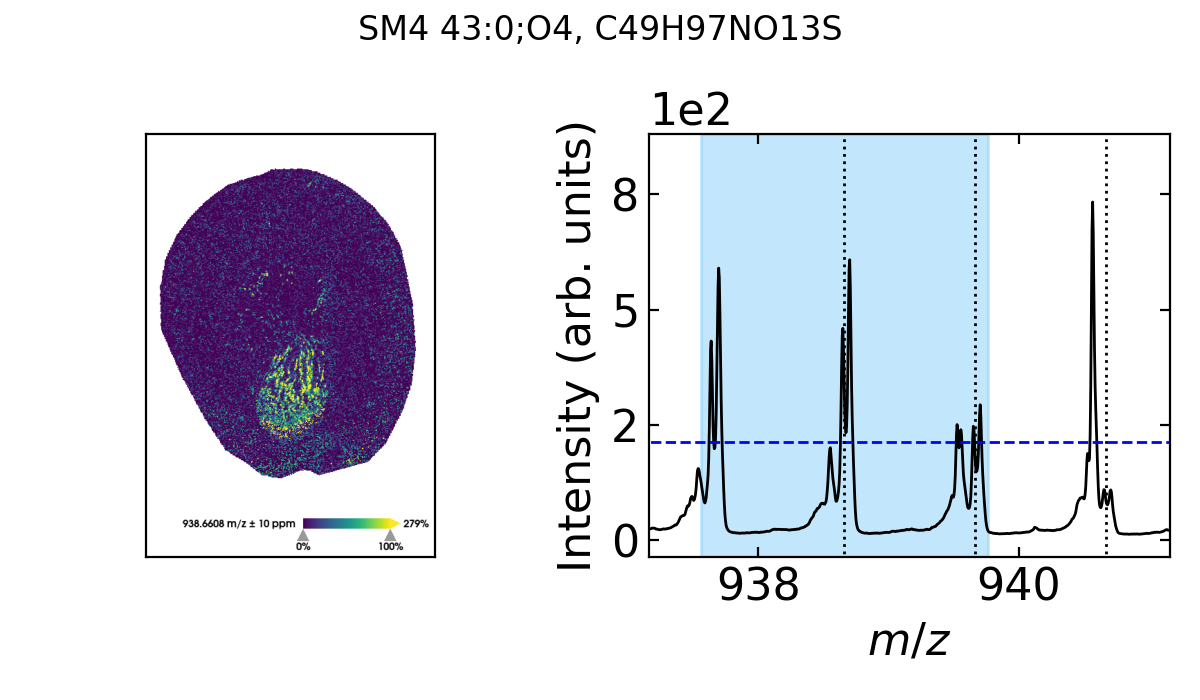

Supplement: Supplementary file 3 — Supplementary Data 1 [file 41467_2025_59839_MOESM3_ESM.zip › Suppl_Dataset_1_REV/qTOF_data1_slide1_python/938.660787_qTOF_60w_1.png]

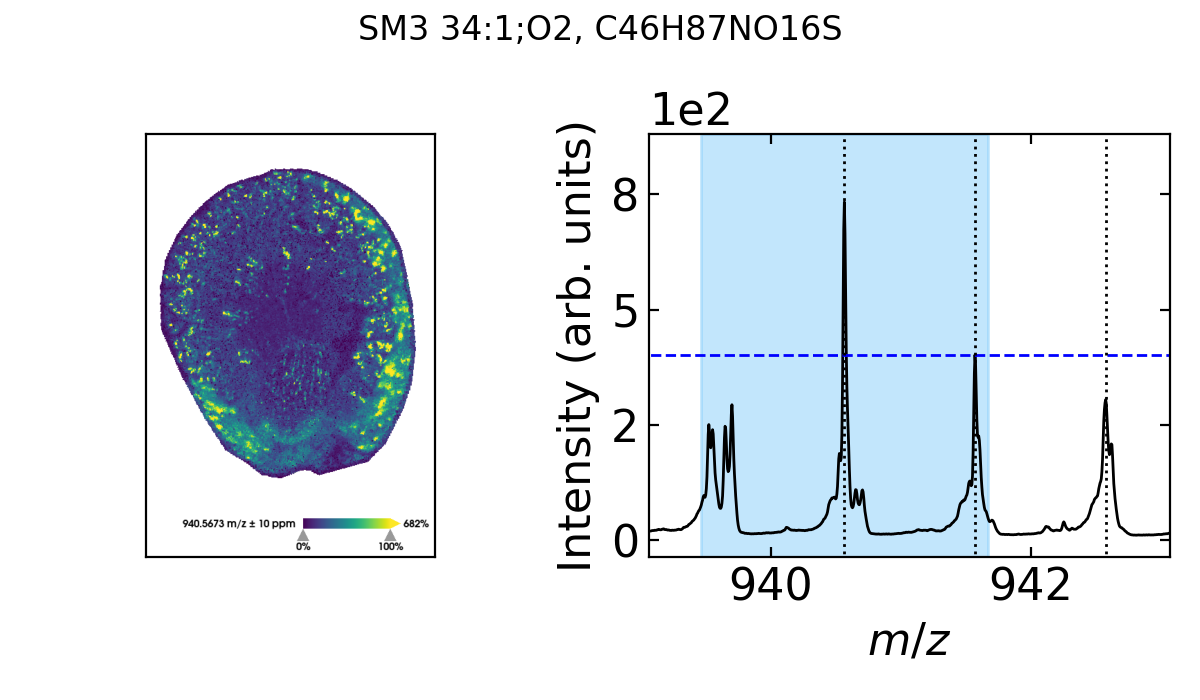

Supplement: Supplementary file 3 — Supplementary Data 1 [file 41467_2025_59839_MOESM3_ESM.zip › Suppl_Dataset_1_REV/qTOF_data1_slide1_python/940.567281_qTOF_60w_1.png]

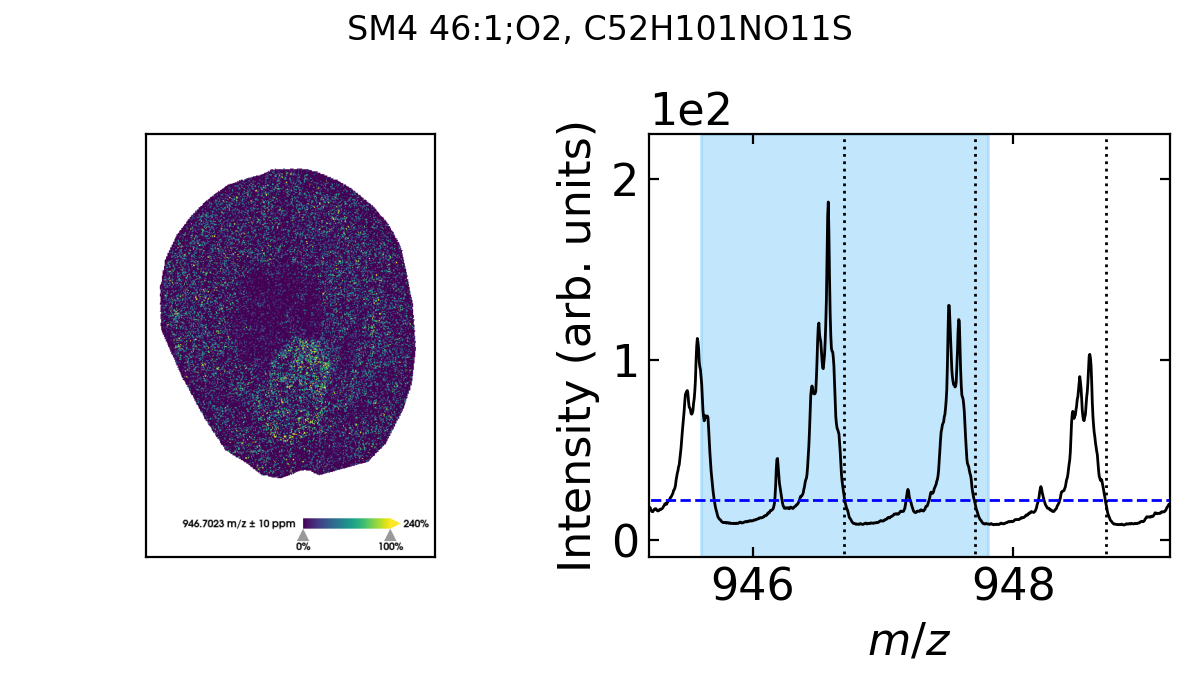

Supplement: Supplementary file 3 — Supplementary Data 1 [file 41467_2025_59839_MOESM3_ESM.zip › Suppl_Dataset_1_REV/qTOF_data1_slide1_python/946.702258_qTOF_60w_1.png]

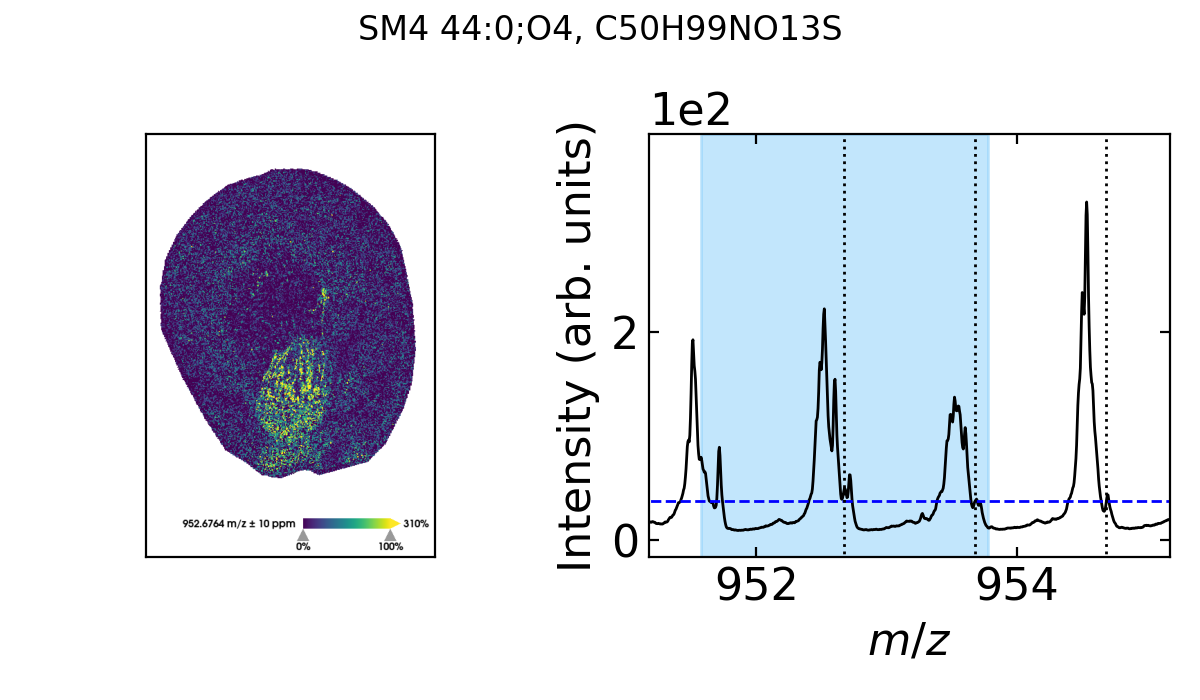

Supplement: Supplementary file 3 — Supplementary Data 1 [file 41467_2025_59839_MOESM3_ESM.zip › Suppl_Dataset_1_REV/qTOF_data1_slide1_python/952.676438_qTOF_60w_1.png]

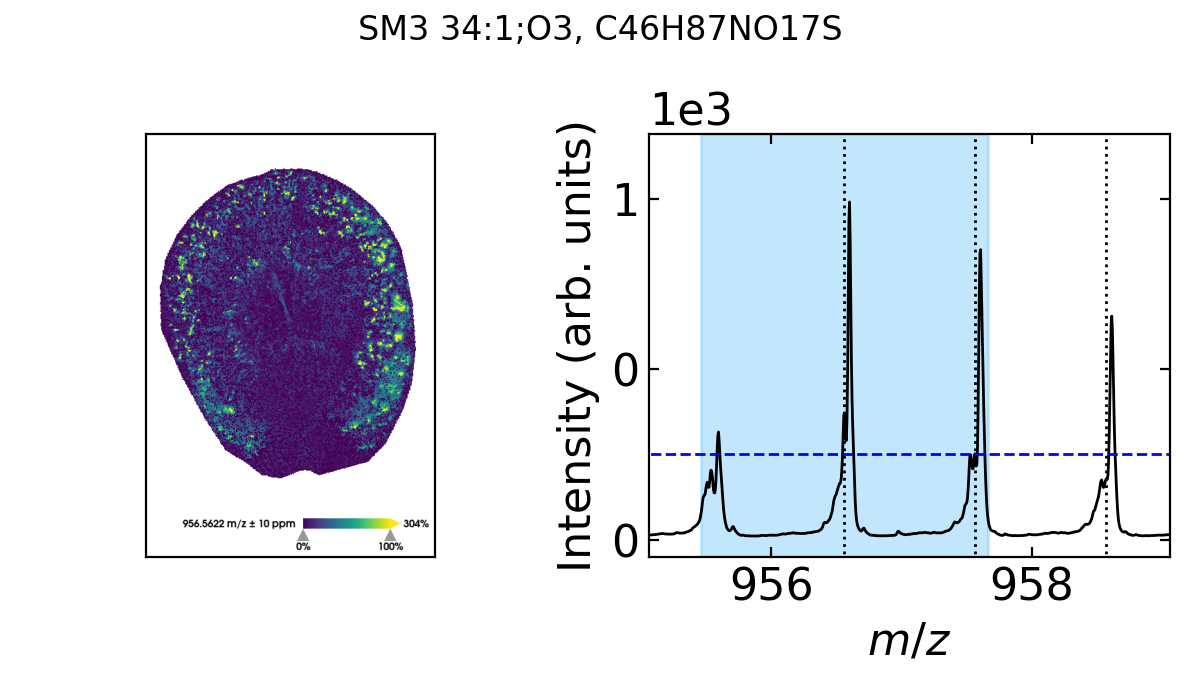

Supplement: Supplementary file 3 — Supplementary Data 1 [file 41467_2025_59839_MOESM3_ESM.zip › Suppl_Dataset_1_REV/qTOF_data1_slide1_python/956.562196_qTOF_60w_1.png]

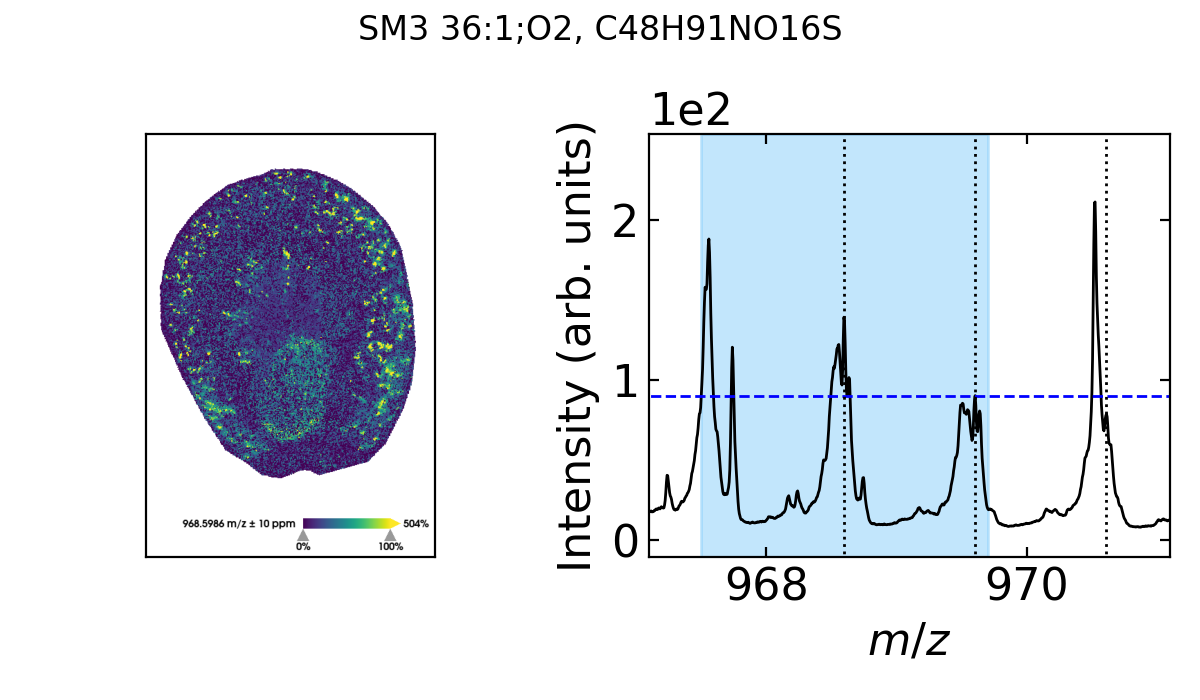

Supplement: Supplementary file 3 — Supplementary Data 1 [file 41467_2025_59839_MOESM3_ESM.zip › Suppl_Dataset_1_REV/qTOF_data1_slide1_python/968.598581_qTOF_60w_1.png]

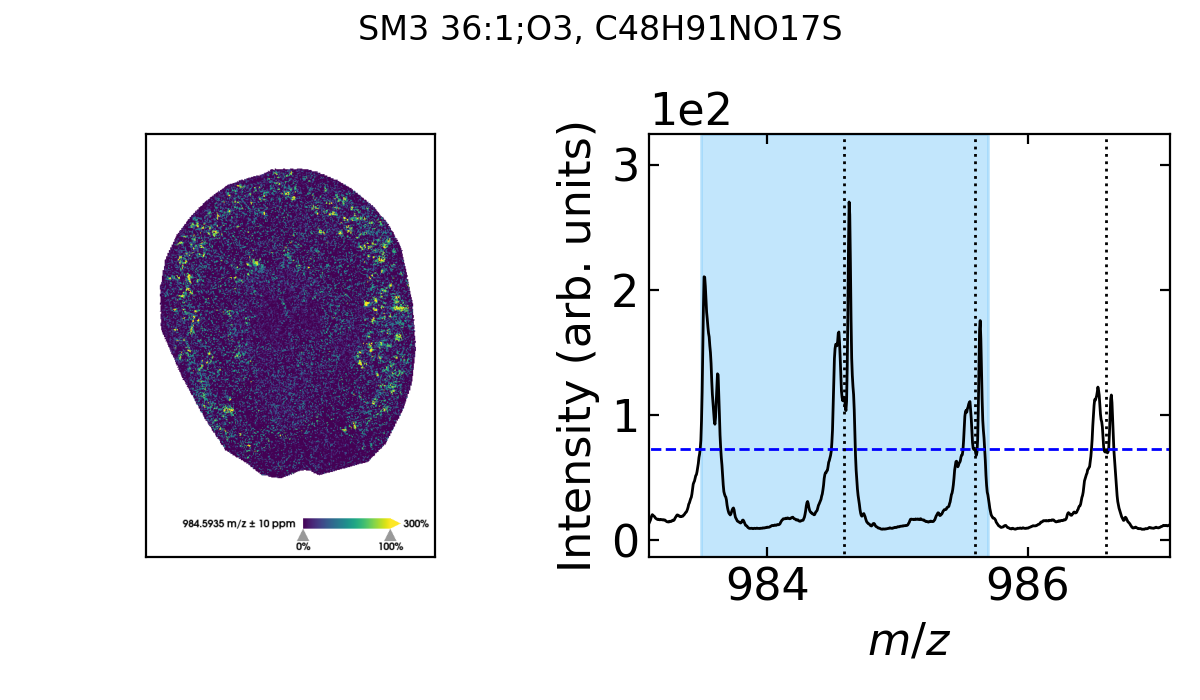

Supplement: Supplementary file 3 — Supplementary Data 1 [file 41467_2025_59839_MOESM3_ESM.zip › Suppl_Dataset_1_REV/qTOF_data1_slide1_python/984.593496_qTOF_60w_1.png]

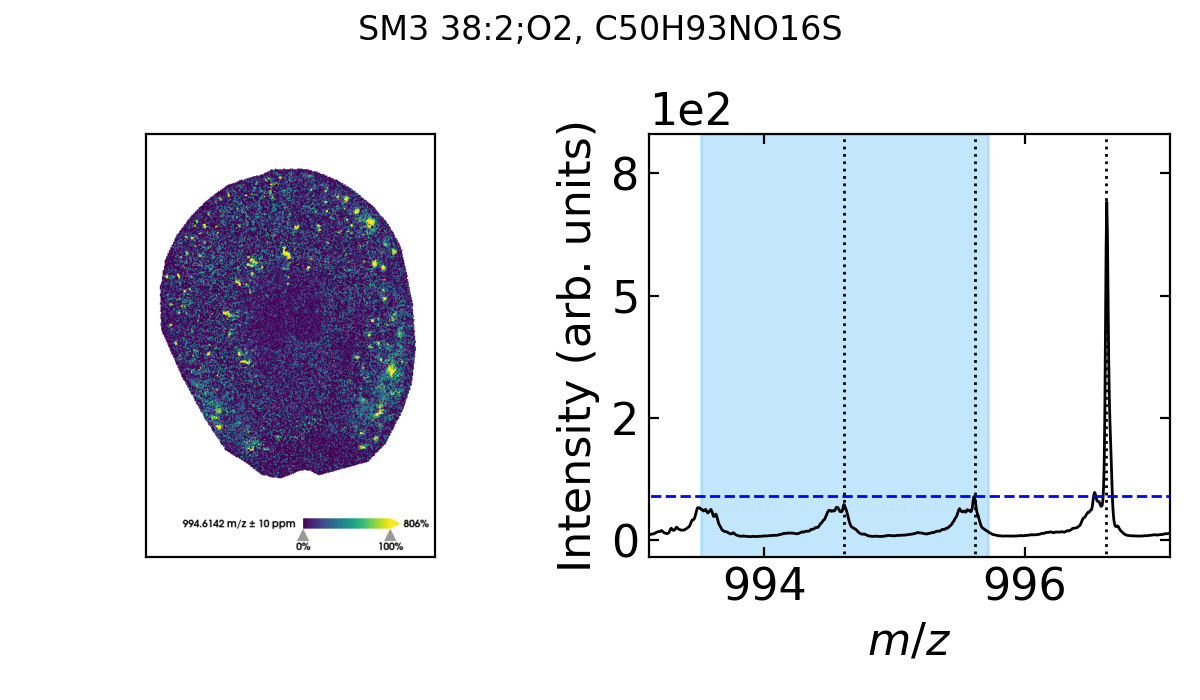

Supplement: Supplementary file 3 — Supplementary Data 1 [file 41467_2025_59839_MOESM3_ESM.zip › Suppl_Dataset_1_REV/qTOF_data1_slide1_python/994.614231_qTOF_60w_1.png]

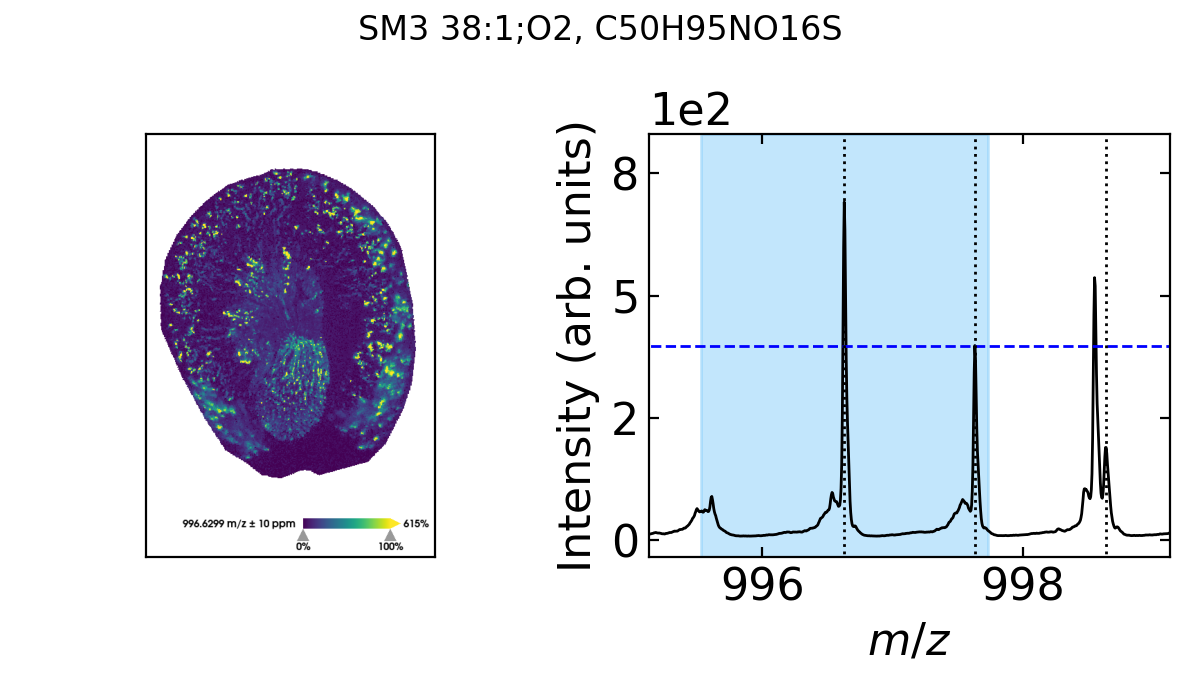

Supplement: Supplementary file 3 — Supplementary Data 1 [file 41467_2025_59839_MOESM3_ESM.zip › Suppl_Dataset_1_REV/qTOF_data1_slide1_python/996.629881_qTOF_60w_1.png]

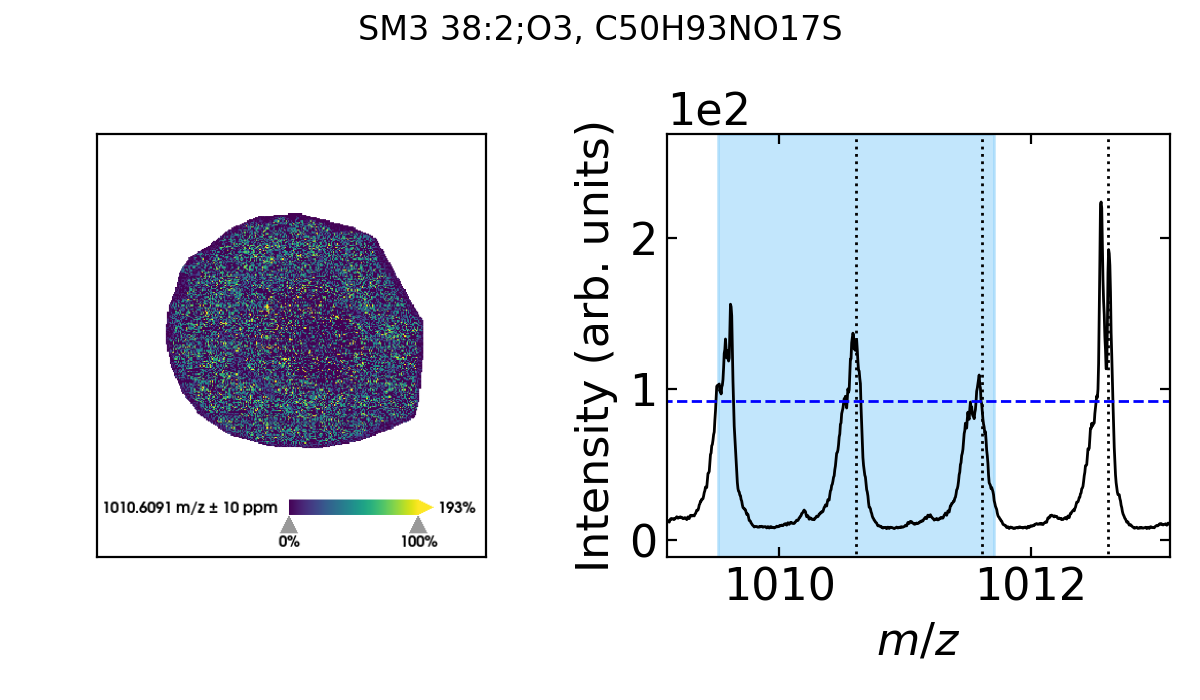

Supplement: Supplementary file 3 — Supplementary Data 1 [file 41467_2025_59839_MOESM3_ESM.zip › Suppl_Dataset_1_REV/qTOF_data3_slide1_python/1010.609146_qTOF_12w_1.png]

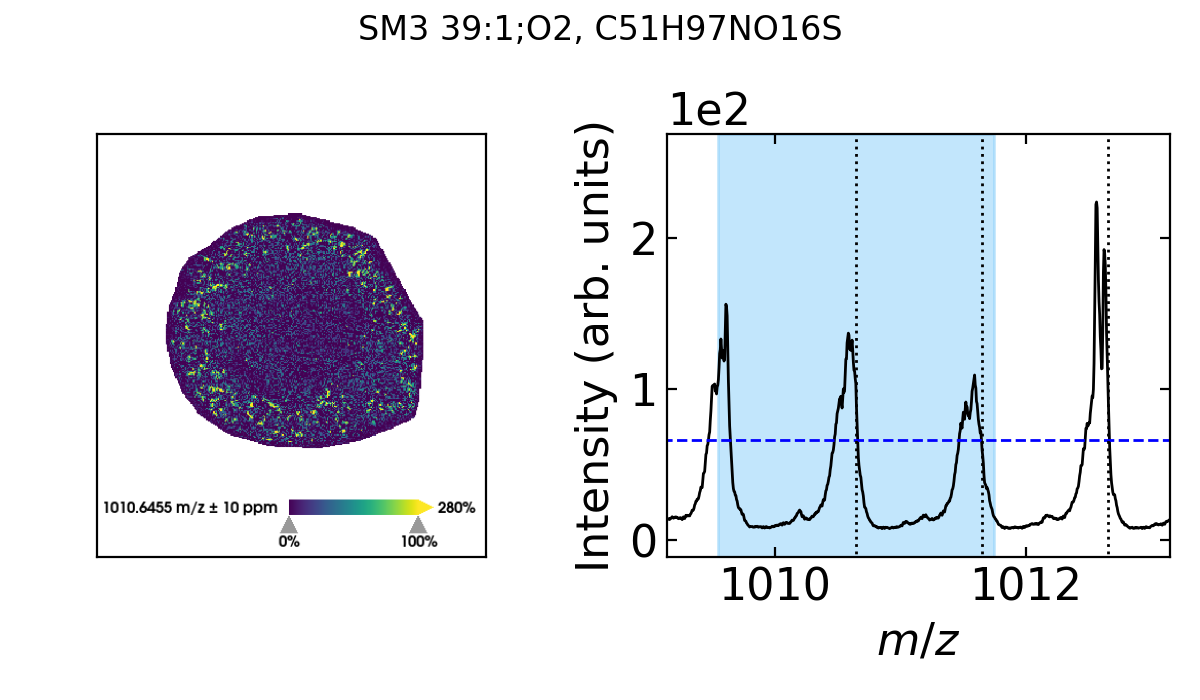

Supplement: Supplementary file 3 — Supplementary Data 1 [file 41467_2025_59839_MOESM3_ESM.zip › Suppl_Dataset_1_REV/qTOF_data3_slide1_python/1010.645531_qTOF_12w_1.png]

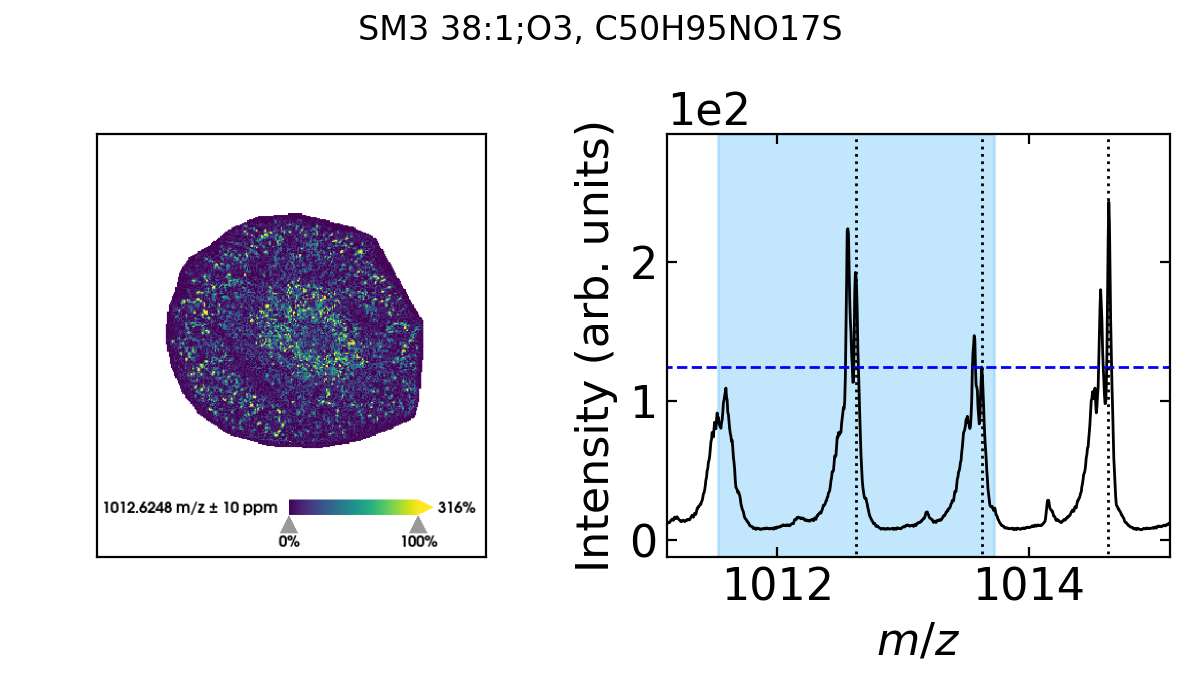

Supplement: Supplementary file 3 — Supplementary Data 1 [file 41467_2025_59839_MOESM3_ESM.zip › Suppl_Dataset_1_REV/qTOF_data3_slide1_python/1012.624796_qTOF_12w_1.png]

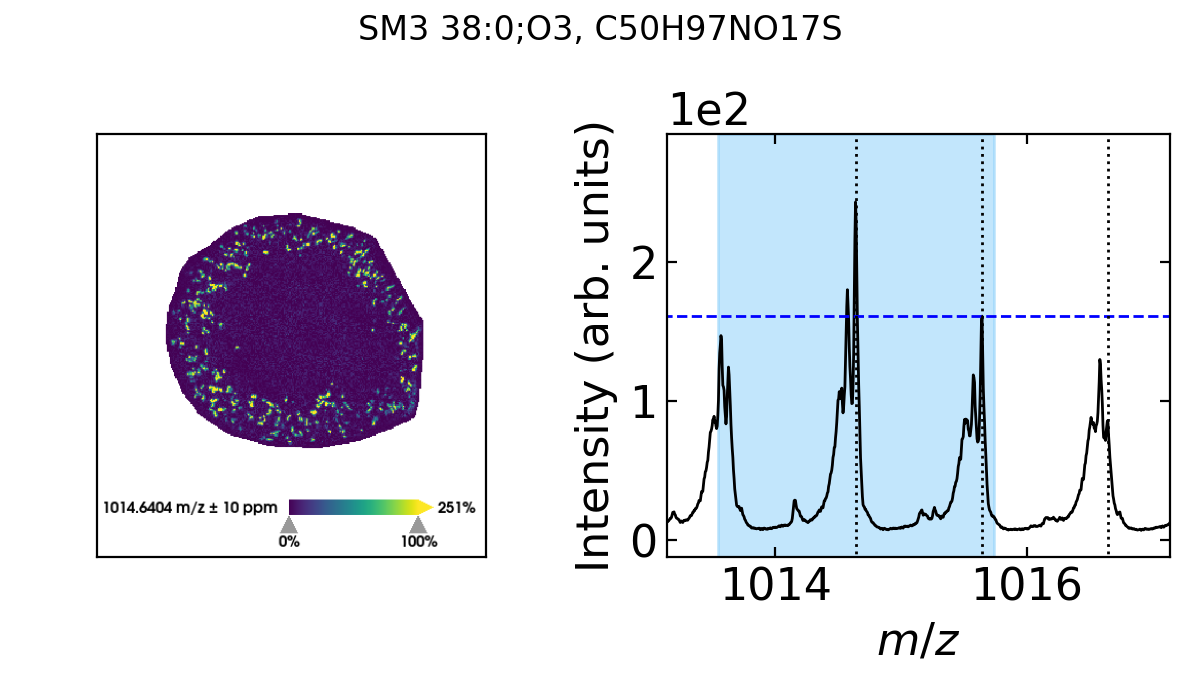

Supplement: Supplementary file 3 — Supplementary Data 1 [file 41467_2025_59839_MOESM3_ESM.zip › Suppl_Dataset_1_REV/qTOF_data3_slide1_python/1014.640446_qTOF_12w_1.png]

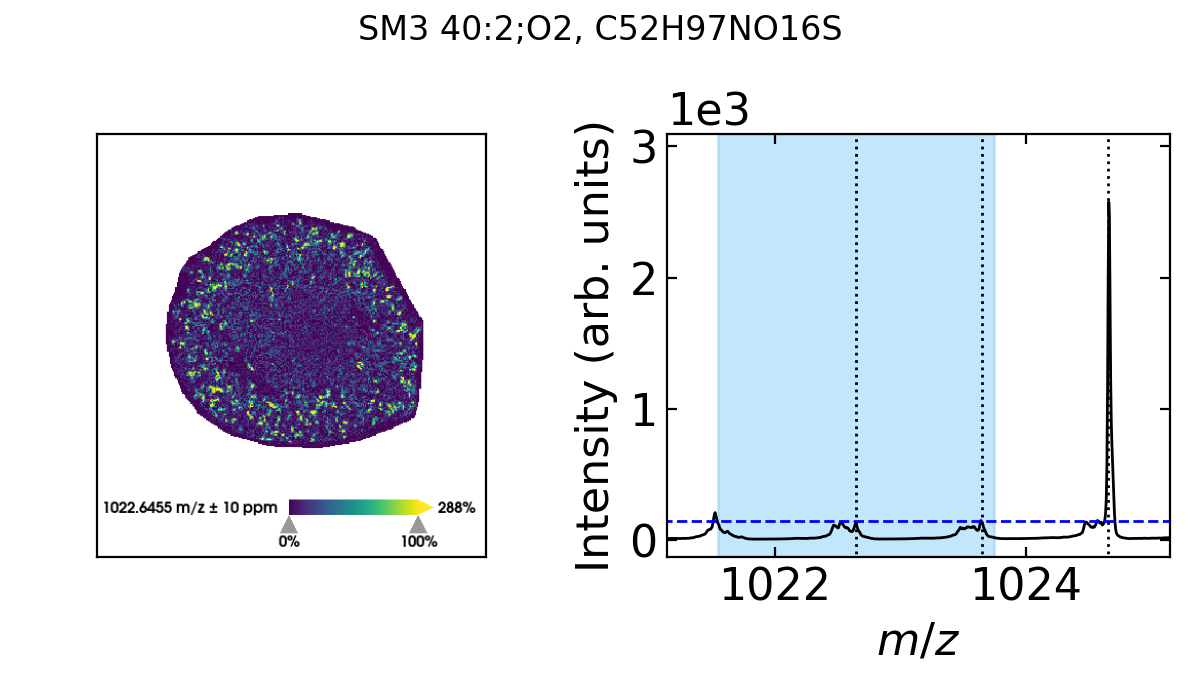

Supplement: Supplementary file 3 — Supplementary Data 1 [file 41467_2025_59839_MOESM3_ESM.zip › Suppl_Dataset_1_REV/qTOF_data3_slide1_python/1022.645531_qTOF_12w_1.png]

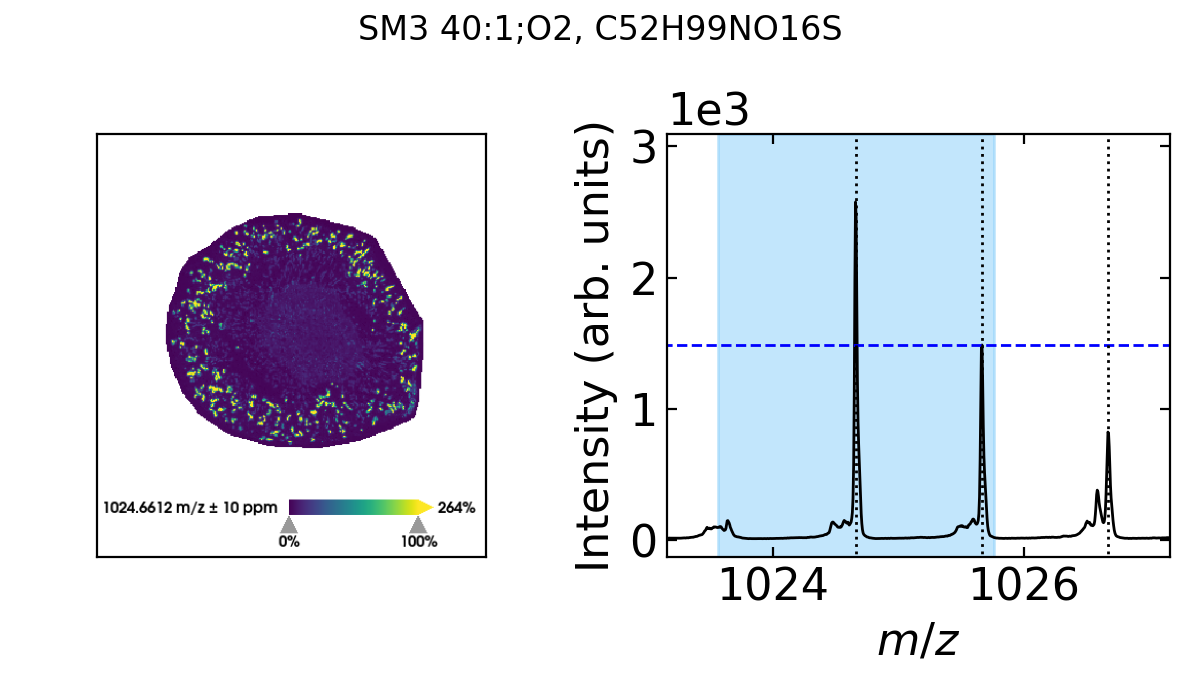

Supplement: Supplementary file 3 — Supplementary Data 1 [file 41467_2025_59839_MOESM3_ESM.zip › Suppl_Dataset_1_REV/qTOF_data3_slide1_python/1024.661181_qTOF_12w_1.png]

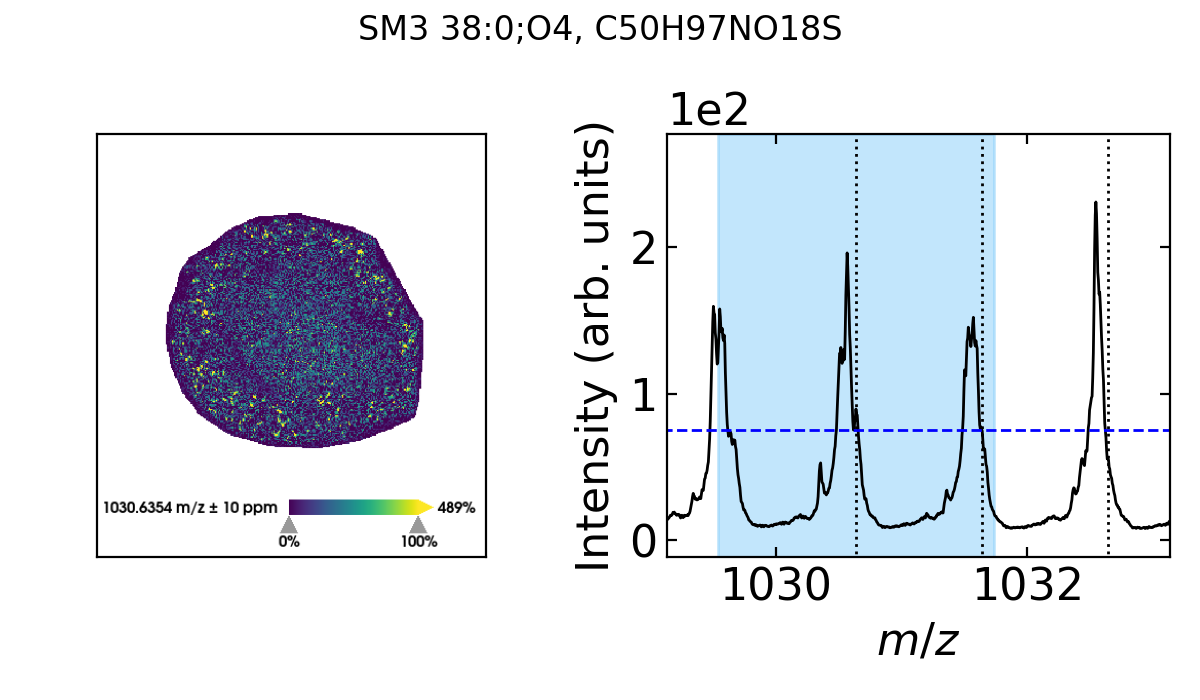

Supplement: Supplementary file 3 — Supplementary Data 1 [file 41467_2025_59839_MOESM3_ESM.zip › Suppl_Dataset_1_REV/qTOF_data3_slide1_python/1030.63536_qTOF_12w_1.png]

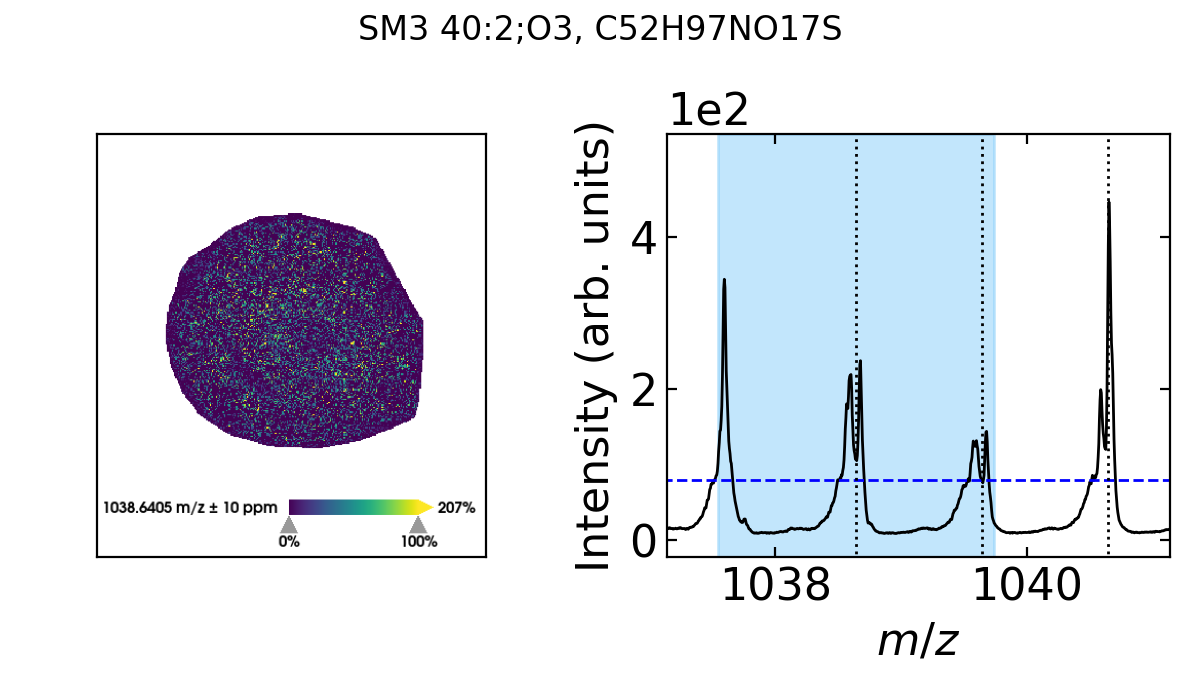

Supplement: Supplementary file 3 — Supplementary Data 1 [file 41467_2025_59839_MOESM3_ESM.zip › Suppl_Dataset_1_REV/qTOF_data3_slide1_python/1038.640446_qTOF_12w_1.png]

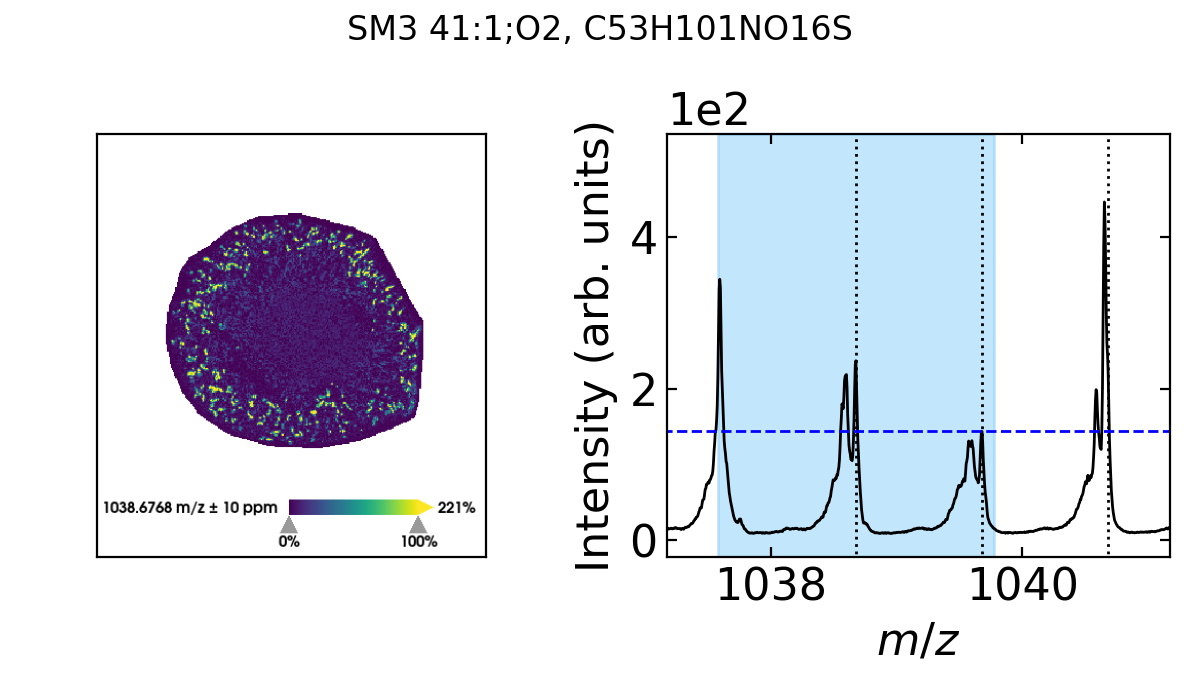

Supplement: Supplementary file 3 — Supplementary Data 1 [file 41467_2025_59839_MOESM3_ESM.zip › Suppl_Dataset_1_REV/qTOF_data3_slide1_python/1038.676831_qTOF_12w_1.png]

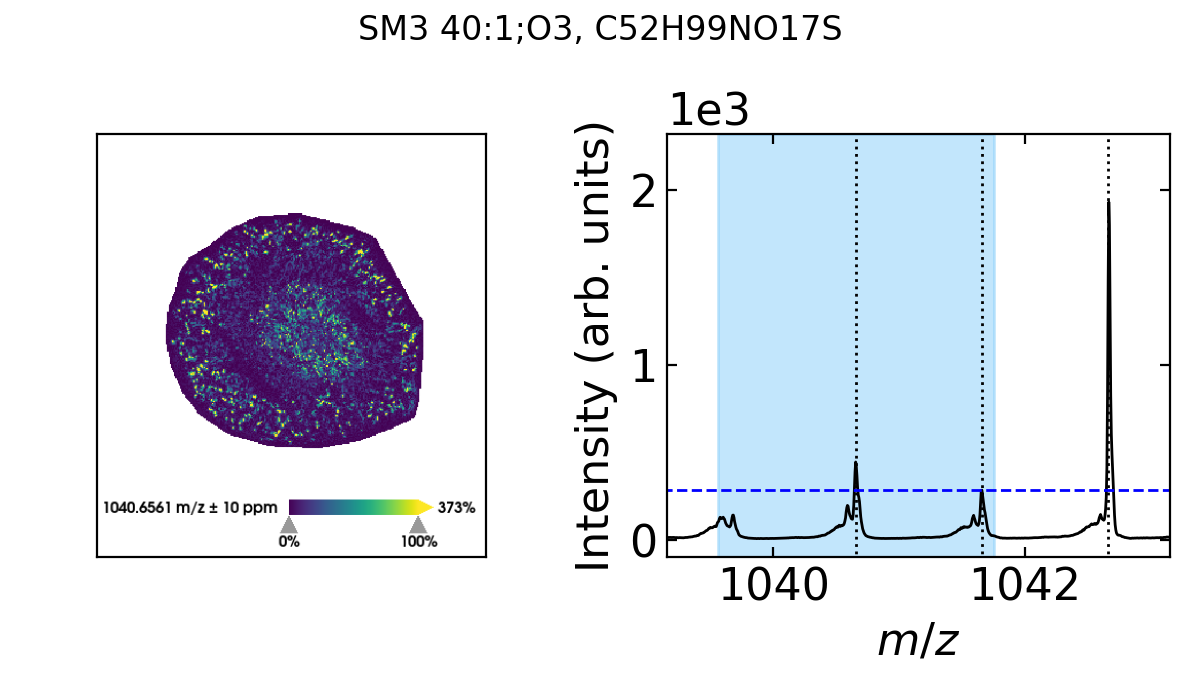

Supplement: Supplementary file 3 — Supplementary Data 1 [file 41467_2025_59839_MOESM3_ESM.zip › Suppl_Dataset_1_REV/qTOF_data3_slide1_python/1040.656096_qTOF_12w_1.png]

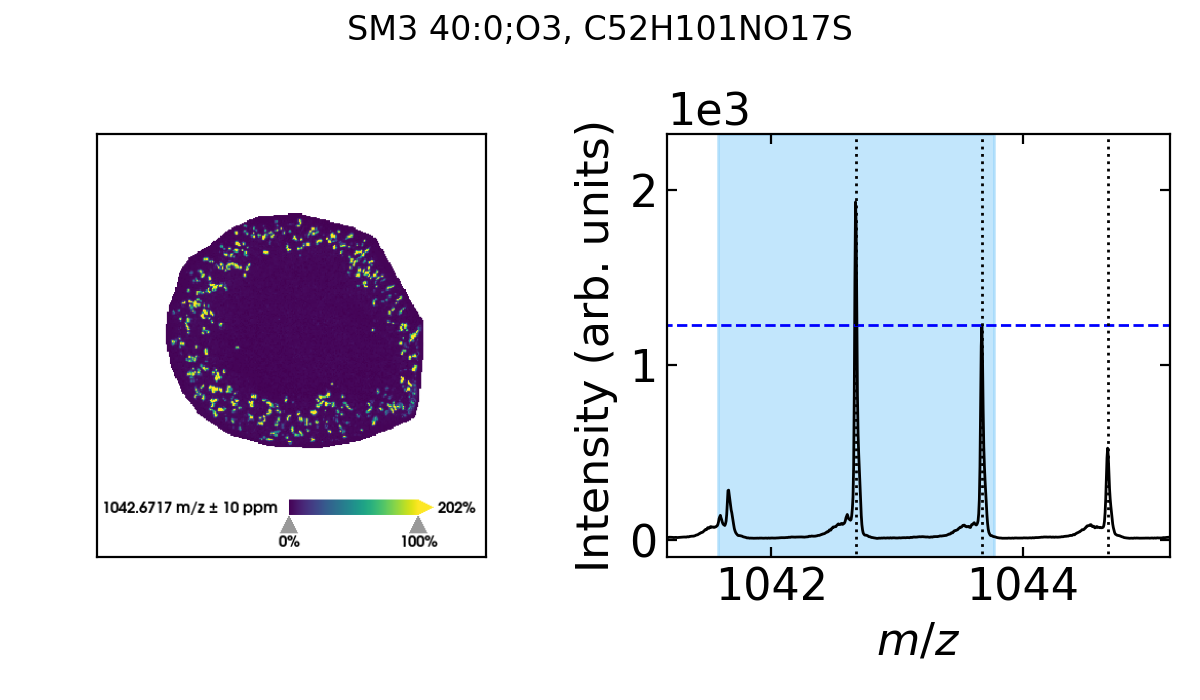

Supplement: Supplementary file 3 — Supplementary Data 1 [file 41467_2025_59839_MOESM3_ESM.zip › Suppl_Dataset_1_REV/qTOF_data3_slide1_python/1042.671746_qTOF_12w_1.png]

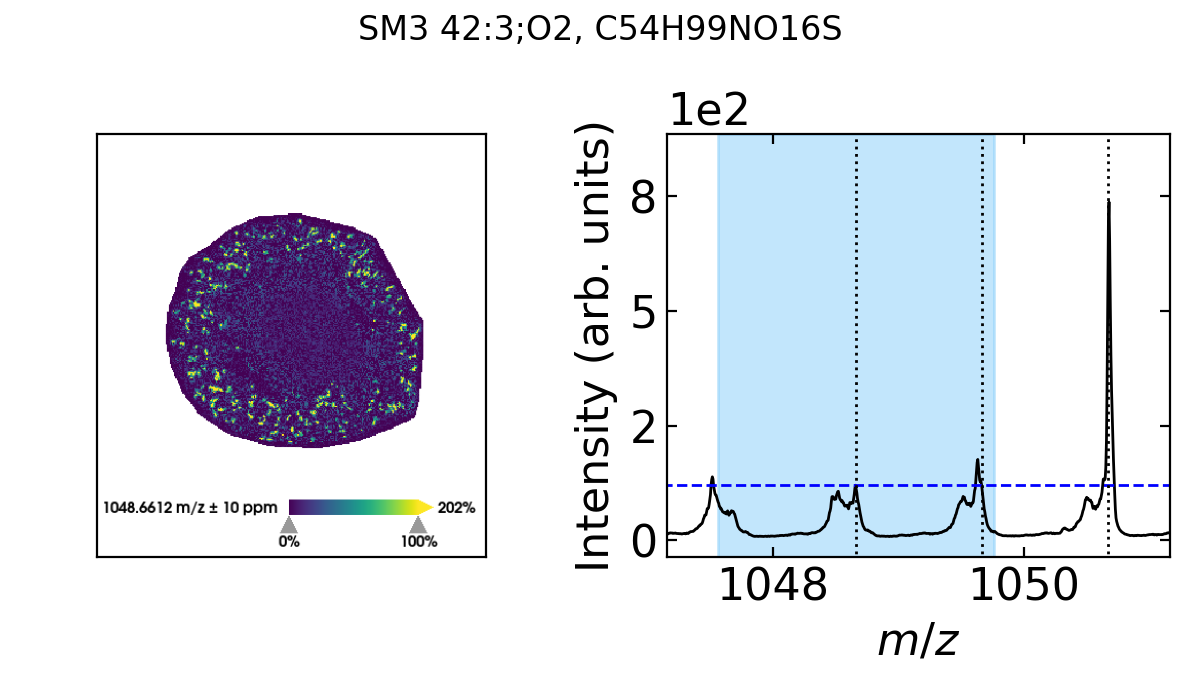

Supplement: Supplementary file 3 — Supplementary Data 1 [file 41467_2025_59839_MOESM3_ESM.zip › Suppl_Dataset_1_REV/qTOF_data3_slide1_python/1048.661181_qTOF_12w_1.png]

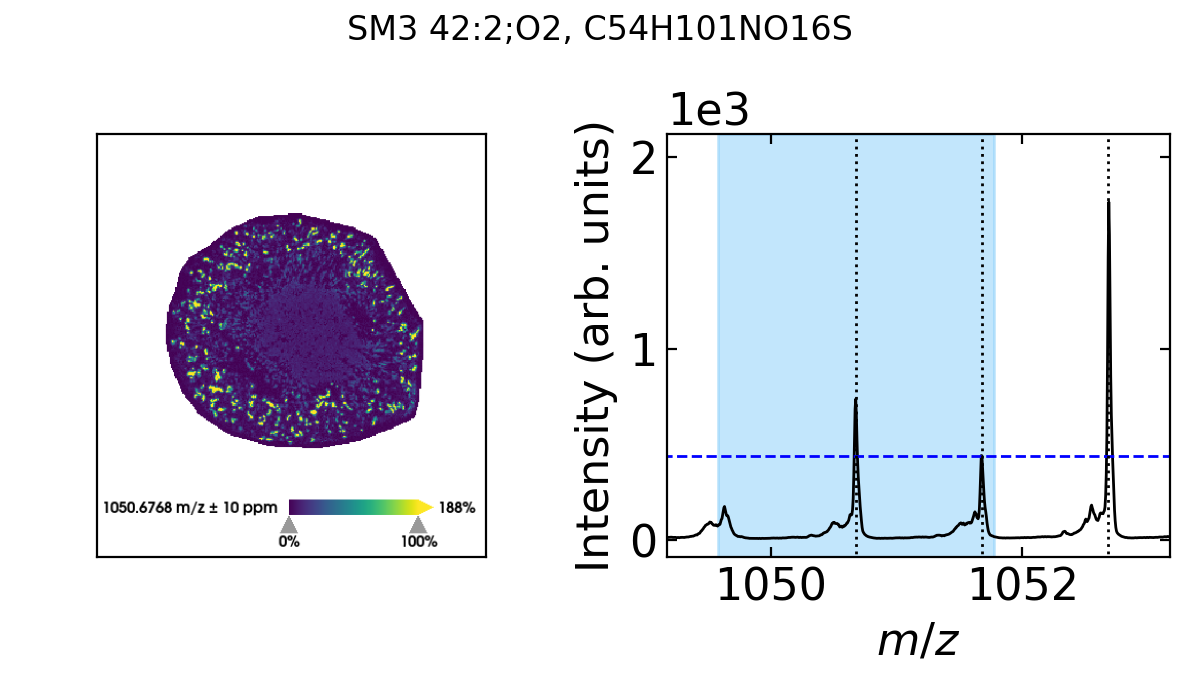

Supplement: Supplementary file 3 — Supplementary Data 1 [file 41467_2025_59839_MOESM3_ESM.zip › Suppl_Dataset_1_REV/qTOF_data3_slide1_python/1050.676831_qTOF_12w_1.png]

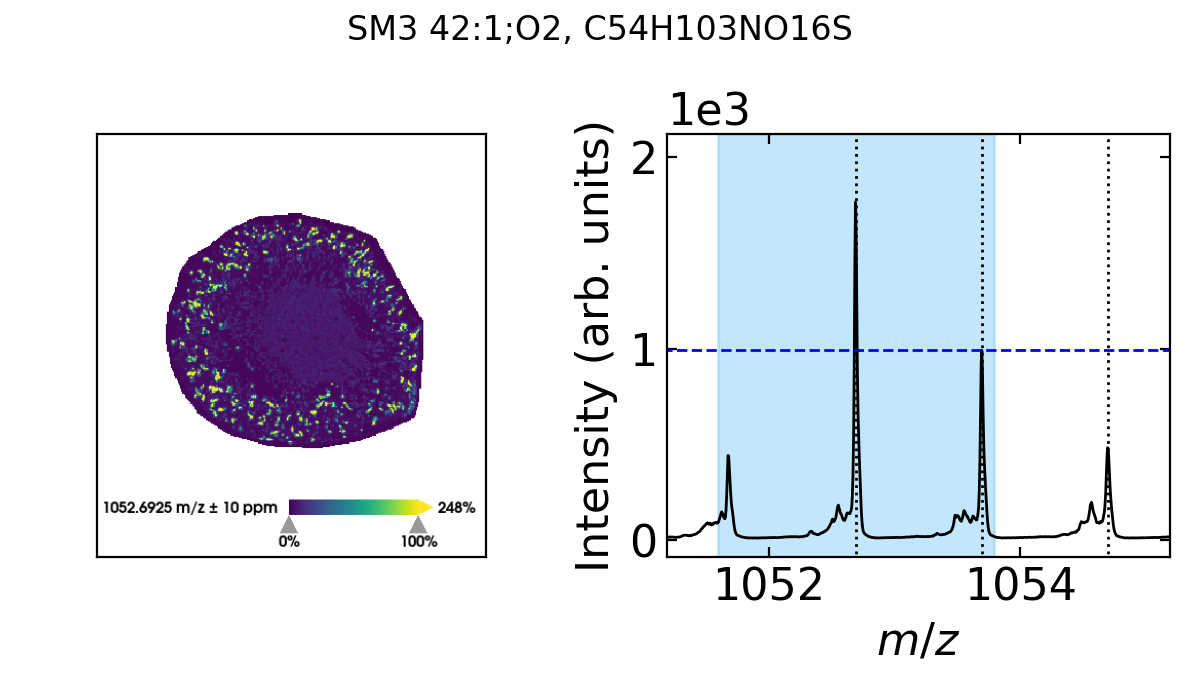

Supplement: Supplementary file 3 — Supplementary Data 1 [file 41467_2025_59839_MOESM3_ESM.zip › Suppl_Dataset_1_REV/qTOF_data3_slide1_python/1052.692481_qTOF_12w_1.png]

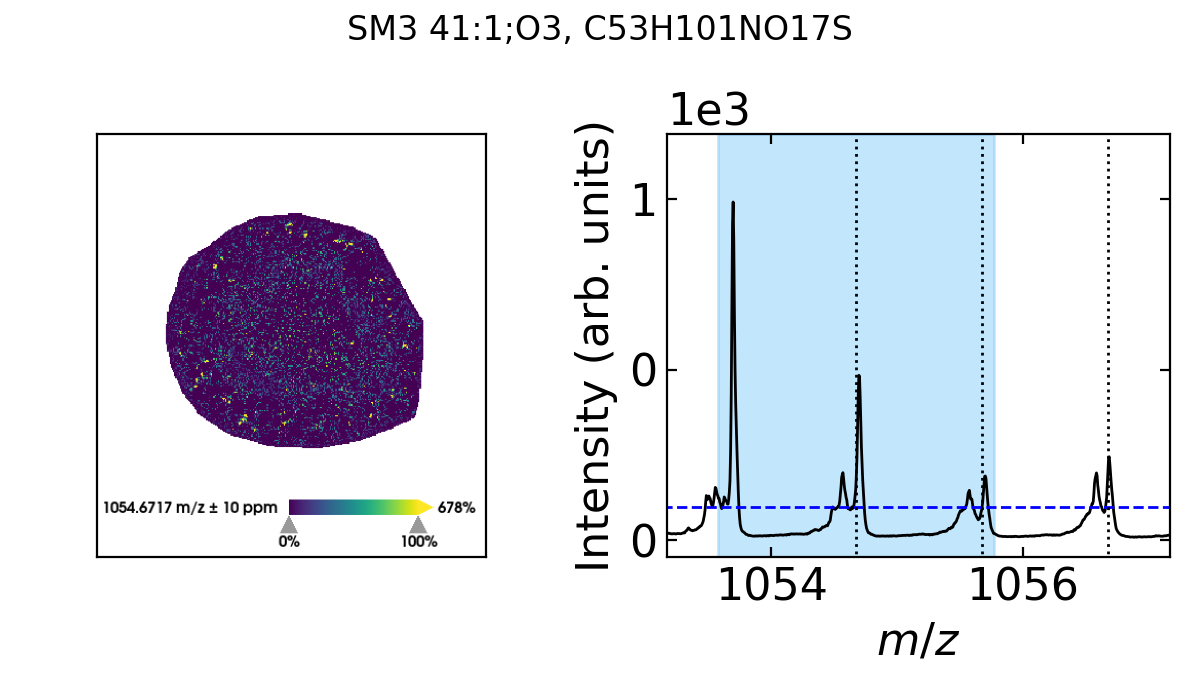

Supplement: Supplementary file 3 — Supplementary Data 1 [file 41467_2025_59839_MOESM3_ESM.zip › Suppl_Dataset_1_REV/qTOF_data3_slide1_python/1054.671746_qTOF_12w_1.png]

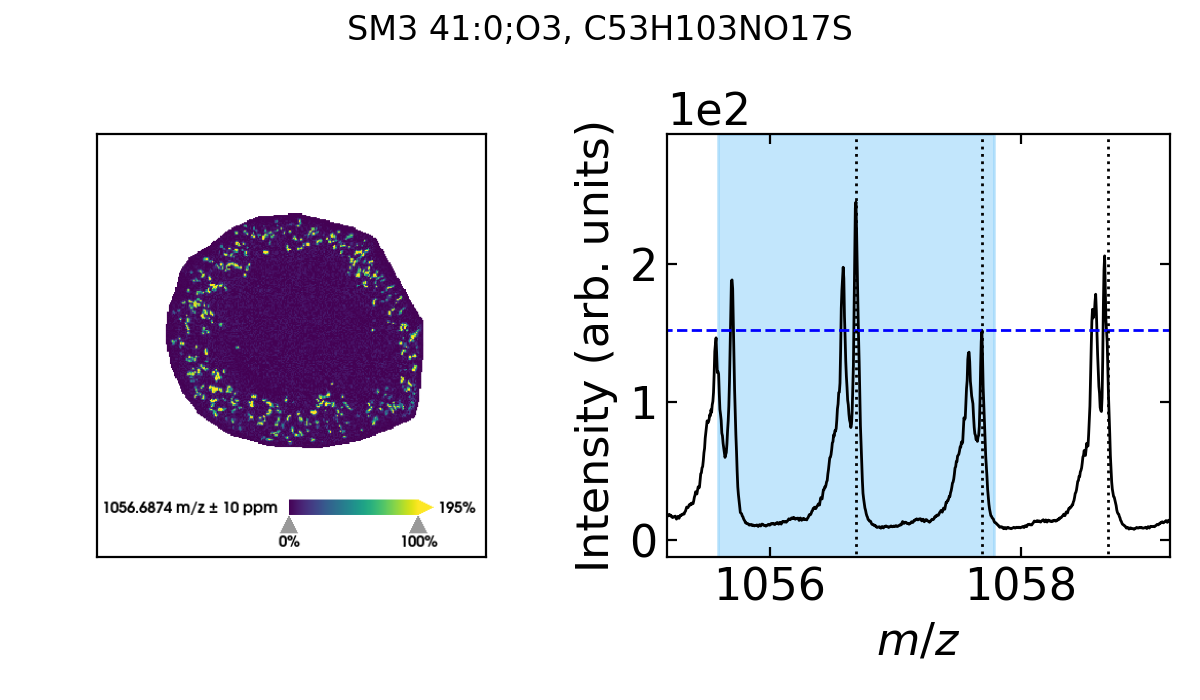

Supplement: Supplementary file 3 — Supplementary Data 1 [file 41467_2025_59839_MOESM3_ESM.zip › Suppl_Dataset_1_REV/qTOF_data3_slide1_python/1056.687396_qTOF_12w_1.png]

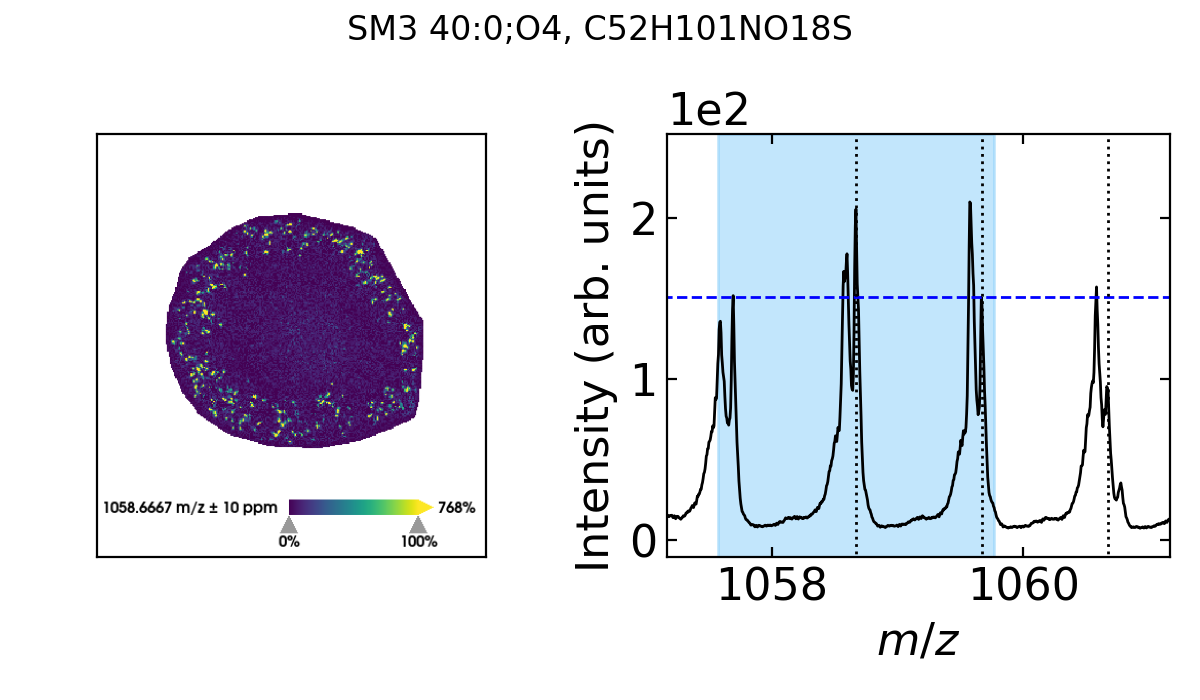

Supplement: Supplementary file 3 — Supplementary Data 1 [file 41467_2025_59839_MOESM3_ESM.zip › Suppl_Dataset_1_REV/qTOF_data3_slide1_python/1058.666661_qTOF_12w_1.png]

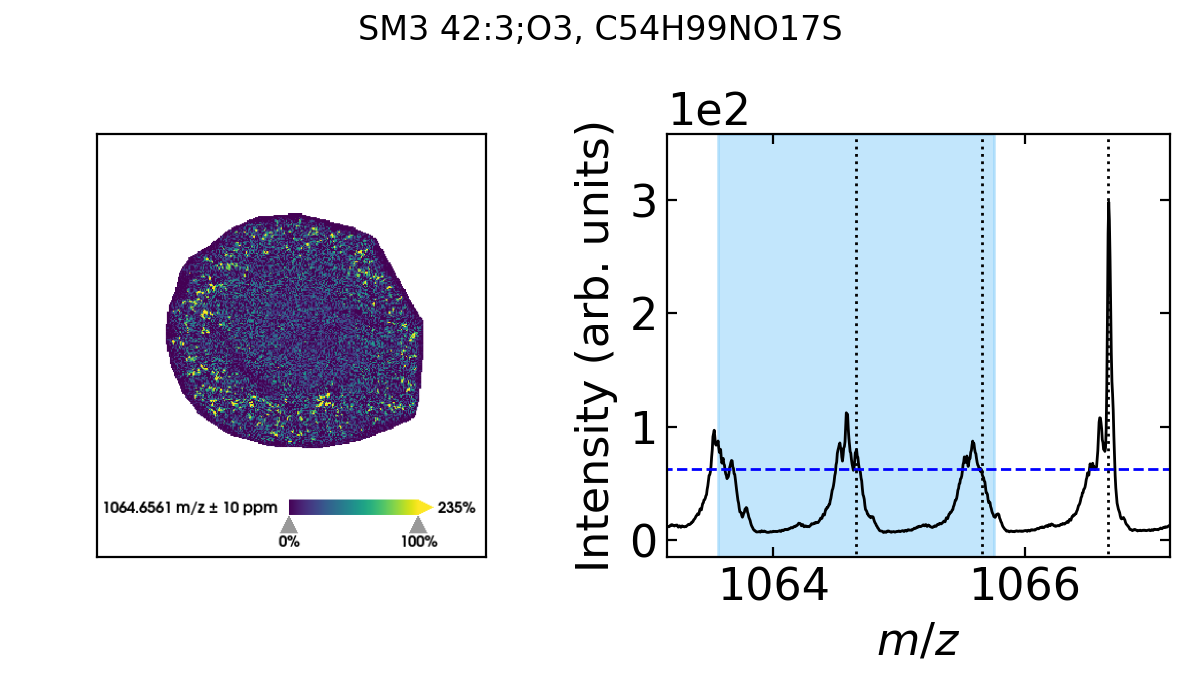

Supplement: Supplementary file 3 — Supplementary Data 1 [file 41467_2025_59839_MOESM3_ESM.zip › Suppl_Dataset_1_REV/qTOF_data3_slide1_python/1064.656096_qTOF_12w_1.png]

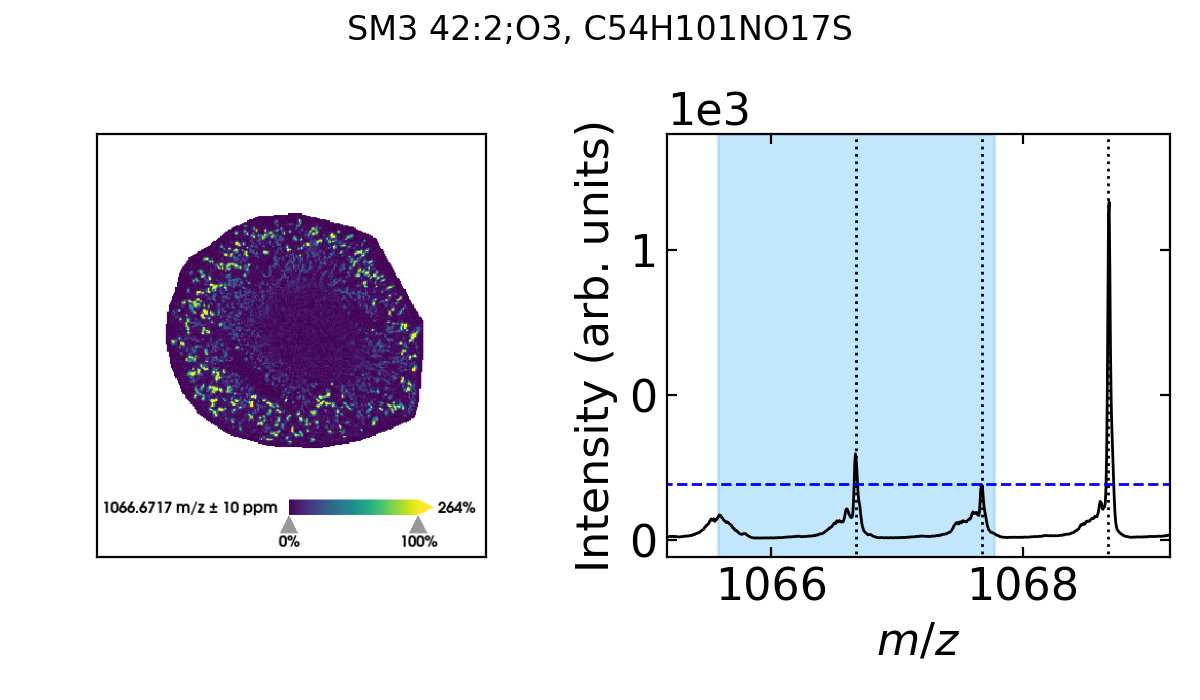

Supplement: Supplementary file 3 — Supplementary Data 1 [file 41467_2025_59839_MOESM3_ESM.zip › Suppl_Dataset_1_REV/qTOF_data3_slide1_python/1066.671746_qTOF_12w_1.png]

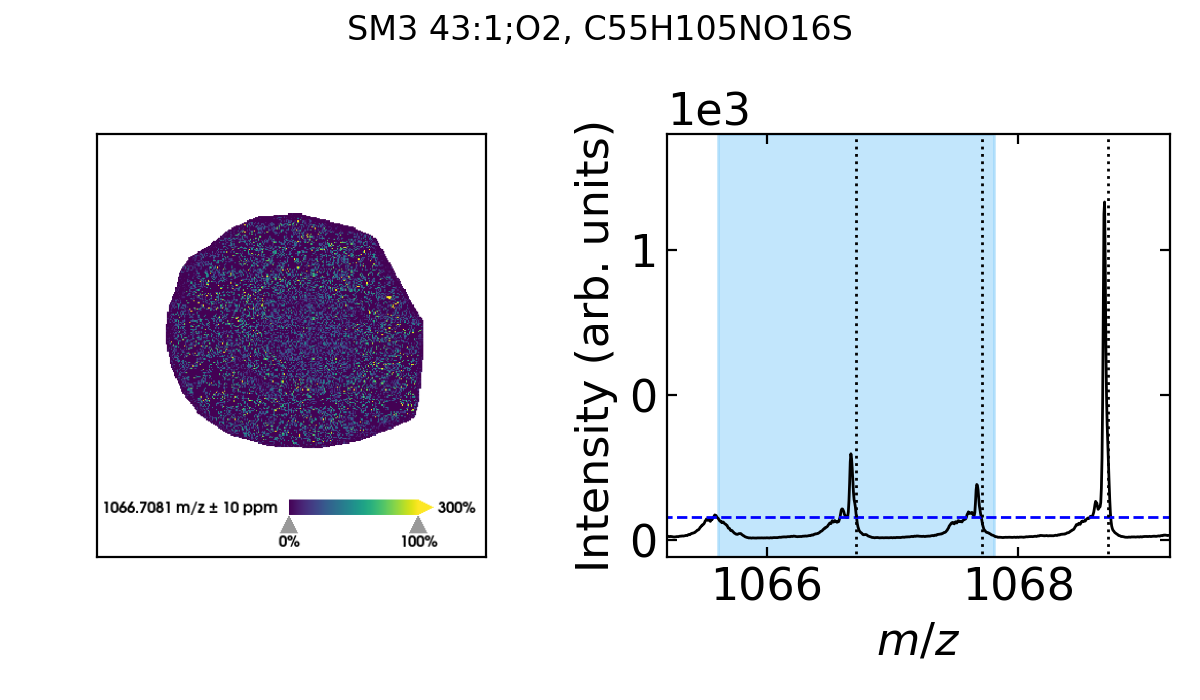

Supplement: Supplementary file 3 — Supplementary Data 1 [file 41467_2025_59839_MOESM3_ESM.zip › Suppl_Dataset_1_REV/qTOF_data3_slide1_python/1066.708131_qTOF_12w_1.png]

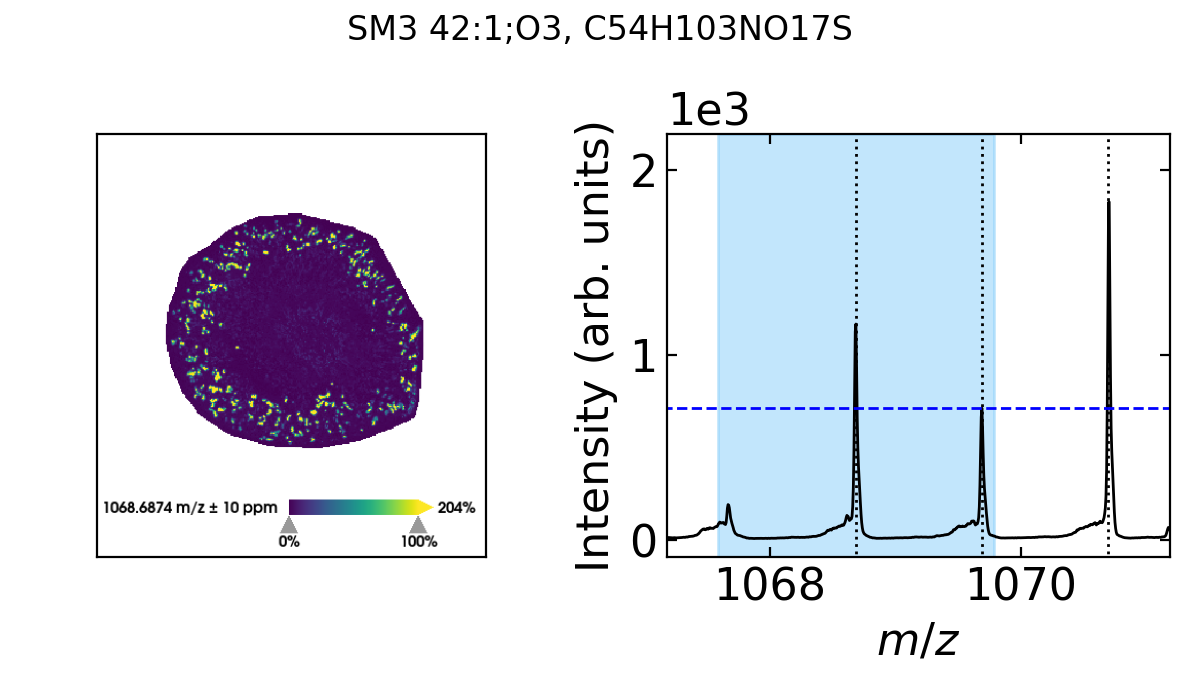

Supplement: Supplementary file 3 — Supplementary Data 1 [file 41467_2025_59839_MOESM3_ESM.zip › Suppl_Dataset_1_REV/qTOF_data3_slide1_python/1068.687396_qTOF_12w_1.png]

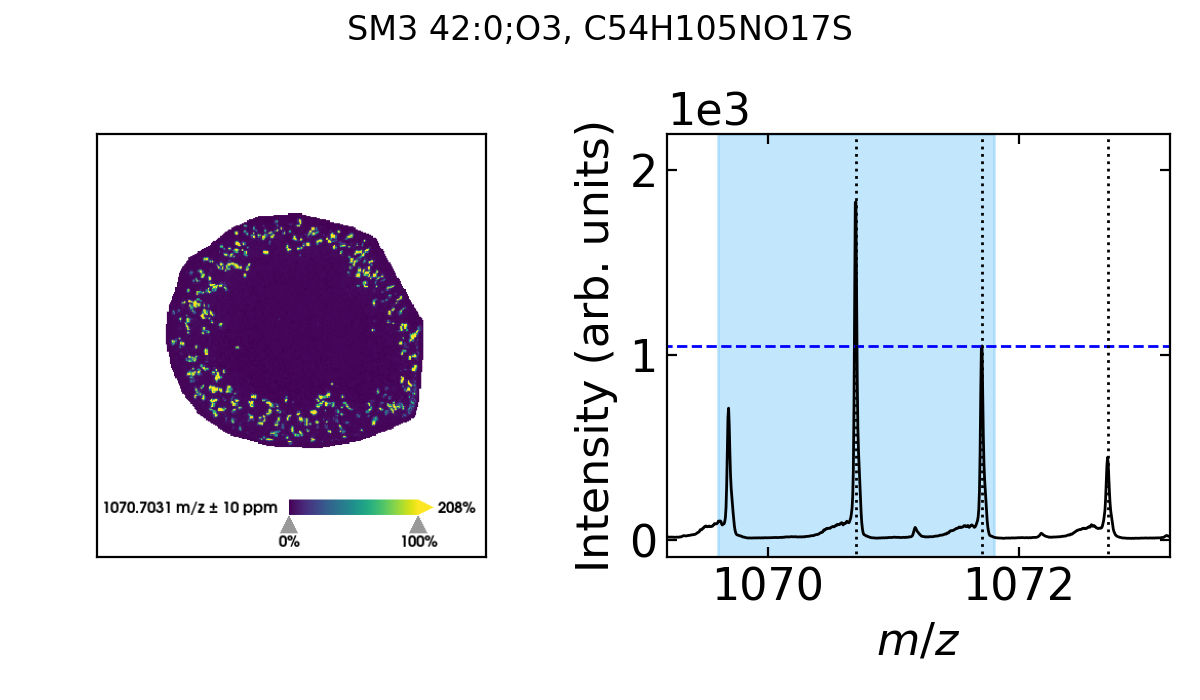

Supplement: Supplementary file 3 — Supplementary Data 1 [file 41467_2025_59839_MOESM3_ESM.zip › Suppl_Dataset_1_REV/qTOF_data3_slide1_python/1070.703046_qTOF_12w_1.png]

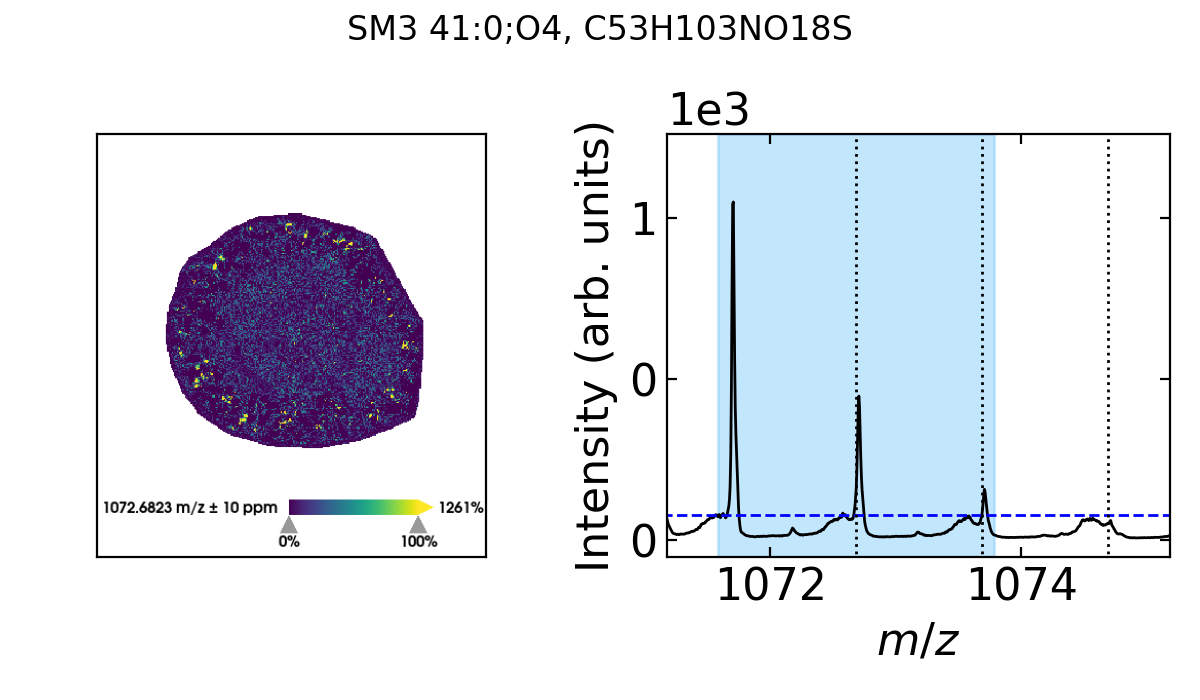

Supplement: Supplementary file 3 — Supplementary Data 1 [file 41467_2025_59839_MOESM3_ESM.zip › Suppl_Dataset_1_REV/qTOF_data3_slide1_python/1072.682311_qTOF_12w_1.png]

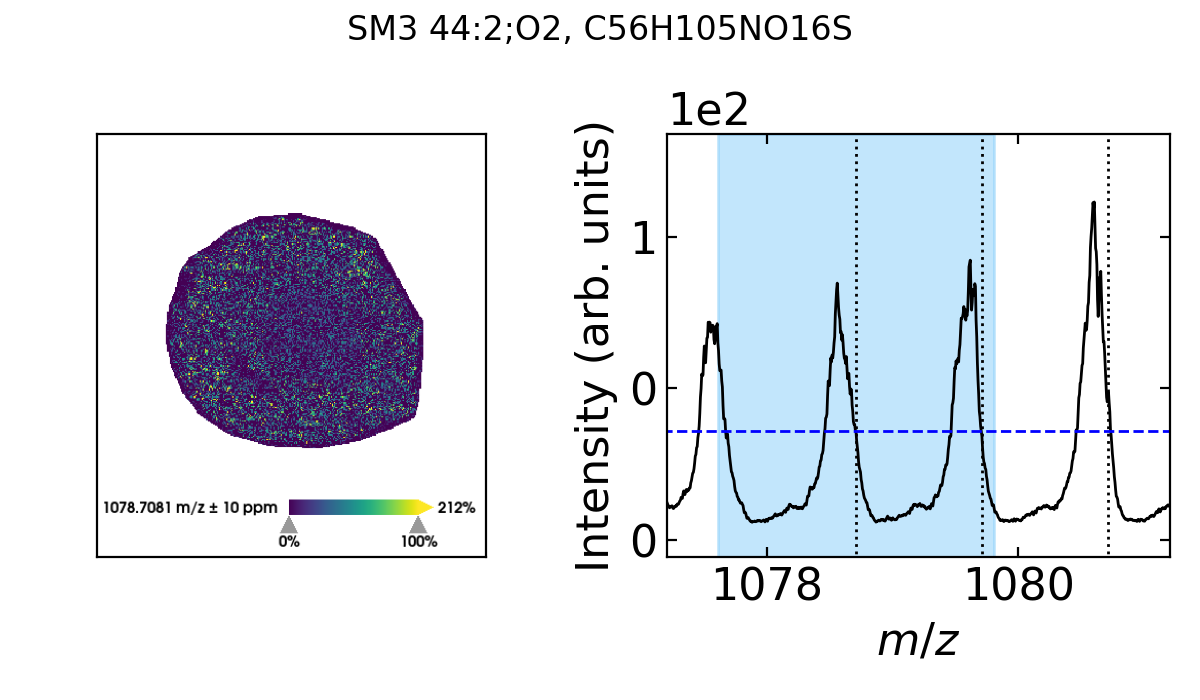

Supplement: Supplementary file 3 — Supplementary Data 1 [file 41467_2025_59839_MOESM3_ESM.zip › Suppl_Dataset_1_REV/qTOF_data3_slide1_python/1078.708131_qTOF_12w_1.png]

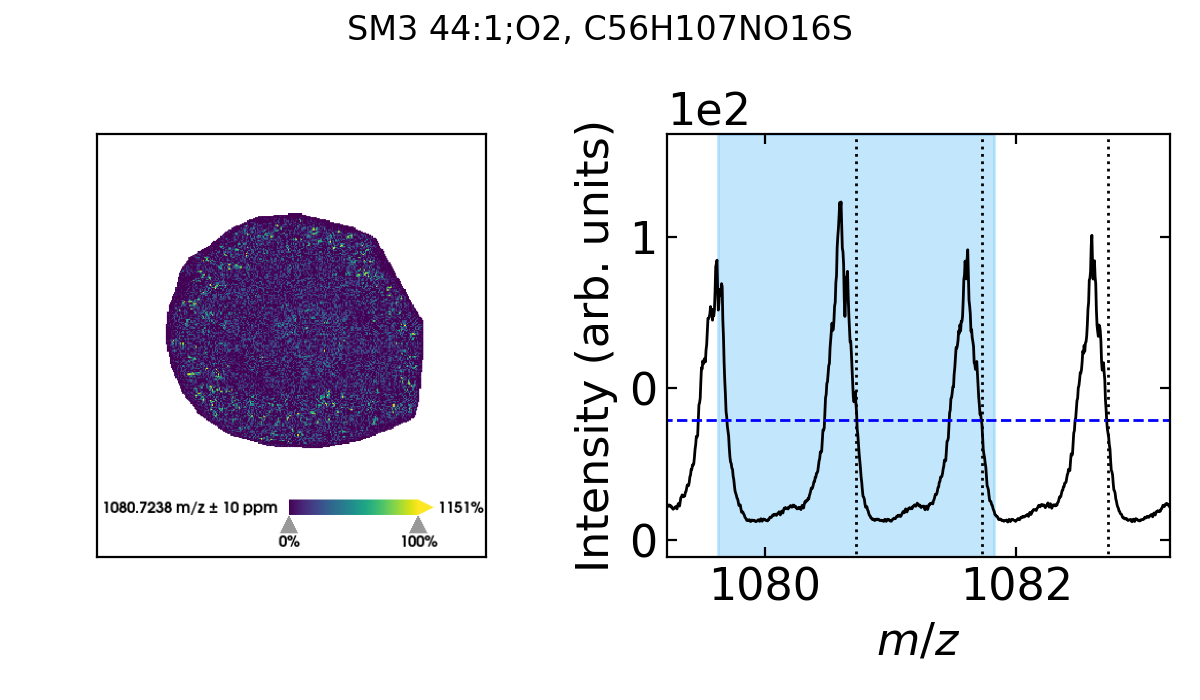

Supplement: Supplementary file 3 — Supplementary Data 1 [file 41467_2025_59839_MOESM3_ESM.zip › Suppl_Dataset_1_REV/qTOF_data3_slide1_python/1080.723781_qTOF_12w_1.png]

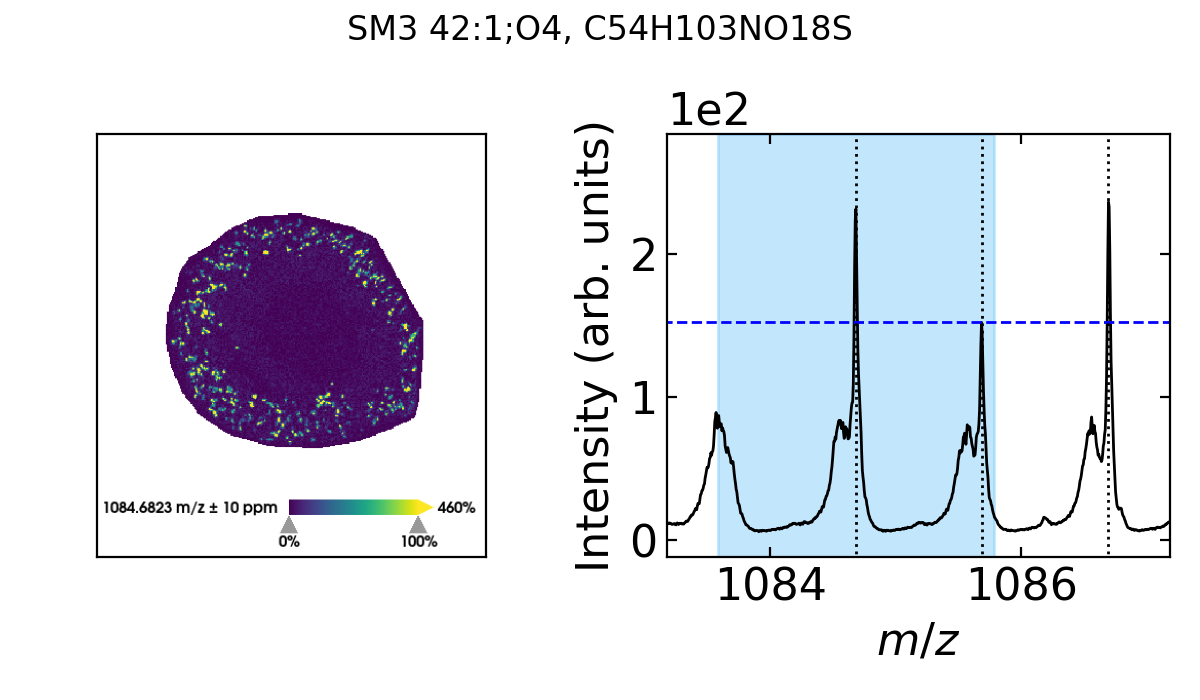

Supplement: Supplementary file 3 — Supplementary Data 1 [file 41467_2025_59839_MOESM3_ESM.zip › Suppl_Dataset_1_REV/qTOF_data3_slide1_python/1084.682311_qTOF_12w_1.png]

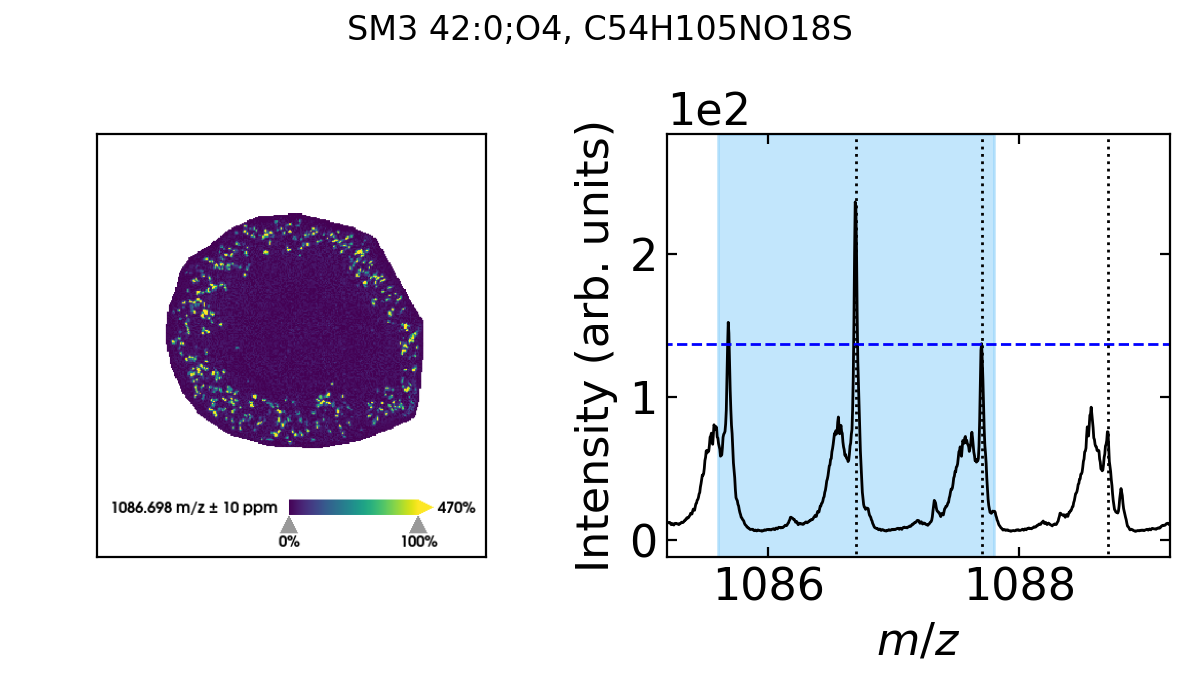

Supplement: Supplementary file 3 — Supplementary Data 1 [file 41467_2025_59839_MOESM3_ESM.zip › Suppl_Dataset_1_REV/qTOF_data3_slide1_python/1086.697961_qTOF_12w_1.png]

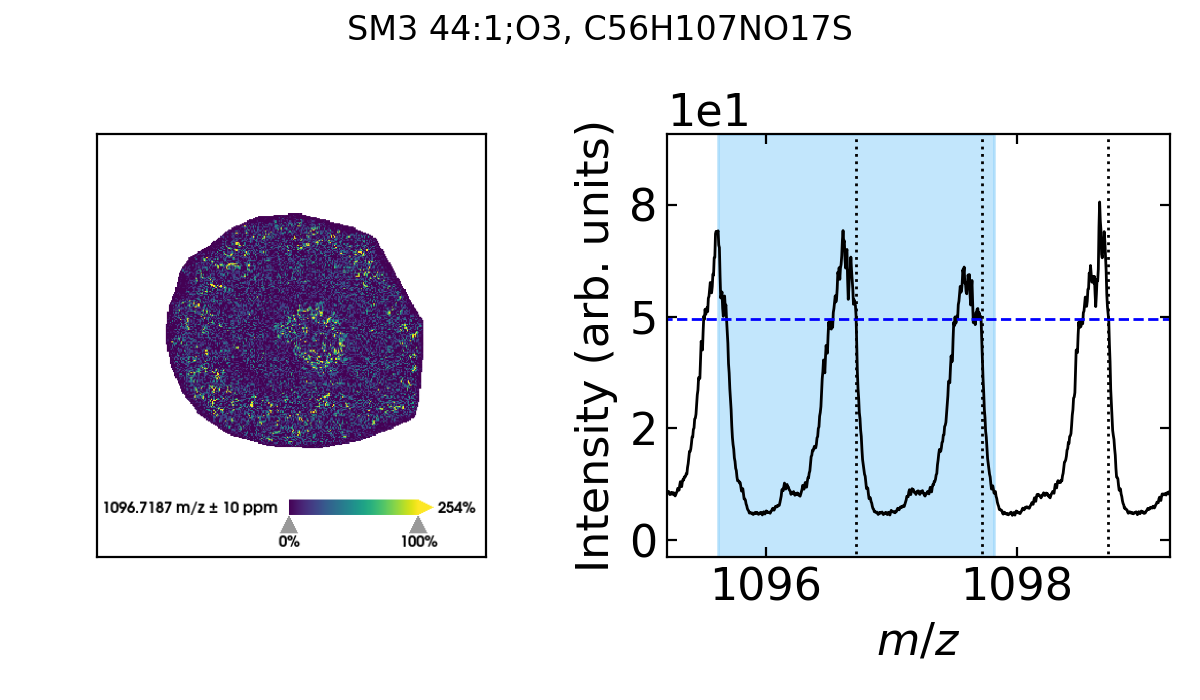

Supplement: Supplementary file 3 — Supplementary Data 1 [file 41467_2025_59839_MOESM3_ESM.zip › Suppl_Dataset_1_REV/qTOF_data3_slide1_python/1096.718696_qTOF_12w_1.png]

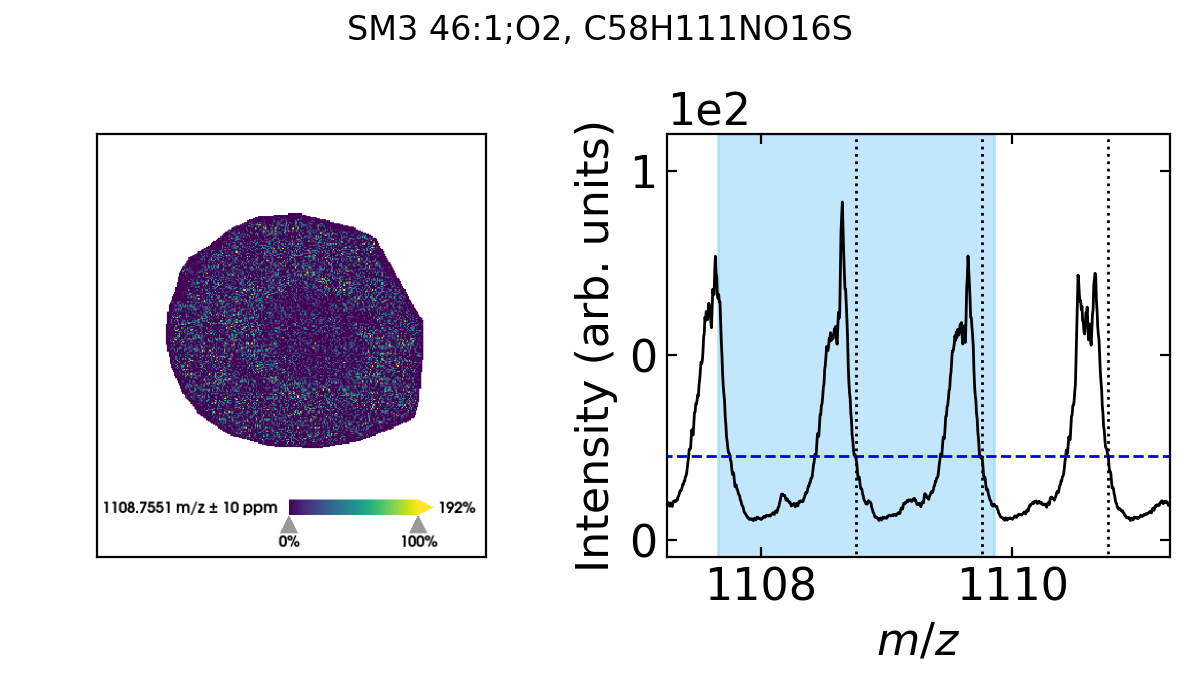

Supplement: Supplementary file 3 — Supplementary Data 1 [file 41467_2025_59839_MOESM3_ESM.zip › Suppl_Dataset_1_REV/qTOF_data3_slide1_python/1108.755082_qTOF_12w_1.png]

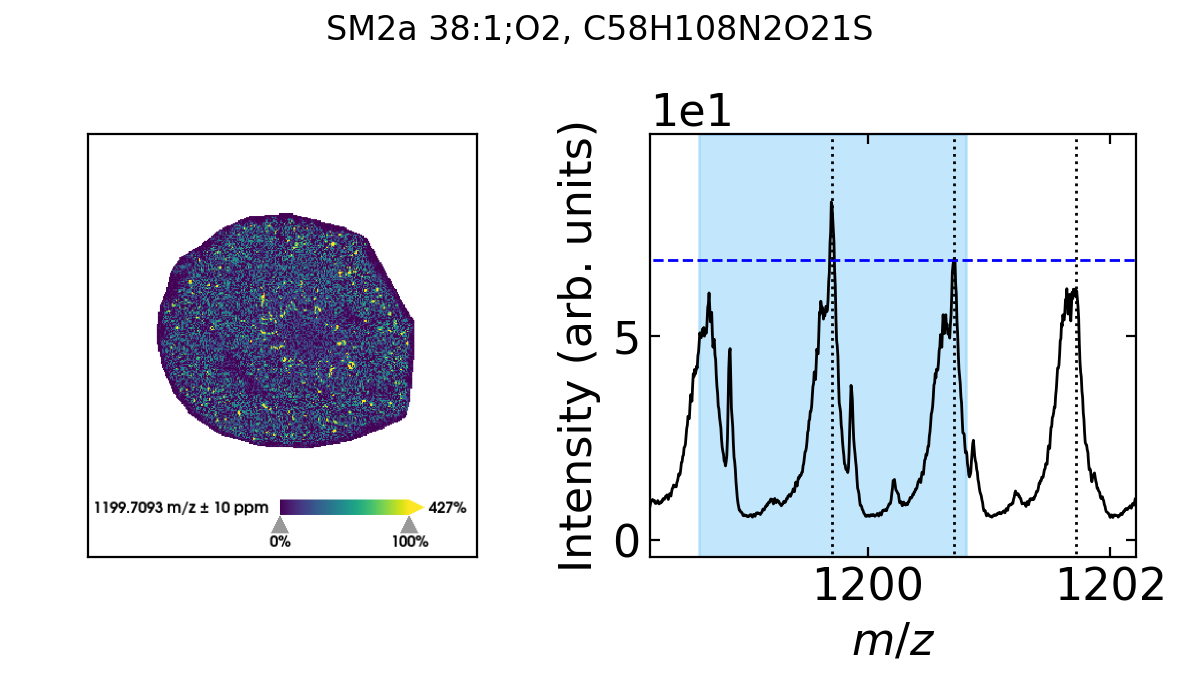

Supplement: Supplementary file 3 — Supplementary Data 1 [file 41467_2025_59839_MOESM3_ESM.zip › Suppl_Dataset_1_REV/qTOF_data3_slide1_python/1199.709253_qTOF_12w_1.png]

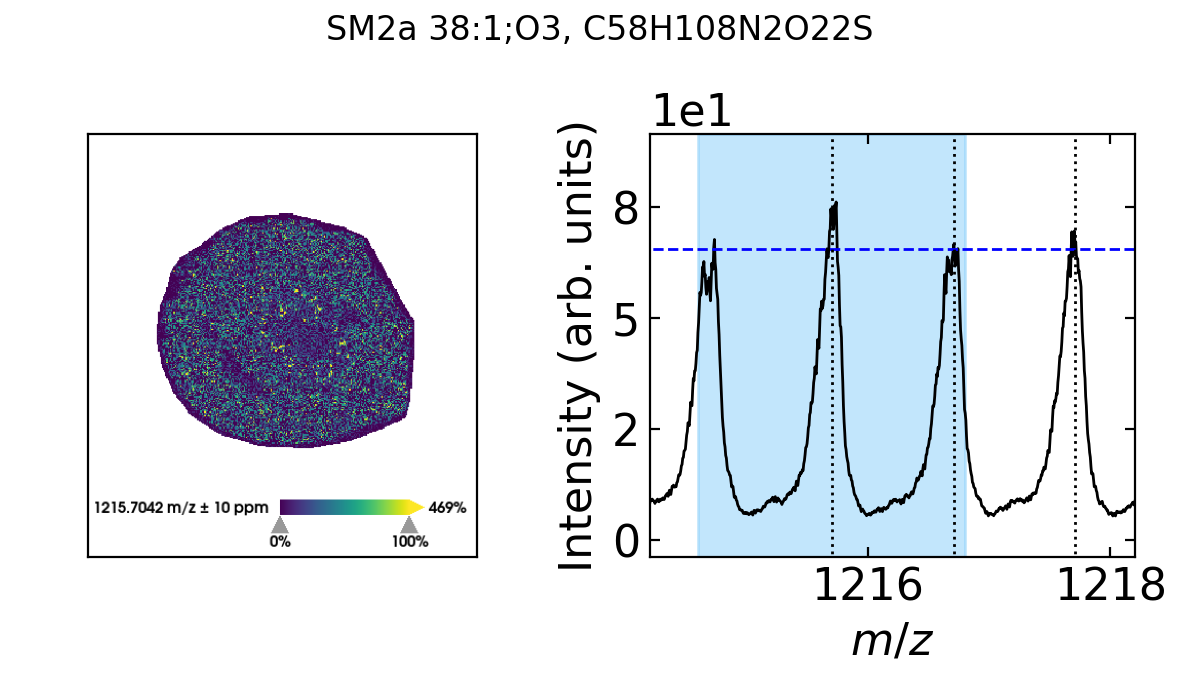

Supplement: Supplementary file 3 — Supplementary Data 1 [file 41467_2025_59839_MOESM3_ESM.zip › Suppl_Dataset_1_REV/qTOF_data3_slide1_python/1215.704168_qTOF_12w_1.png]

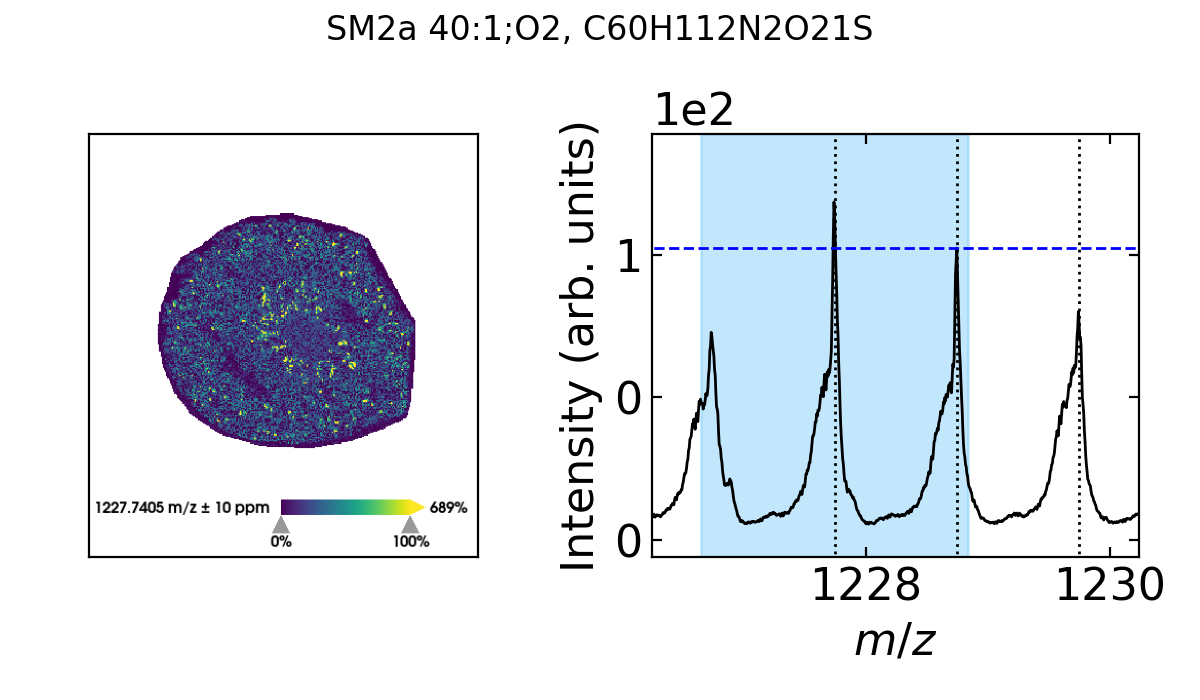

Supplement: Supplementary file 3 — Supplementary Data 1 [file 41467_2025_59839_MOESM3_ESM.zip › Suppl_Dataset_1_REV/qTOF_data3_slide1_python/1227.740553_qTOF_12w_1.png]

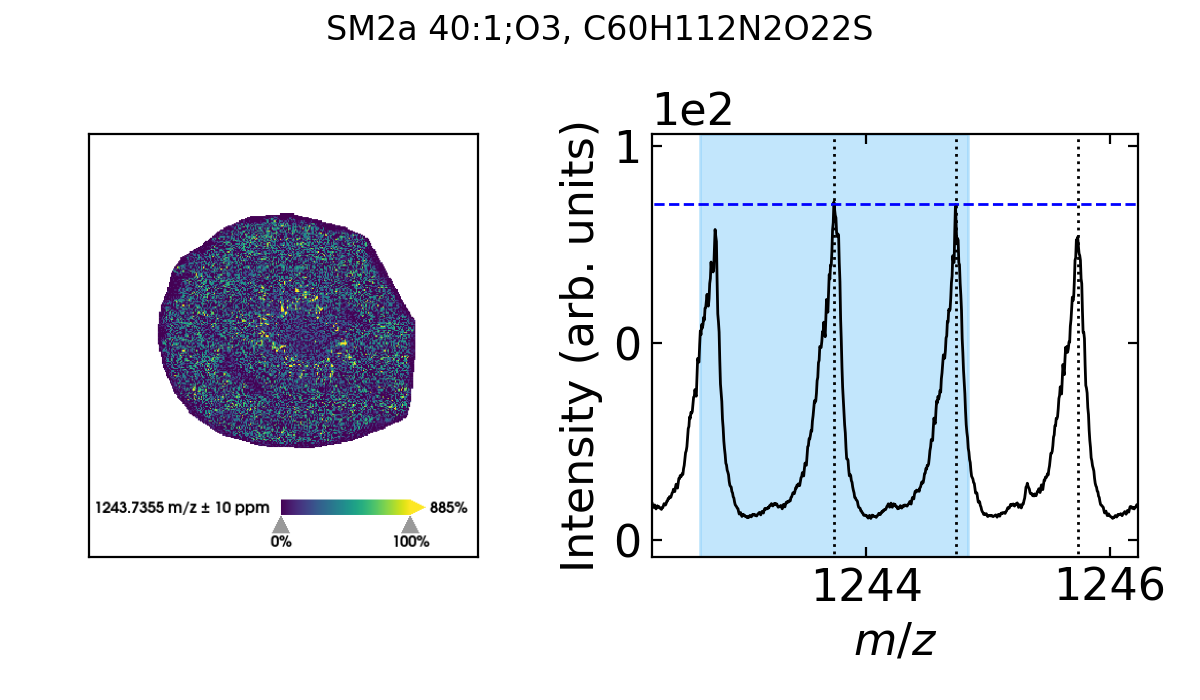

Supplement: Supplementary file 3 — Supplementary Data 1 [file 41467_2025_59839_MOESM3_ESM.zip › Suppl_Dataset_1_REV/qTOF_data3_slide1_python/1243.735468_qTOF_12w_1.png]

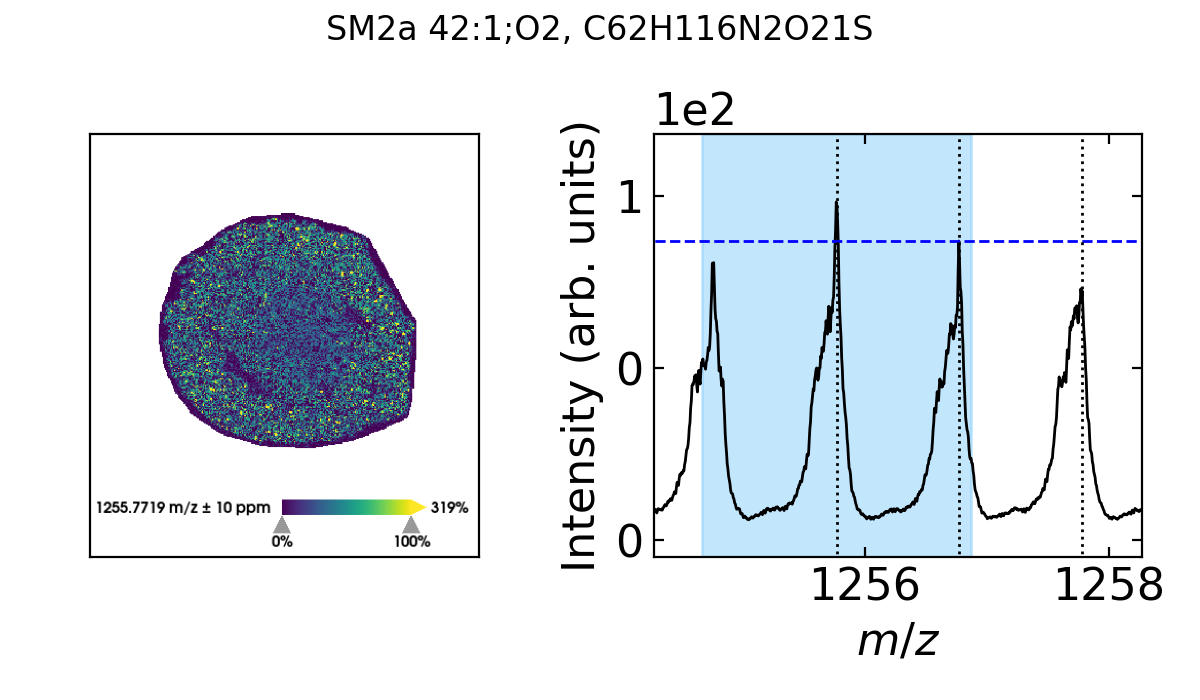

Supplement: Supplementary file 3 — Supplementary Data 1 [file 41467_2025_59839_MOESM3_ESM.zip › Suppl_Dataset_1_REV/qTOF_data3_slide1_python/1255.771853_qTOF_12w_1.png]

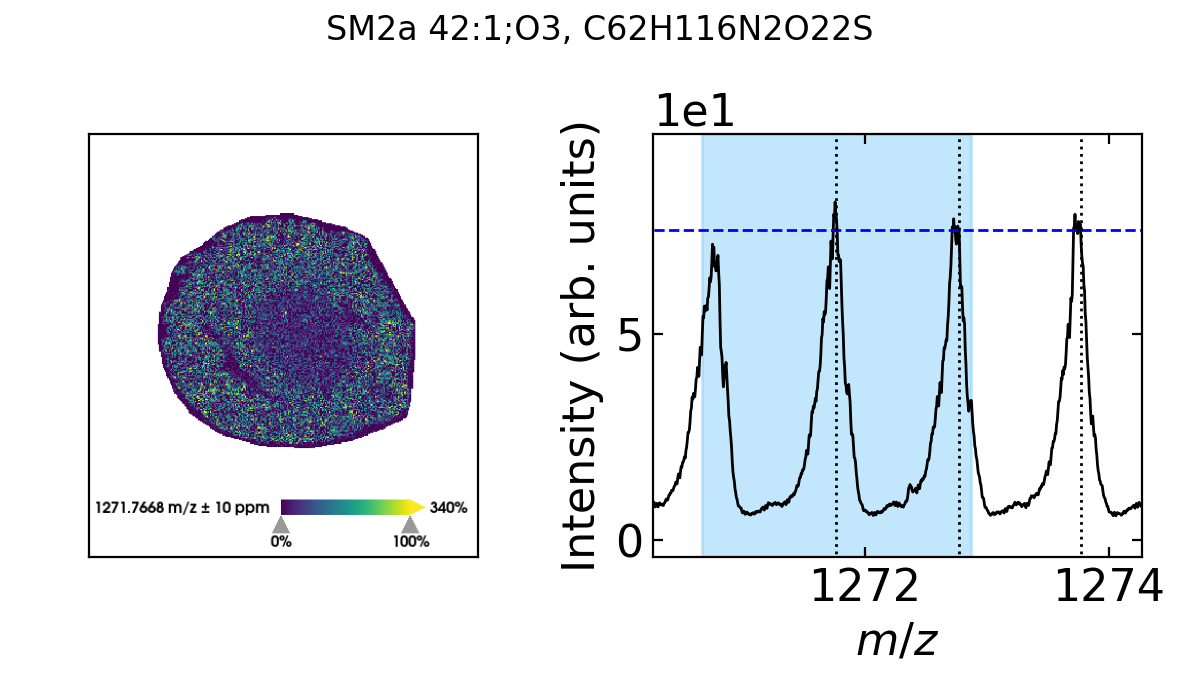

Supplement: Supplementary file 3 — Supplementary Data 1 [file 41467_2025_59839_MOESM3_ESM.zip › Suppl_Dataset_1_REV/qTOF_data3_slide1_python/1271.766768_qTOF_12w_1.png]

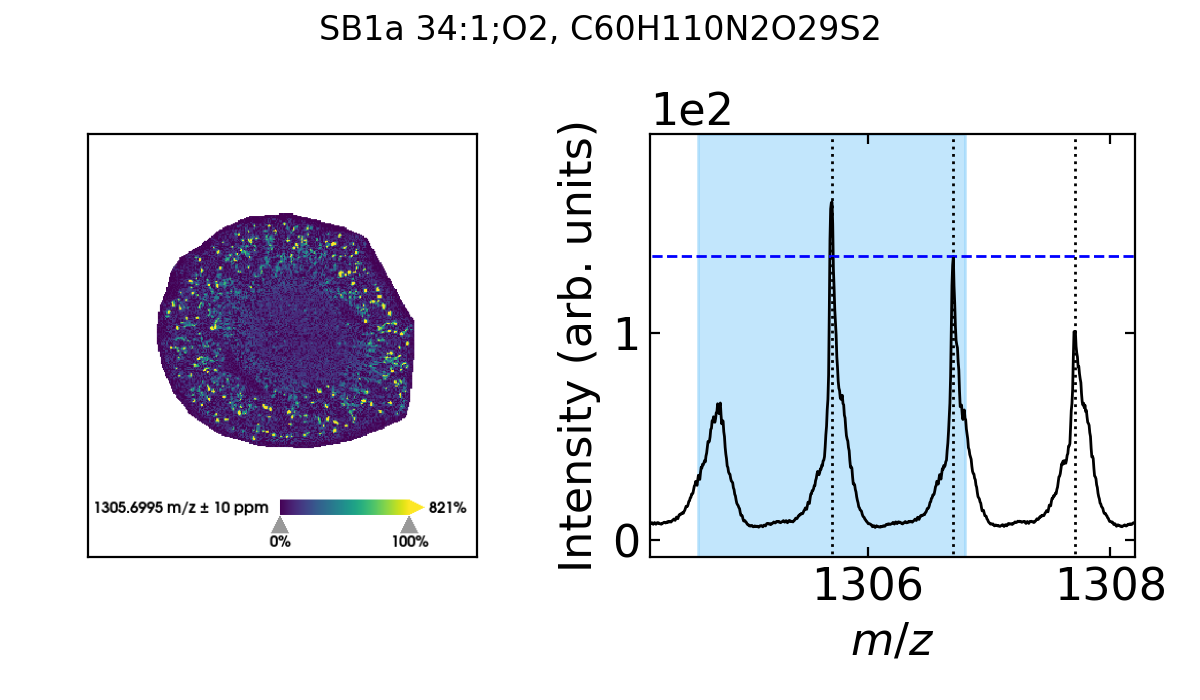

Supplement: Supplementary file 3 — Supplementary Data 1 [file 41467_2025_59839_MOESM3_ESM.zip › Suppl_Dataset_1_REV/qTOF_data3_slide1_python/1305.699477_qTOF_12w_1.png]

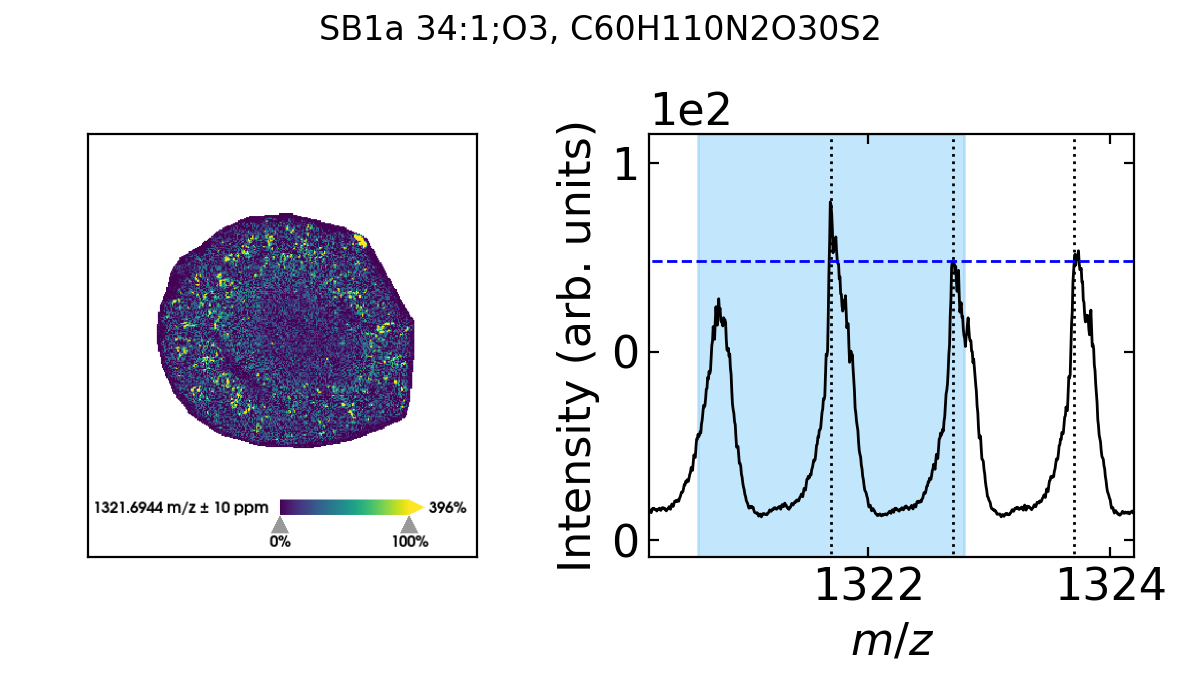

Supplement: Supplementary file 3 — Supplementary Data 1 [file 41467_2025_59839_MOESM3_ESM.zip › Suppl_Dataset_1_REV/qTOF_data3_slide1_python/1321.694392_qTOF_12w_1.png]

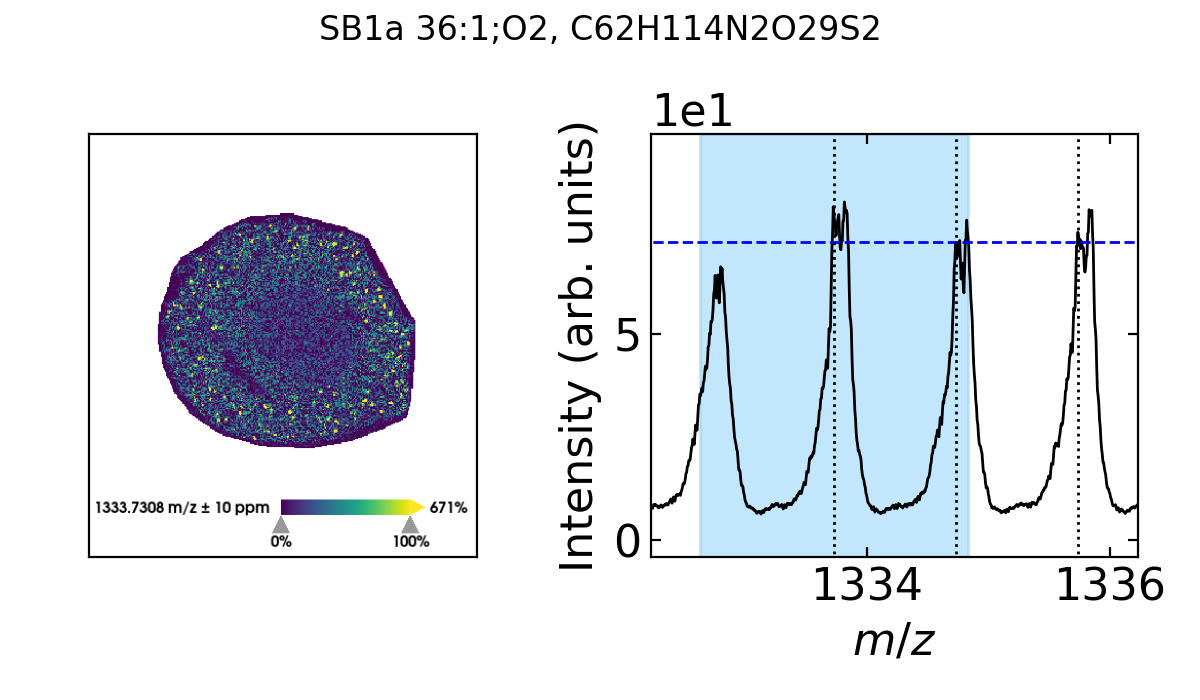

Supplement: Supplementary file 3 — Supplementary Data 1 [file 41467_2025_59839_MOESM3_ESM.zip › Suppl_Dataset_1_REV/qTOF_data3_slide1_python/1333.730777_qTOF_12w_1.png]

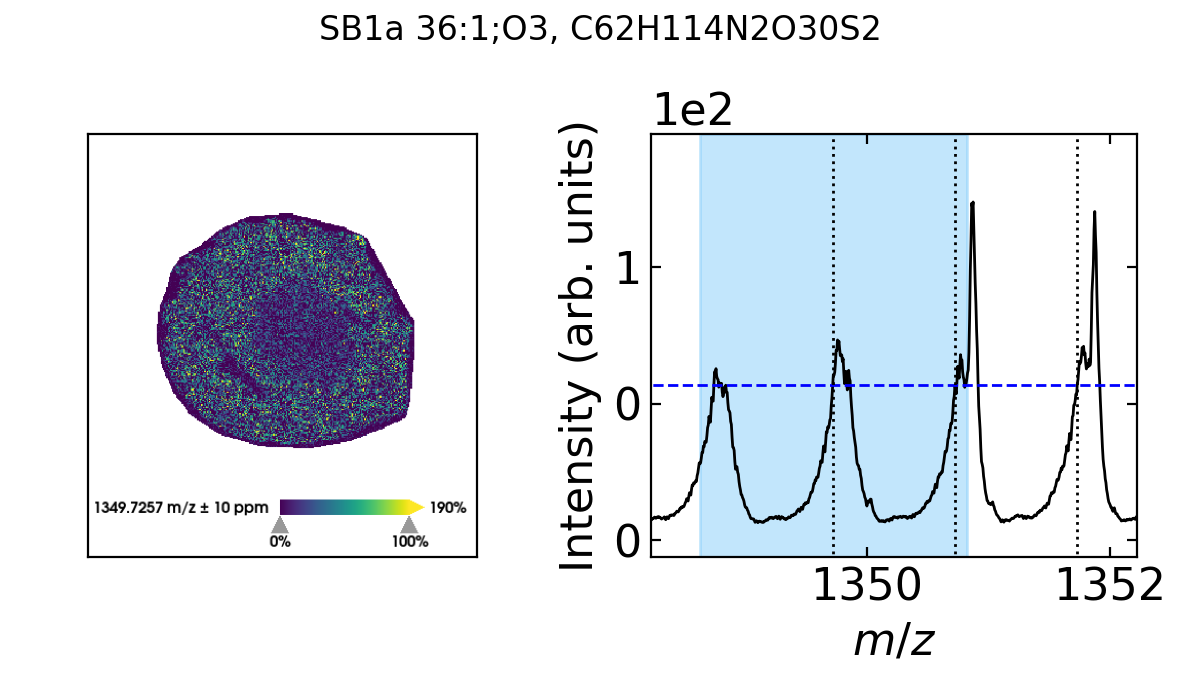

Supplement: Supplementary file 3 — Supplementary Data 1 [file 41467_2025_59839_MOESM3_ESM.zip › Suppl_Dataset_1_REV/qTOF_data3_slide1_python/1349.725692_qTOF_12w_1.png]

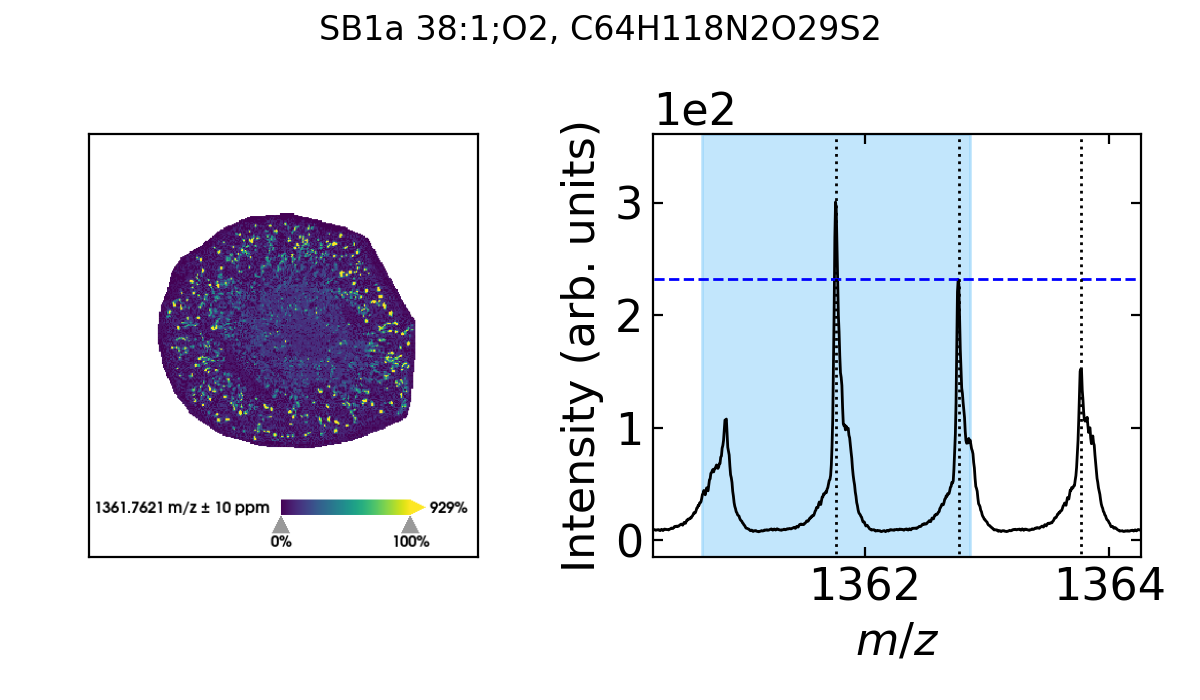

Supplement: Supplementary file 3 — Supplementary Data 1 [file 41467_2025_59839_MOESM3_ESM.zip › Suppl_Dataset_1_REV/qTOF_data3_slide1_python/1361.762077_qTOF_12w_1.png]

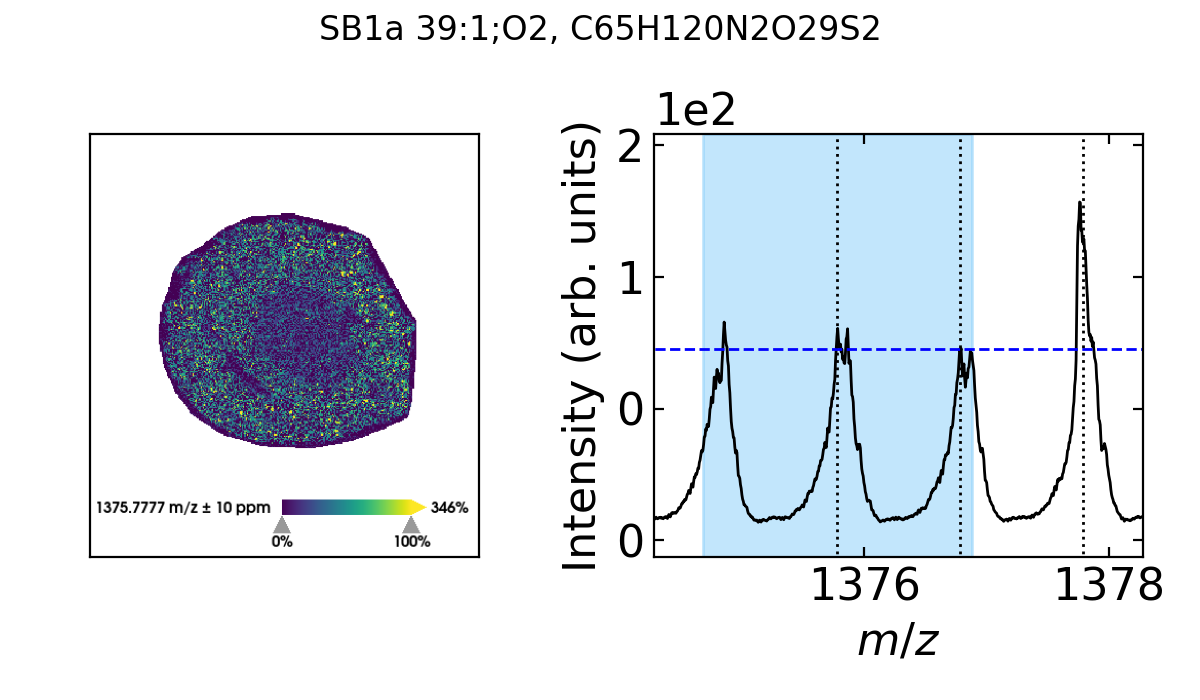

Supplement: Supplementary file 3 — Supplementary Data 1 [file 41467_2025_59839_MOESM3_ESM.zip › Suppl_Dataset_1_REV/qTOF_data3_slide1_python/1375.777727_qTOF_12w_1.png]

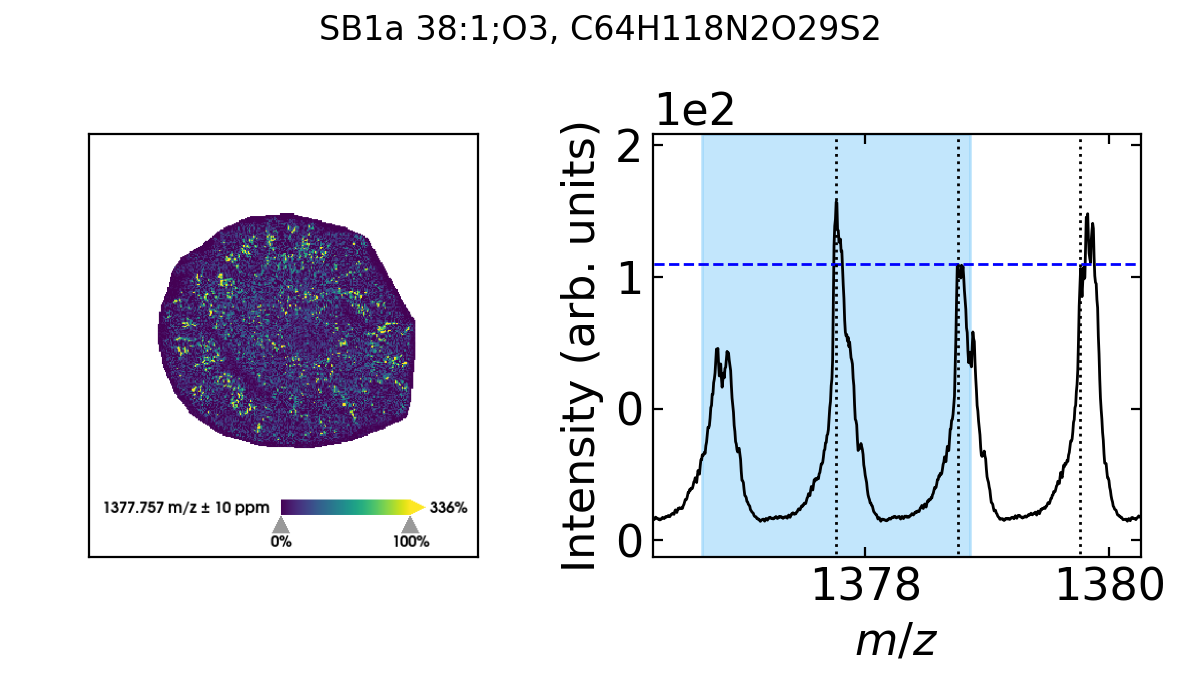

Supplement: Supplementary file 3 — Supplementary Data 1 [file 41467_2025_59839_MOESM3_ESM.zip › Suppl_Dataset_1_REV/qTOF_data3_slide1_python/1377.756991_qTOF_12w_1.png]

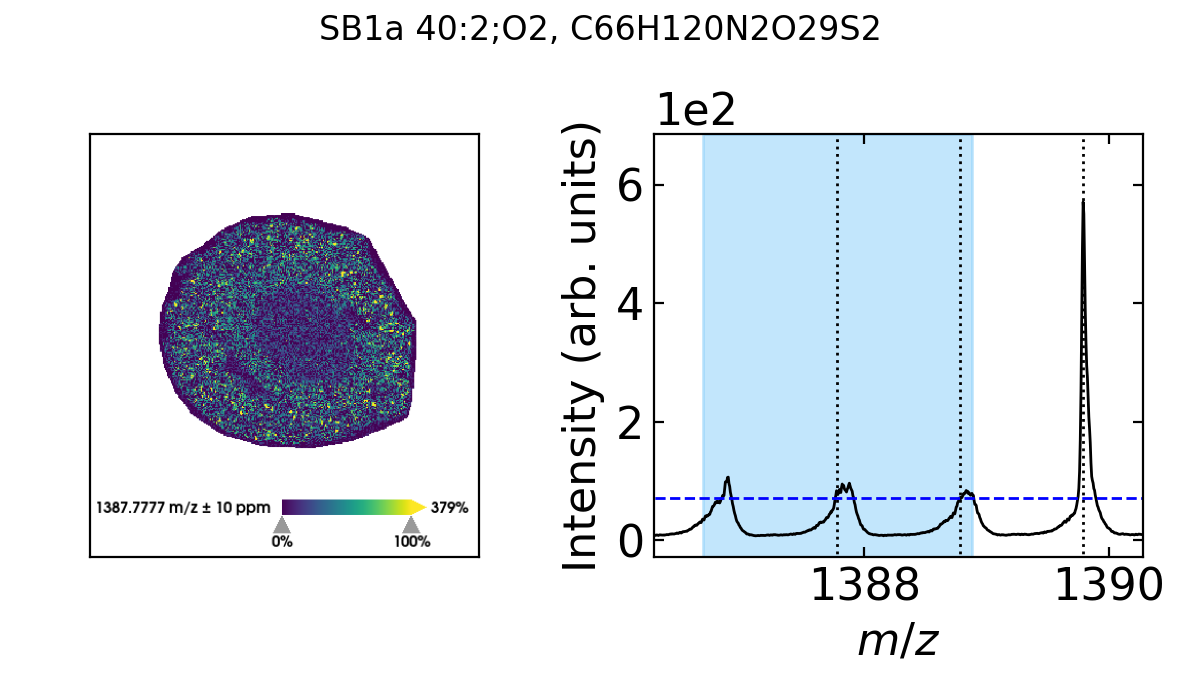

Supplement: Supplementary file 3 — Supplementary Data 1 [file 41467_2025_59839_MOESM3_ESM.zip › Suppl_Dataset_1_REV/qTOF_data3_slide1_python/1387.777727_qTOF_12w_1.png]

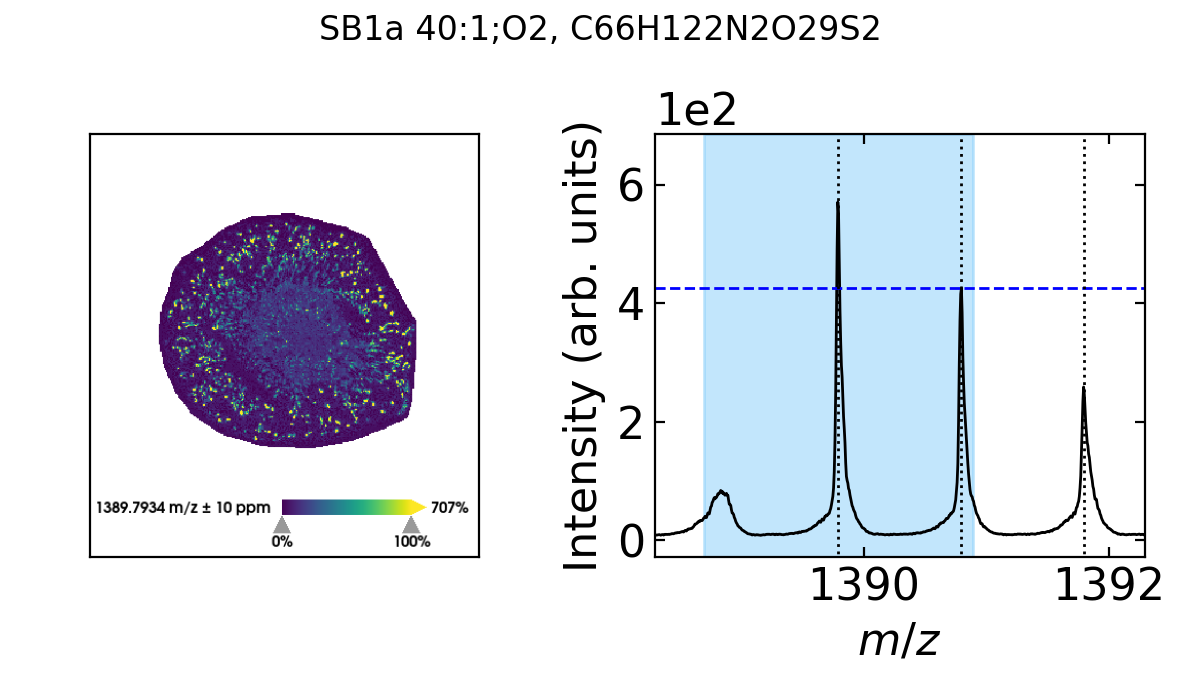

Supplement: Supplementary file 3 — Supplementary Data 1 [file 41467_2025_59839_MOESM3_ESM.zip › Suppl_Dataset_1_REV/qTOF_data3_slide1_python/1389.793377_qTOF_12w_1.png]

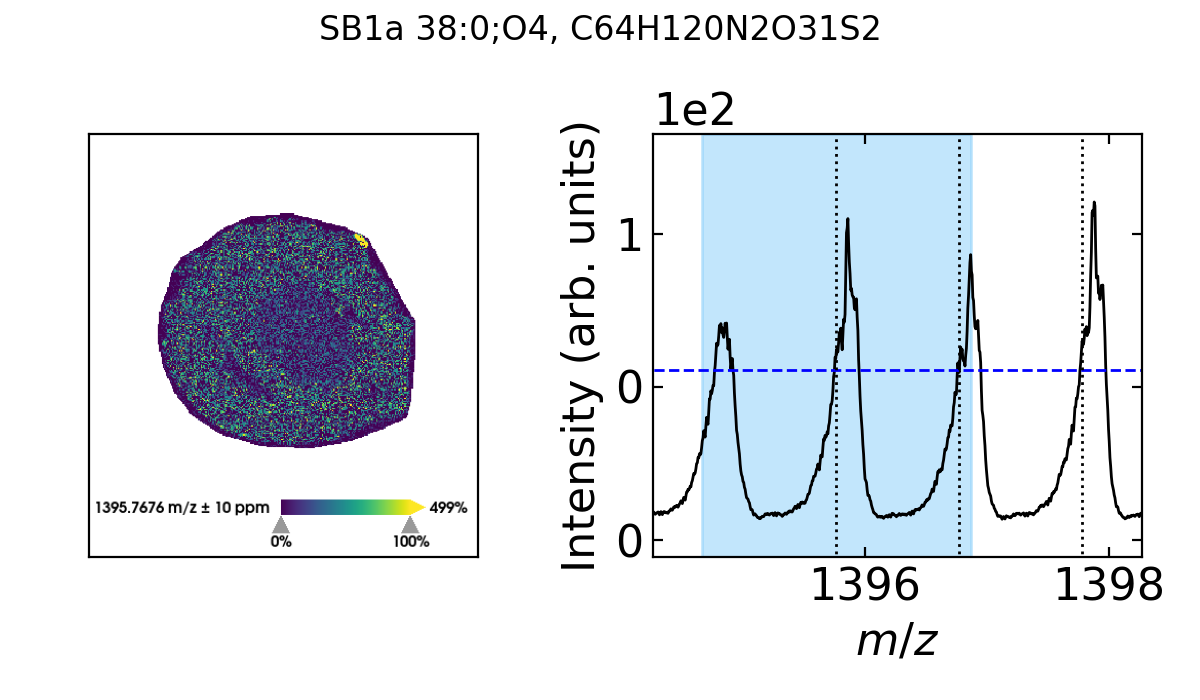

Supplement: Supplementary file 3 — Supplementary Data 1 [file 41467_2025_59839_MOESM3_ESM.zip › Suppl_Dataset_1_REV/qTOF_data3_slide1_python/1395.767556_qTOF_12w_1.png]

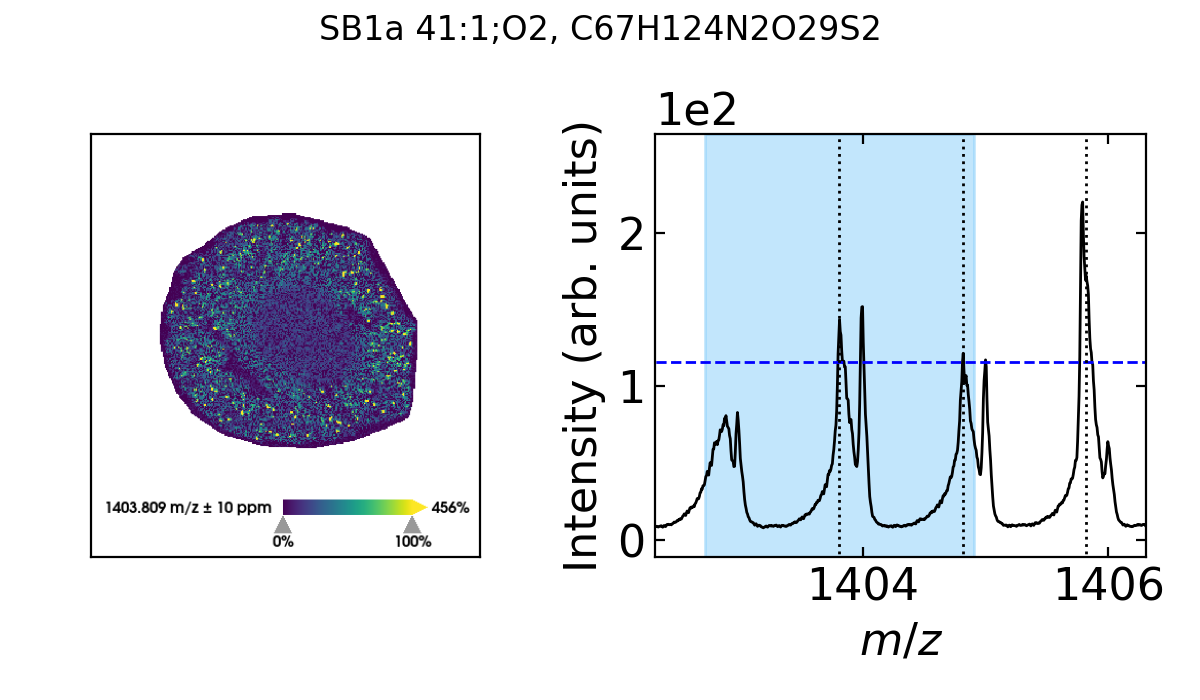

Supplement: Supplementary file 3 — Supplementary Data 1 [file 41467_2025_59839_MOESM3_ESM.zip › Suppl_Dataset_1_REV/qTOF_data3_slide1_python/1403.809027_qTOF_12w_1.png]

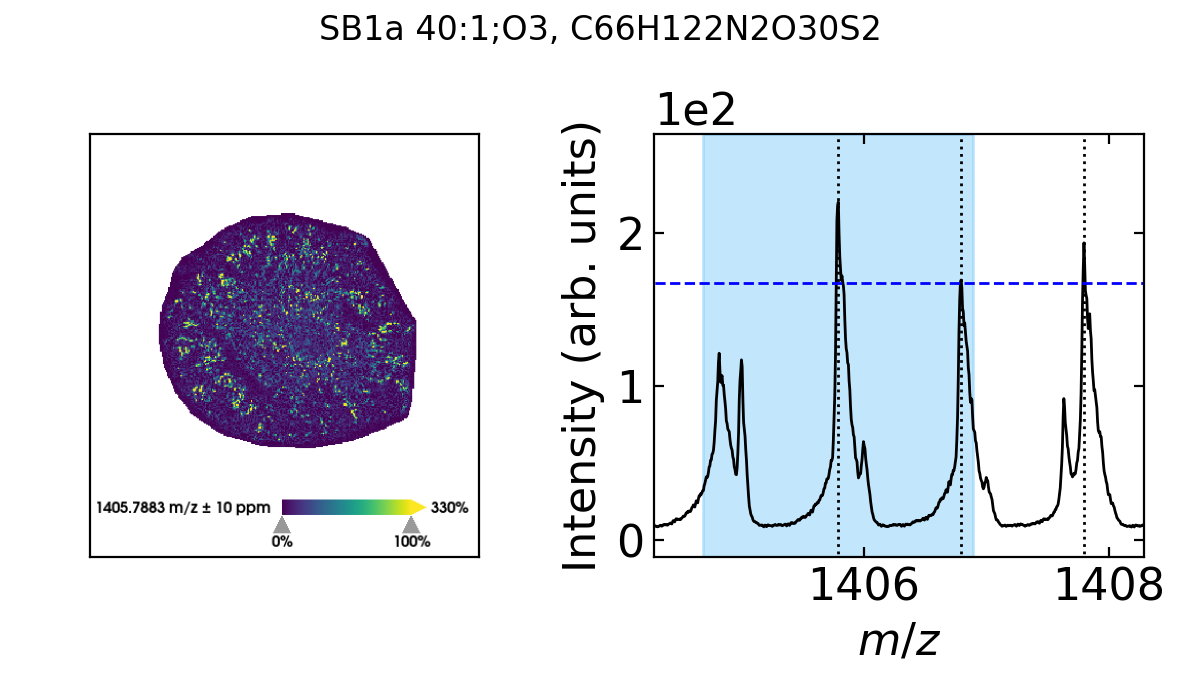

Supplement: Supplementary file 3 — Supplementary Data 1 [file 41467_2025_59839_MOESM3_ESM.zip › Suppl_Dataset_1_REV/qTOF_data3_slide1_python/1405.788292_qTOF_12w_1.png]

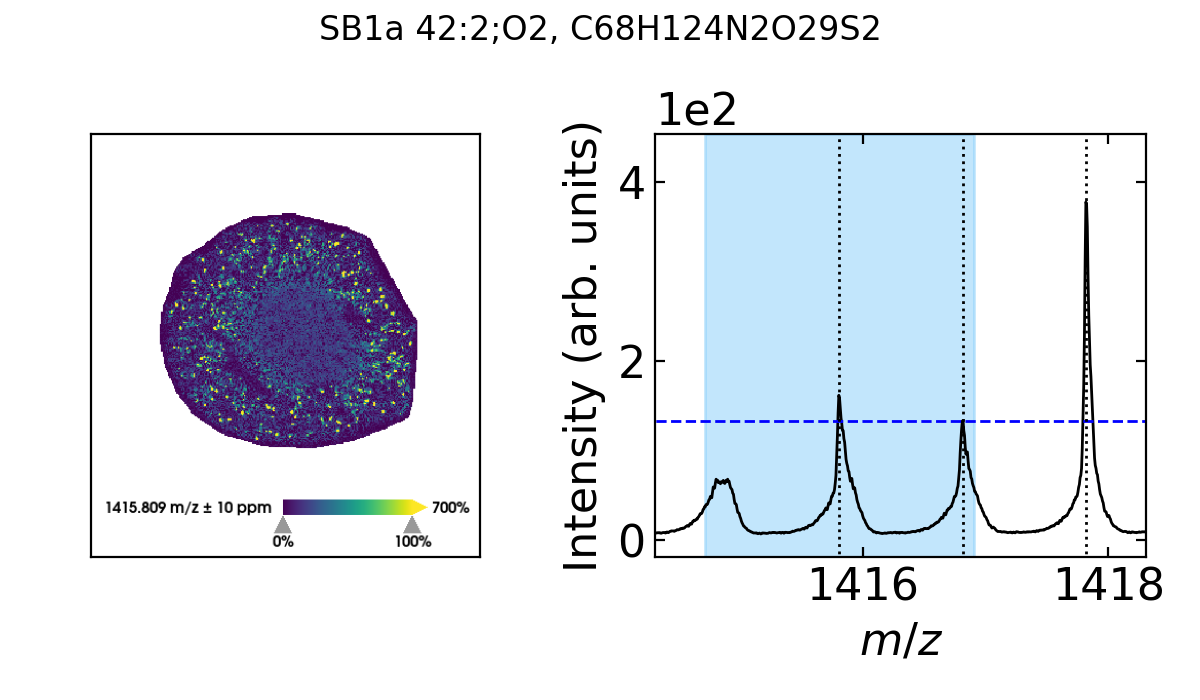

Supplement: Supplementary file 3 — Supplementary Data 1 [file 41467_2025_59839_MOESM3_ESM.zip › Suppl_Dataset_1_REV/qTOF_data3_slide1_python/1415.809027_qTOF_12w_1.png]

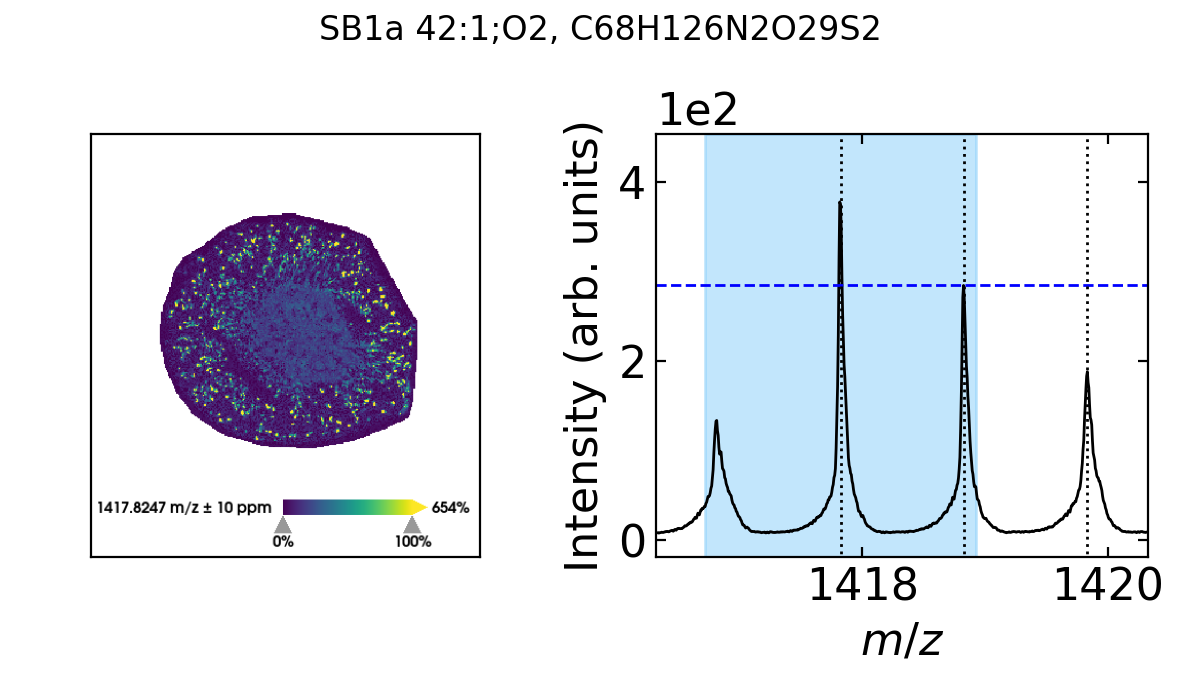

Supplement: Supplementary file 3 — Supplementary Data 1 [file 41467_2025_59839_MOESM3_ESM.zip › Suppl_Dataset_1_REV/qTOF_data3_slide1_python/1417.824677_qTOF_12w_1.png]

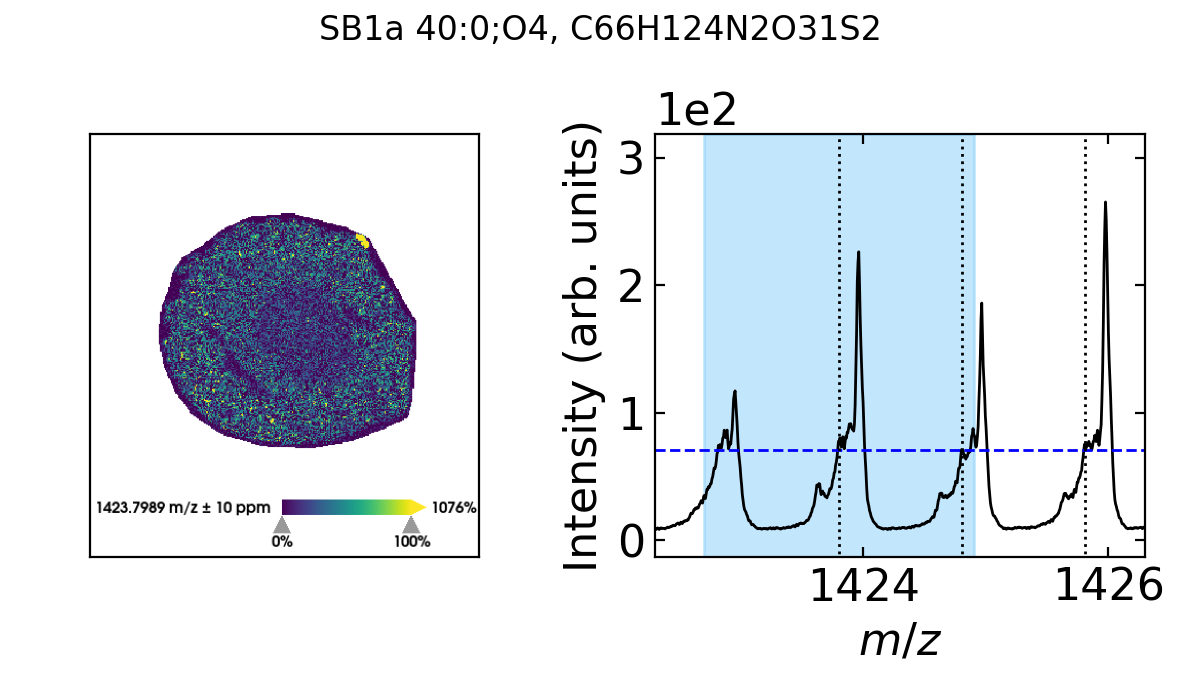

Supplement: Supplementary file 3 — Supplementary Data 1 [file 41467_2025_59839_MOESM3_ESM.zip › Suppl_Dataset_1_REV/qTOF_data3_slide1_python/1423.798857_qTOF_12w_1.png]

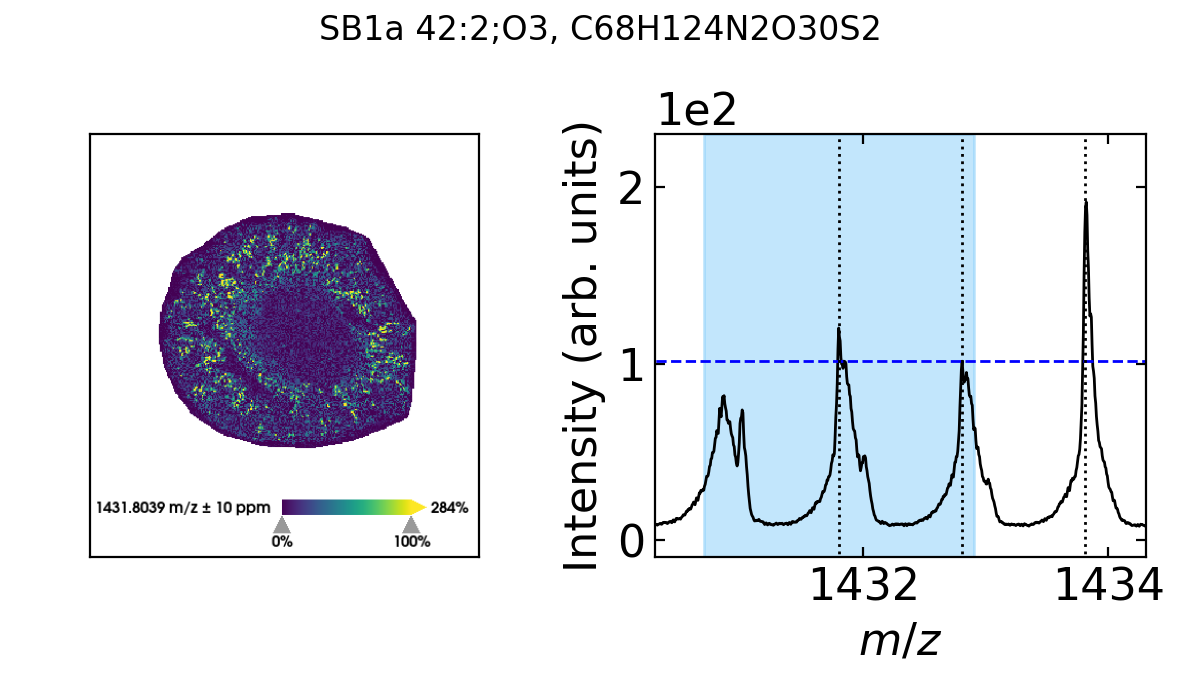

Supplement: Supplementary file 3 — Supplementary Data 1 [file 41467_2025_59839_MOESM3_ESM.zip › Suppl_Dataset_1_REV/qTOF_data3_slide1_python/1431.803942_qTOF_12w_1.png]

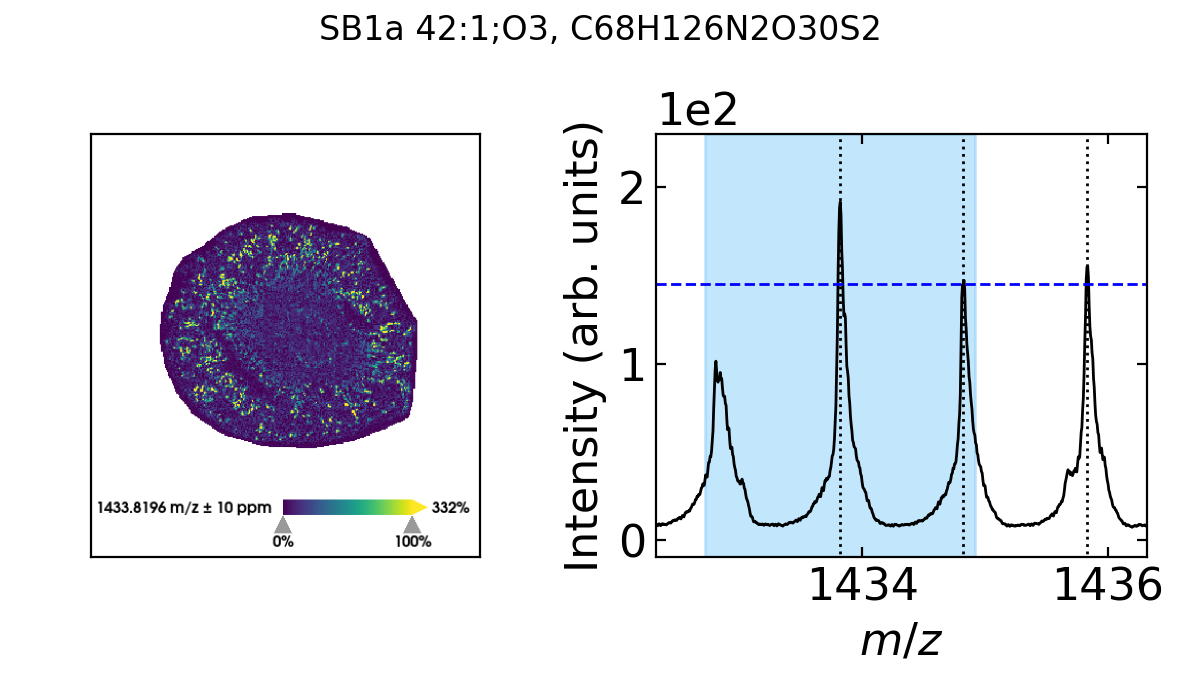

Supplement: Supplementary file 3 — Supplementary Data 1 [file 41467_2025_59839_MOESM3_ESM.zip › Suppl_Dataset_1_REV/qTOF_data3_slide1_python/1433.819592_qTOF_12w_1.png]

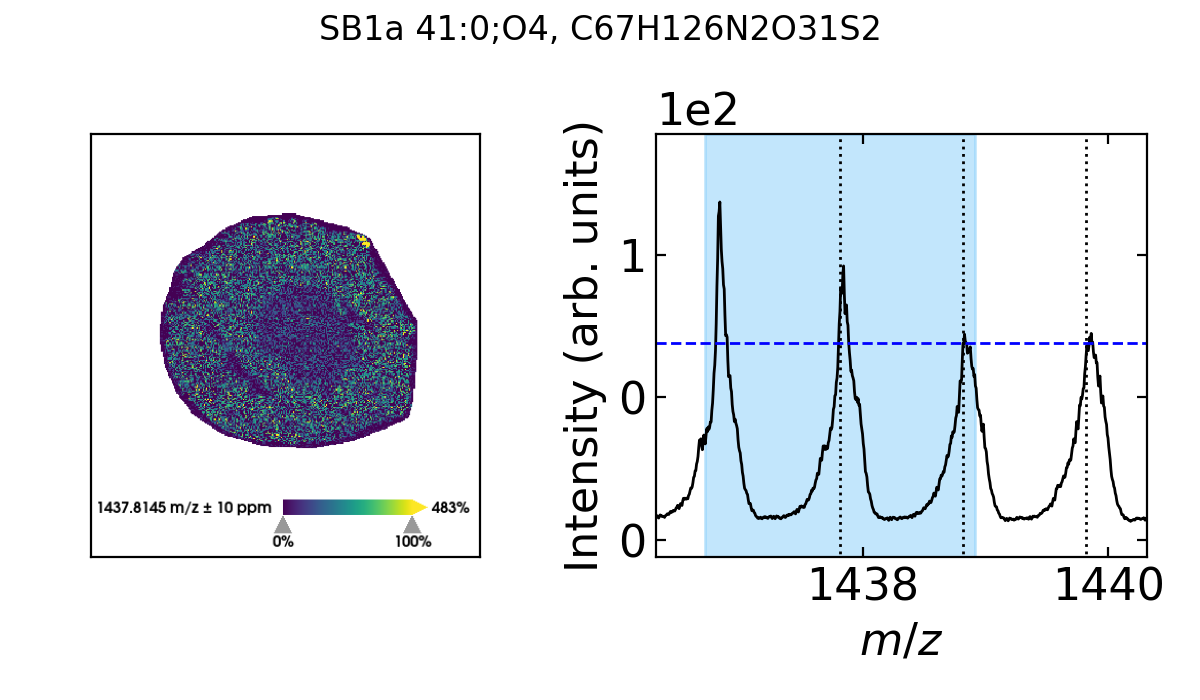

Supplement: Supplementary file 3 — Supplementary Data 1 [file 41467_2025_59839_MOESM3_ESM.zip › Suppl_Dataset_1_REV/qTOF_data3_slide1_python/1437.814507_qTOF_12w_1.png]

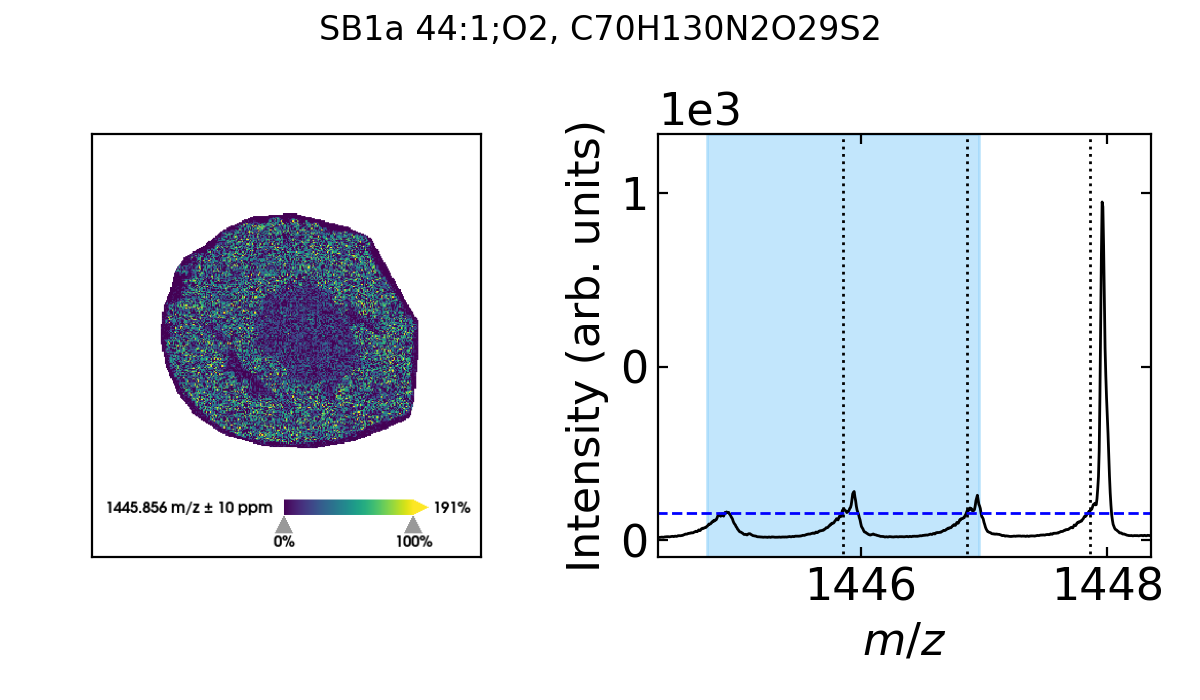

Supplement: Supplementary file 3 — Supplementary Data 1 [file 41467_2025_59839_MOESM3_ESM.zip › Suppl_Dataset_1_REV/qTOF_data3_slide1_python/1445.855977_qTOF_12w_1.png]

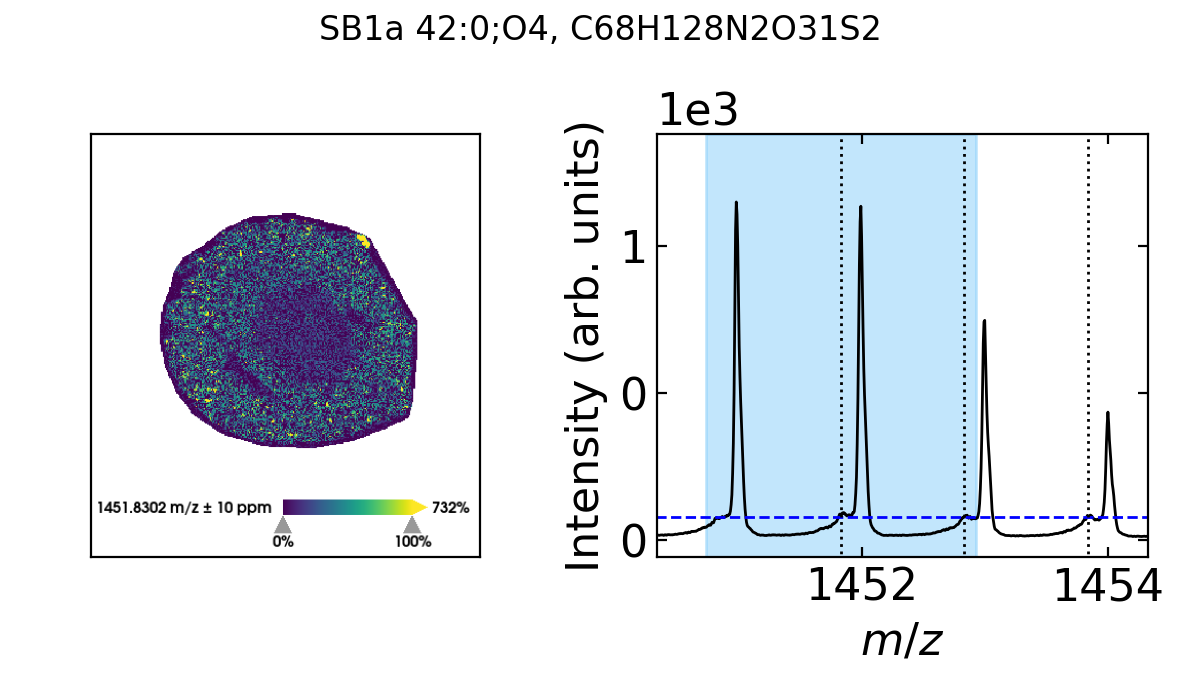

Supplement: Supplementary file 3 — Supplementary Data 1 [file 41467_2025_59839_MOESM3_ESM.zip › Suppl_Dataset_1_REV/qTOF_data3_slide1_python/1451.830157_qTOF_12w_1.png]

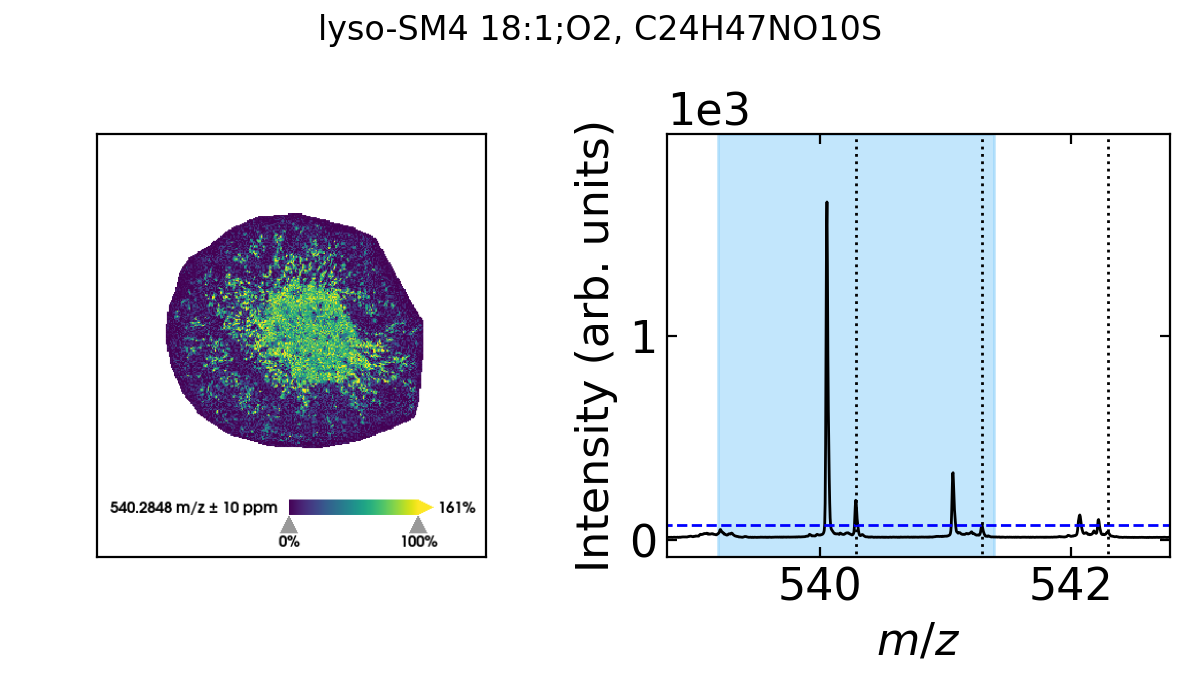

Supplement: Supplementary file 3 — Supplementary Data 1 [file 41467_2025_59839_MOESM3_ESM.zip › Suppl_Dataset_1_REV/qTOF_data3_slide1_python/540.284791_qTOF_12w_1.png]

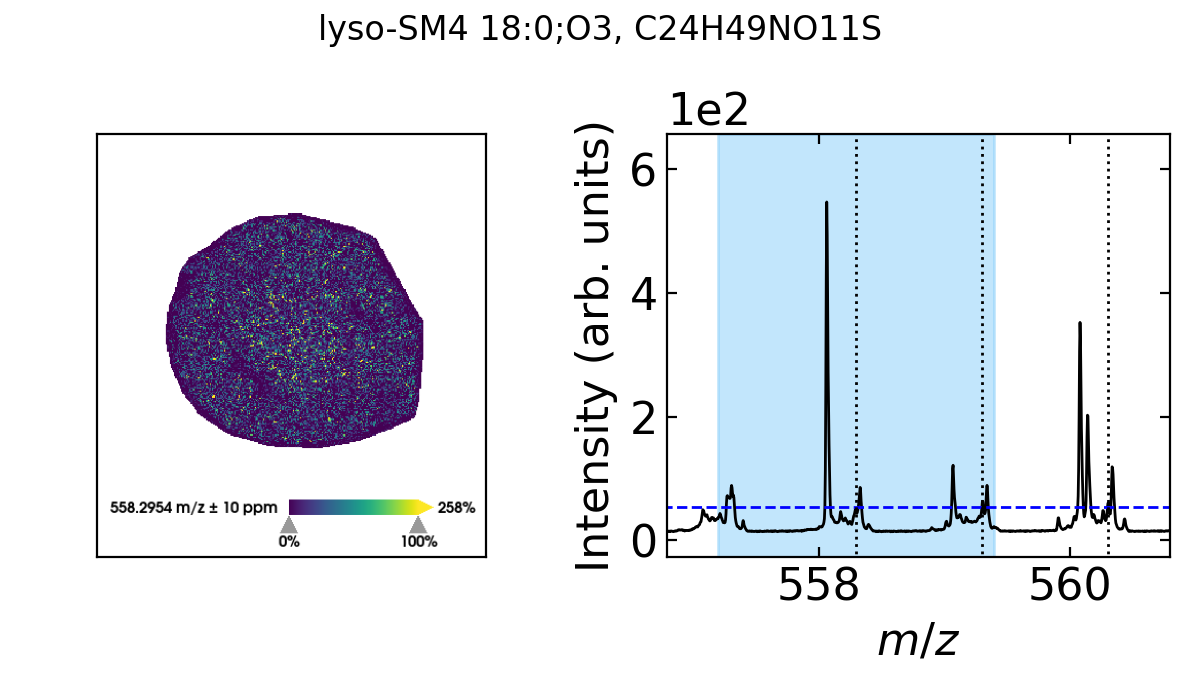

Supplement: Supplementary file 3 — Supplementary Data 1 [file 41467_2025_59839_MOESM3_ESM.zip › Suppl_Dataset_1_REV/qTOF_data3_slide1_python/558.295356_qTOF_12w_1.png]

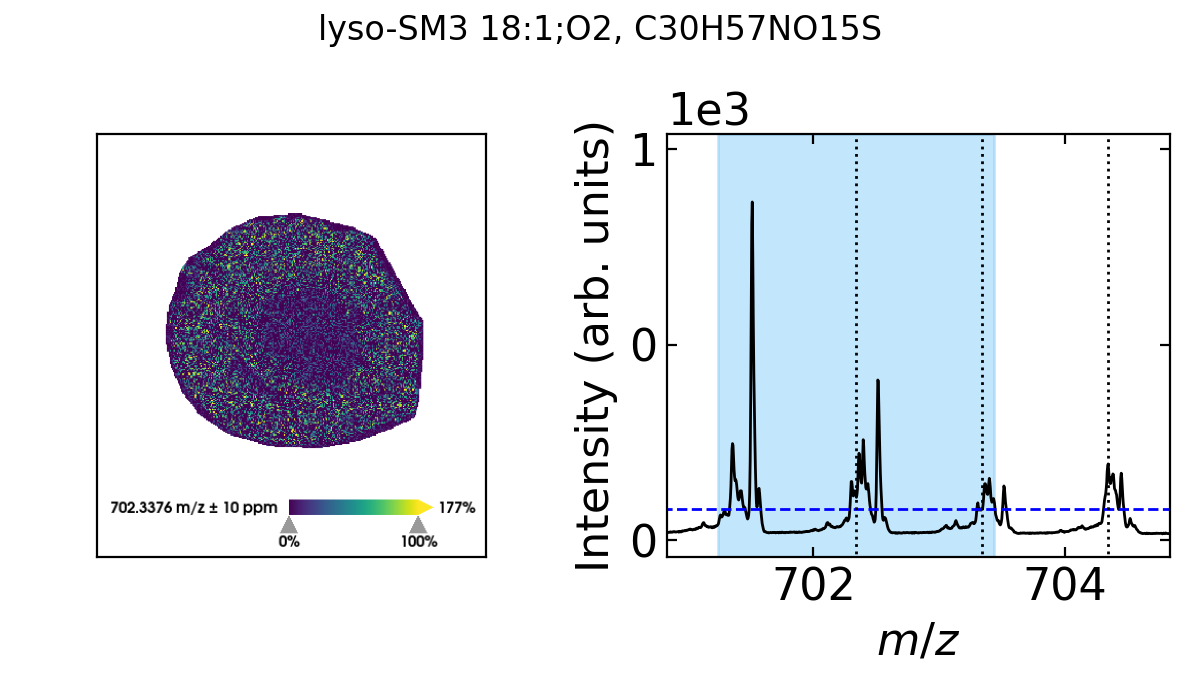

Supplement: Supplementary file 3 — Supplementary Data 1 [file 41467_2025_59839_MOESM3_ESM.zip › Suppl_Dataset_1_REV/qTOF_data3_slide1_python/702.337615_qTOF_12w_1.png]

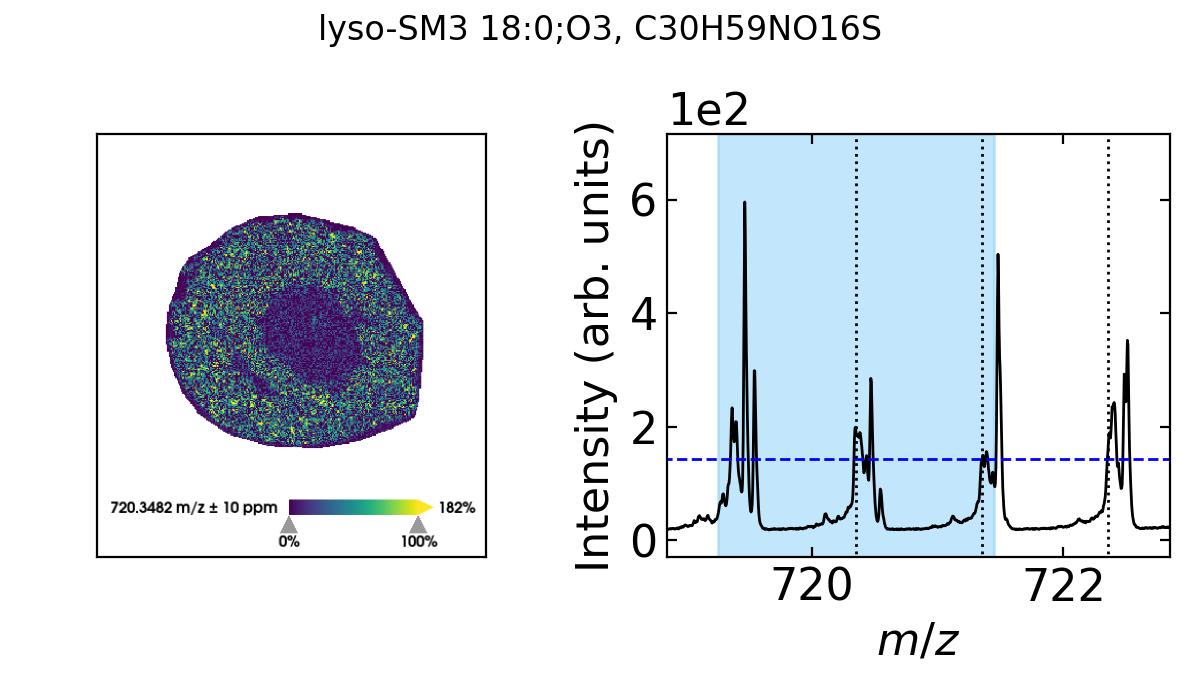

Supplement: Supplementary file 3 — Supplementary Data 1 [file 41467_2025_59839_MOESM3_ESM.zip › Suppl_Dataset_1_REV/qTOF_data3_slide1_python/720.34818_qTOF_12w_1.png]

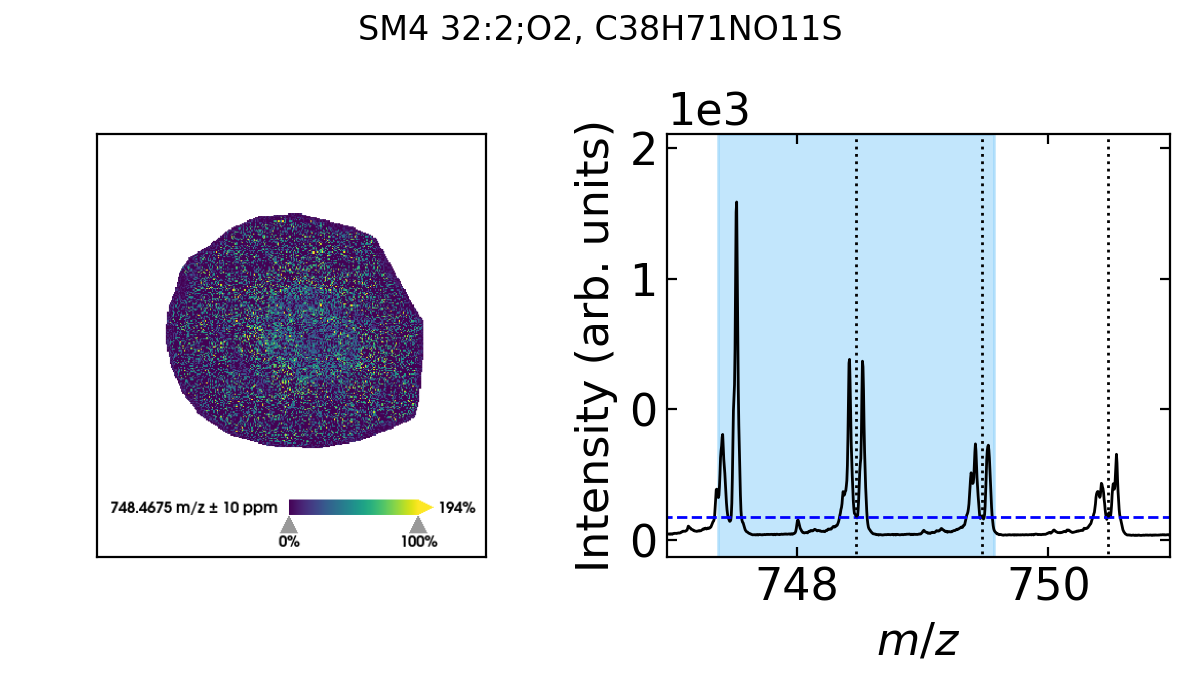

Supplement: Supplementary file 3 — Supplementary Data 1 [file 41467_2025_59839_MOESM3_ESM.zip › Suppl_Dataset_1_REV/qTOF_data3_slide1_python/748.467507_qTOF_12w_1.png]

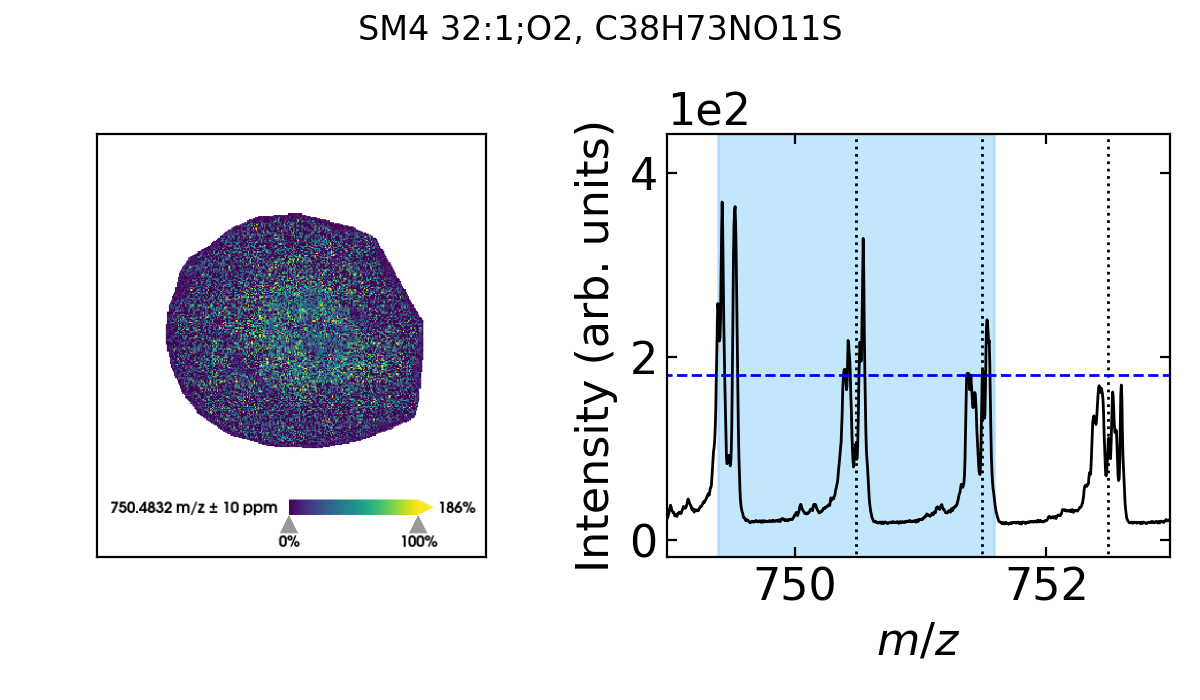

Supplement: Supplementary file 3 — Supplementary Data 1 [file 41467_2025_59839_MOESM3_ESM.zip › Suppl_Dataset_1_REV/qTOF_data3_slide1_python/750.483157_qTOF_12w_1.png]

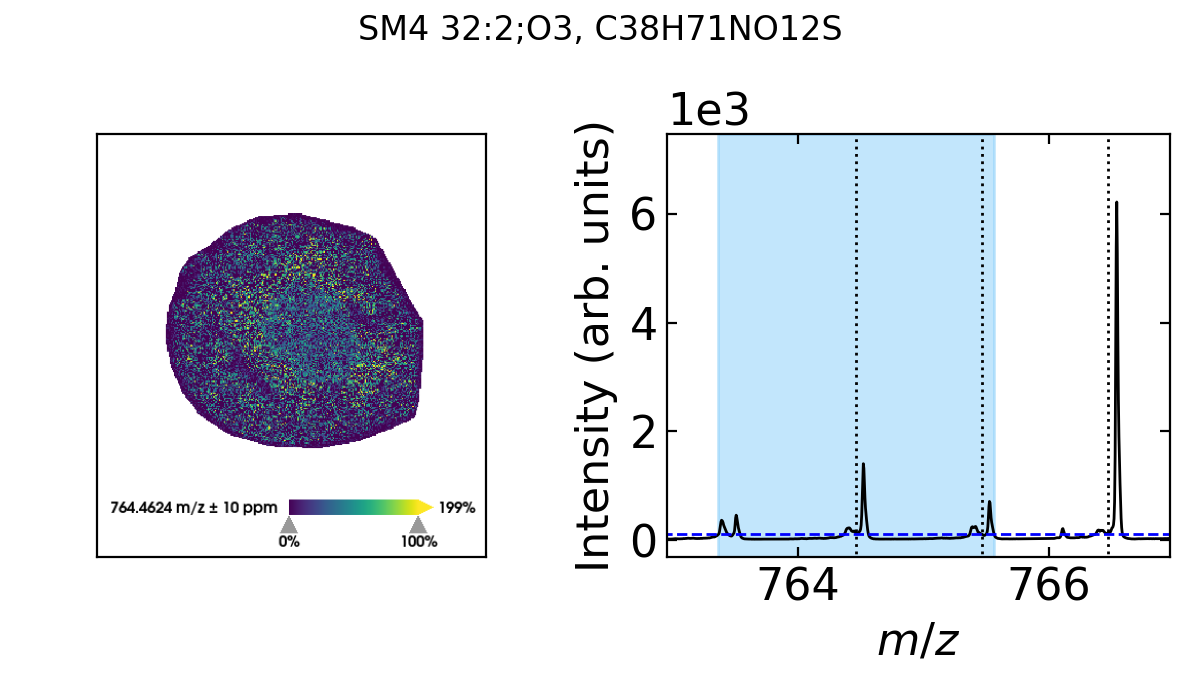

Supplement: Supplementary file 3 — Supplementary Data 1 [file 41467_2025_59839_MOESM3_ESM.zip › Suppl_Dataset_1_REV/qTOF_data3_slide1_python/764.462422_qTOF_12w_1.png]

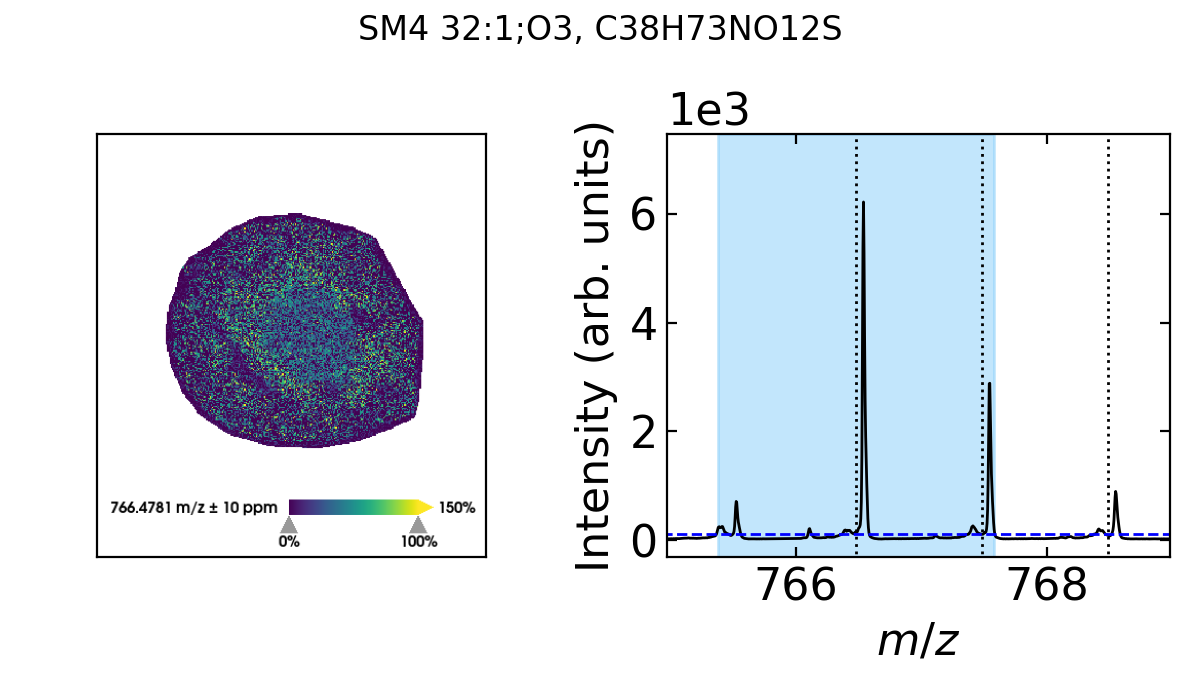

Supplement: Supplementary file 3 — Supplementary Data 1 [file 41467_2025_59839_MOESM3_ESM.zip › Suppl_Dataset_1_REV/qTOF_data3_slide1_python/766.478072_qTOF_12w_1.png]

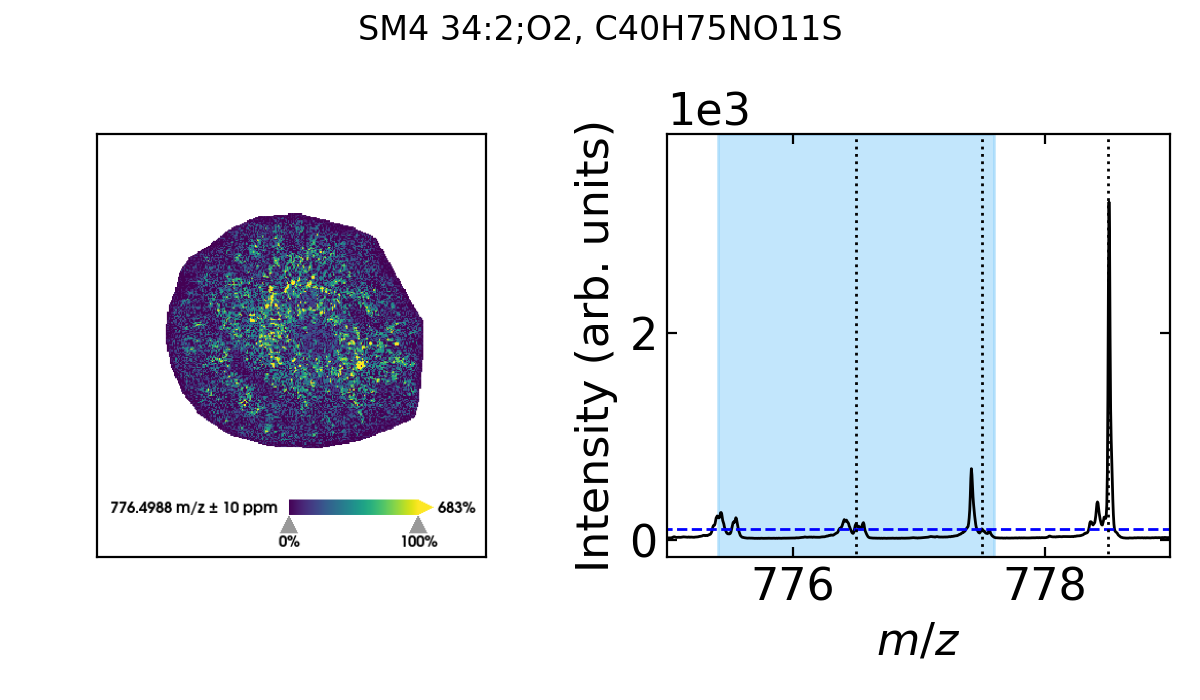

Supplement: Supplementary file 3 — Supplementary Data 1 [file 41467_2025_59839_MOESM3_ESM.zip › Suppl_Dataset_1_REV/qTOF_data3_slide1_python/776.498807_qTOF_12w_1.png]

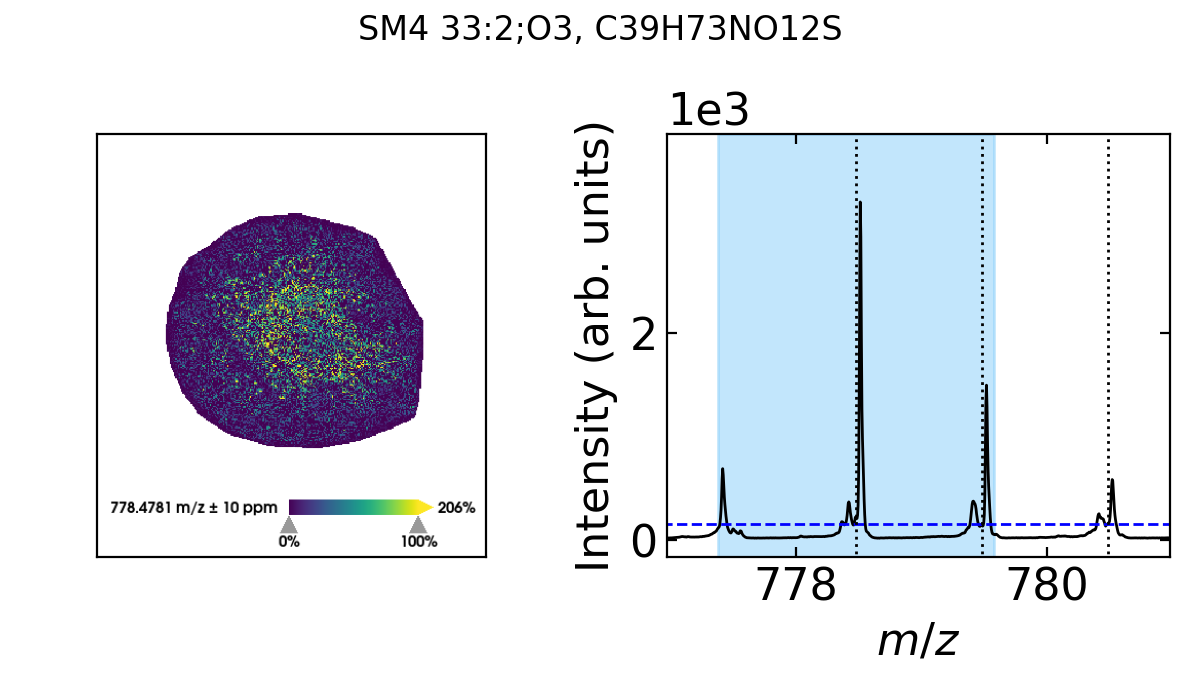

Supplement: Supplementary file 3 — Supplementary Data 1 [file 41467_2025_59839_MOESM3_ESM.zip › Suppl_Dataset_1_REV/qTOF_data3_slide1_python/778.478072_qTOF_12w_1.png]

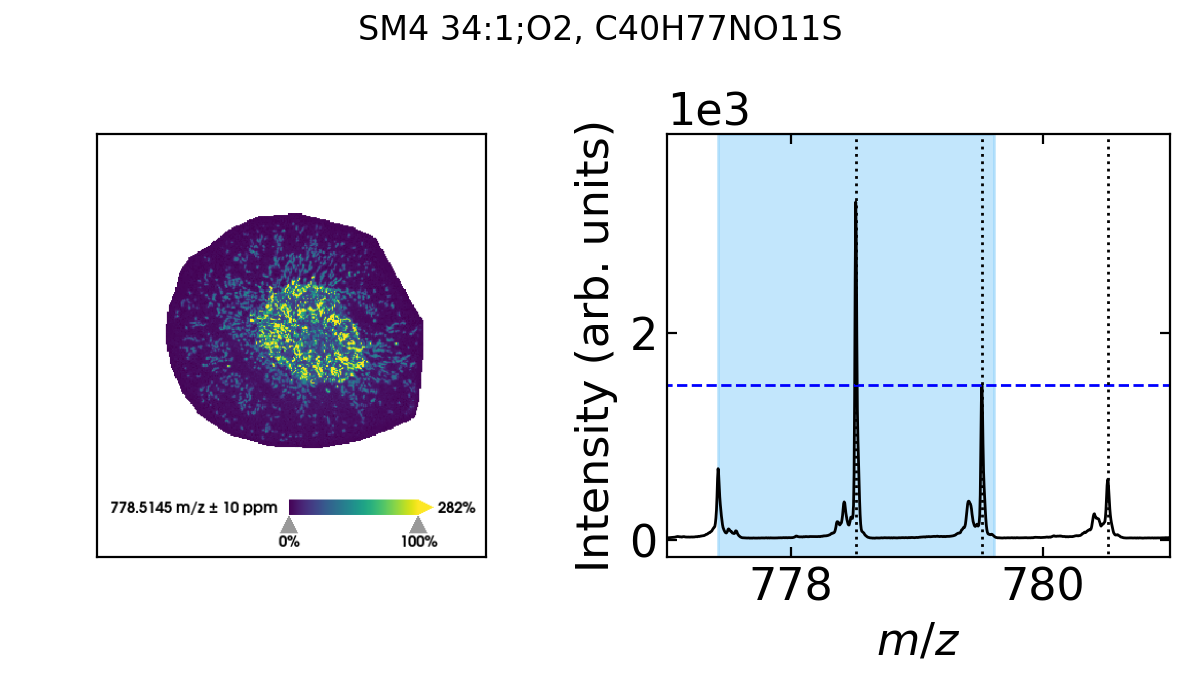

Supplement: Supplementary file 3 — Supplementary Data 1 [file 41467_2025_59839_MOESM3_ESM.zip › Suppl_Dataset_1_REV/qTOF_data3_slide1_python/778.514457_qTOF_12w_1.png]

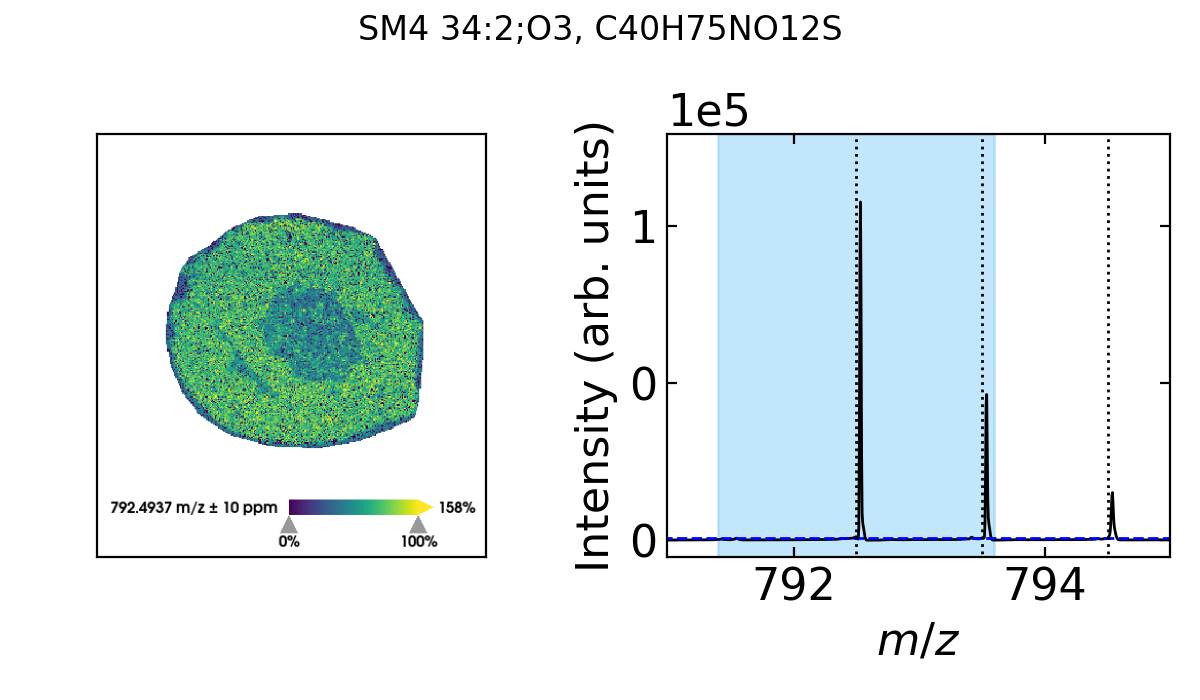

Supplement: Supplementary file 3 — Supplementary Data 1 [file 41467_2025_59839_MOESM3_ESM.zip › Suppl_Dataset_1_REV/qTOF_data3_slide1_python/792.493722_qTOF_12w_1.png]

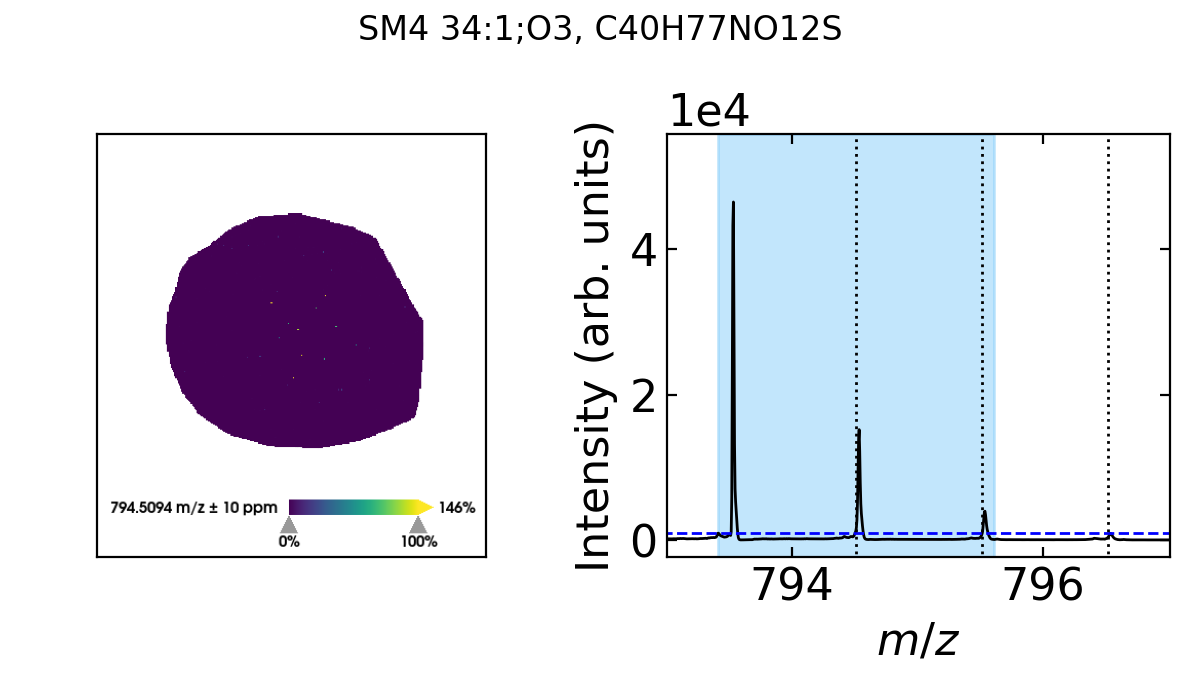

Supplement: Supplementary file 3 — Supplementary Data 1 [file 41467_2025_59839_MOESM3_ESM.zip › Suppl_Dataset_1_REV/qTOF_data3_slide1_python/794.509372_qTOF_12w_1.png]

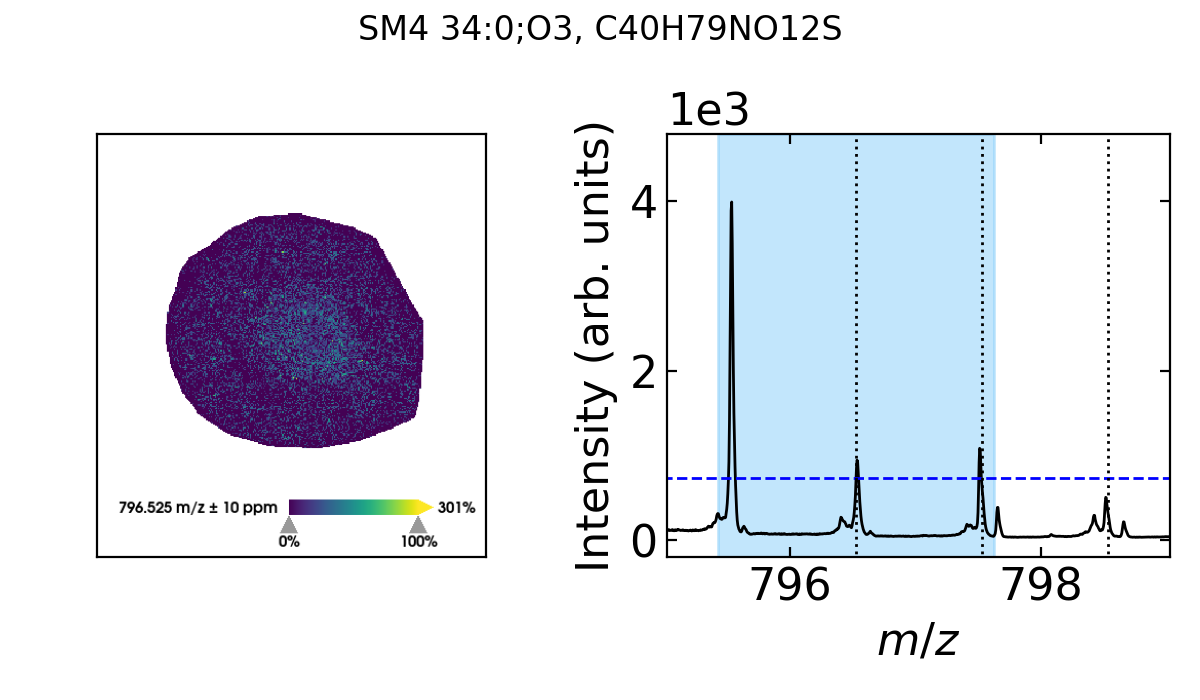

Supplement: Supplementary file 3 — Supplementary Data 1 [file 41467_2025_59839_MOESM3_ESM.zip › Suppl_Dataset_1_REV/qTOF_data3_slide1_python/796.525022_qTOF_12w_1.png]

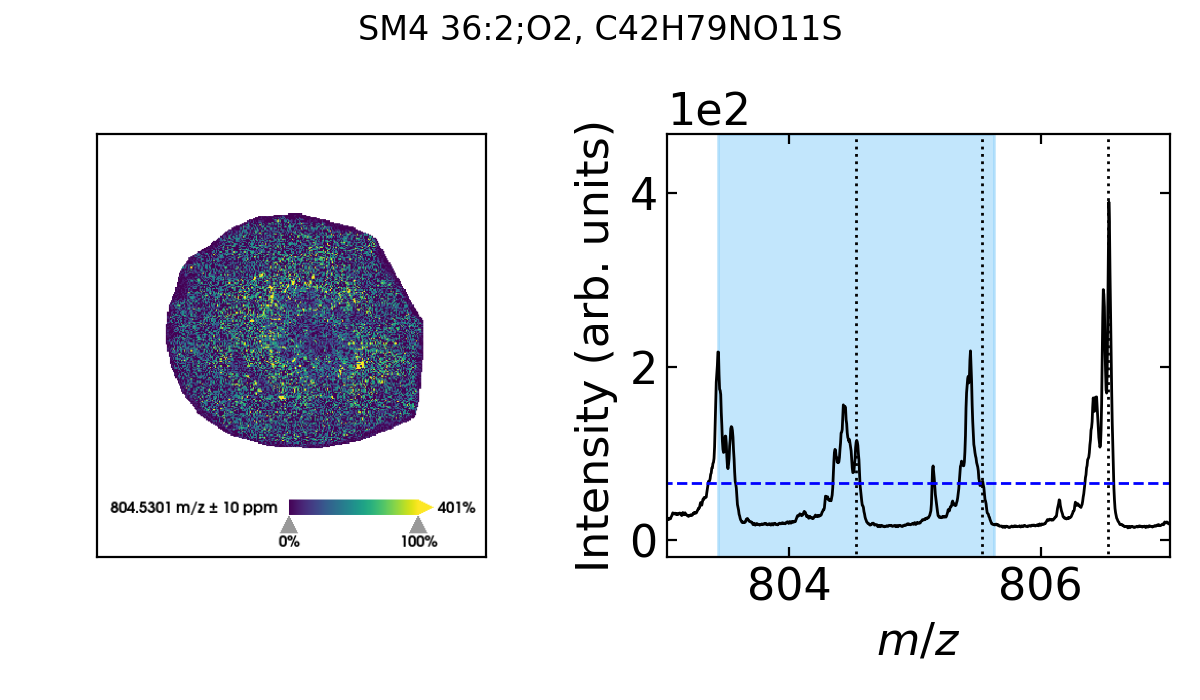

Supplement: Supplementary file 3 — Supplementary Data 1 [file 41467_2025_59839_MOESM3_ESM.zip › Suppl_Dataset_1_REV/qTOF_data3_slide1_python/804.530107_qTOF_12w_1.png]

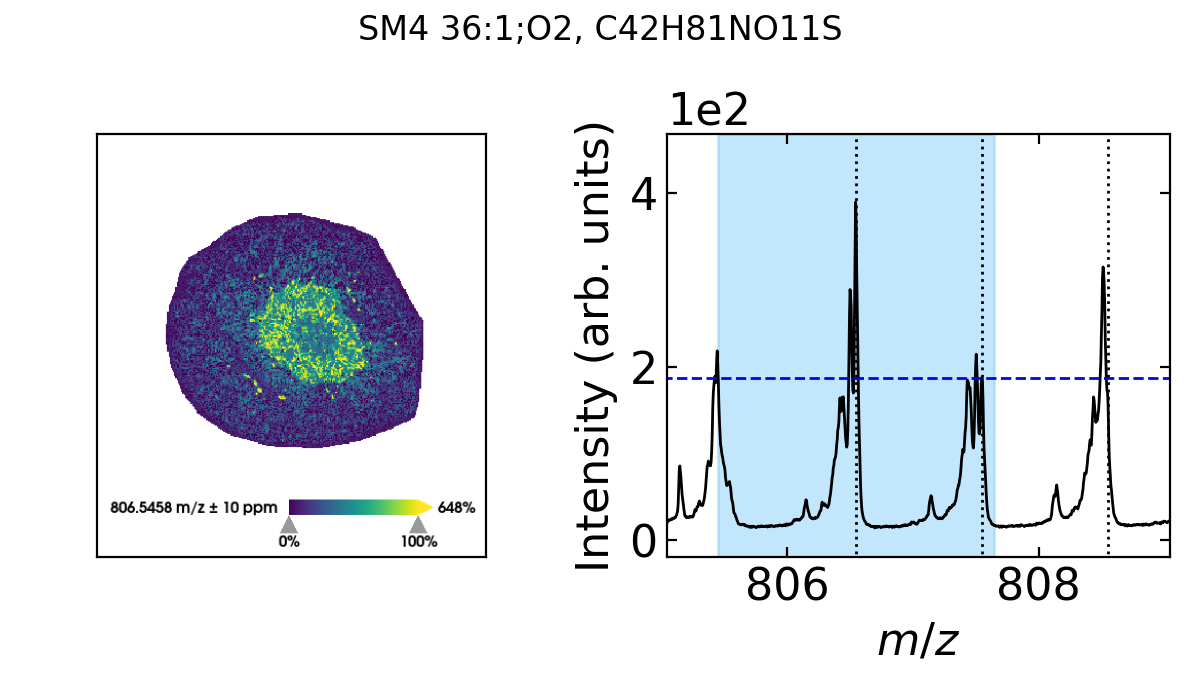

Supplement: Supplementary file 3 — Supplementary Data 1 [file 41467_2025_59839_MOESM3_ESM.zip › Suppl_Dataset_1_REV/qTOF_data3_slide1_python/806.545757_qTOF_12w_1.png]

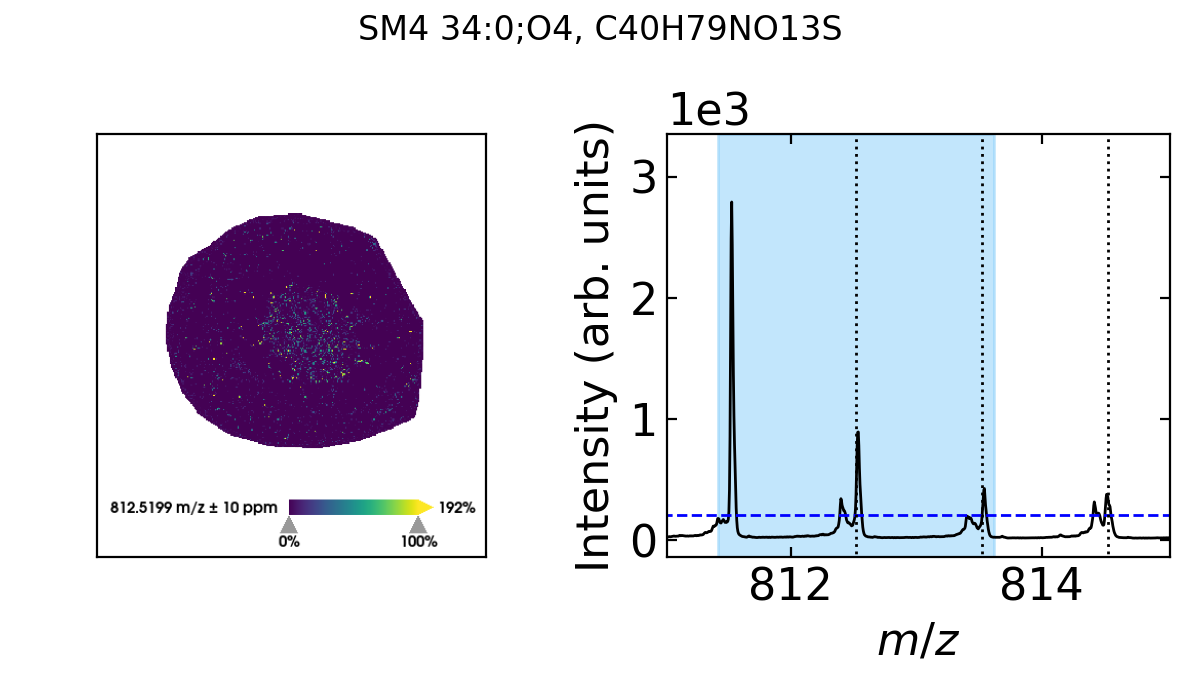

Supplement: Supplementary file 3 — Supplementary Data 1 [file 41467_2025_59839_MOESM3_ESM.zip › Suppl_Dataset_1_REV/qTOF_data3_slide1_python/812.519936_qTOF_12w_1.png]

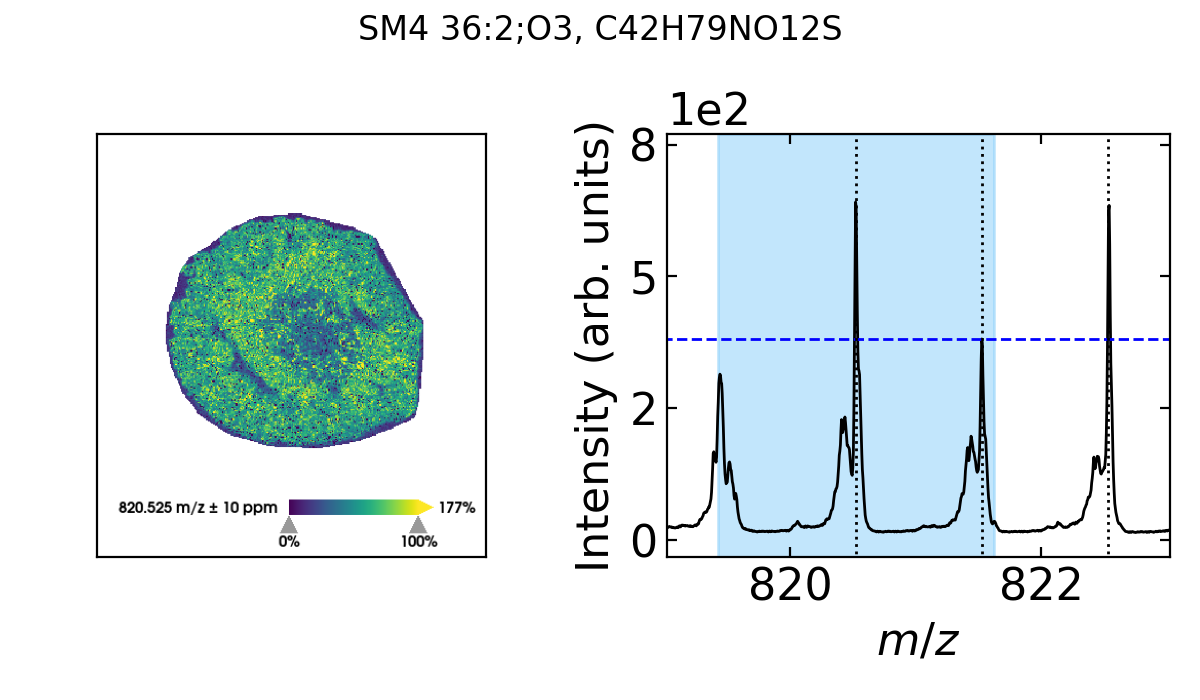

Supplement: Supplementary file 3 — Supplementary Data 1 [file 41467_2025_59839_MOESM3_ESM.zip › Suppl_Dataset_1_REV/qTOF_data3_slide1_python/820.525022_qTOF_12w_1.png]

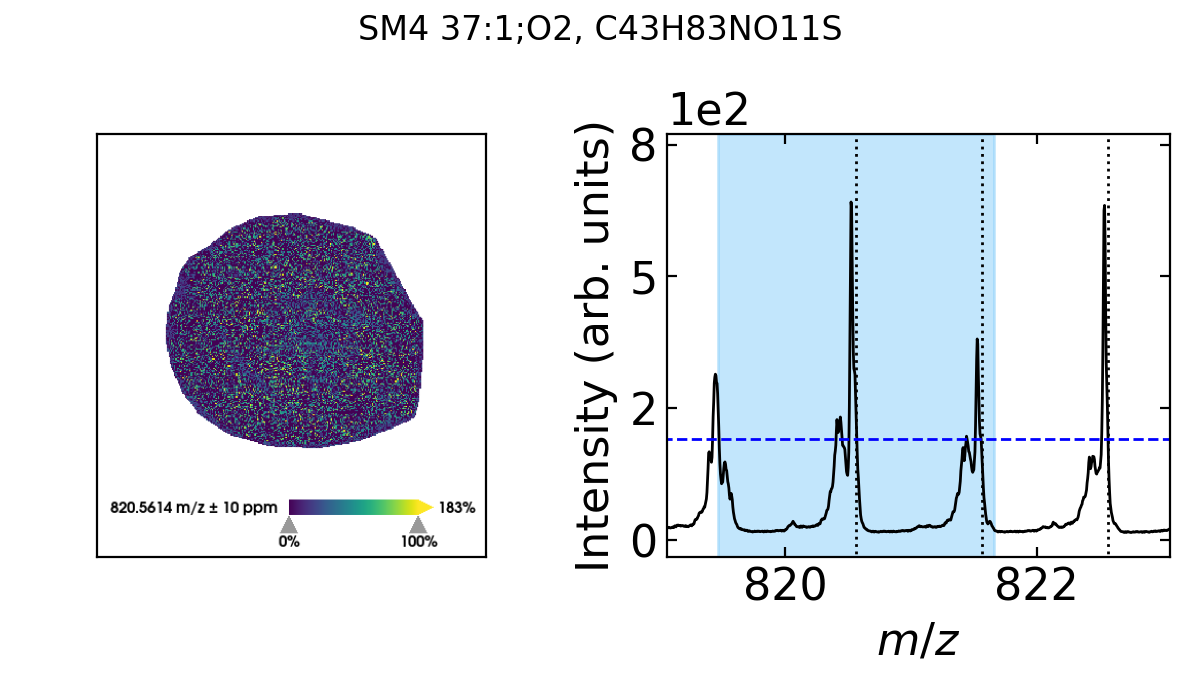

Supplement: Supplementary file 3 — Supplementary Data 1 [file 41467_2025_59839_MOESM3_ESM.zip › Suppl_Dataset_1_REV/qTOF_data3_slide1_python/820.561407_qTOF_12w_1.png]
