# Supplementary figures and images for: Deep MALDI-MS spatial omics guided by quantum cascade laser mid-infrared imaging microscopy (part 1 of 2)
Source: Nat Commun. 2025 May 22;16:4759. doi: 10.1038/s41467-025-59839-3 (PMC12098849; doi:10.1038/s41467-025-59839-3)

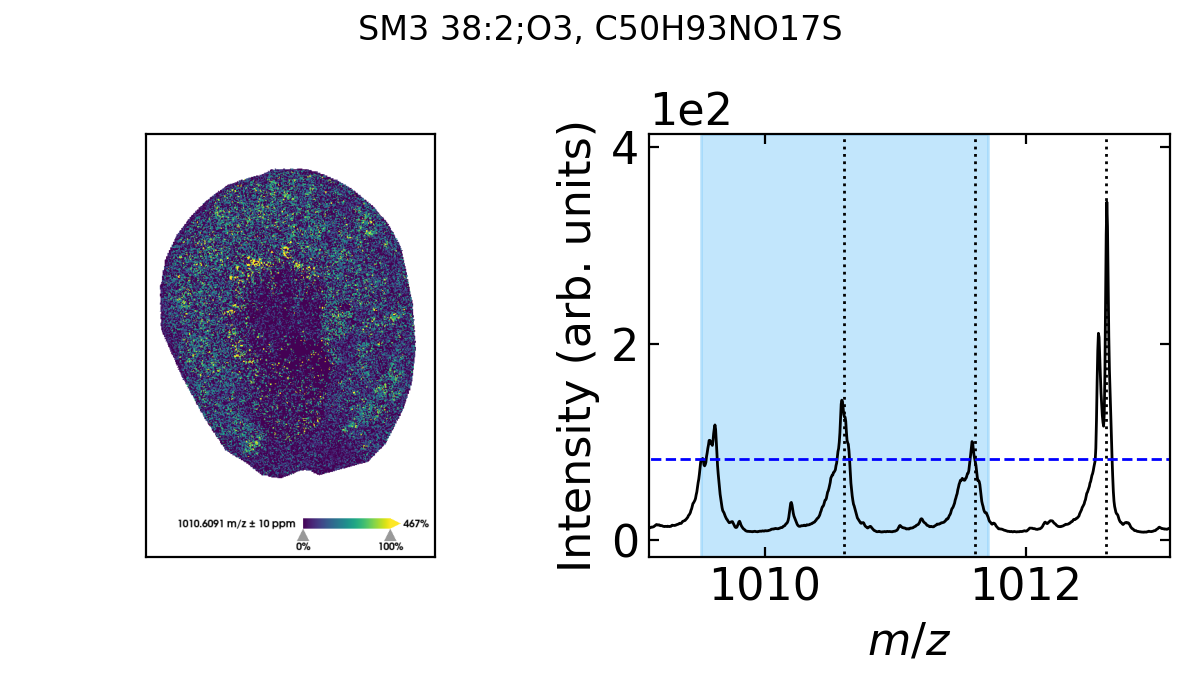

Supplement: Supplementary file 3 — Supplementary Data 1 [file 41467_2025_59839_MOESM3_ESM.zip › Suppl_Dataset_1_REV/qTOF_data1_slide1_python/1010.609146_qTOF_60w_1.png]

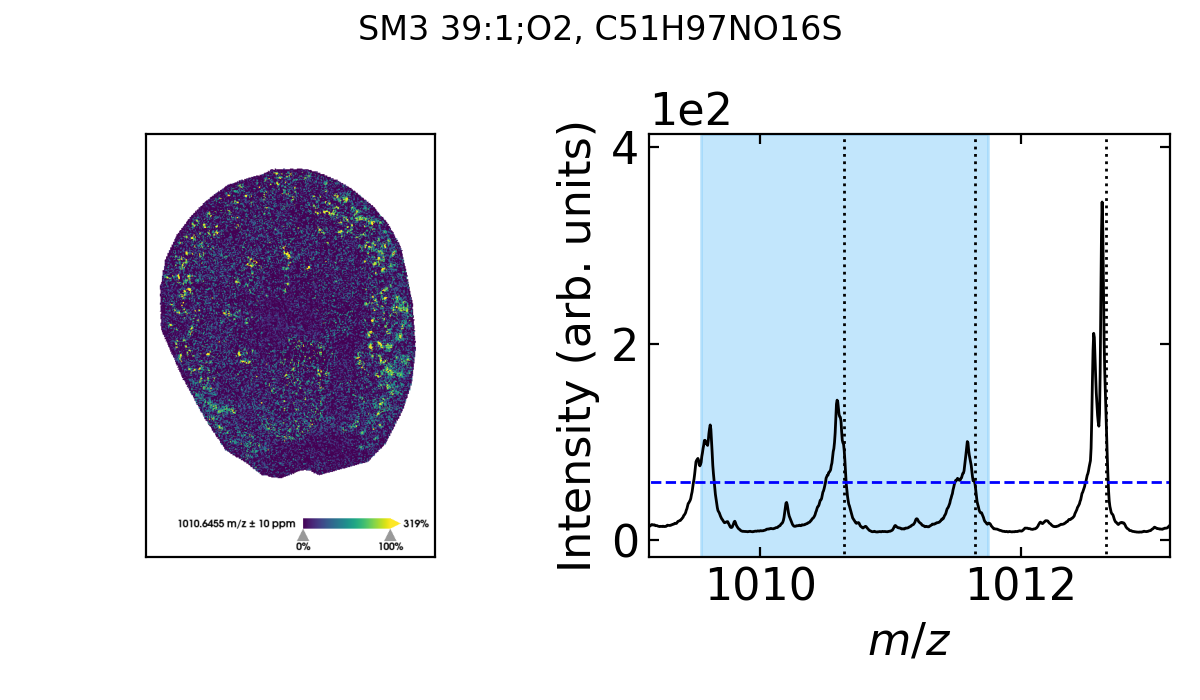

Supplement: Supplementary file 3 — Supplementary Data 1 [file 41467_2025_59839_MOESM3_ESM.zip › Suppl_Dataset_1_REV/qTOF_data1_slide1_python/1010.645531_qTOF_60w_1.png]

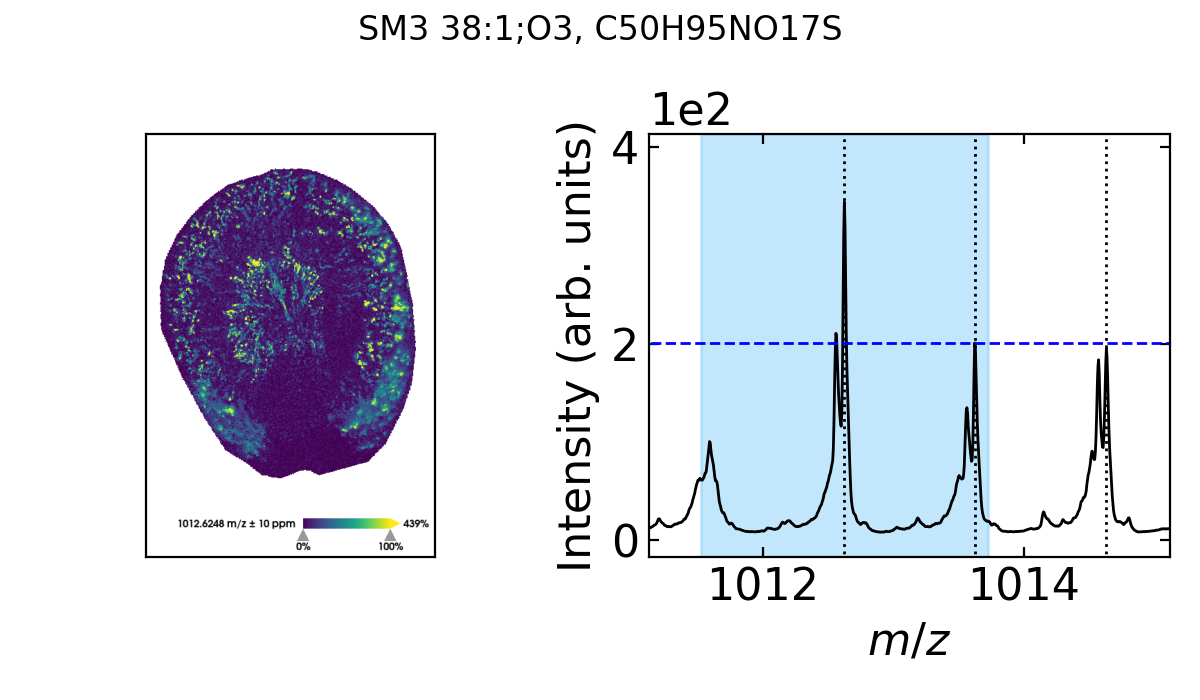

Supplement: Supplementary file 3 — Supplementary Data 1 [file 41467_2025_59839_MOESM3_ESM.zip › Suppl_Dataset_1_REV/qTOF_data1_slide1_python/1012.624796_qTOF_60w_1.png]

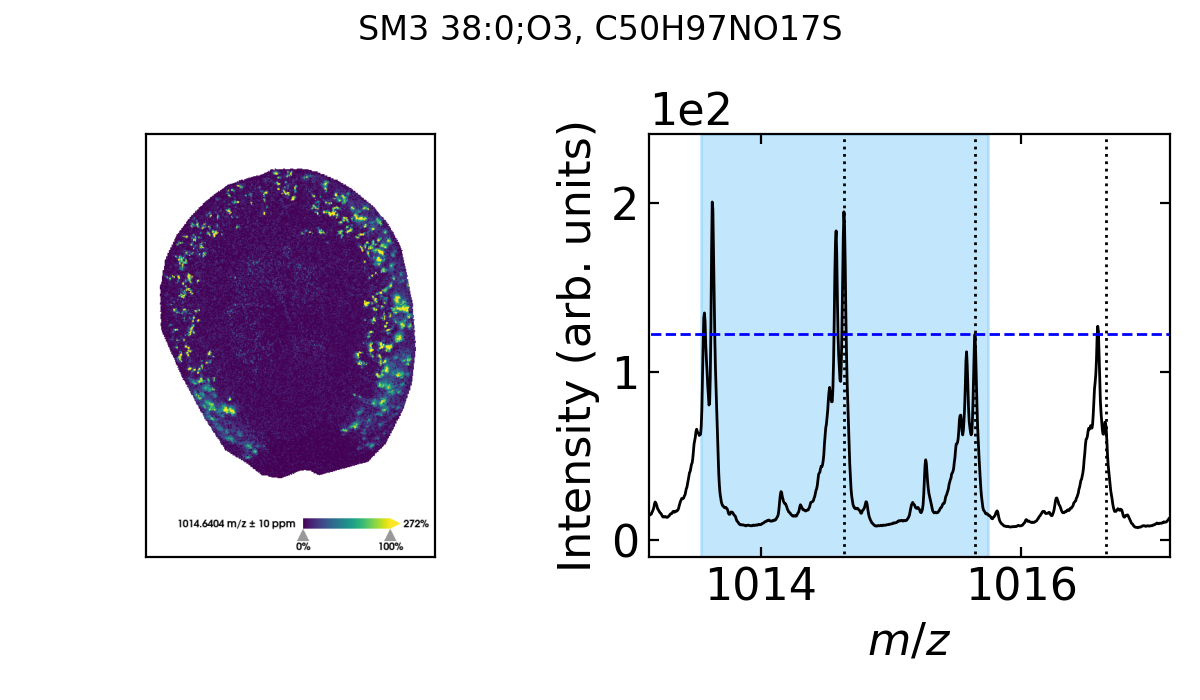

Supplement: Supplementary file 3 — Supplementary Data 1 [file 41467_2025_59839_MOESM3_ESM.zip › Suppl_Dataset_1_REV/qTOF_data1_slide1_python/1014.640446_qTOF_60w_1.png]

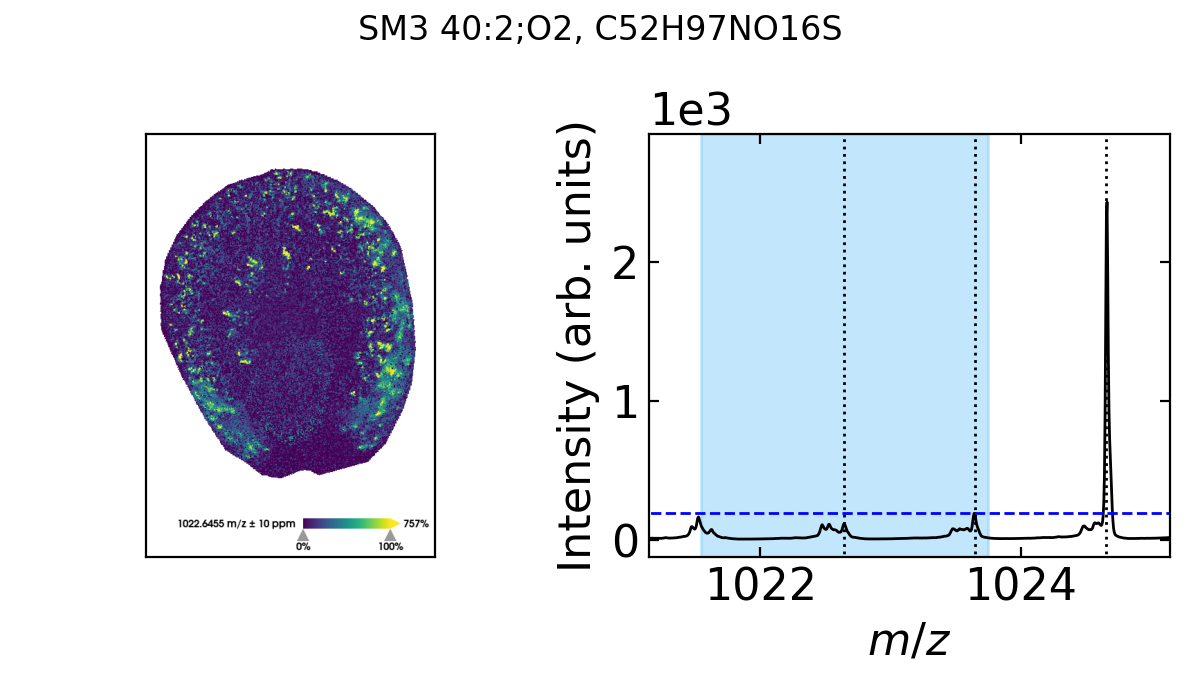

Supplement: Supplementary file 3 — Supplementary Data 1 [file 41467_2025_59839_MOESM3_ESM.zip › Suppl_Dataset_1_REV/qTOF_data1_slide1_python/1022.645531_qTOF_60w_1.png]

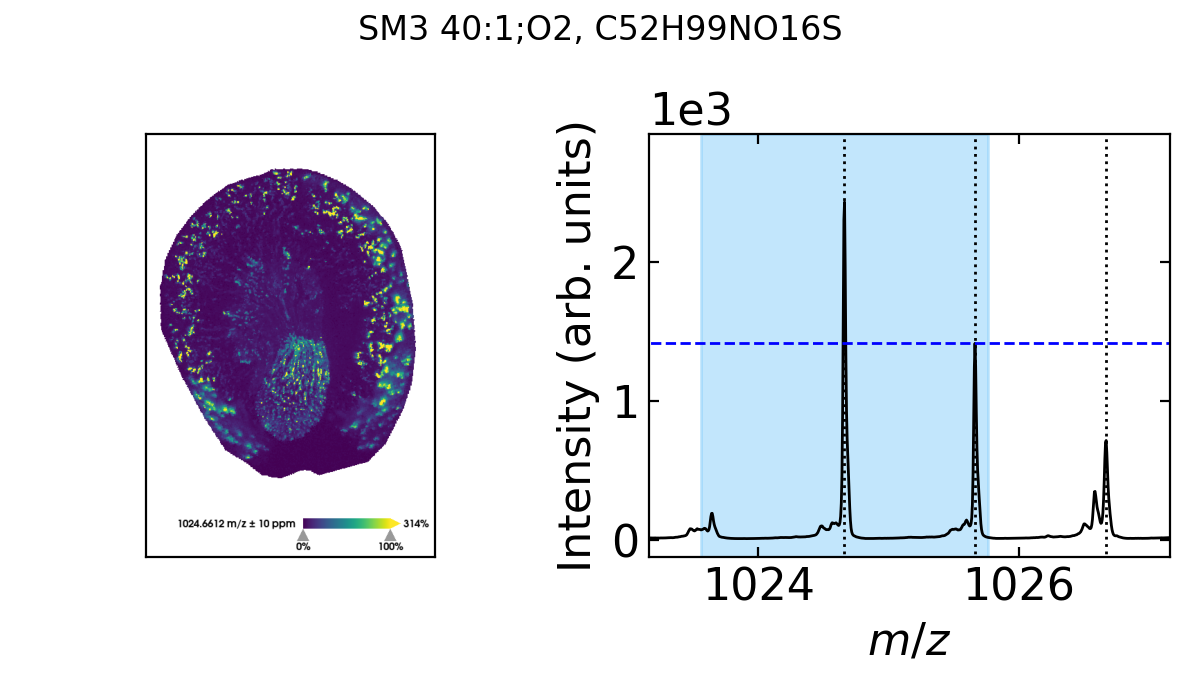

Supplement: Supplementary file 3 — Supplementary Data 1 [file 41467_2025_59839_MOESM3_ESM.zip › Suppl_Dataset_1_REV/qTOF_data1_slide1_python/1024.661181_qTOF_60w_1.png]

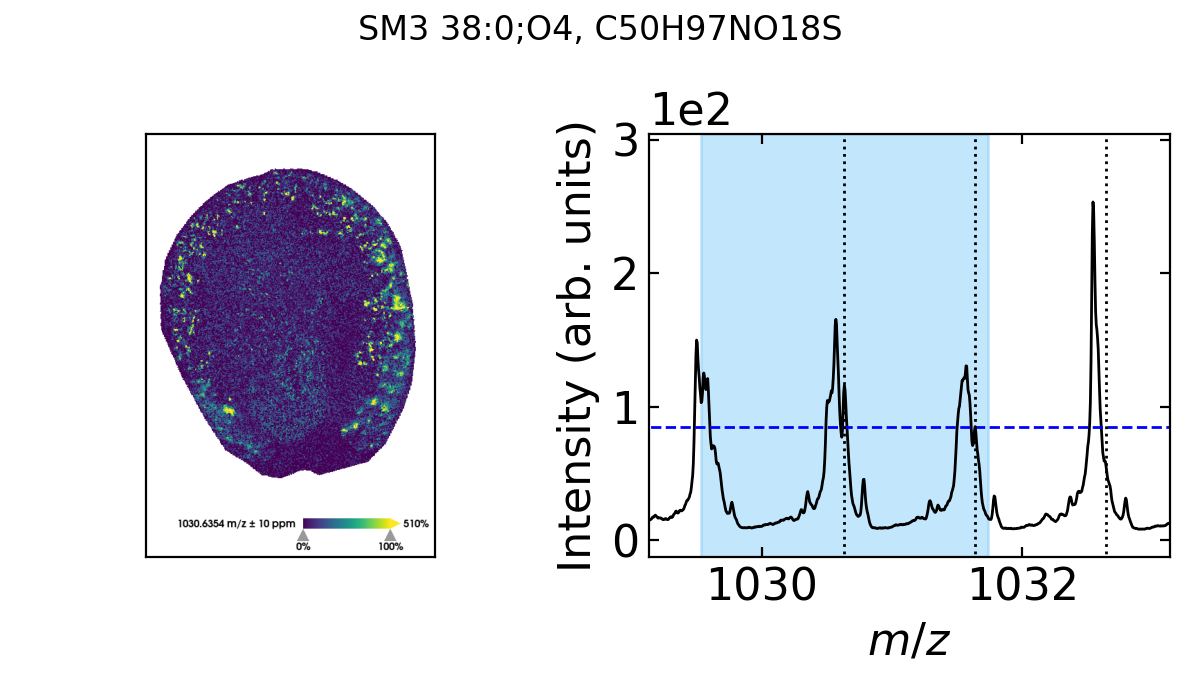

Supplement: Supplementary file 3 — Supplementary Data 1 [file 41467_2025_59839_MOESM3_ESM.zip › Suppl_Dataset_1_REV/qTOF_data1_slide1_python/1030.63536_qTOF_60w_1.png]

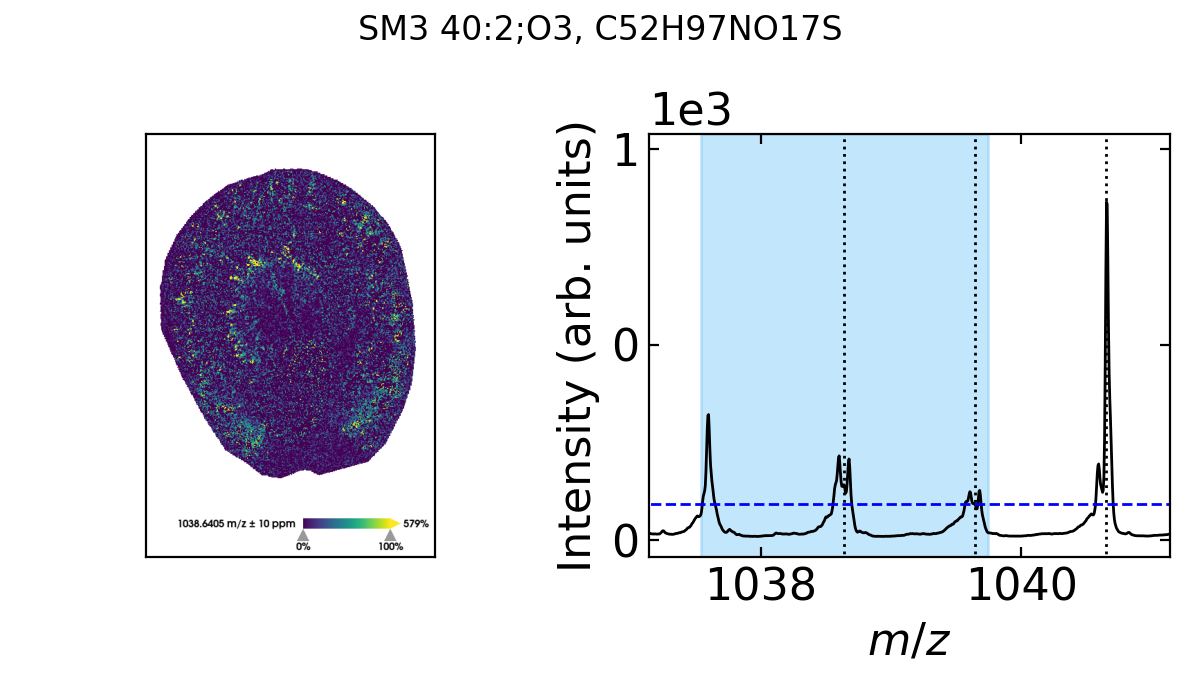

Supplement: Supplementary file 3 — Supplementary Data 1 [file 41467_2025_59839_MOESM3_ESM.zip › Suppl_Dataset_1_REV/qTOF_data1_slide1_python/1038.640446_qTOF_60w_1.png]

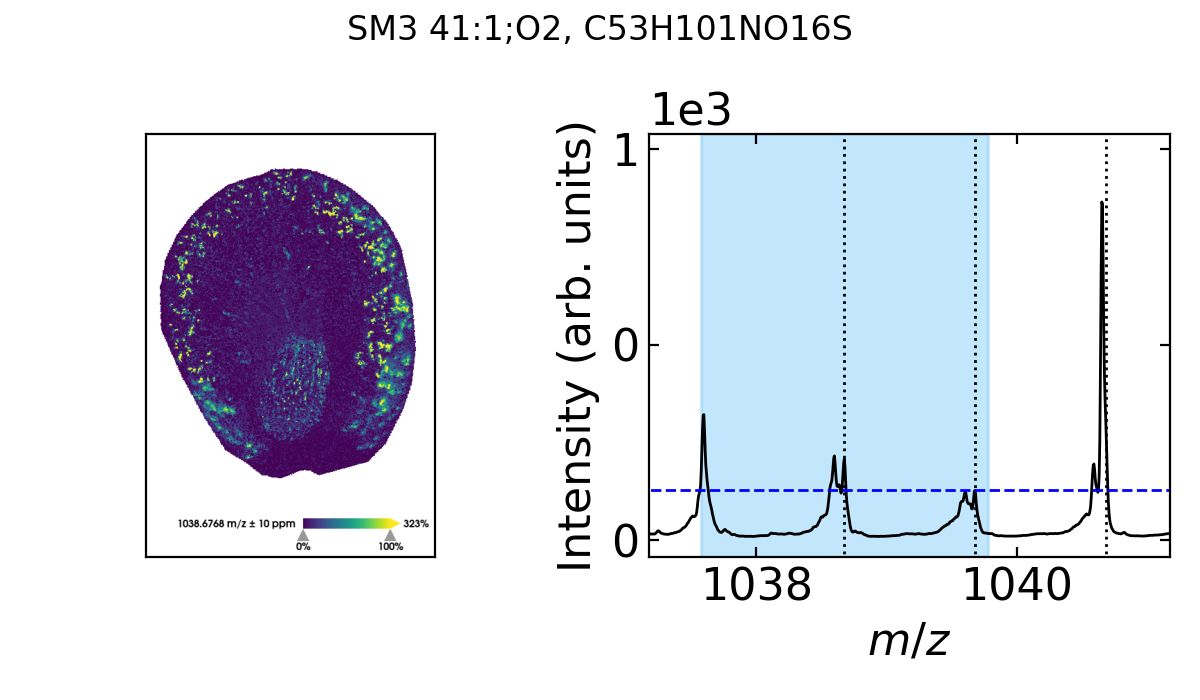

Supplement: Supplementary file 3 — Supplementary Data 1 [file 41467_2025_59839_MOESM3_ESM.zip › Suppl_Dataset_1_REV/qTOF_data1_slide1_python/1038.676831_qTOF_60w_1.png]

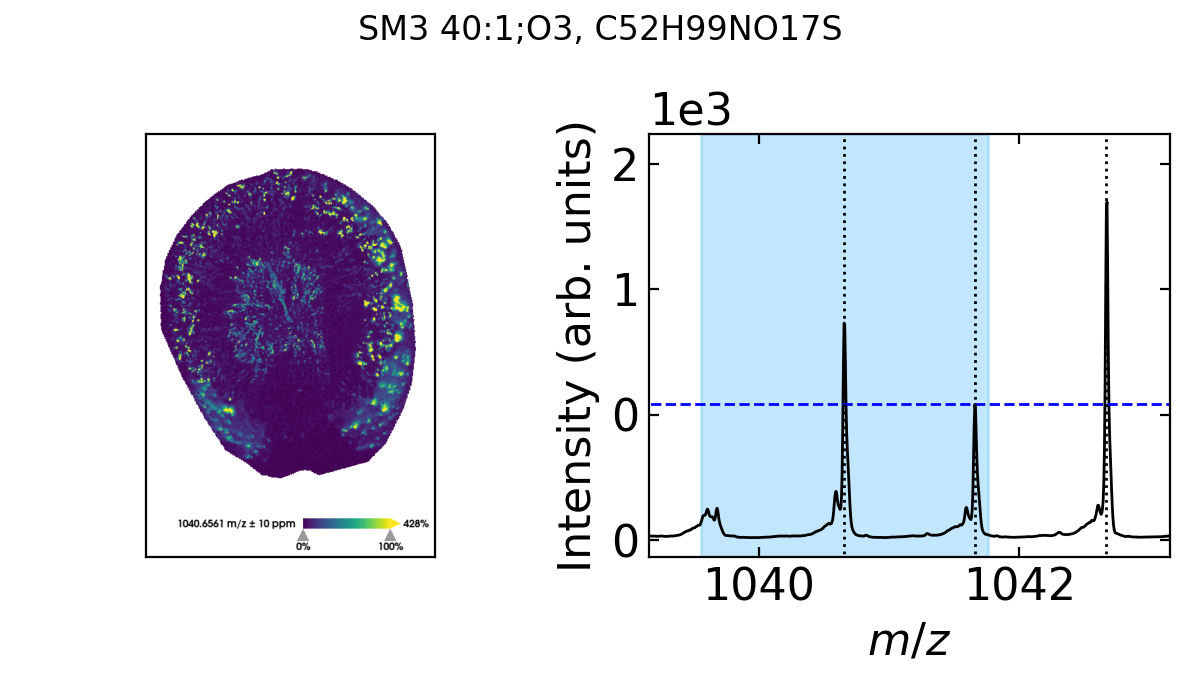

Supplement: Supplementary file 3 — Supplementary Data 1 [file 41467_2025_59839_MOESM3_ESM.zip › Suppl_Dataset_1_REV/qTOF_data1_slide1_python/1040.656096_qTOF_60w_1.png]

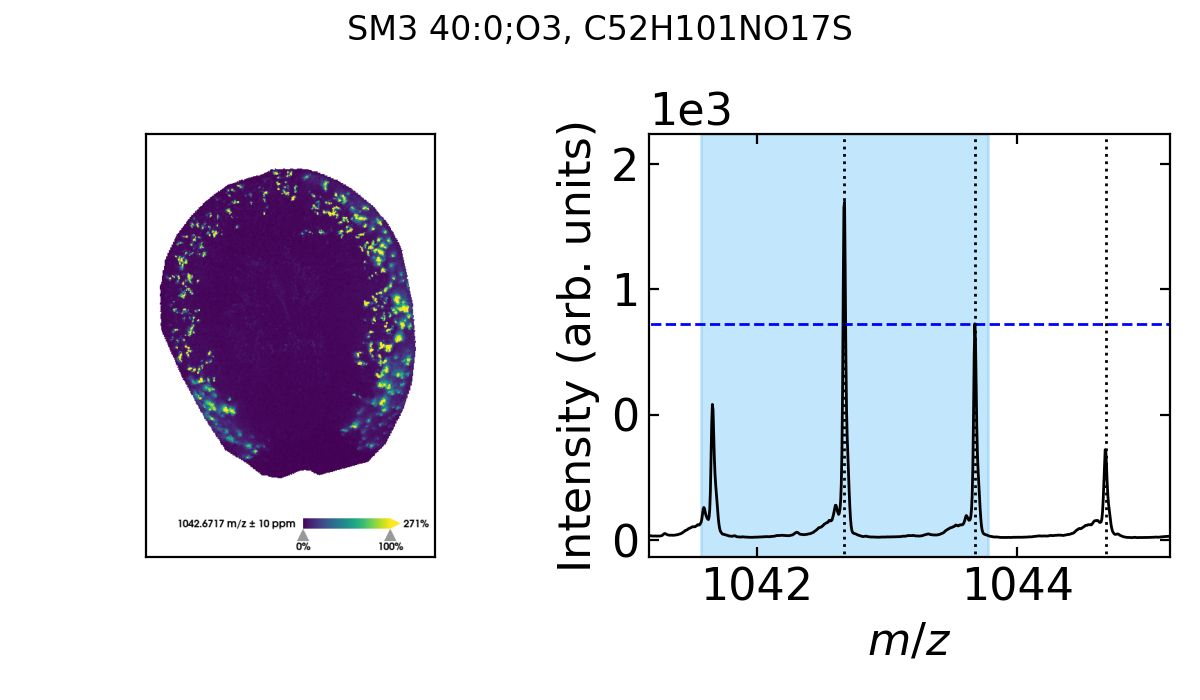

Supplement: Supplementary file 3 — Supplementary Data 1 [file 41467_2025_59839_MOESM3_ESM.zip › Suppl_Dataset_1_REV/qTOF_data1_slide1_python/1042.671746_qTOF_60w_1.png]

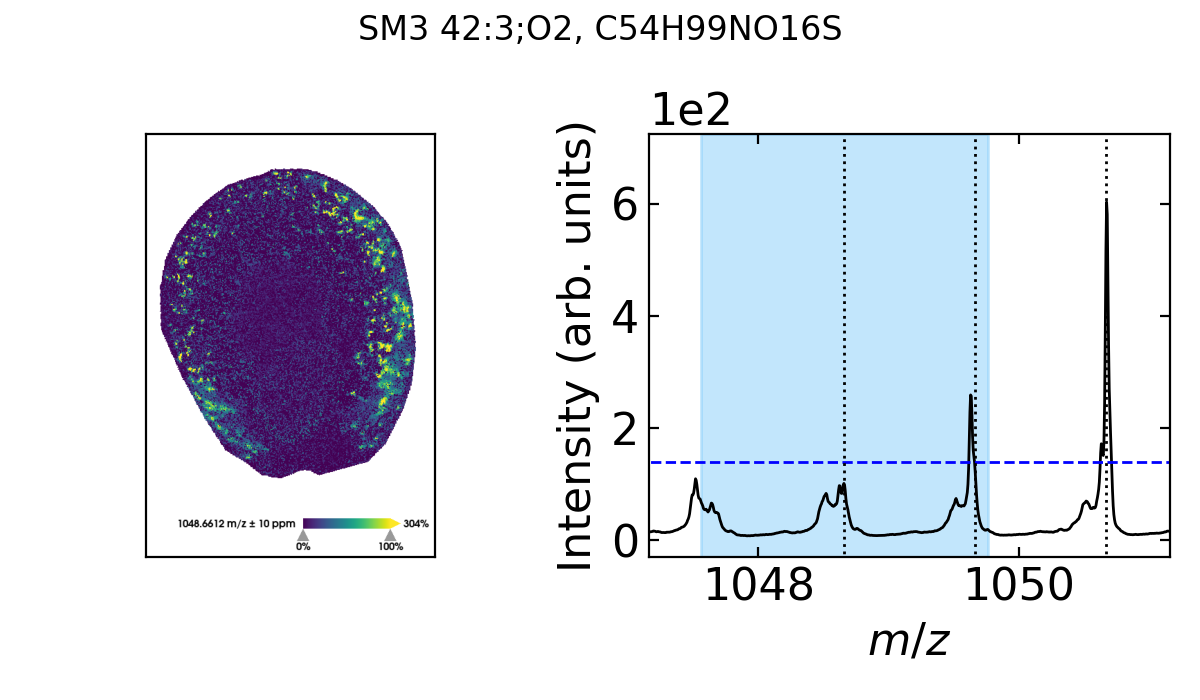

Supplement: Supplementary file 3 — Supplementary Data 1 [file 41467_2025_59839_MOESM3_ESM.zip › Suppl_Dataset_1_REV/qTOF_data1_slide1_python/1048.661181_qTOF_60w_1.png]

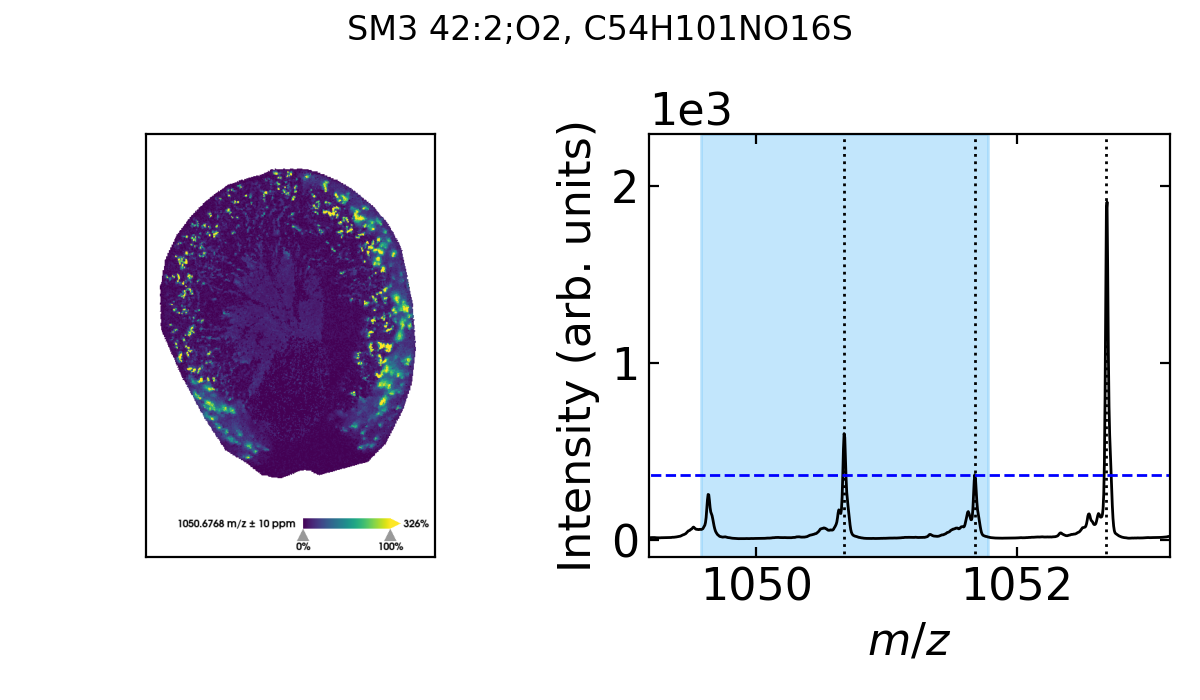

Supplement: Supplementary file 3 — Supplementary Data 1 [file 41467_2025_59839_MOESM3_ESM.zip › Suppl_Dataset_1_REV/qTOF_data1_slide1_python/1050.676831_qTOF_60w_1.png]

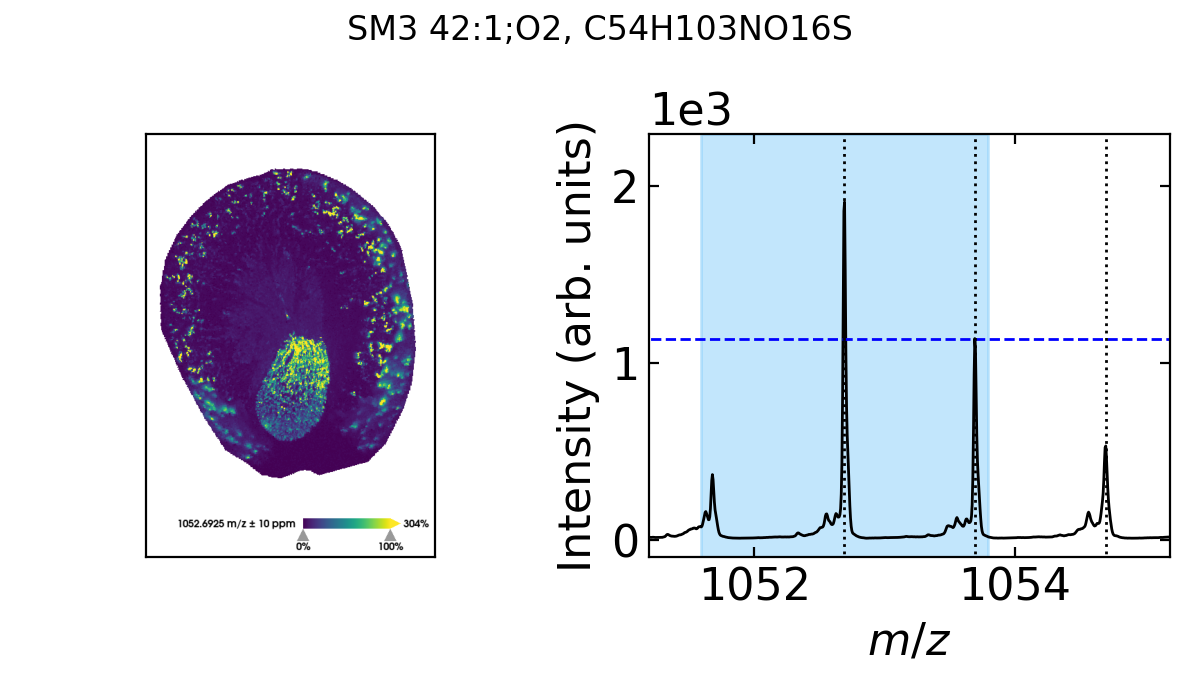

Supplement: Supplementary file 3 — Supplementary Data 1 [file 41467_2025_59839_MOESM3_ESM.zip › Suppl_Dataset_1_REV/qTOF_data1_slide1_python/1052.692481_qTOF_60w_1.png]

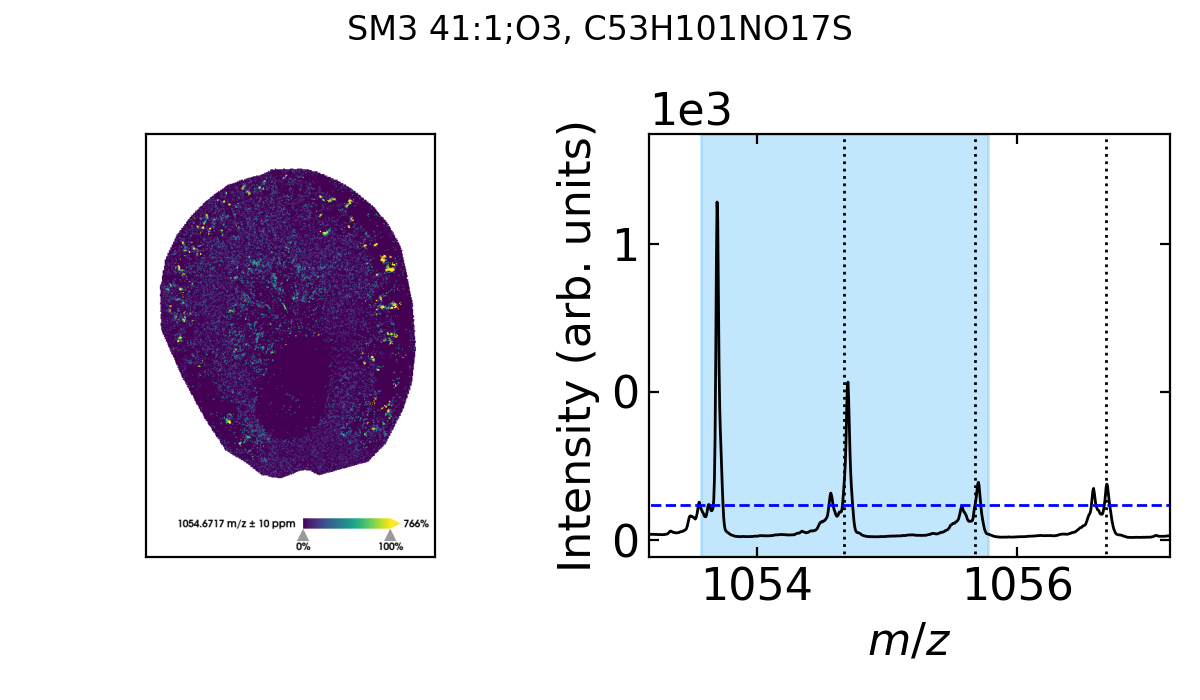

Supplement: Supplementary file 3 — Supplementary Data 1 [file 41467_2025_59839_MOESM3_ESM.zip › Suppl_Dataset_1_REV/qTOF_data1_slide1_python/1054.671746_qTOF_60w_1.png]

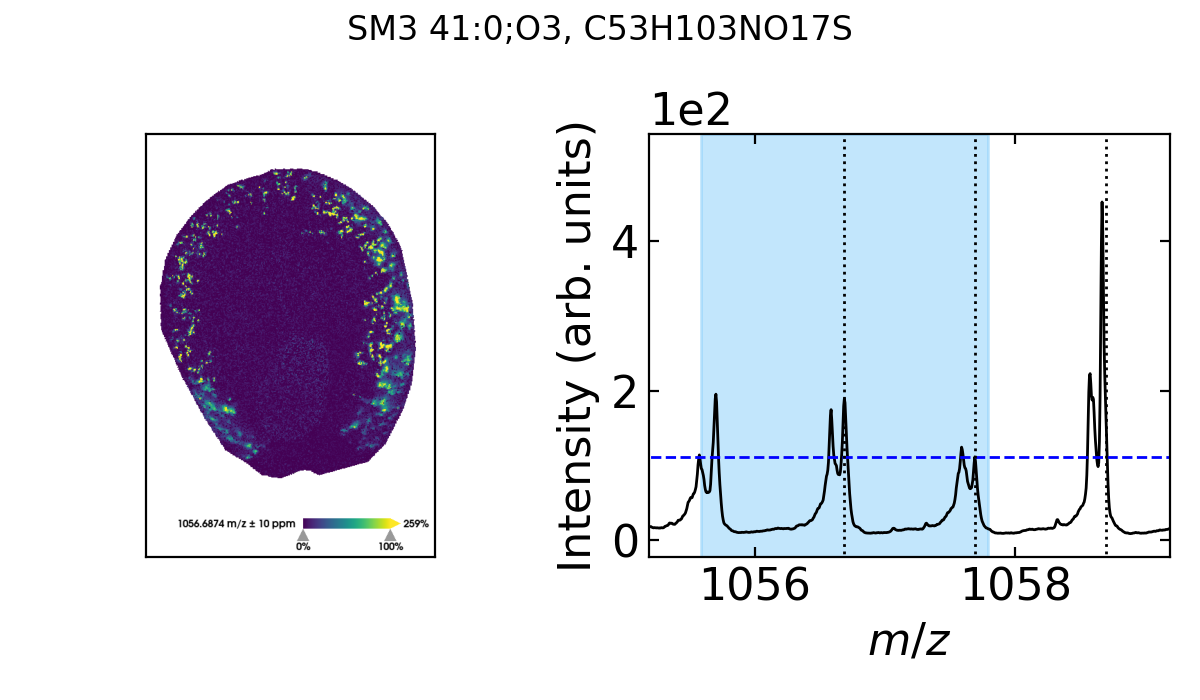

Supplement: Supplementary file 3 — Supplementary Data 1 [file 41467_2025_59839_MOESM3_ESM.zip › Suppl_Dataset_1_REV/qTOF_data1_slide1_python/1056.687396_qTOF_60w_1.png]

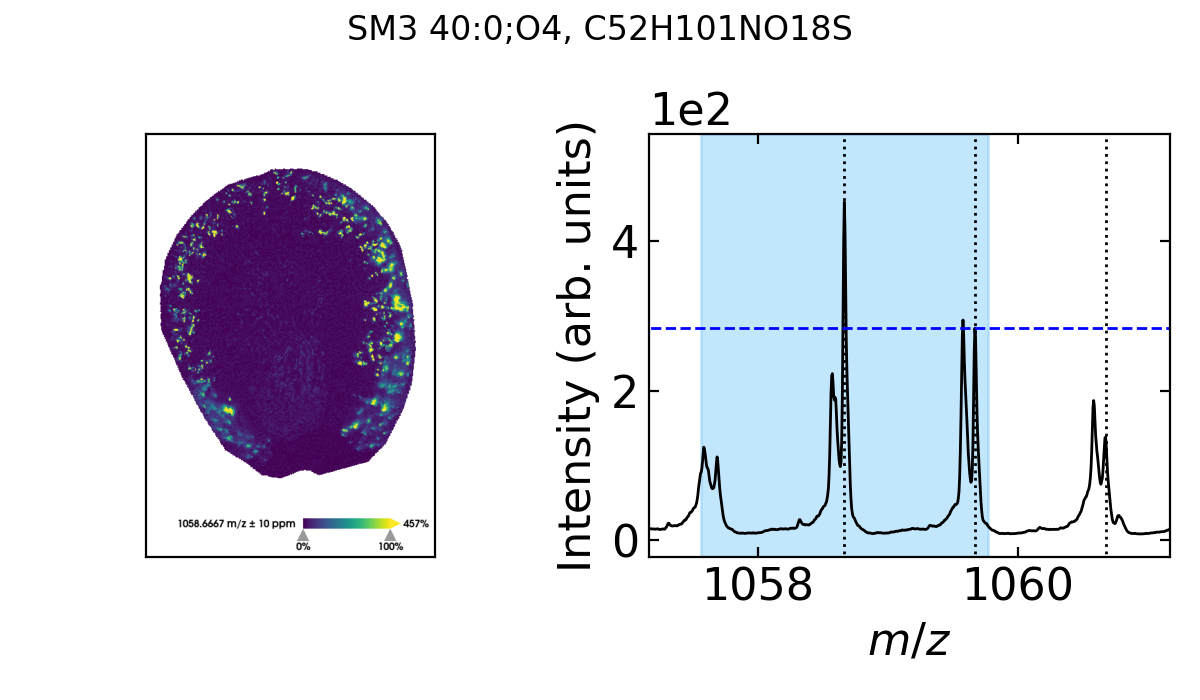

Supplement: Supplementary file 3 — Supplementary Data 1 [file 41467_2025_59839_MOESM3_ESM.zip › Suppl_Dataset_1_REV/qTOF_data1_slide1_python/1058.666661_qTOF_60w_1.png]

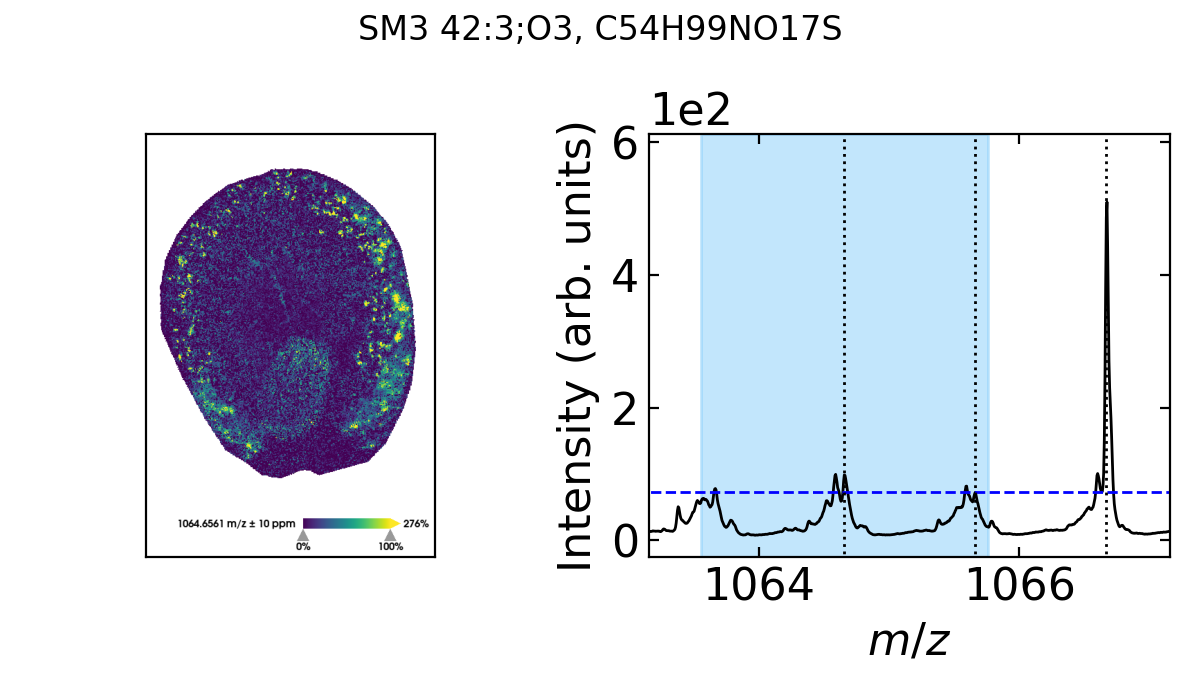

Supplement: Supplementary file 3 — Supplementary Data 1 [file 41467_2025_59839_MOESM3_ESM.zip › Suppl_Dataset_1_REV/qTOF_data1_slide1_python/1064.656096_qTOF_60w_1.png]

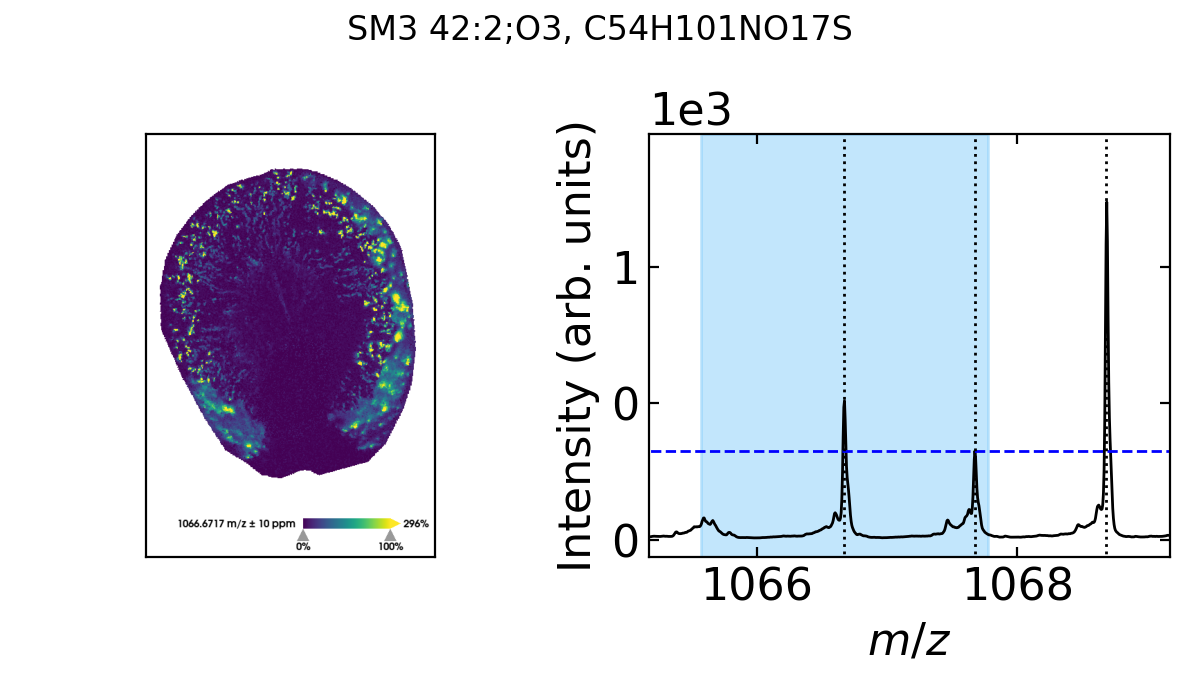

Supplement: Supplementary file 3 — Supplementary Data 1 [file 41467_2025_59839_MOESM3_ESM.zip › Suppl_Dataset_1_REV/qTOF_data1_slide1_python/1066.671746_qTOF_60w_1.png]

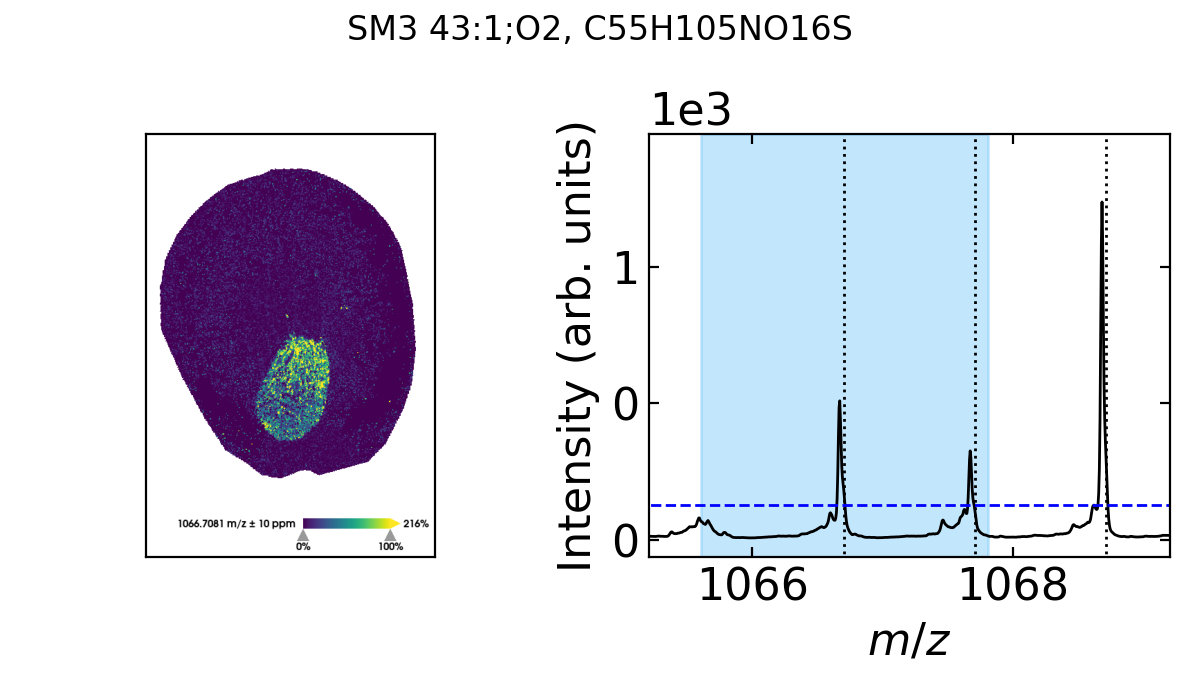

Supplement: Supplementary file 3 — Supplementary Data 1 [file 41467_2025_59839_MOESM3_ESM.zip › Suppl_Dataset_1_REV/qTOF_data1_slide1_python/1066.708131_qTOF_60w_1.png]

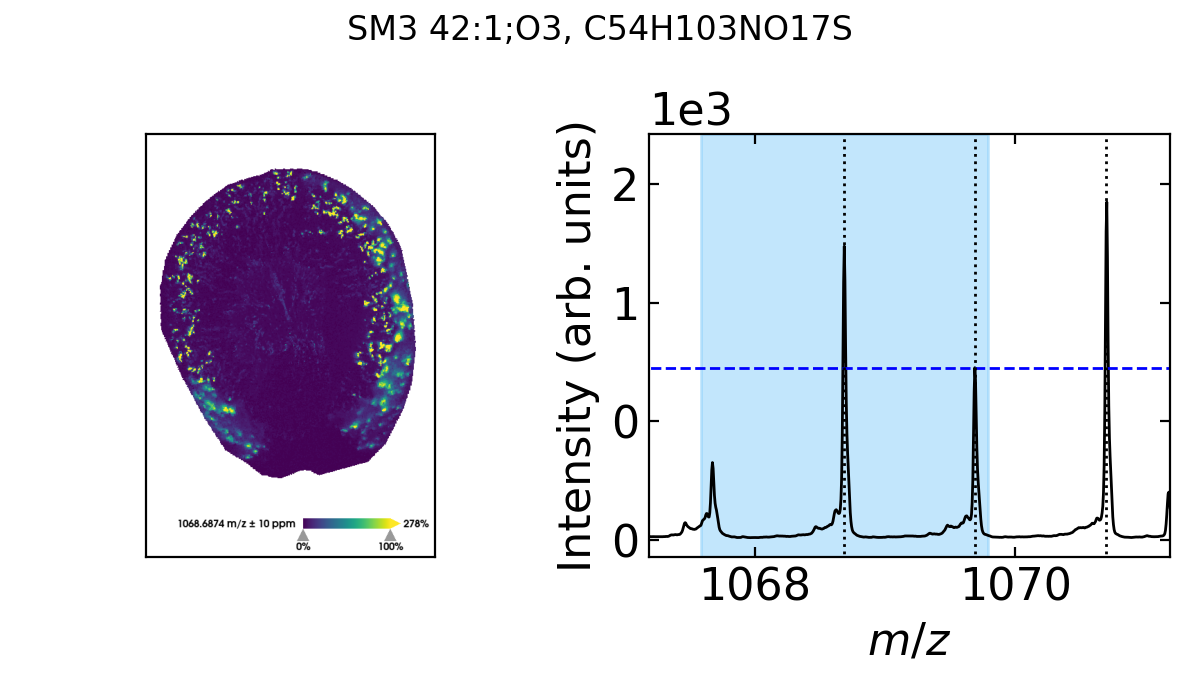

Supplement: Supplementary file 3 — Supplementary Data 1 [file 41467_2025_59839_MOESM3_ESM.zip › Suppl_Dataset_1_REV/qTOF_data1_slide1_python/1068.687396_qTOF_60w_1.png]

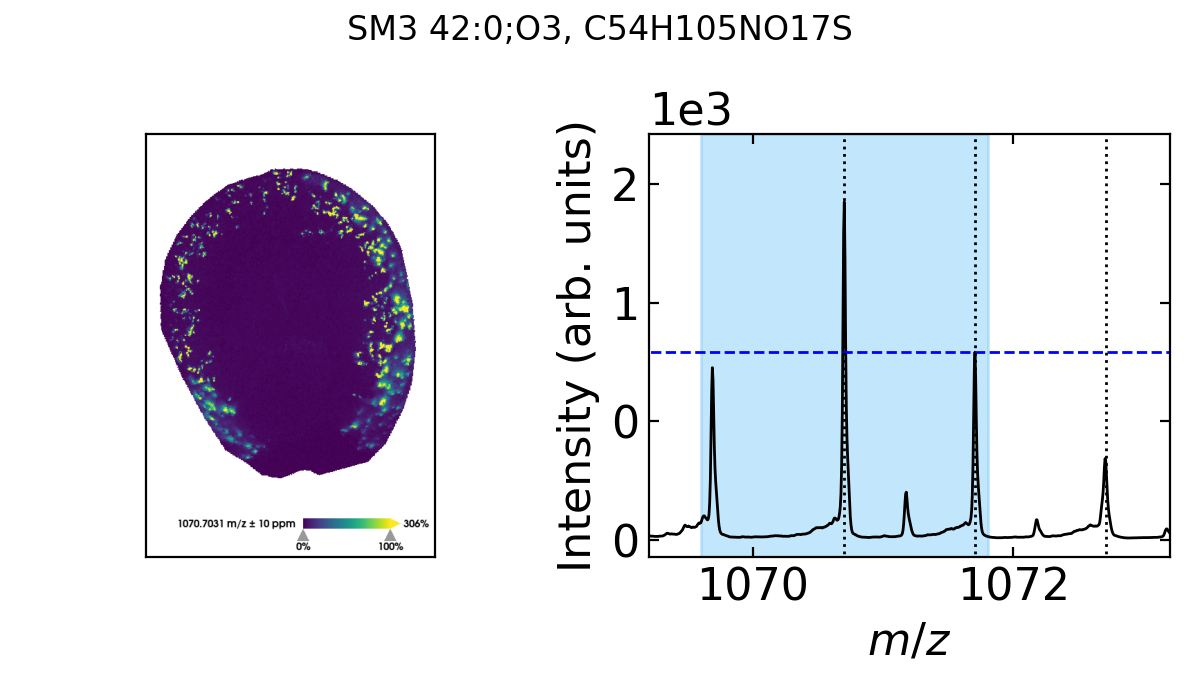

Supplement: Supplementary file 3 — Supplementary Data 1 [file 41467_2025_59839_MOESM3_ESM.zip › Suppl_Dataset_1_REV/qTOF_data1_slide1_python/1070.703046_qTOF_60w_1.png]

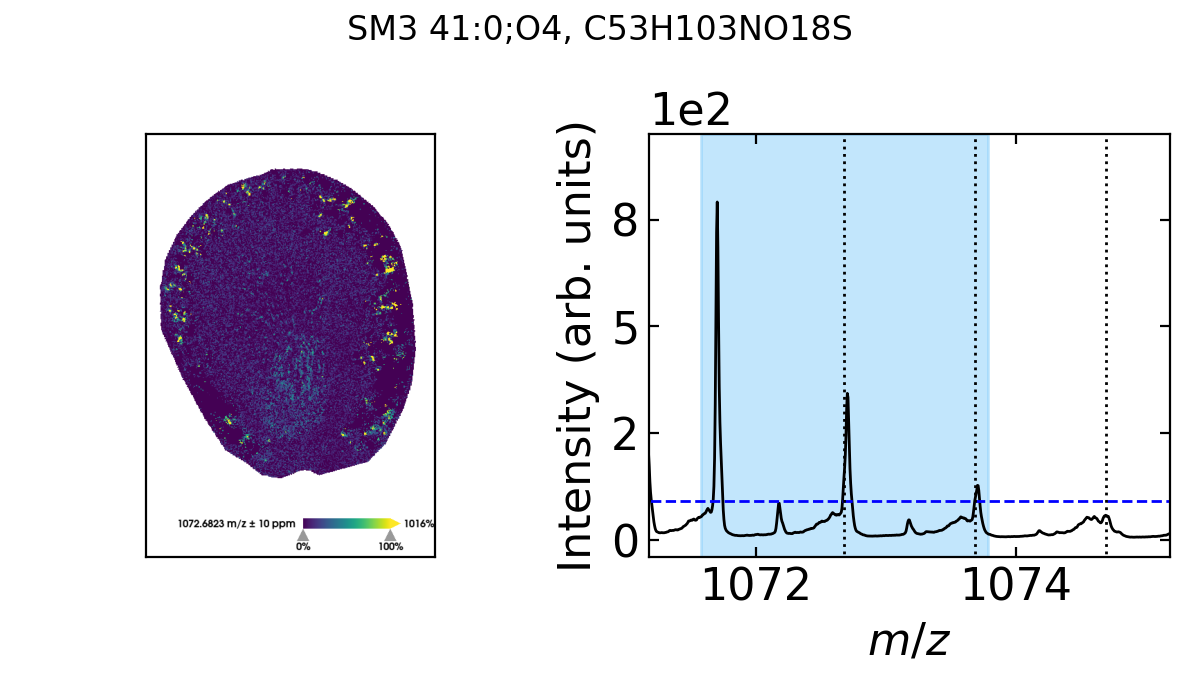

Supplement: Supplementary file 3 — Supplementary Data 1 [file 41467_2025_59839_MOESM3_ESM.zip › Suppl_Dataset_1_REV/qTOF_data1_slide1_python/1072.682311_qTOF_60w_1.png]

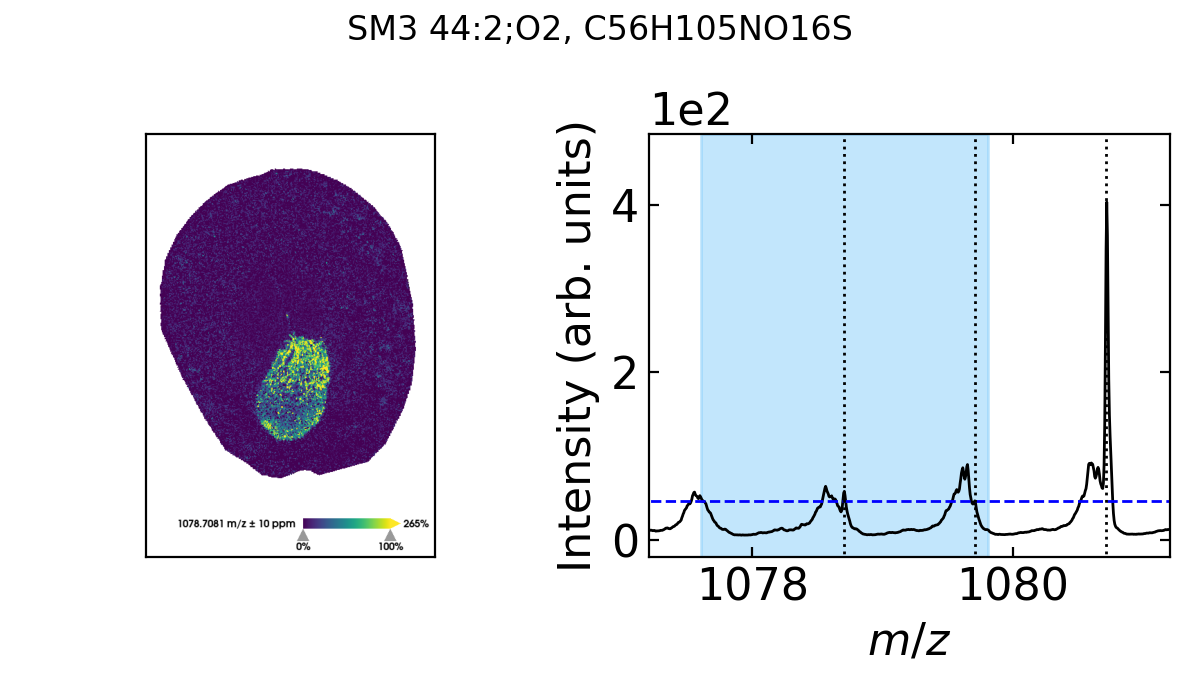

Supplement: Supplementary file 3 — Supplementary Data 1 [file 41467_2025_59839_MOESM3_ESM.zip › Suppl_Dataset_1_REV/qTOF_data1_slide1_python/1078.708131_qTOF_60w_1.png]

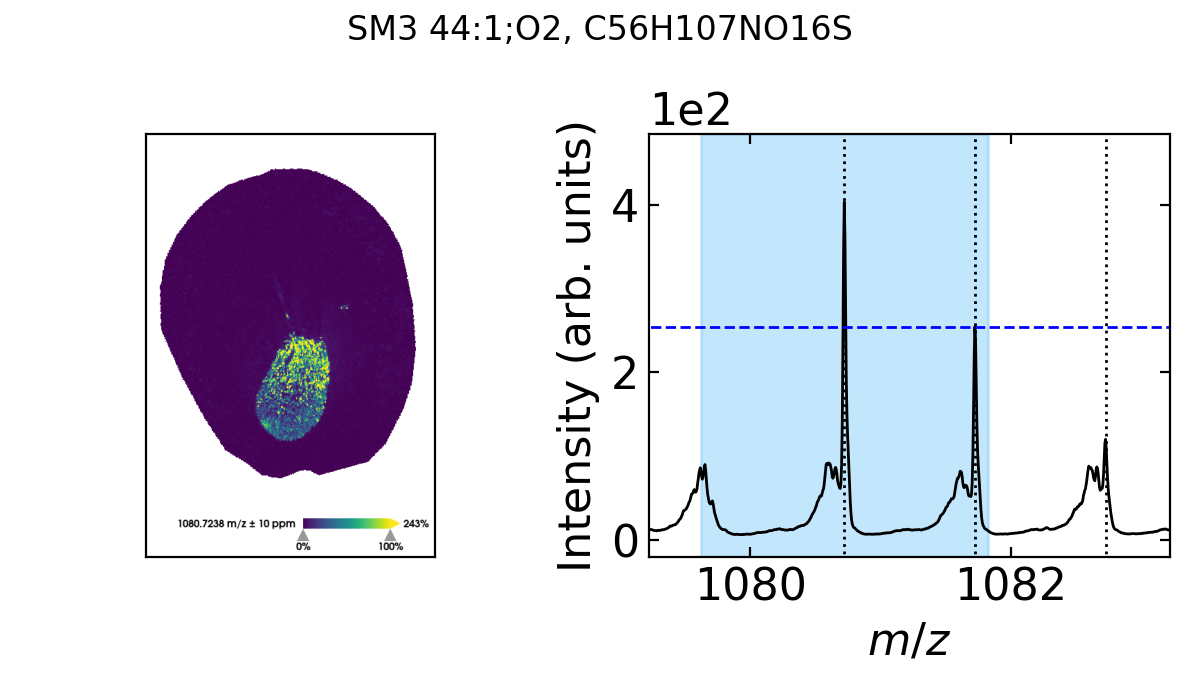

Supplement: Supplementary file 3 — Supplementary Data 1 [file 41467_2025_59839_MOESM3_ESM.zip › Suppl_Dataset_1_REV/qTOF_data1_slide1_python/1080.723781_qTOF_60w_1.png]

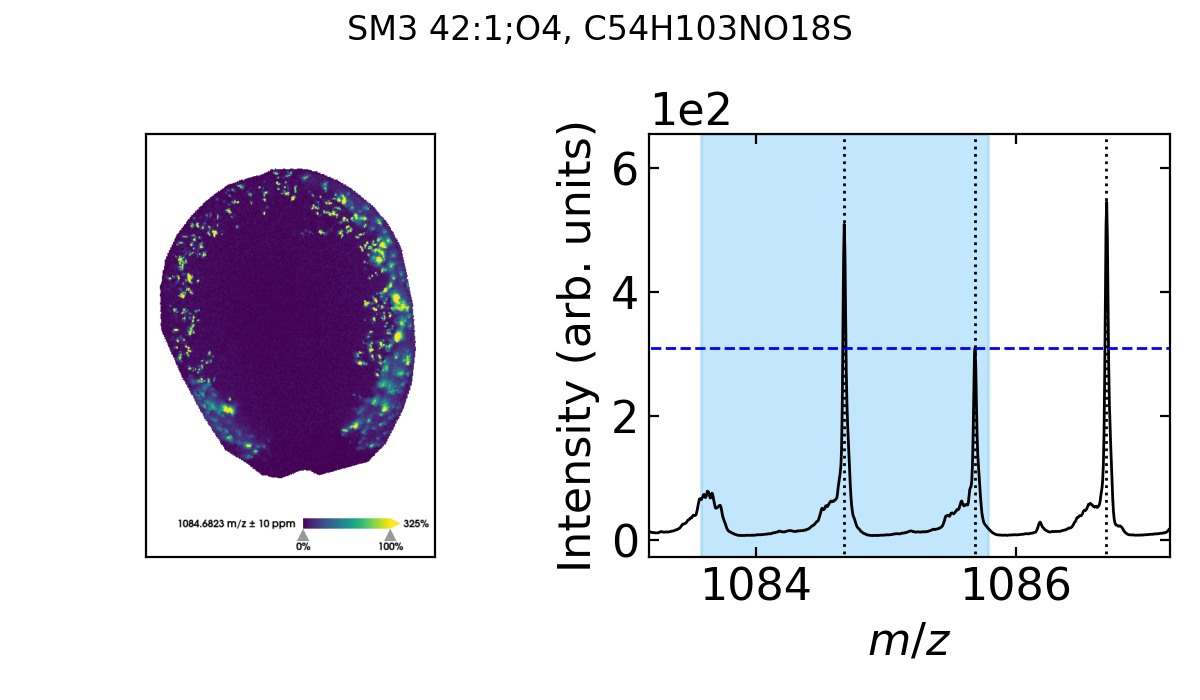

Supplement: Supplementary file 3 — Supplementary Data 1 [file 41467_2025_59839_MOESM3_ESM.zip › Suppl_Dataset_1_REV/qTOF_data1_slide1_python/1084.682311_qTOF_60w_1.png]

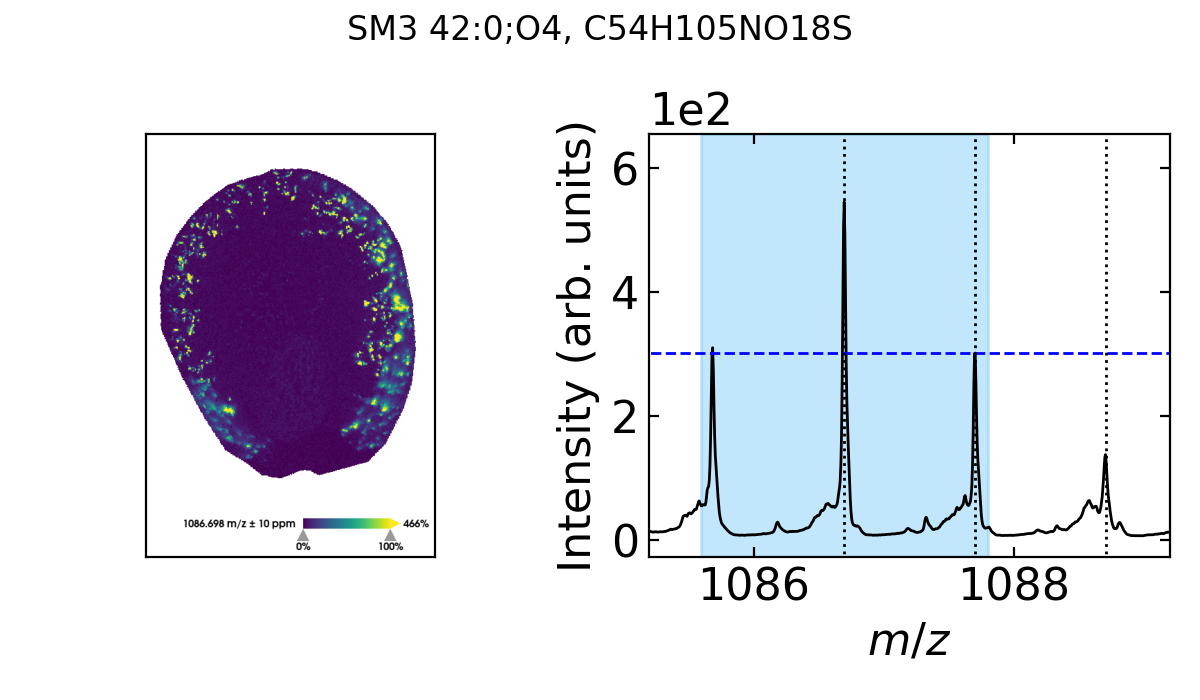

Supplement: Supplementary file 3 — Supplementary Data 1 [file 41467_2025_59839_MOESM3_ESM.zip › Suppl_Dataset_1_REV/qTOF_data1_slide1_python/1086.697961_qTOF_60w_1.png]

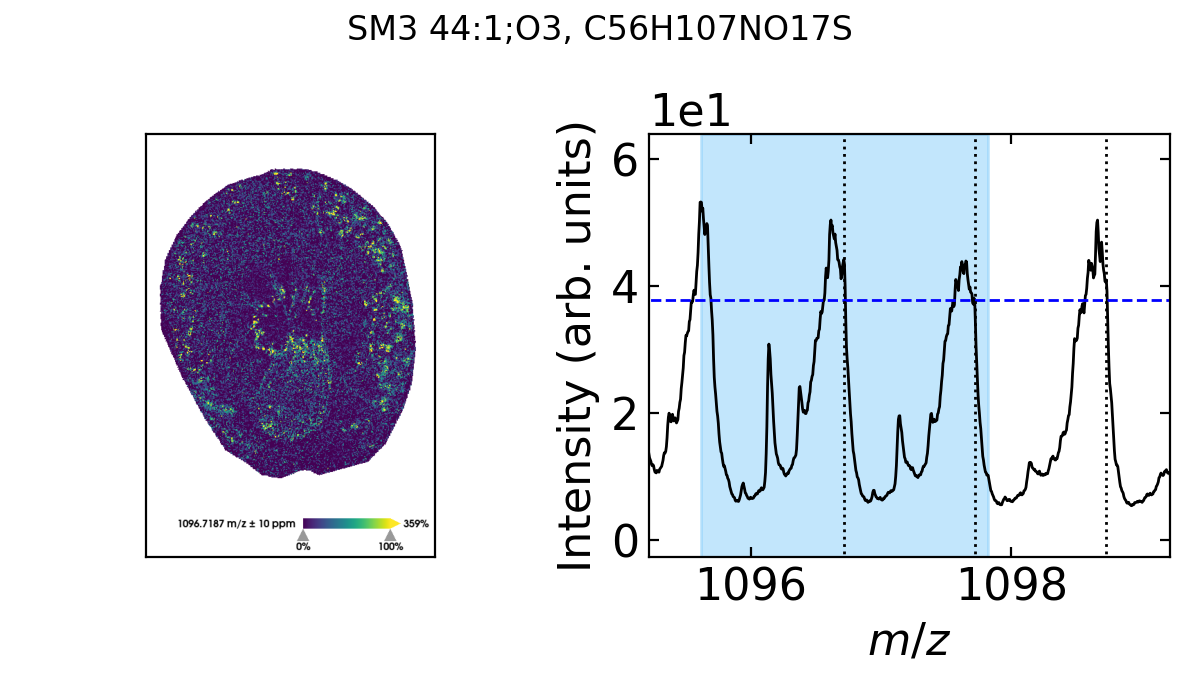

Supplement: Supplementary file 3 — Supplementary Data 1 [file 41467_2025_59839_MOESM3_ESM.zip › Suppl_Dataset_1_REV/qTOF_data1_slide1_python/1096.718696_qTOF_60w_1.png]

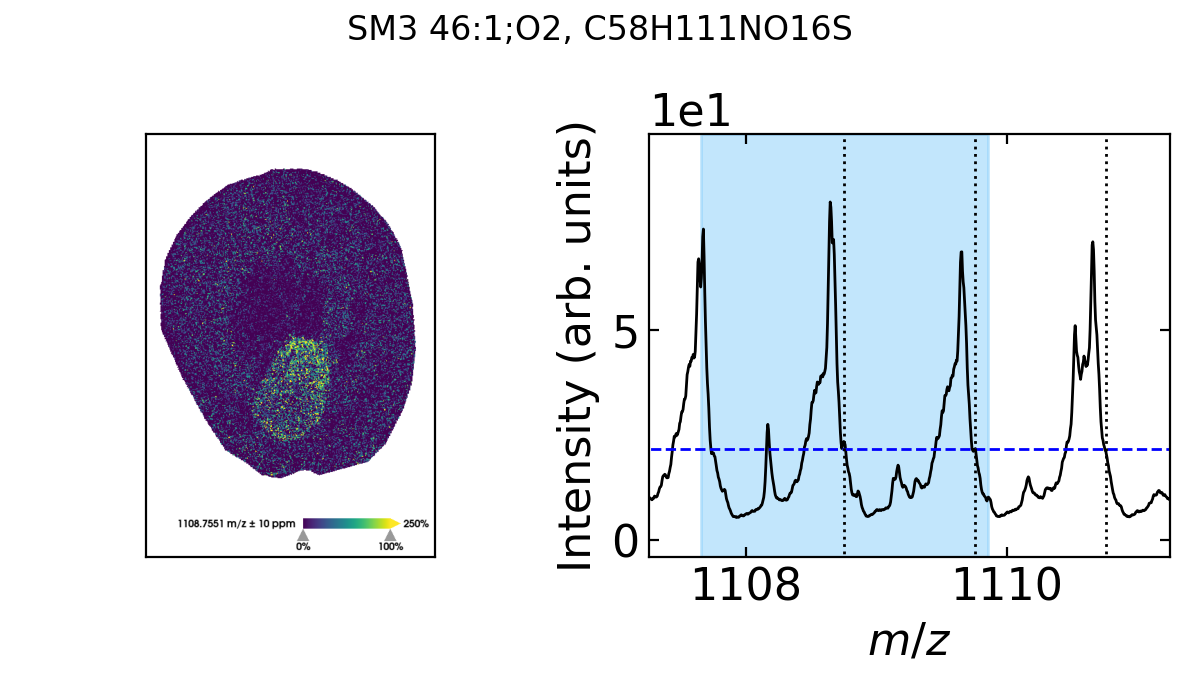

Supplement: Supplementary file 3 — Supplementary Data 1 [file 41467_2025_59839_MOESM3_ESM.zip › Suppl_Dataset_1_REV/qTOF_data1_slide1_python/1108.755082_qTOF_60w_1.png]

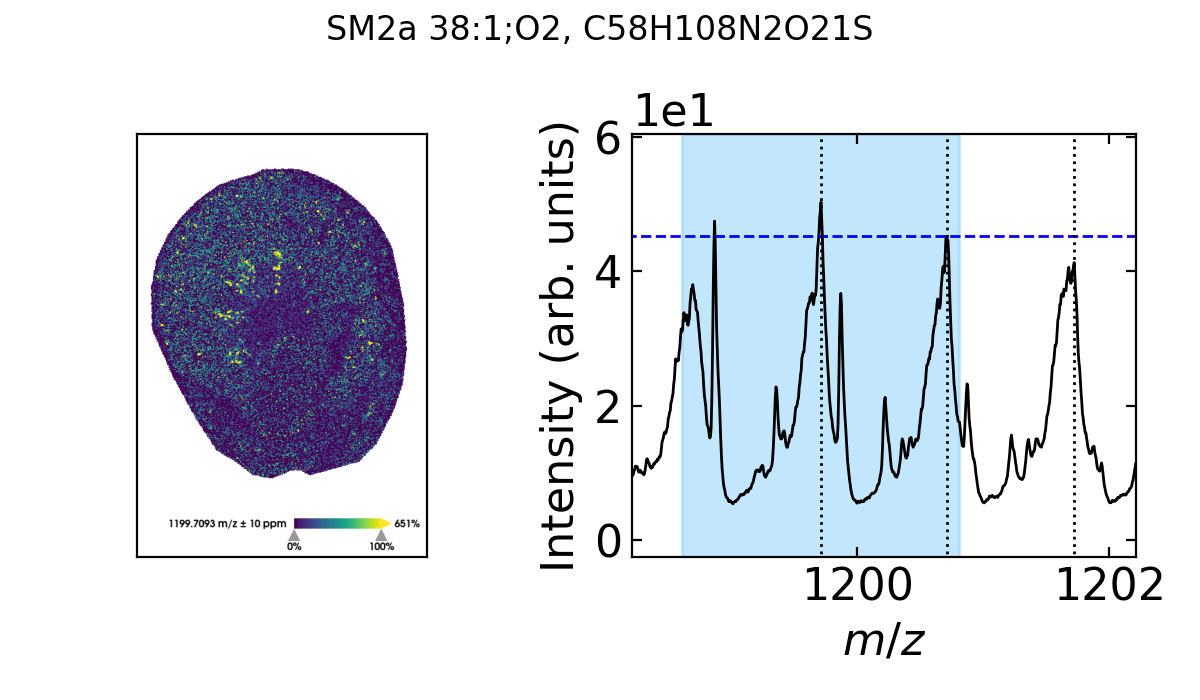

Supplement: Supplementary file 3 — Supplementary Data 1 [file 41467_2025_59839_MOESM3_ESM.zip › Suppl_Dataset_1_REV/qTOF_data1_slide1_python/1199.709253_qTOF_60w_1.png]

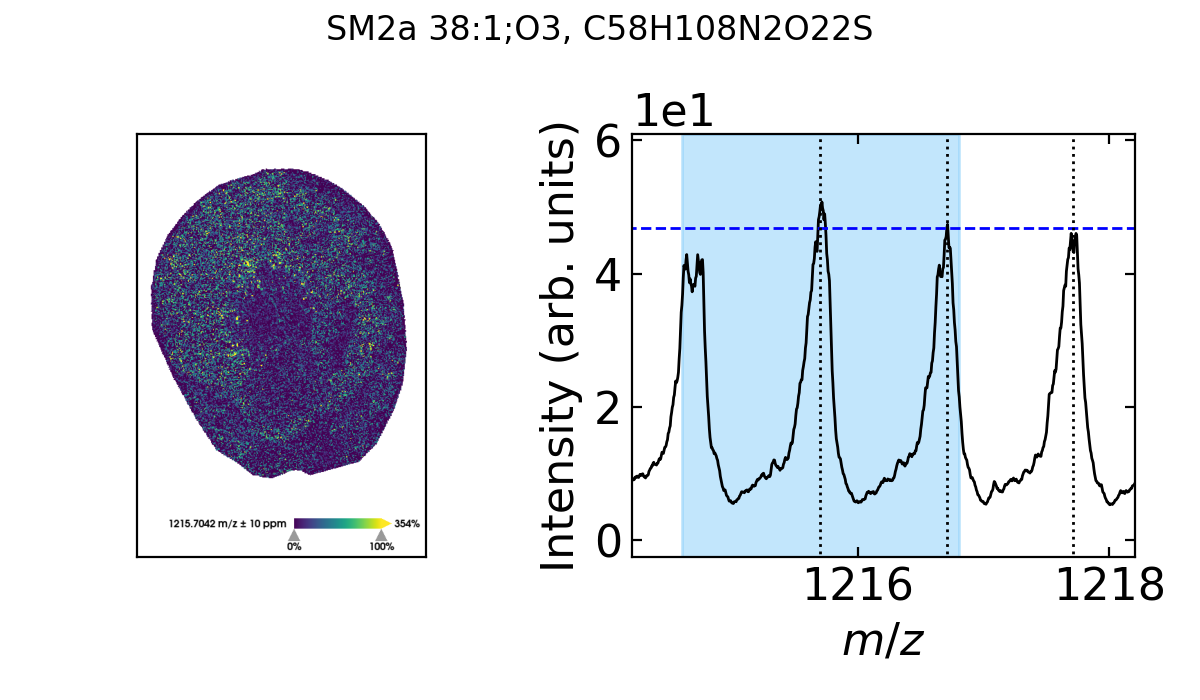

Supplement: Supplementary file 3 — Supplementary Data 1 [file 41467_2025_59839_MOESM3_ESM.zip › Suppl_Dataset_1_REV/qTOF_data1_slide1_python/1215.704168_qTOF_60w_1.png]

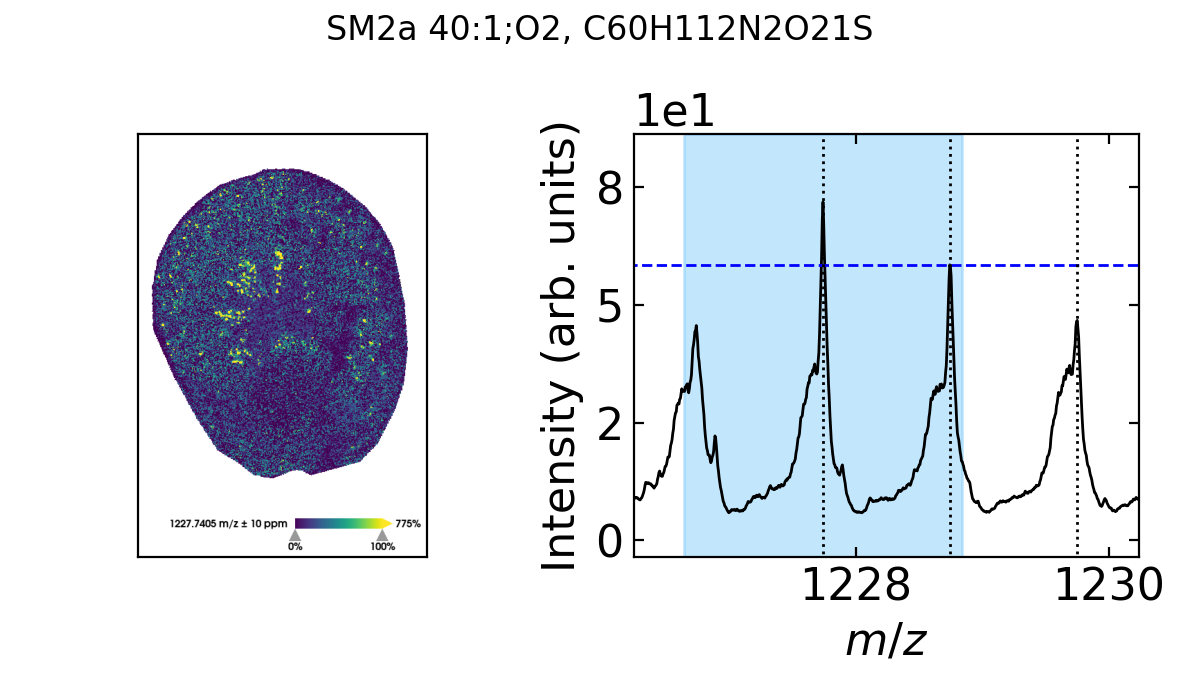

Supplement: Supplementary file 3 — Supplementary Data 1 [file 41467_2025_59839_MOESM3_ESM.zip › Suppl_Dataset_1_REV/qTOF_data1_slide1_python/1227.740553_qTOF_60w_1.png]

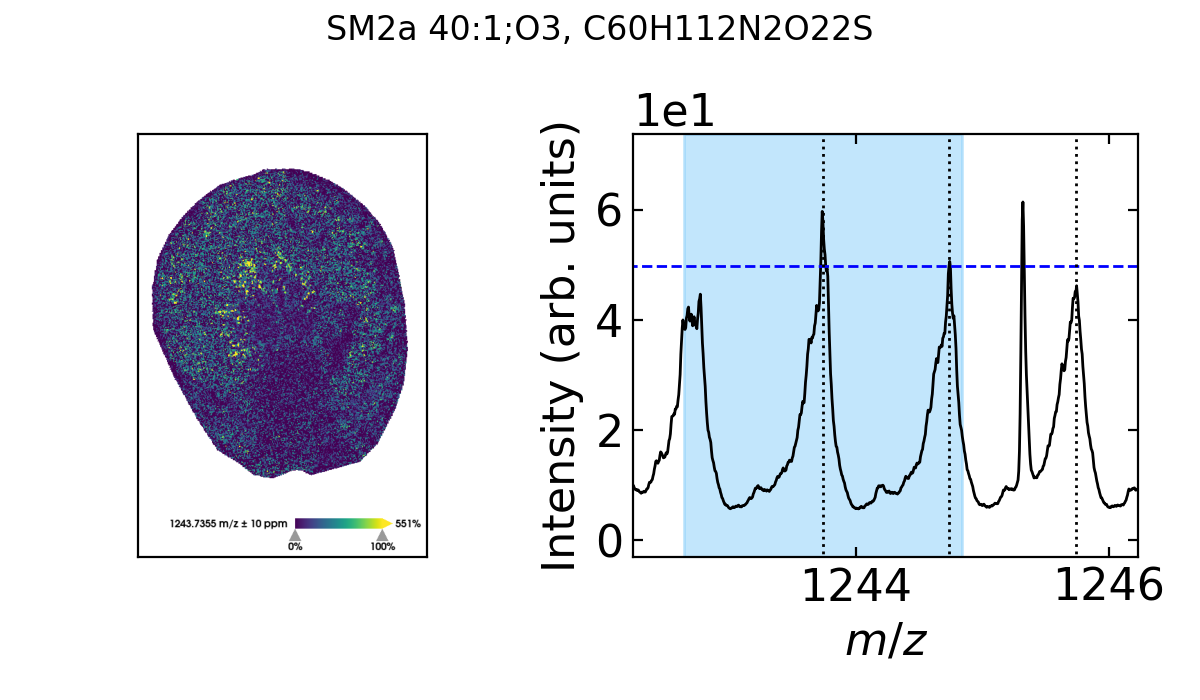

Supplement: Supplementary file 3 — Supplementary Data 1 [file 41467_2025_59839_MOESM3_ESM.zip › Suppl_Dataset_1_REV/qTOF_data1_slide1_python/1243.735468_qTOF_60w_1.png]

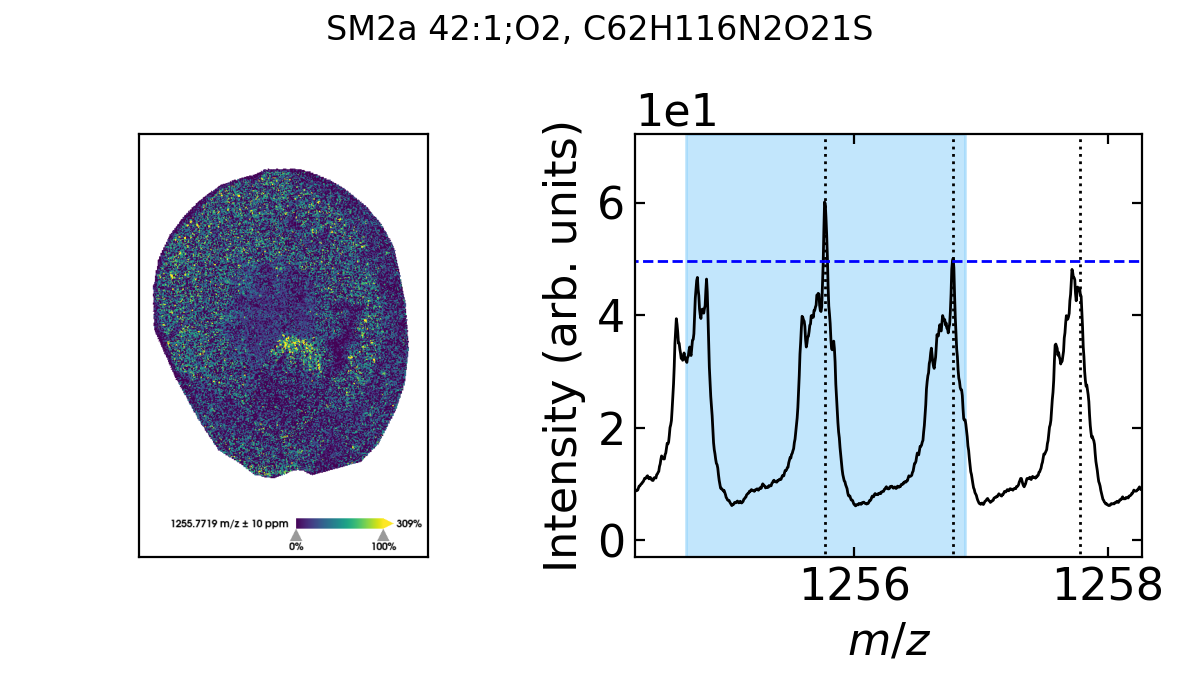

Supplement: Supplementary file 3 — Supplementary Data 1 [file 41467_2025_59839_MOESM3_ESM.zip › Suppl_Dataset_1_REV/qTOF_data1_slide1_python/1255.771853_qTOF_60w_1.png]

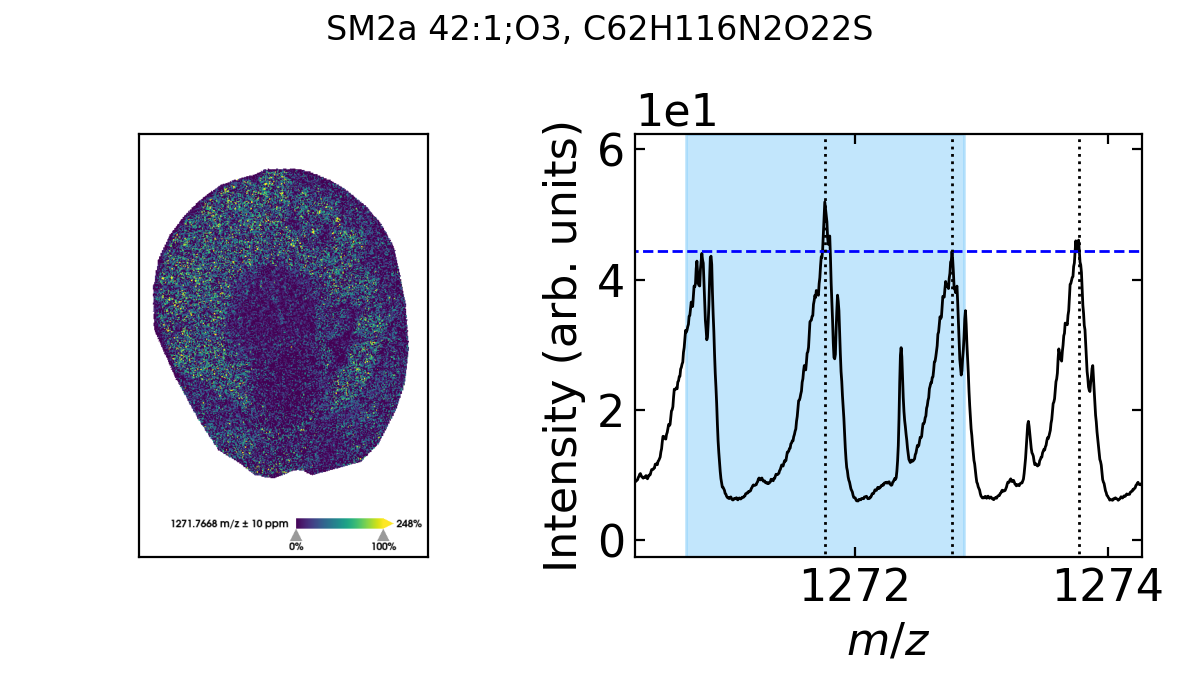

Supplement: Supplementary file 3 — Supplementary Data 1 [file 41467_2025_59839_MOESM3_ESM.zip › Suppl_Dataset_1_REV/qTOF_data1_slide1_python/1271.766768_qTOF_60w_1.png]

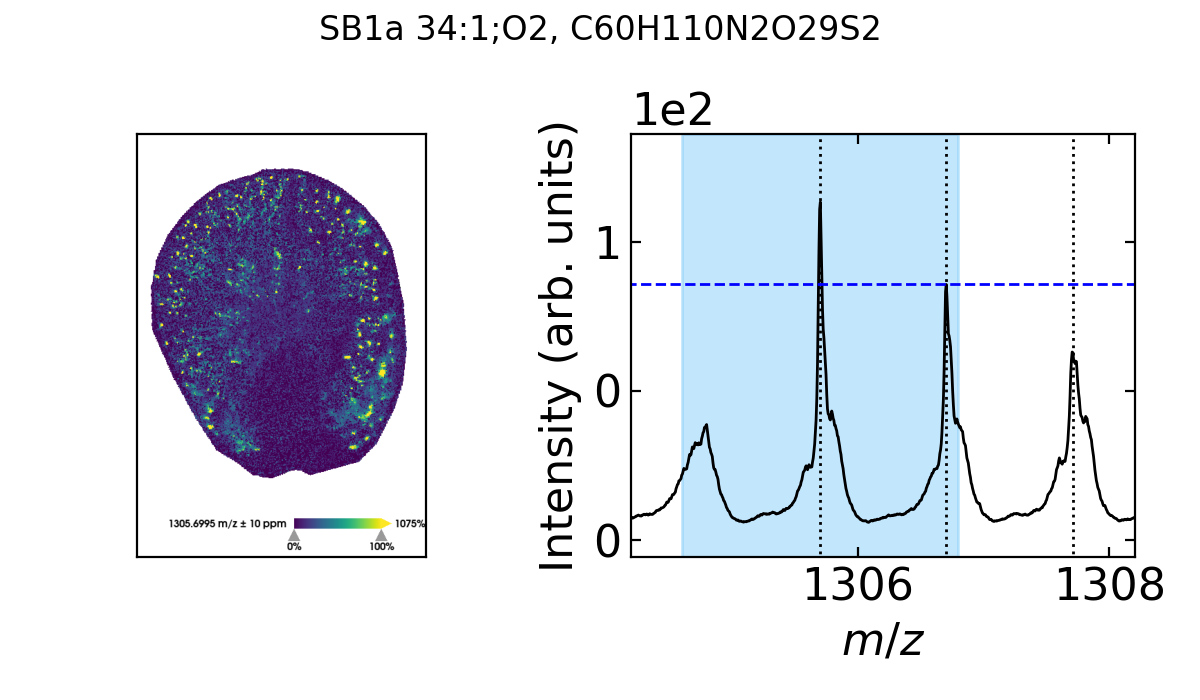

Supplement: Supplementary file 3 — Supplementary Data 1 [file 41467_2025_59839_MOESM3_ESM.zip › Suppl_Dataset_1_REV/qTOF_data1_slide1_python/1305.699477_qTOF_60w_1.png]

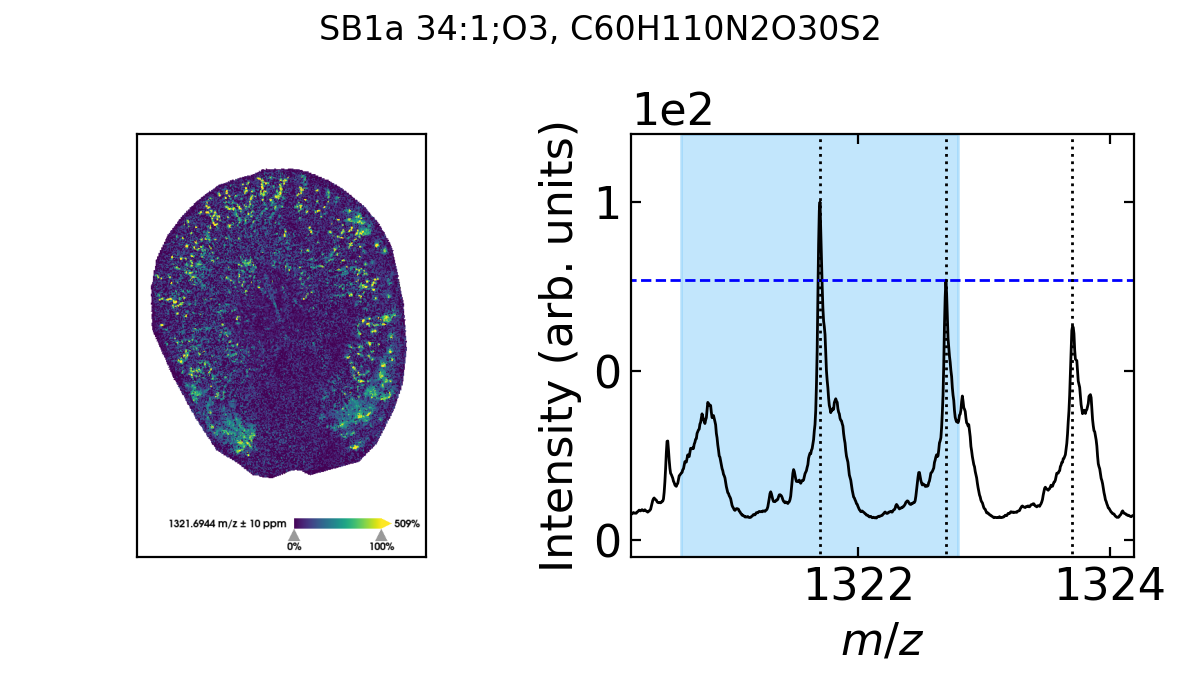

Supplement: Supplementary file 3 — Supplementary Data 1 [file 41467_2025_59839_MOESM3_ESM.zip › Suppl_Dataset_1_REV/qTOF_data1_slide1_python/1321.694392_qTOF_60w_1.png]

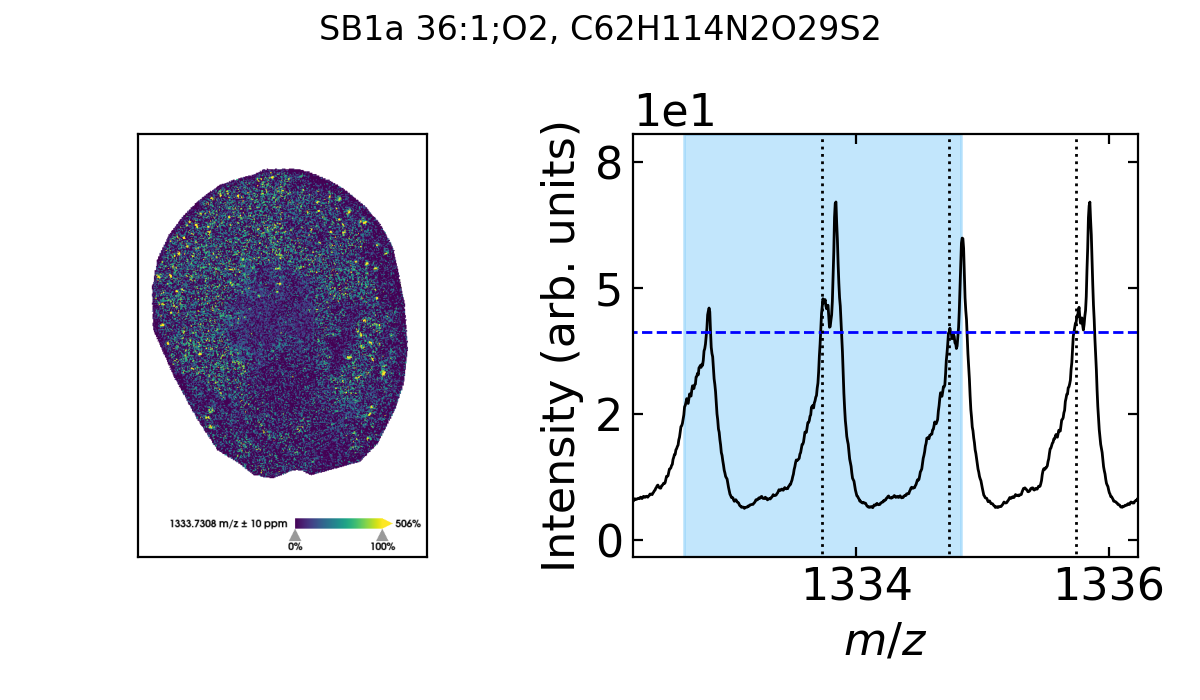

Supplement: Supplementary file 3 — Supplementary Data 1 [file 41467_2025_59839_MOESM3_ESM.zip › Suppl_Dataset_1_REV/qTOF_data1_slide1_python/1333.730777_qTOF_60w_1.png]

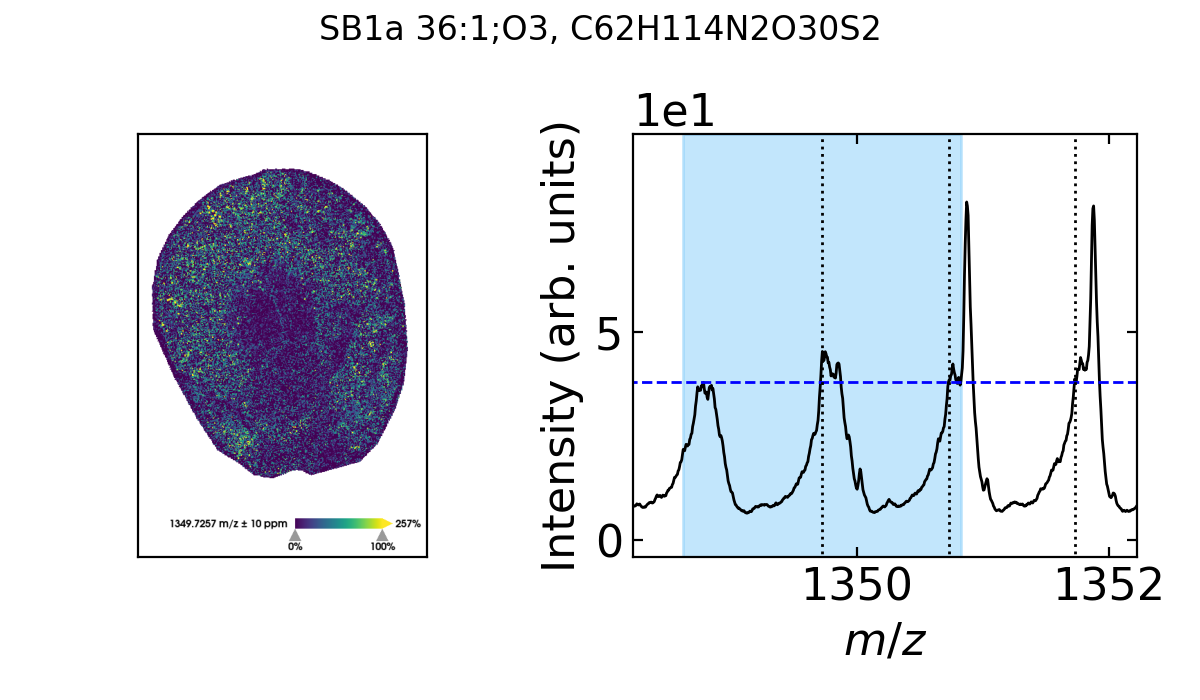

Supplement: Supplementary file 3 — Supplementary Data 1 [file 41467_2025_59839_MOESM3_ESM.zip › Suppl_Dataset_1_REV/qTOF_data1_slide1_python/1349.725692_qTOF_60w_1.png]

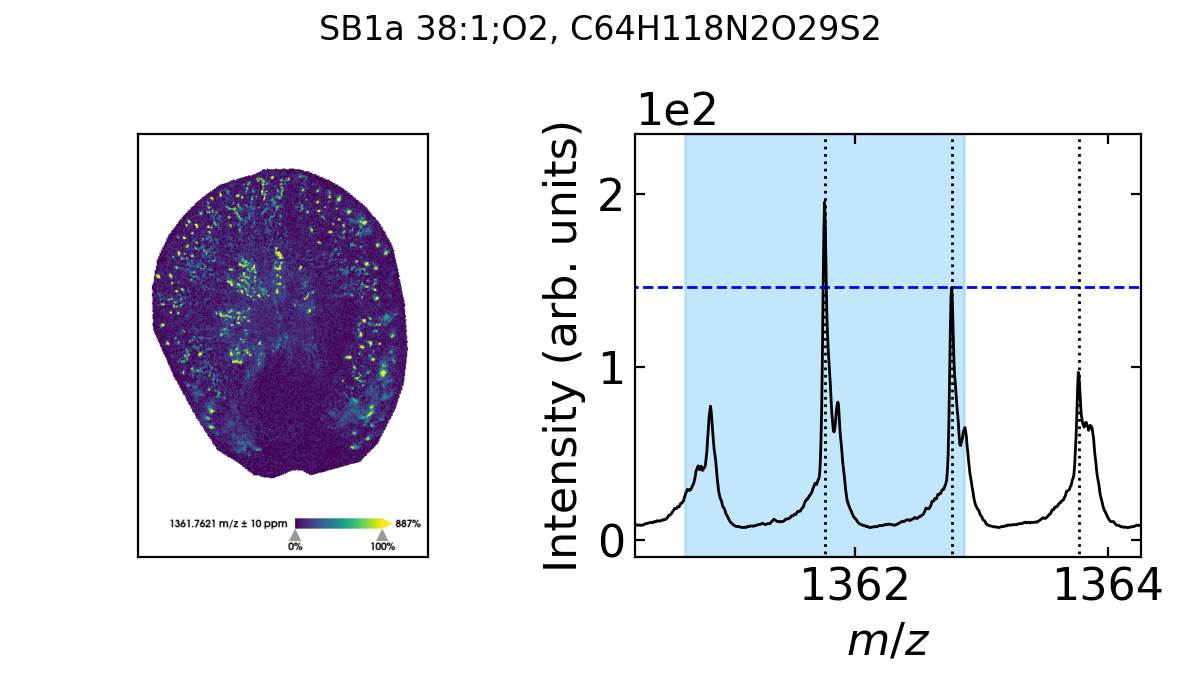

Supplement: Supplementary file 3 — Supplementary Data 1 [file 41467_2025_59839_MOESM3_ESM.zip › Suppl_Dataset_1_REV/qTOF_data1_slide1_python/1361.762077_qTOF_60w_1.png]

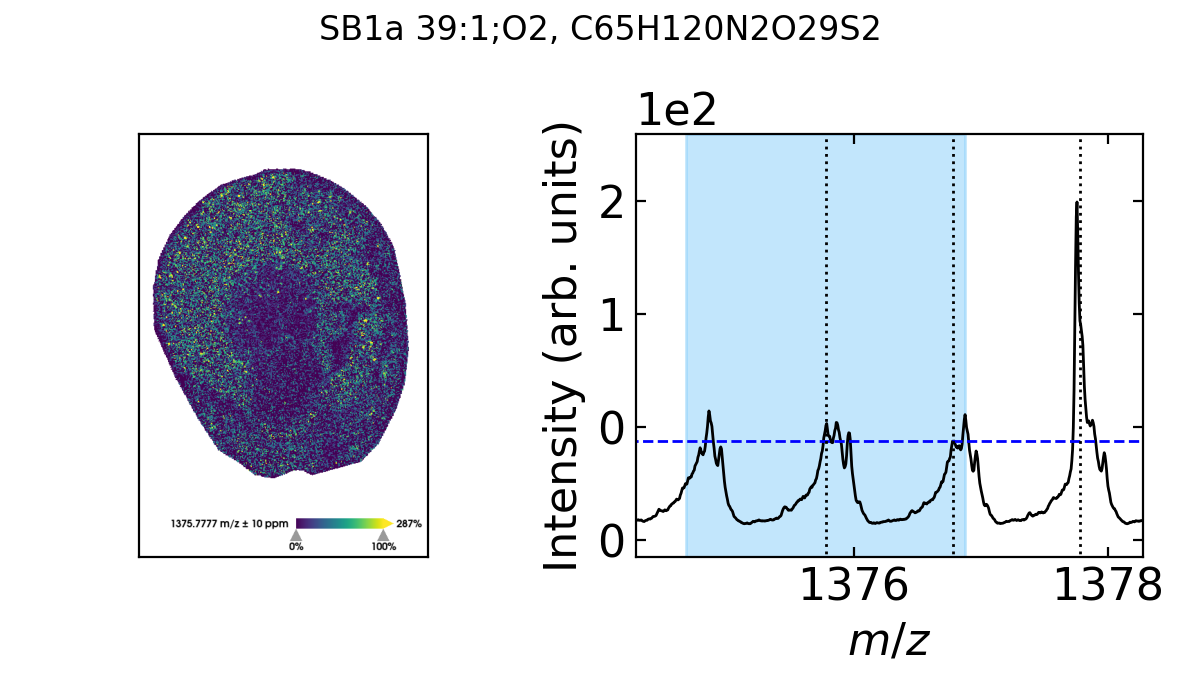

Supplement: Supplementary file 3 — Supplementary Data 1 [file 41467_2025_59839_MOESM3_ESM.zip › Suppl_Dataset_1_REV/qTOF_data1_slide1_python/1375.777727_qTOF_60w_1.png]

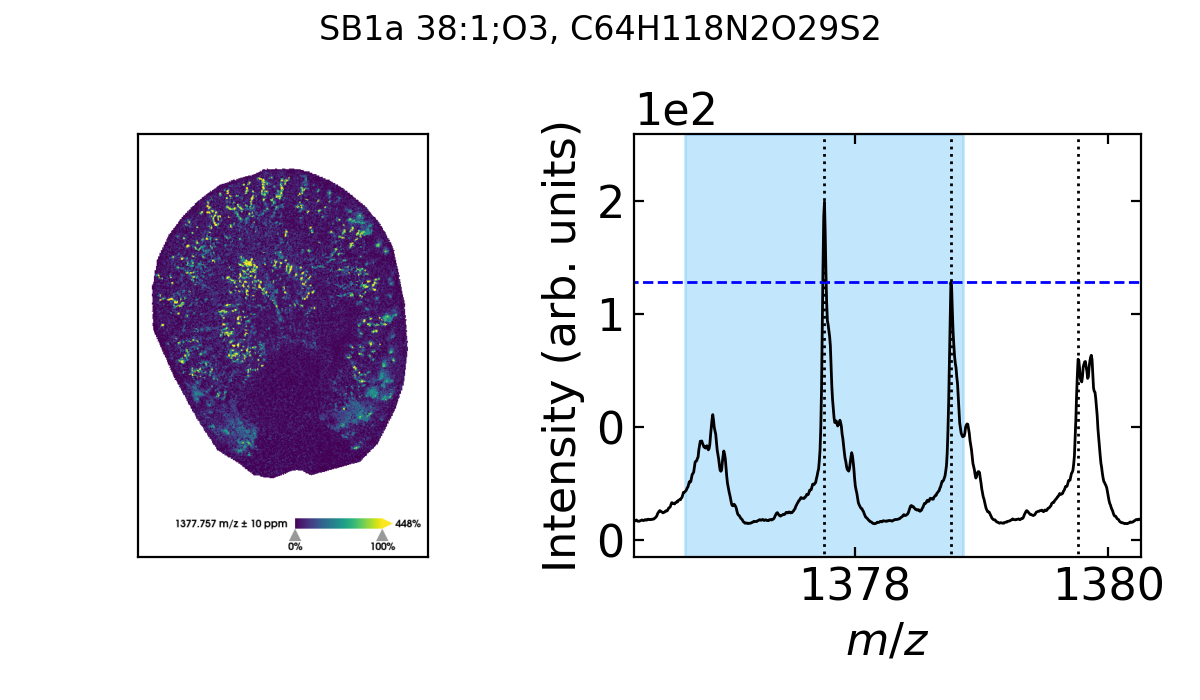

Supplement: Supplementary file 3 — Supplementary Data 1 [file 41467_2025_59839_MOESM3_ESM.zip › Suppl_Dataset_1_REV/qTOF_data1_slide1_python/1377.756991_qTOF_60w_1.png]

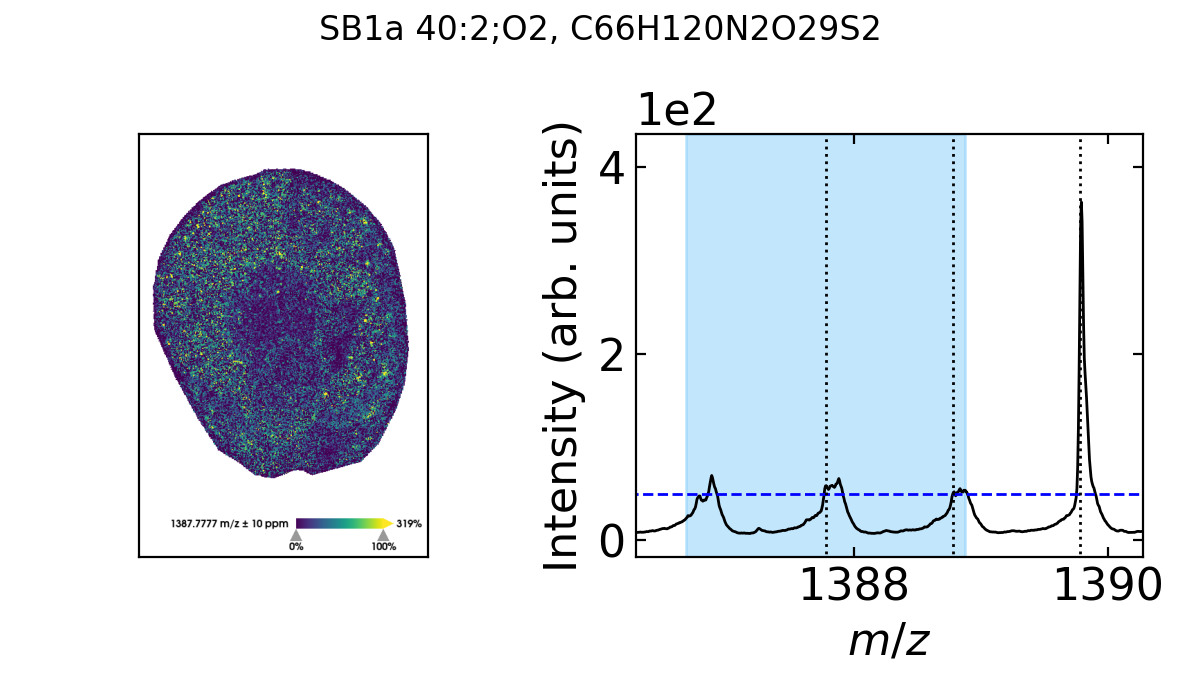

Supplement: Supplementary file 3 — Supplementary Data 1 [file 41467_2025_59839_MOESM3_ESM.zip › Suppl_Dataset_1_REV/qTOF_data1_slide1_python/1387.777727_qTOF_60w_1.png]

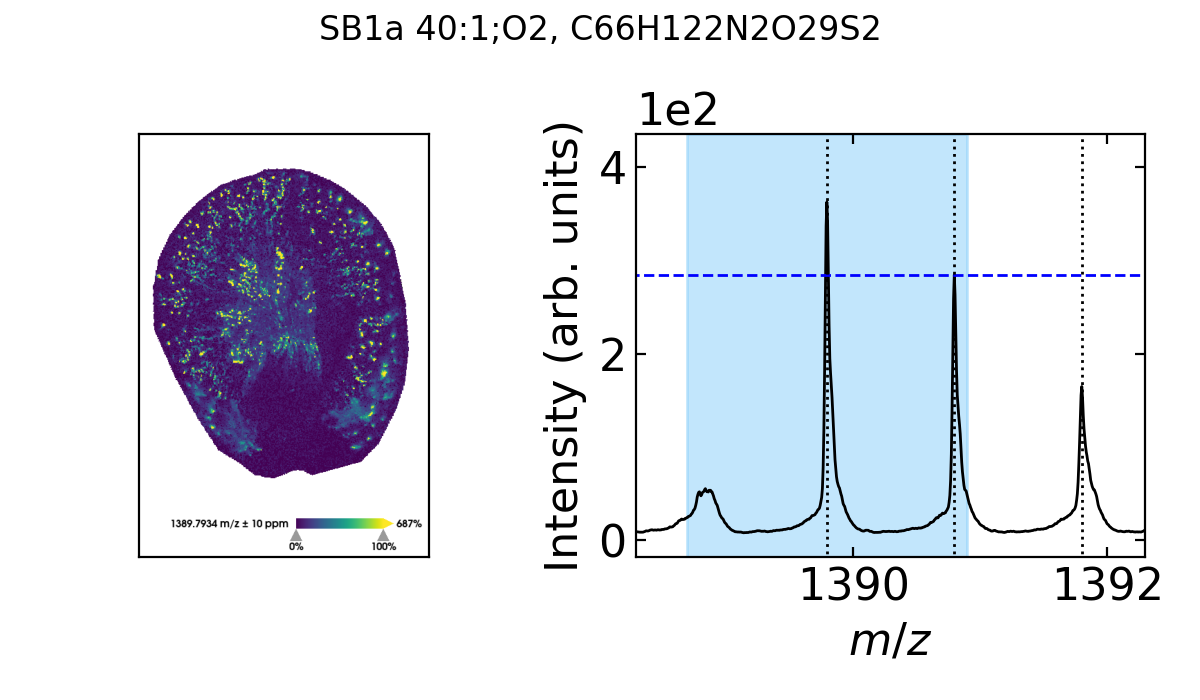

Supplement: Supplementary file 3 — Supplementary Data 1 [file 41467_2025_59839_MOESM3_ESM.zip › Suppl_Dataset_1_REV/qTOF_data1_slide1_python/1389.793377_qTOF_60w_1.png]

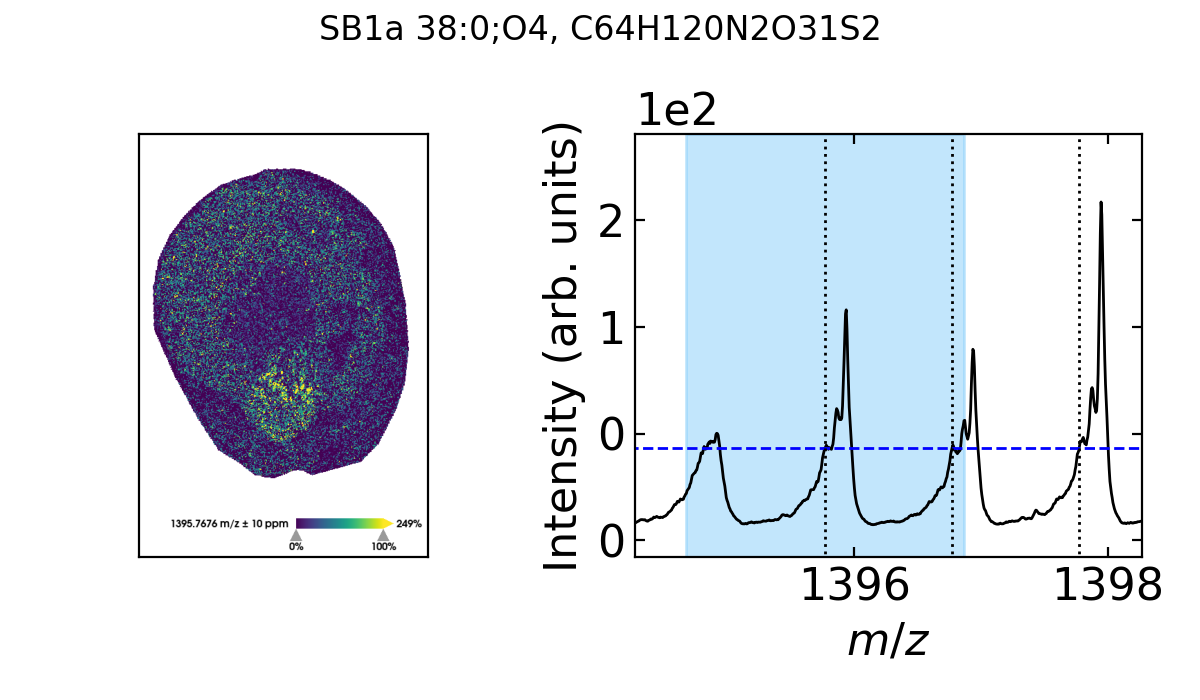

Supplement: Supplementary file 3 — Supplementary Data 1 [file 41467_2025_59839_MOESM3_ESM.zip › Suppl_Dataset_1_REV/qTOF_data1_slide1_python/1395.767556_qTOF_60w_1.png]

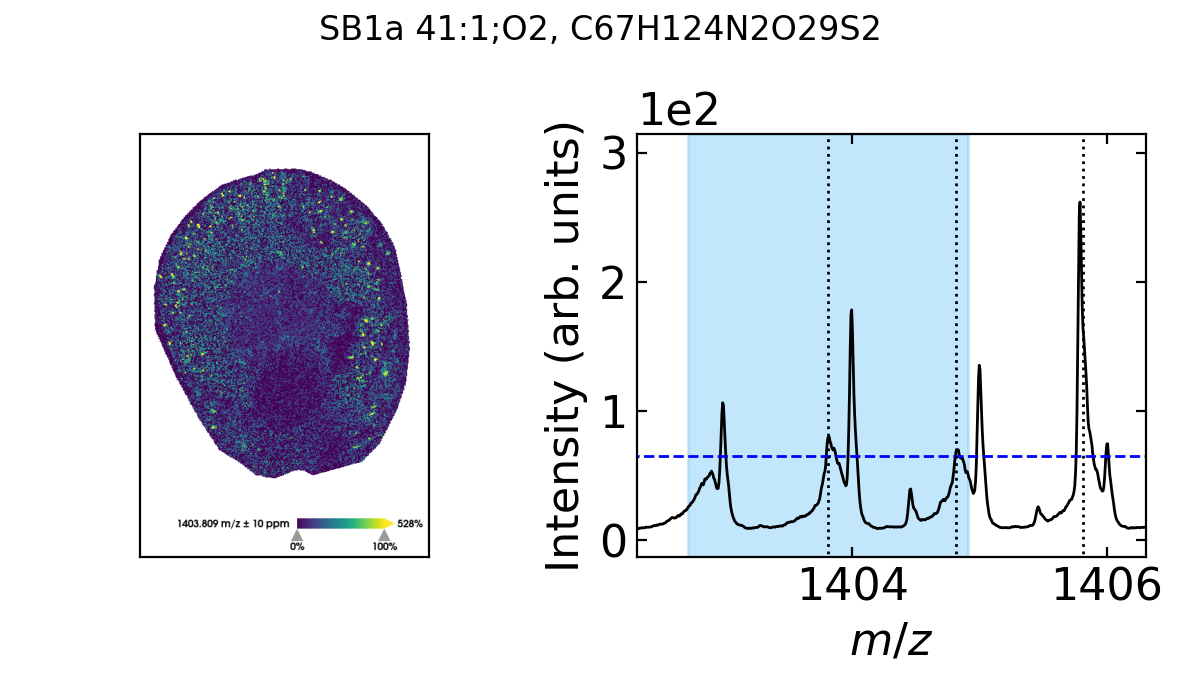

Supplement: Supplementary file 3 — Supplementary Data 1 [file 41467_2025_59839_MOESM3_ESM.zip › Suppl_Dataset_1_REV/qTOF_data1_slide1_python/1403.809027_qTOF_60w_1.png]

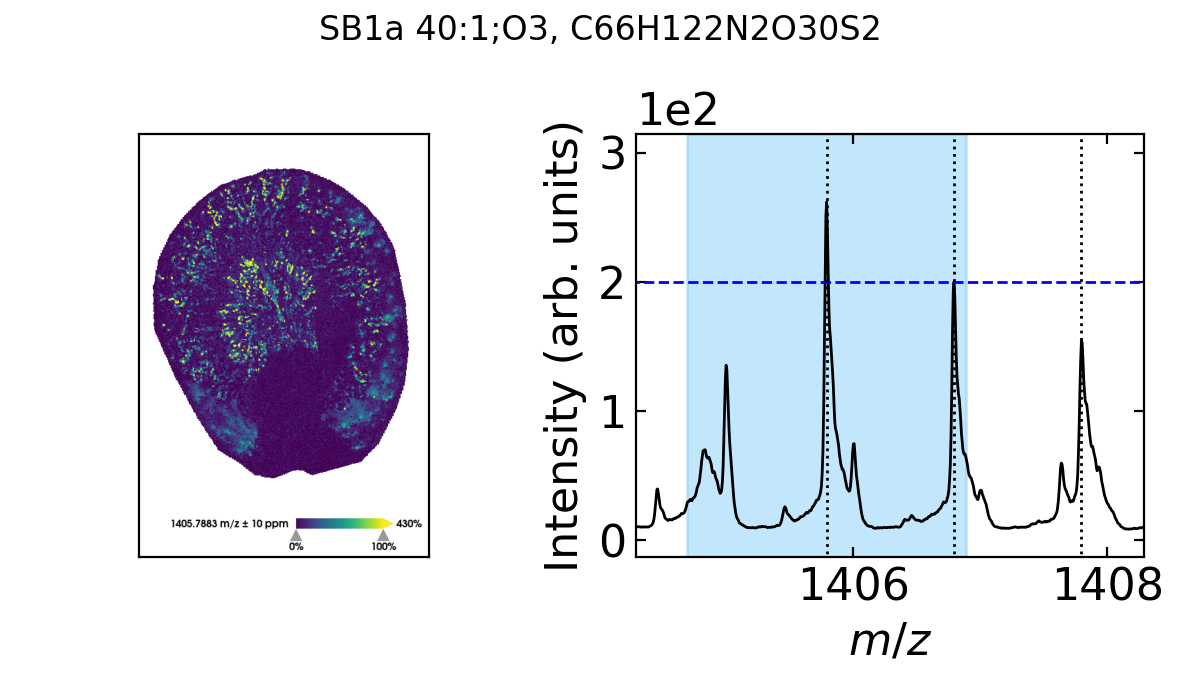

Supplement: Supplementary file 3 — Supplementary Data 1 [file 41467_2025_59839_MOESM3_ESM.zip › Suppl_Dataset_1_REV/qTOF_data1_slide1_python/1405.788292_qTOF_60w_1.png]

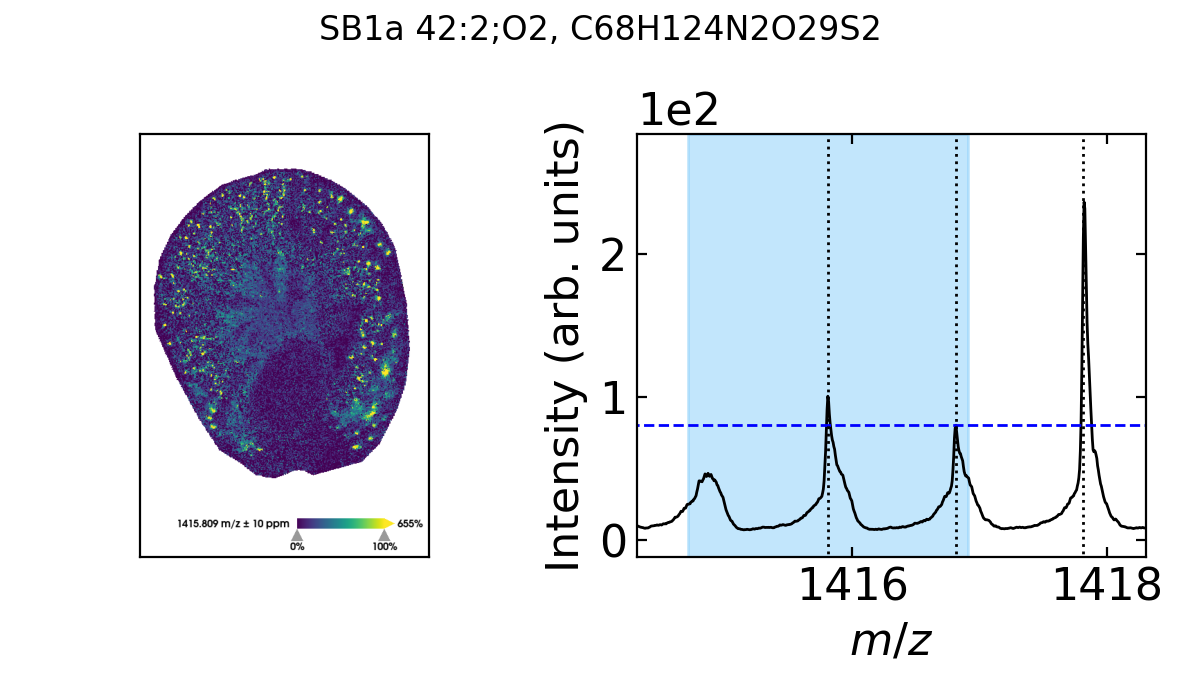

Supplement: Supplementary file 3 — Supplementary Data 1 [file 41467_2025_59839_MOESM3_ESM.zip › Suppl_Dataset_1_REV/qTOF_data1_slide1_python/1415.809027_qTOF_60w_1.png]

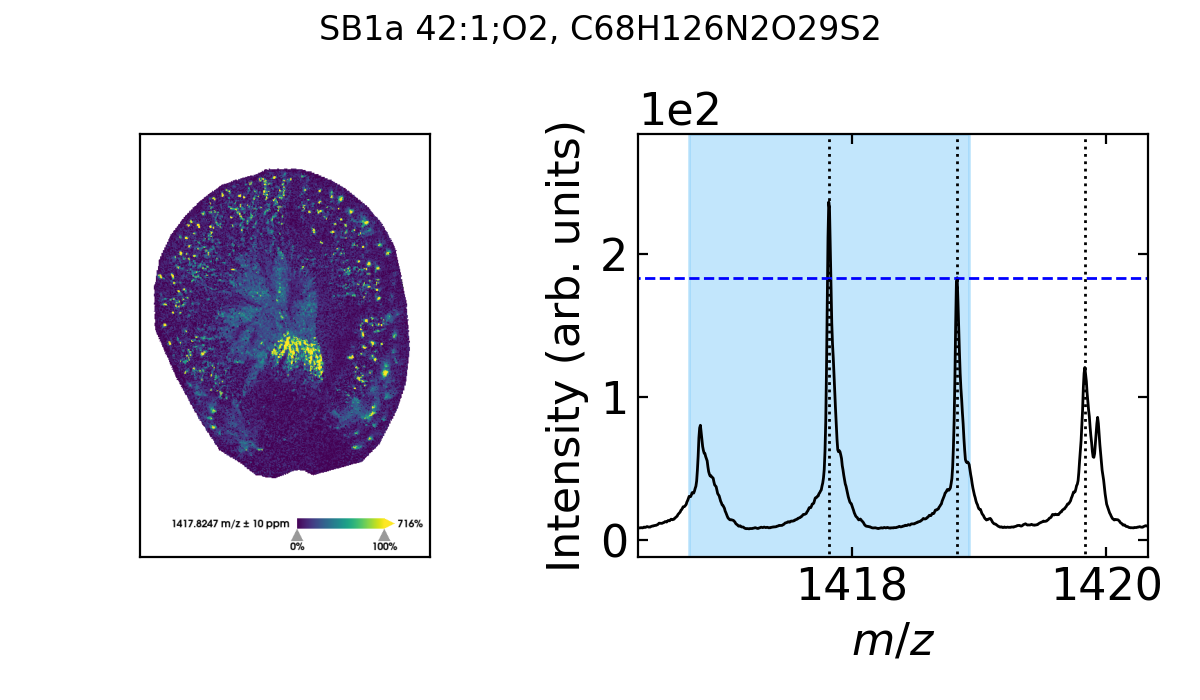

Supplement: Supplementary file 3 — Supplementary Data 1 [file 41467_2025_59839_MOESM3_ESM.zip › Suppl_Dataset_1_REV/qTOF_data1_slide1_python/1417.824677_qTOF_60w_1.png]

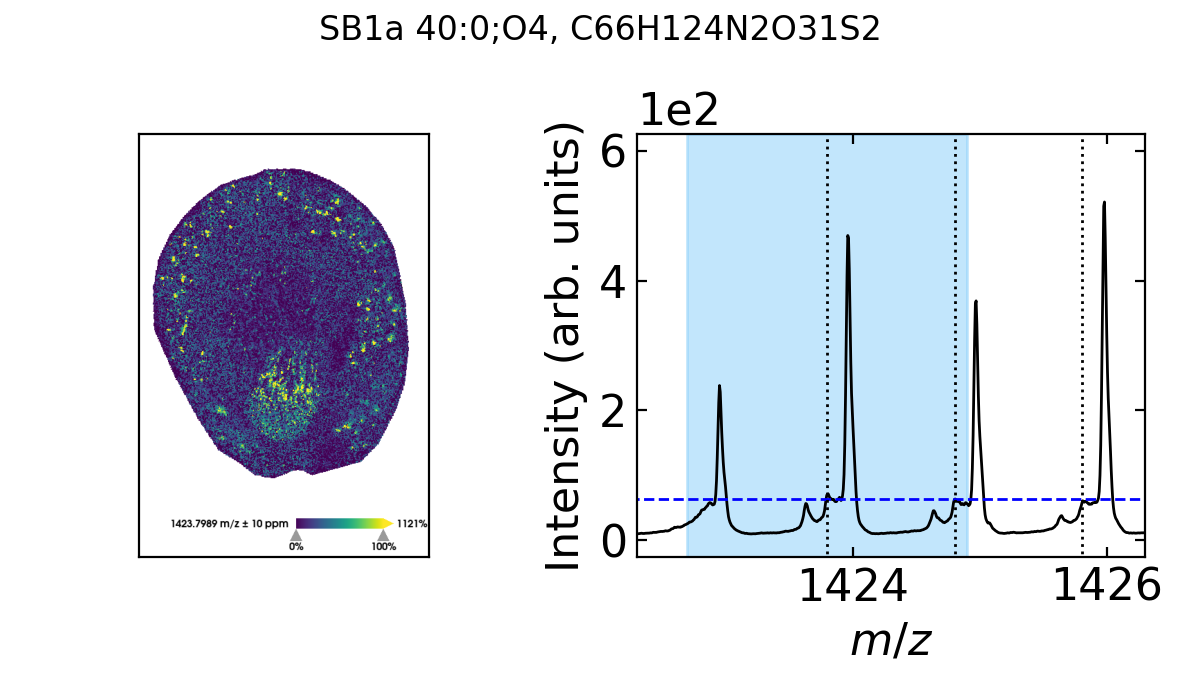

Supplement: Supplementary file 3 — Supplementary Data 1 [file 41467_2025_59839_MOESM3_ESM.zip › Suppl_Dataset_1_REV/qTOF_data1_slide1_python/1423.798857_qTOF_60w_1.png]

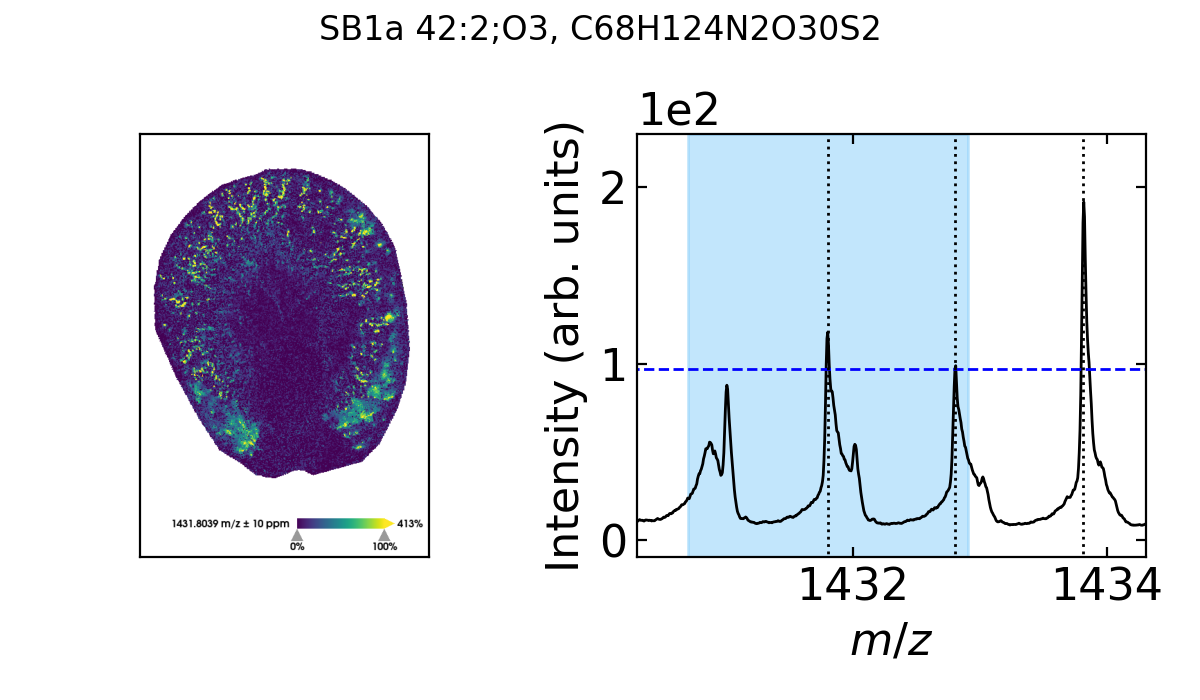

Supplement: Supplementary file 3 — Supplementary Data 1 [file 41467_2025_59839_MOESM3_ESM.zip › Suppl_Dataset_1_REV/qTOF_data1_slide1_python/1431.803942_qTOF_60w_1.png]

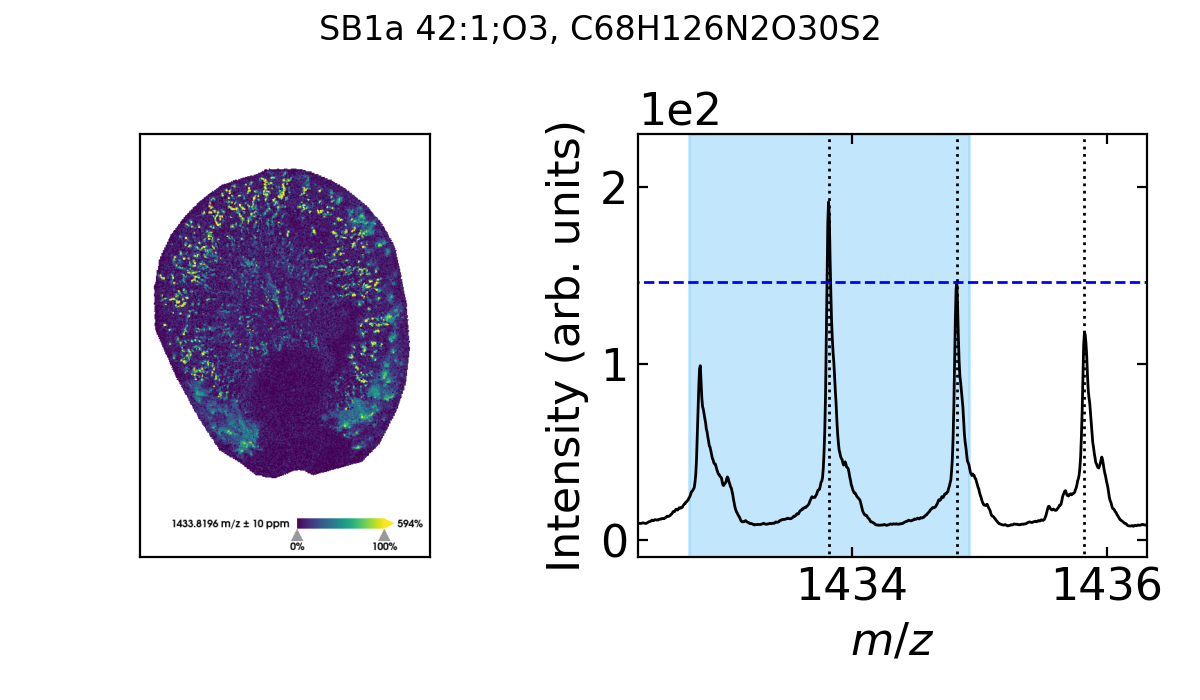

Supplement: Supplementary file 3 — Supplementary Data 1 [file 41467_2025_59839_MOESM3_ESM.zip › Suppl_Dataset_1_REV/qTOF_data1_slide1_python/1433.819592_qTOF_60w_1.png]

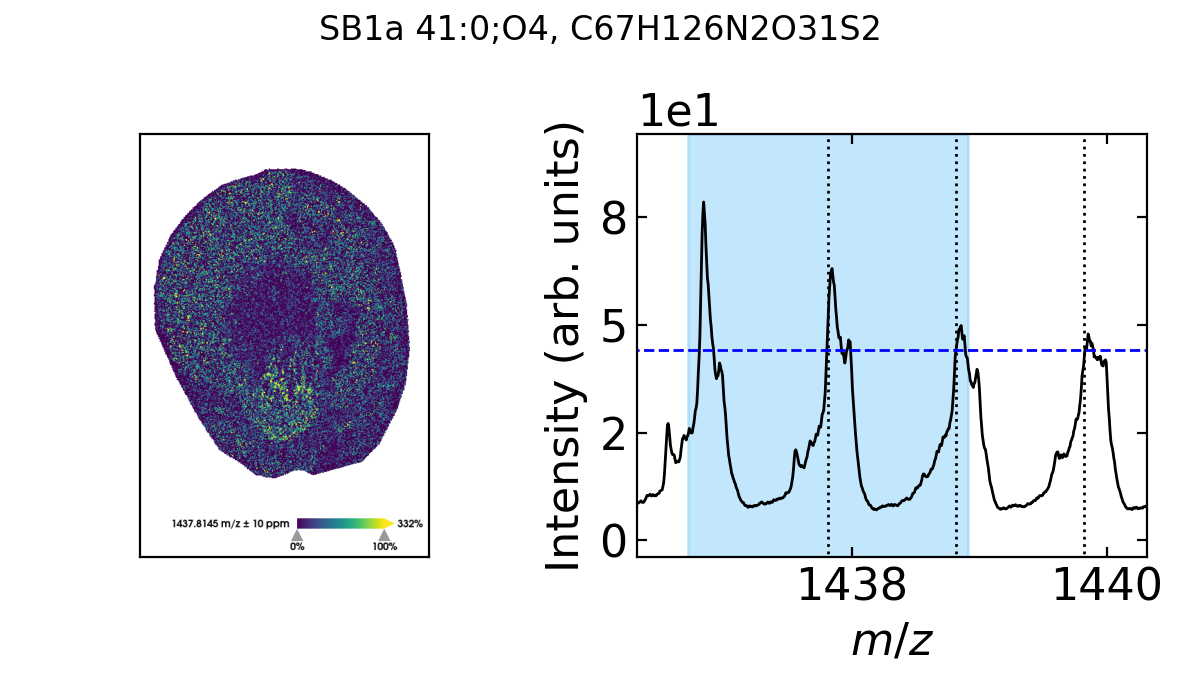

Supplement: Supplementary file 3 — Supplementary Data 1 [file 41467_2025_59839_MOESM3_ESM.zip › Suppl_Dataset_1_REV/qTOF_data1_slide1_python/1437.814507_qTOF_60w_1.png]

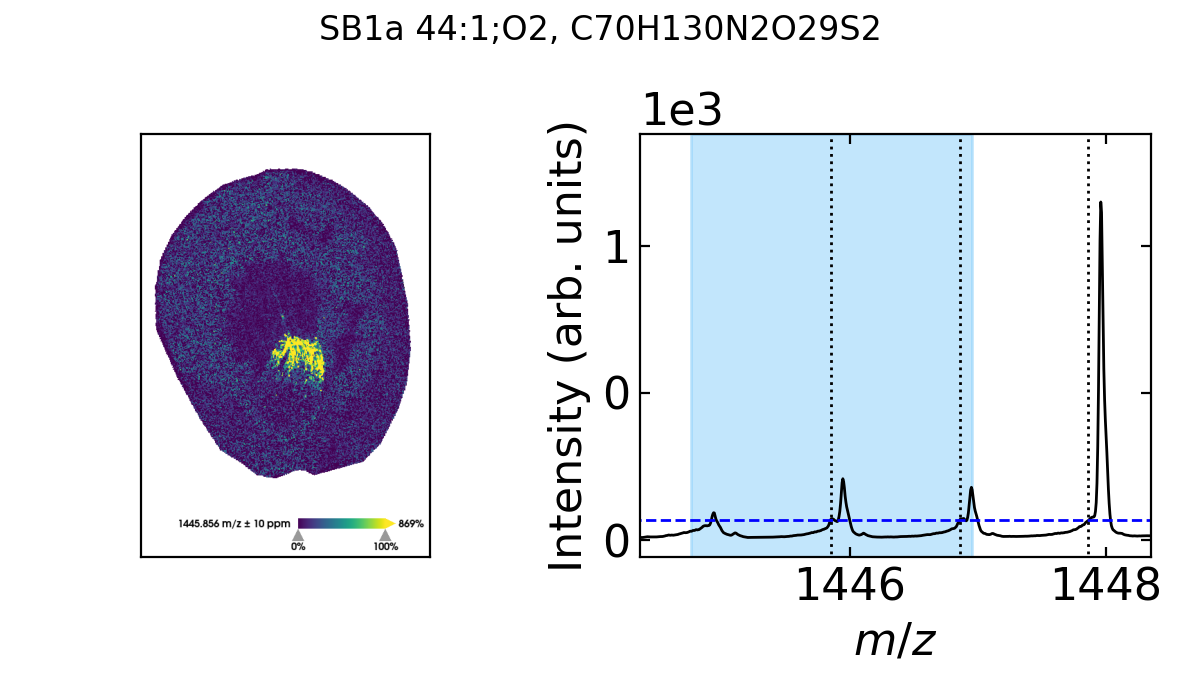

Supplement: Supplementary file 3 — Supplementary Data 1 [file 41467_2025_59839_MOESM3_ESM.zip › Suppl_Dataset_1_REV/qTOF_data1_slide1_python/1445.855977_qTOF_60w_1.png]

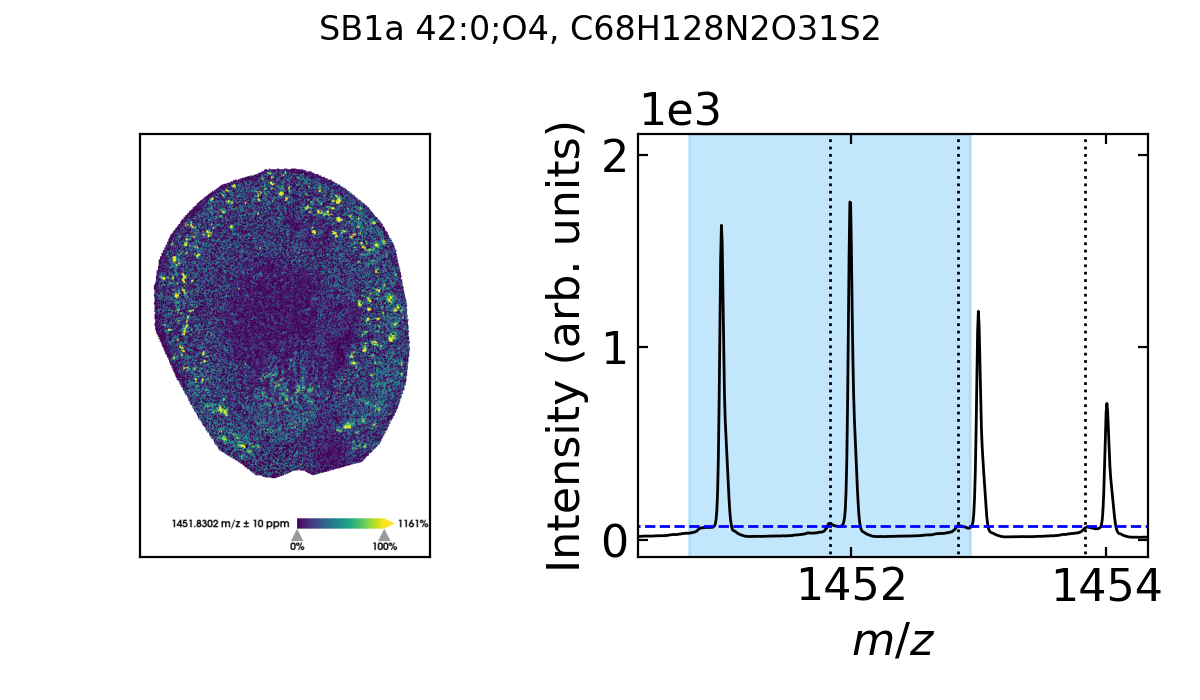

Supplement: Supplementary file 3 — Supplementary Data 1 [file 41467_2025_59839_MOESM3_ESM.zip › Suppl_Dataset_1_REV/qTOF_data1_slide1_python/1451.830157_qTOF_60w_1.png]

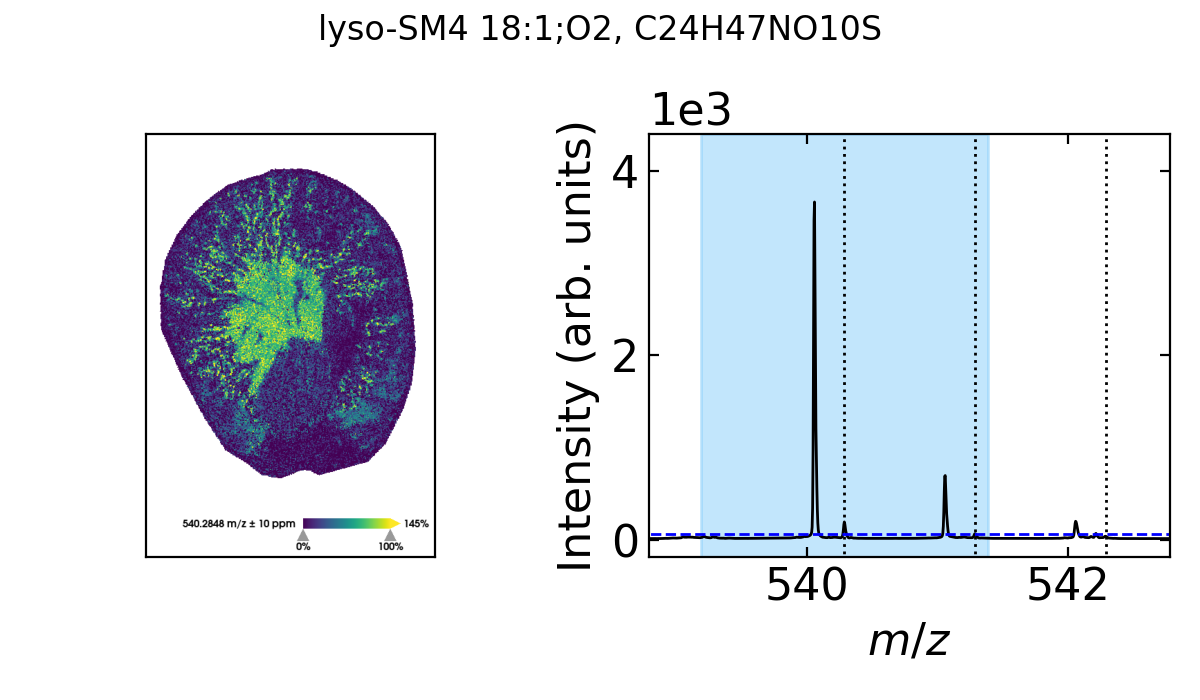

Supplement: Supplementary file 3 — Supplementary Data 1 [file 41467_2025_59839_MOESM3_ESM.zip › Suppl_Dataset_1_REV/qTOF_data1_slide1_python/540.284791_qTOF_60w_1.png]

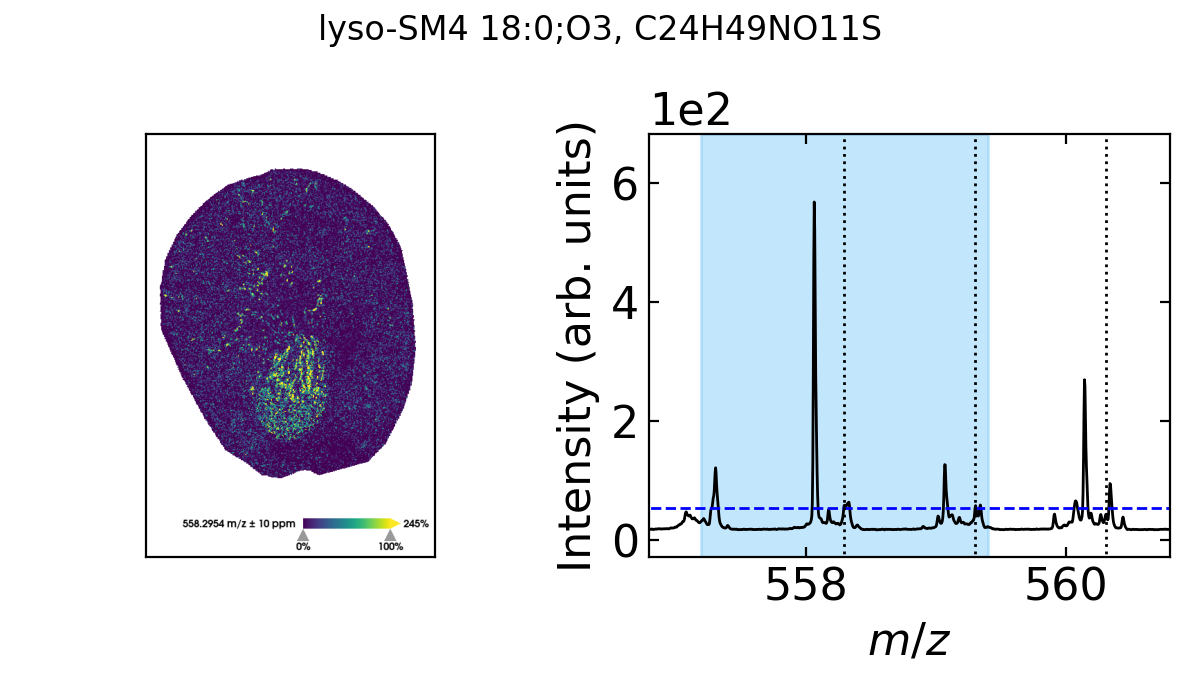

Supplement: Supplementary file 3 — Supplementary Data 1 [file 41467_2025_59839_MOESM3_ESM.zip › Suppl_Dataset_1_REV/qTOF_data1_slide1_python/558.295356_qTOF_60w_1.png]

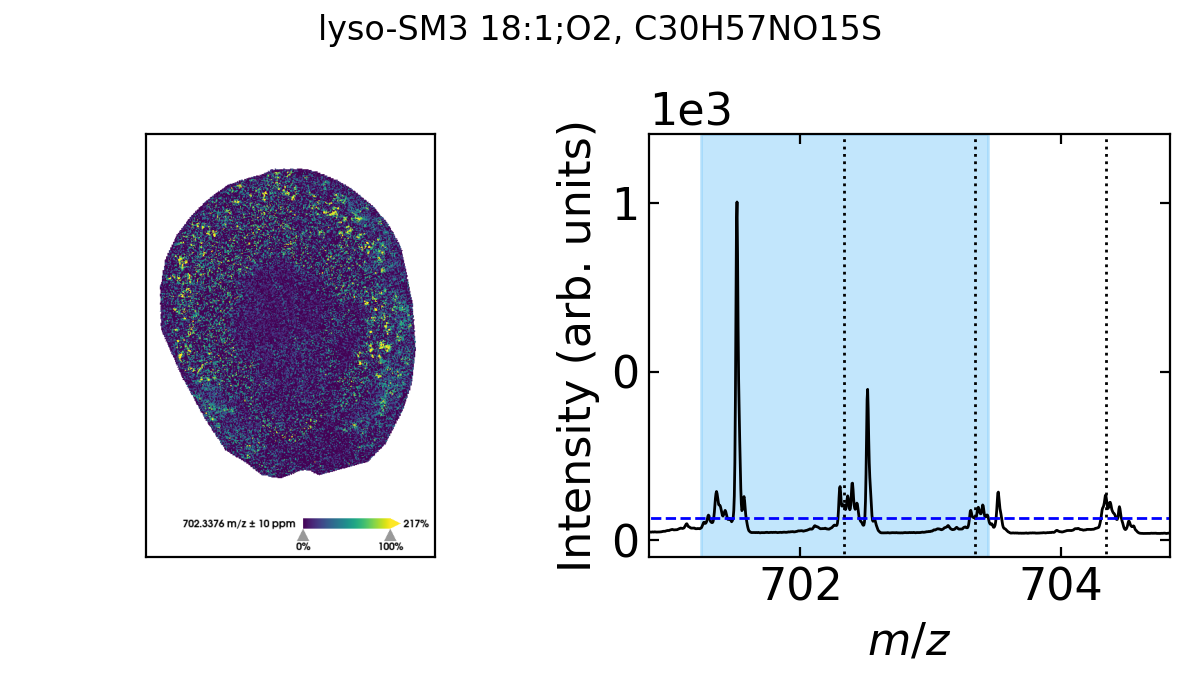

Supplement: Supplementary file 3 — Supplementary Data 1 [file 41467_2025_59839_MOESM3_ESM.zip › Suppl_Dataset_1_REV/qTOF_data1_slide1_python/702.337615_qTOF_60w_1.png]

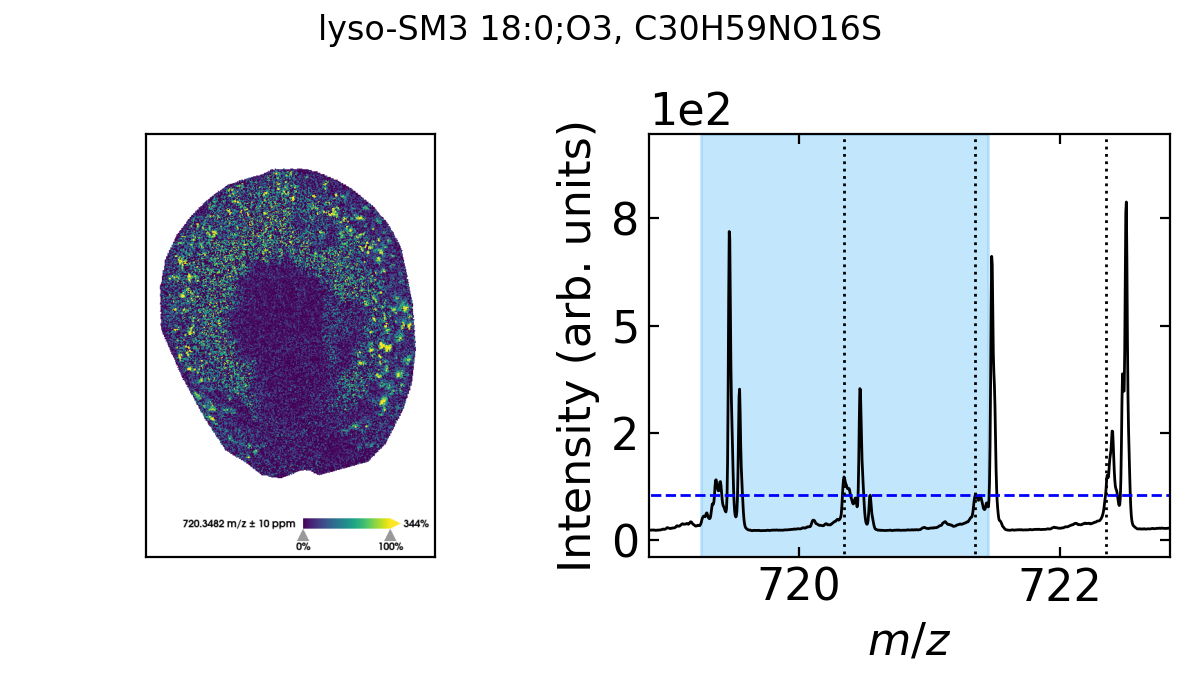

Supplement: Supplementary file 3 — Supplementary Data 1 [file 41467_2025_59839_MOESM3_ESM.zip › Suppl_Dataset_1_REV/qTOF_data1_slide1_python/720.34818_qTOF_60w_1.png]

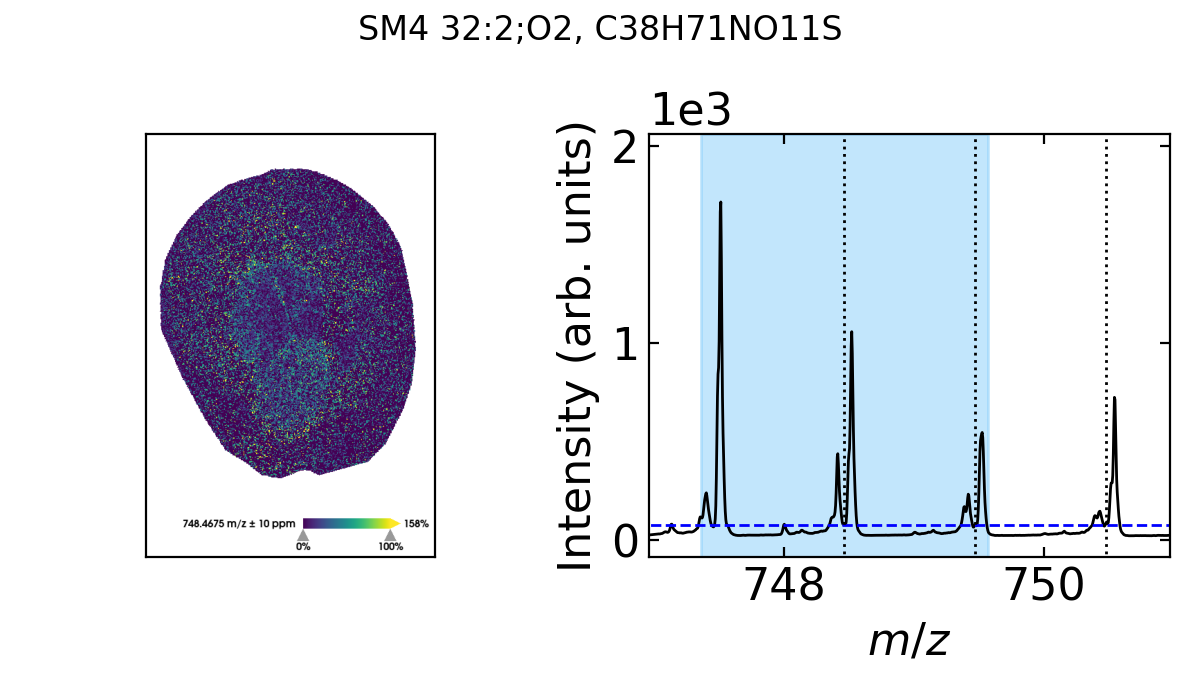

Supplement: Supplementary file 3 — Supplementary Data 1 [file 41467_2025_59839_MOESM3_ESM.zip › Suppl_Dataset_1_REV/qTOF_data1_slide1_python/748.467507_qTOF_60w_1.png]

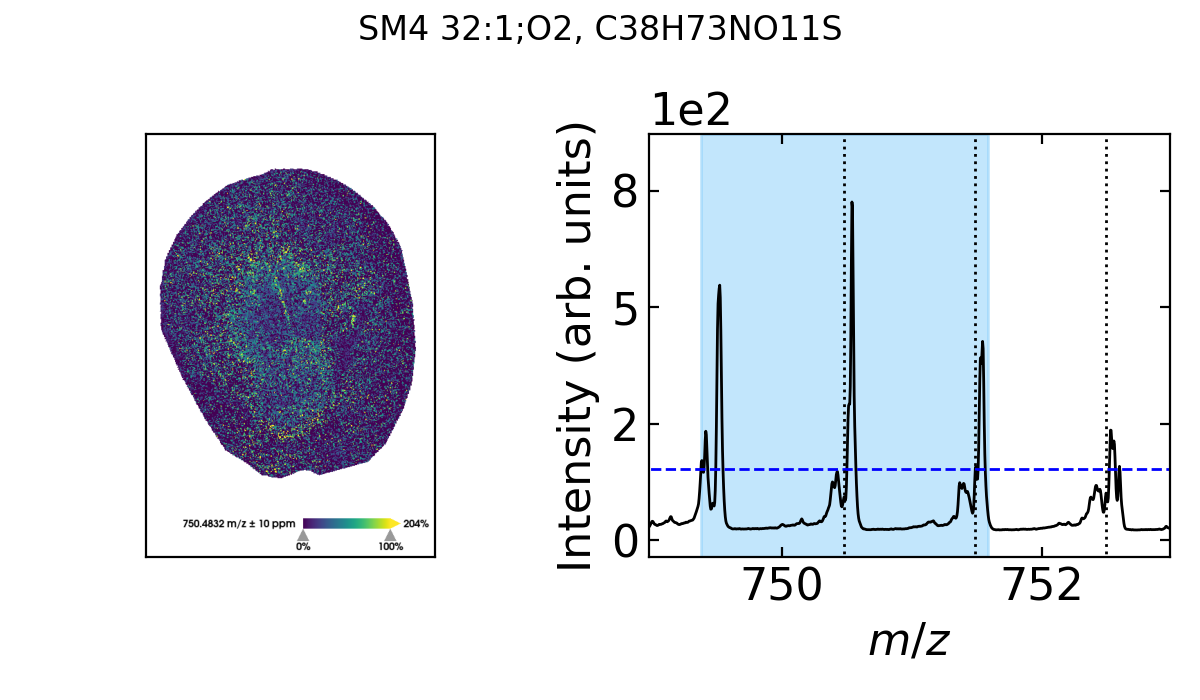

Supplement: Supplementary file 3 — Supplementary Data 1 [file 41467_2025_59839_MOESM3_ESM.zip › Suppl_Dataset_1_REV/qTOF_data1_slide1_python/750.483157_qTOF_60w_1.png]

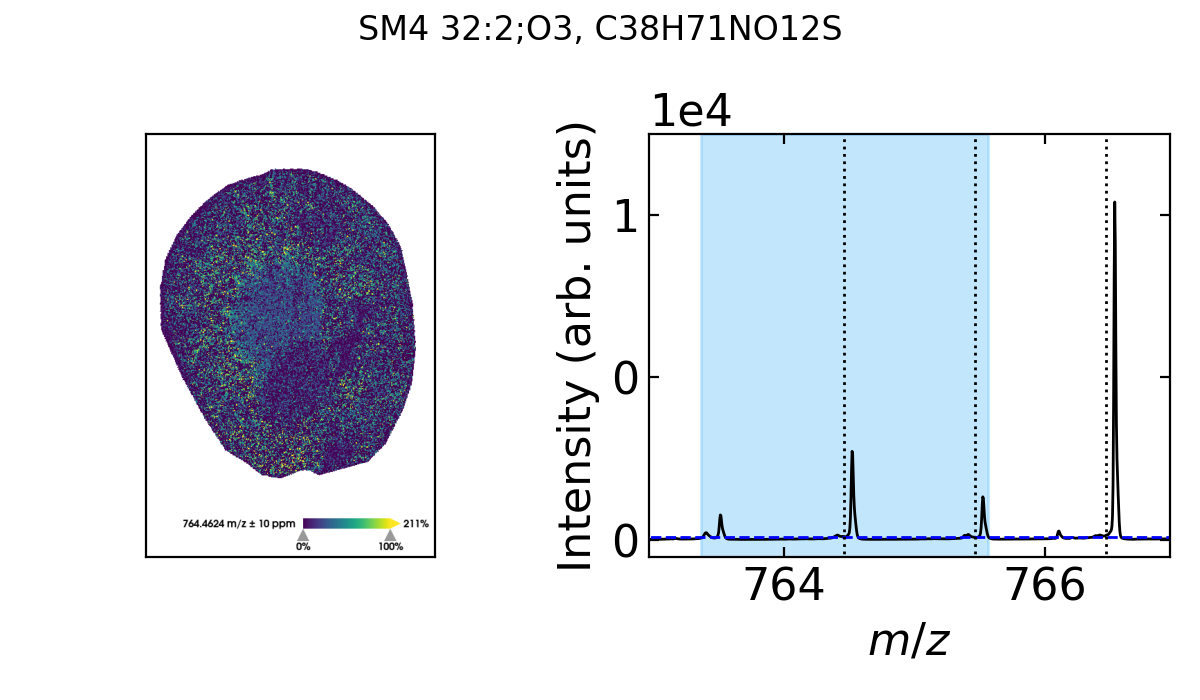

Supplement: Supplementary file 3 — Supplementary Data 1 [file 41467_2025_59839_MOESM3_ESM.zip › Suppl_Dataset_1_REV/qTOF_data1_slide1_python/764.462422_qTOF_60w_1.png]

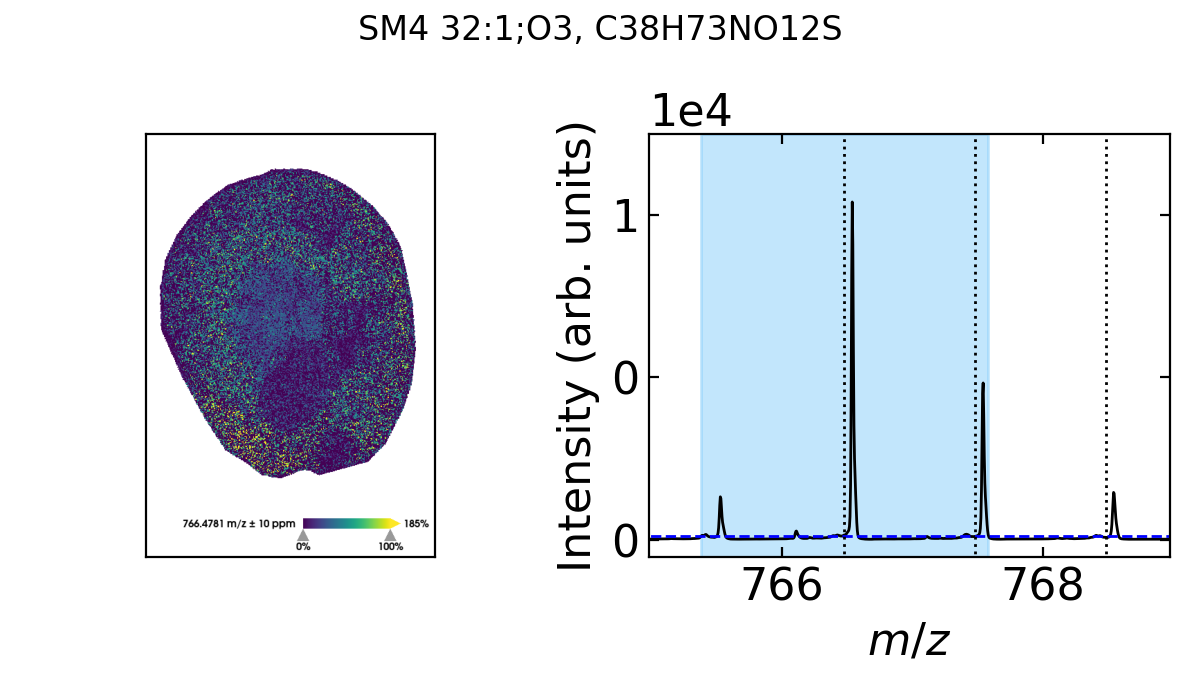

Supplement: Supplementary file 3 — Supplementary Data 1 [file 41467_2025_59839_MOESM3_ESM.zip › Suppl_Dataset_1_REV/qTOF_data1_slide1_python/766.478072_qTOF_60w_1.png]

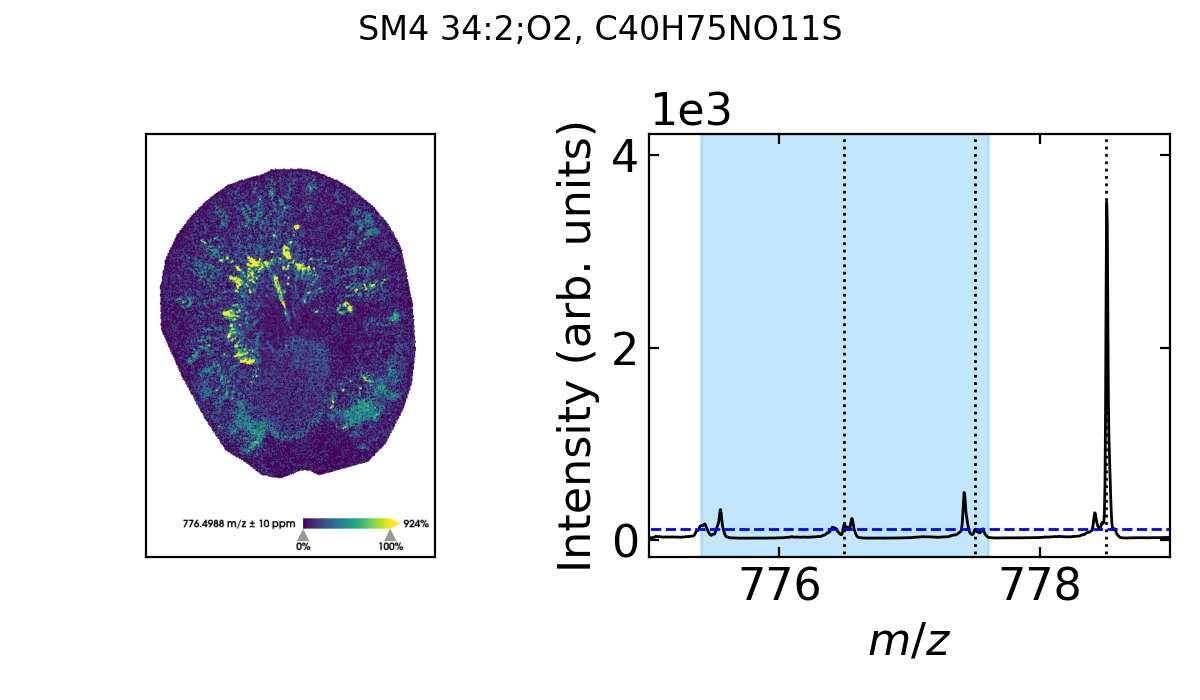

Supplement: Supplementary file 3 — Supplementary Data 1 [file 41467_2025_59839_MOESM3_ESM.zip › Suppl_Dataset_1_REV/qTOF_data1_slide1_python/776.498807_qTOF_60w_1.png]

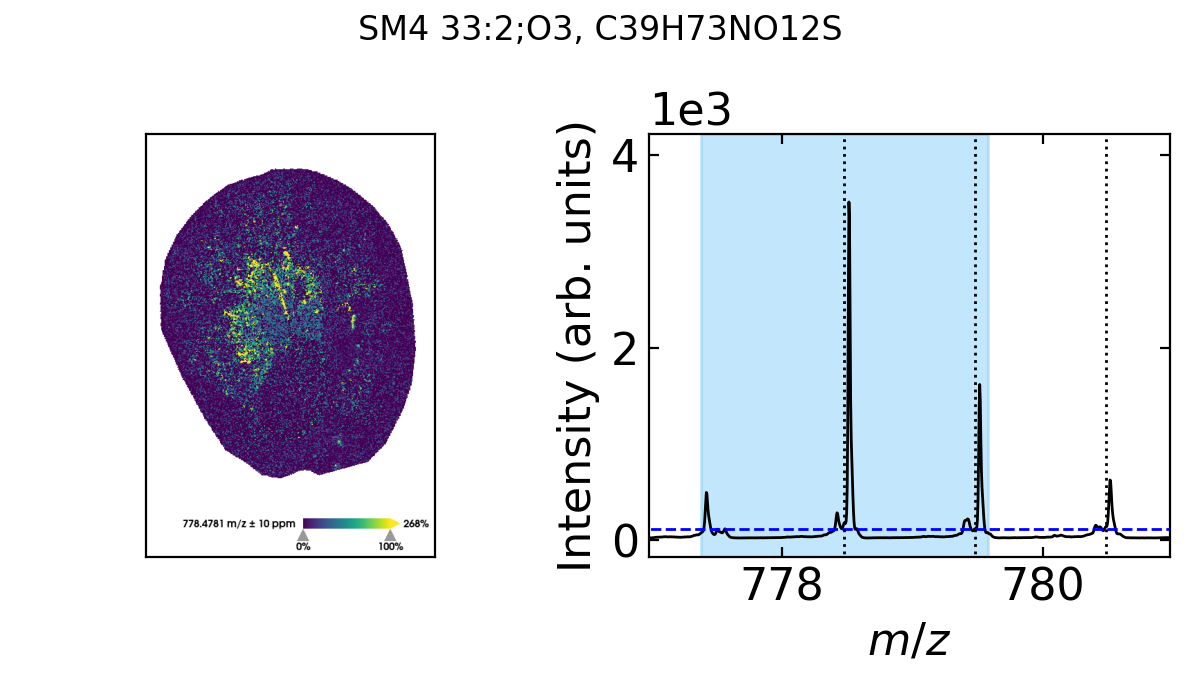

Supplement: Supplementary file 3 — Supplementary Data 1 [file 41467_2025_59839_MOESM3_ESM.zip › Suppl_Dataset_1_REV/qTOF_data1_slide1_python/778.478072_qTOF_60w_1.png]

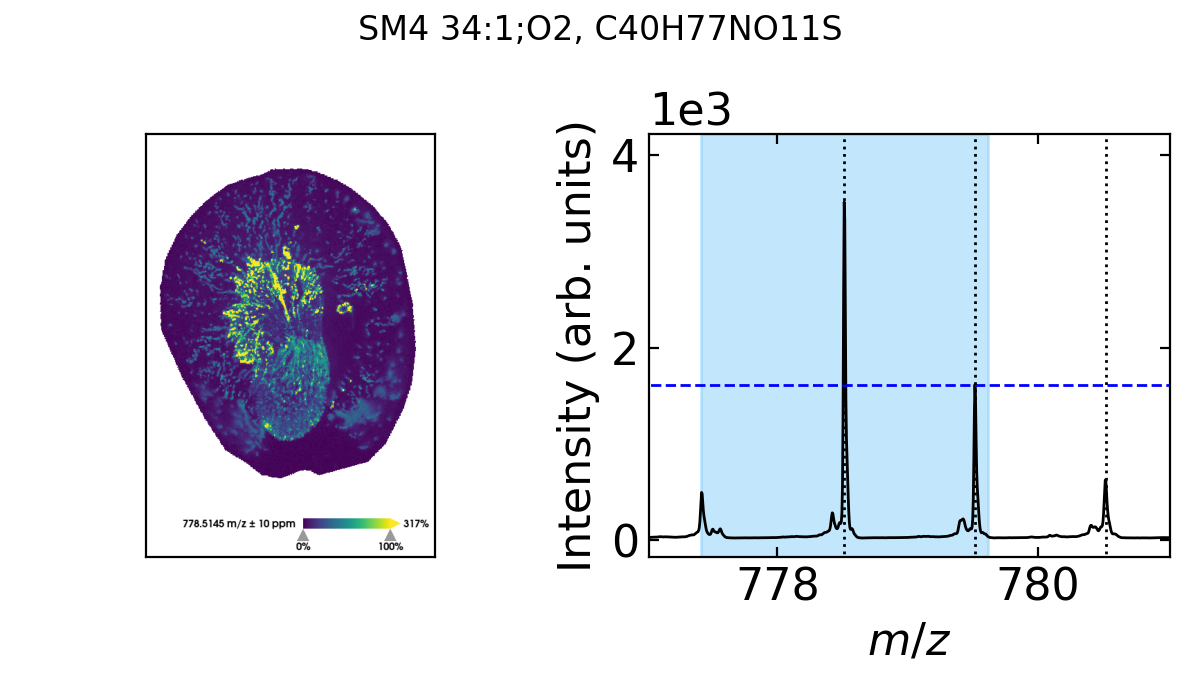

Supplement: Supplementary file 3 — Supplementary Data 1 [file 41467_2025_59839_MOESM3_ESM.zip › Suppl_Dataset_1_REV/qTOF_data1_slide1_python/778.514457_qTOF_60w_1.png]

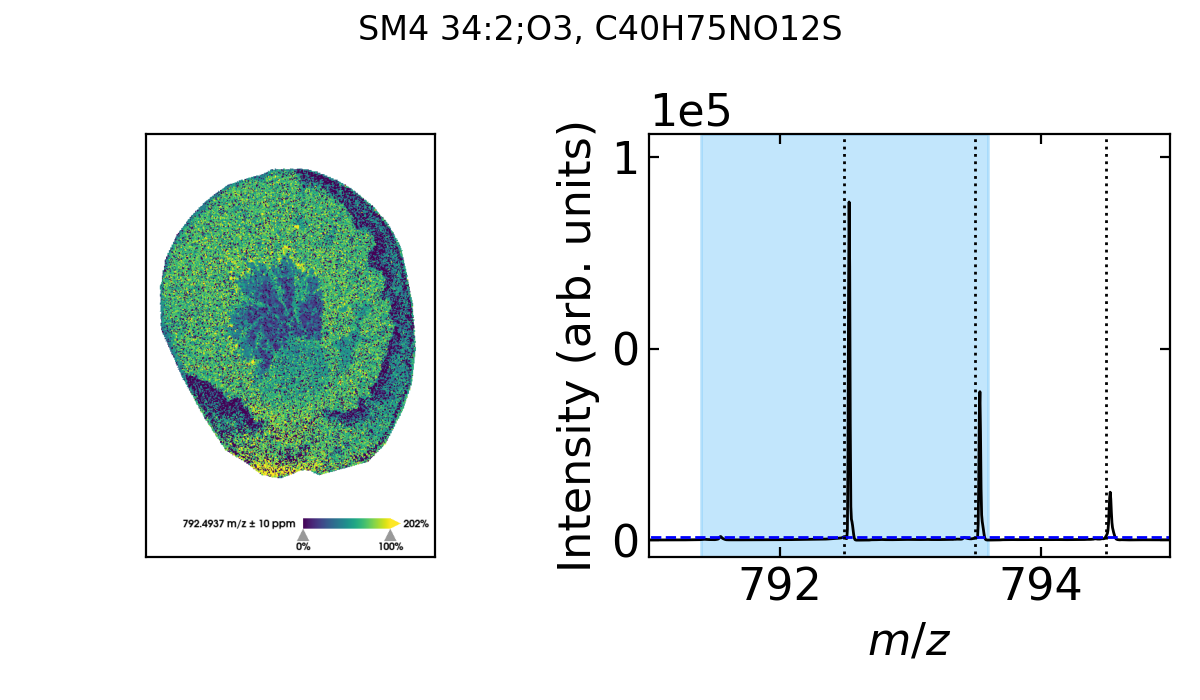

Supplement: Supplementary file 3 — Supplementary Data 1 [file 41467_2025_59839_MOESM3_ESM.zip › Suppl_Dataset_1_REV/qTOF_data1_slide1_python/792.493722_qTOF_60w_1.png]

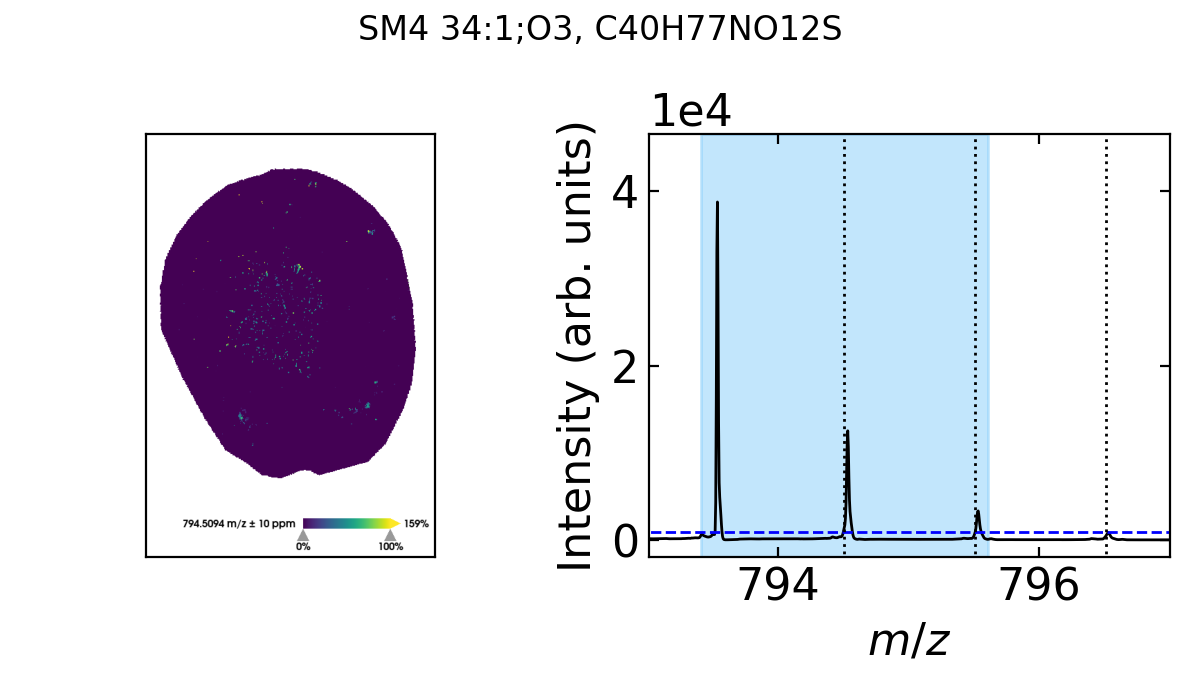

Supplement: Supplementary file 3 — Supplementary Data 1 [file 41467_2025_59839_MOESM3_ESM.zip › Suppl_Dataset_1_REV/qTOF_data1_slide1_python/794.509372_qTOF_60w_1.png]

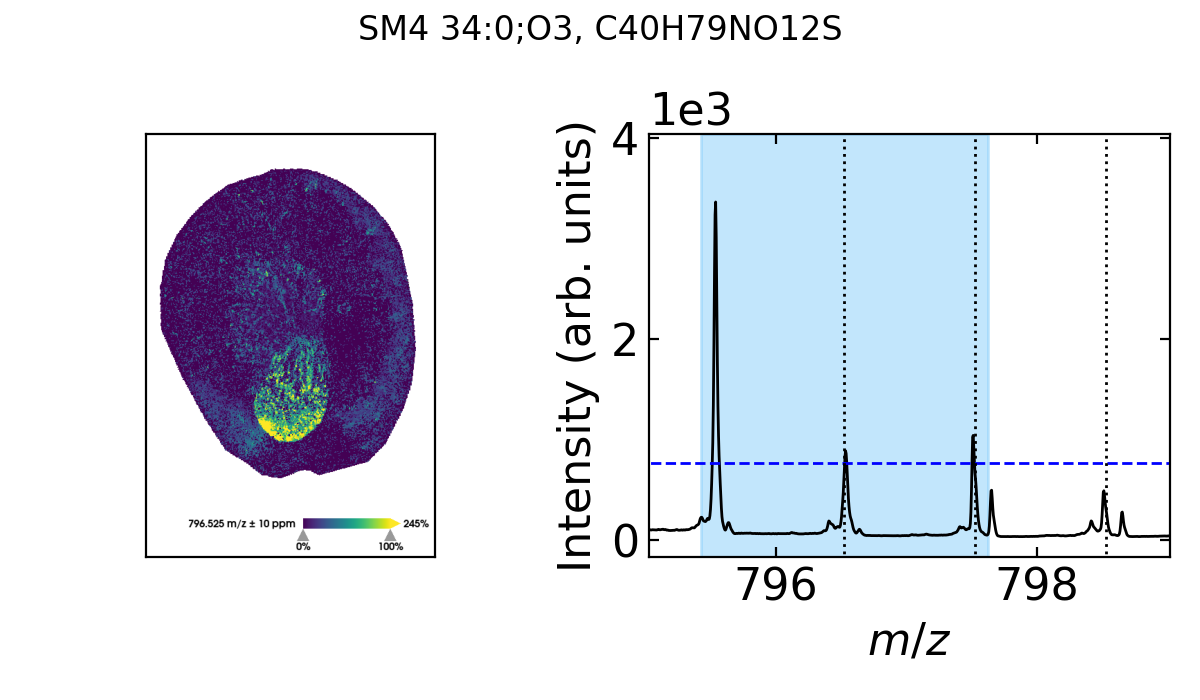

Supplement: Supplementary file 3 — Supplementary Data 1 [file 41467_2025_59839_MOESM3_ESM.zip › Suppl_Dataset_1_REV/qTOF_data1_slide1_python/796.525022_qTOF_60w_1.png]

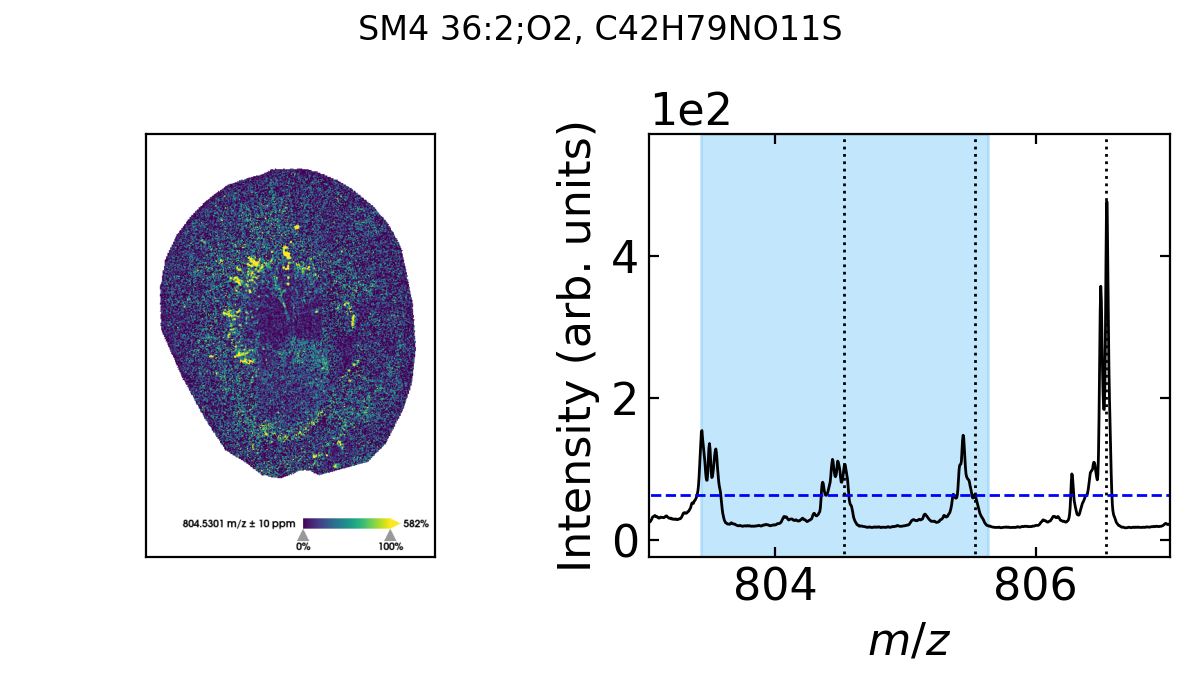

Supplement: Supplementary file 3 — Supplementary Data 1 [file 41467_2025_59839_MOESM3_ESM.zip › Suppl_Dataset_1_REV/qTOF_data1_slide1_python/804.530107_qTOF_60w_1.png]

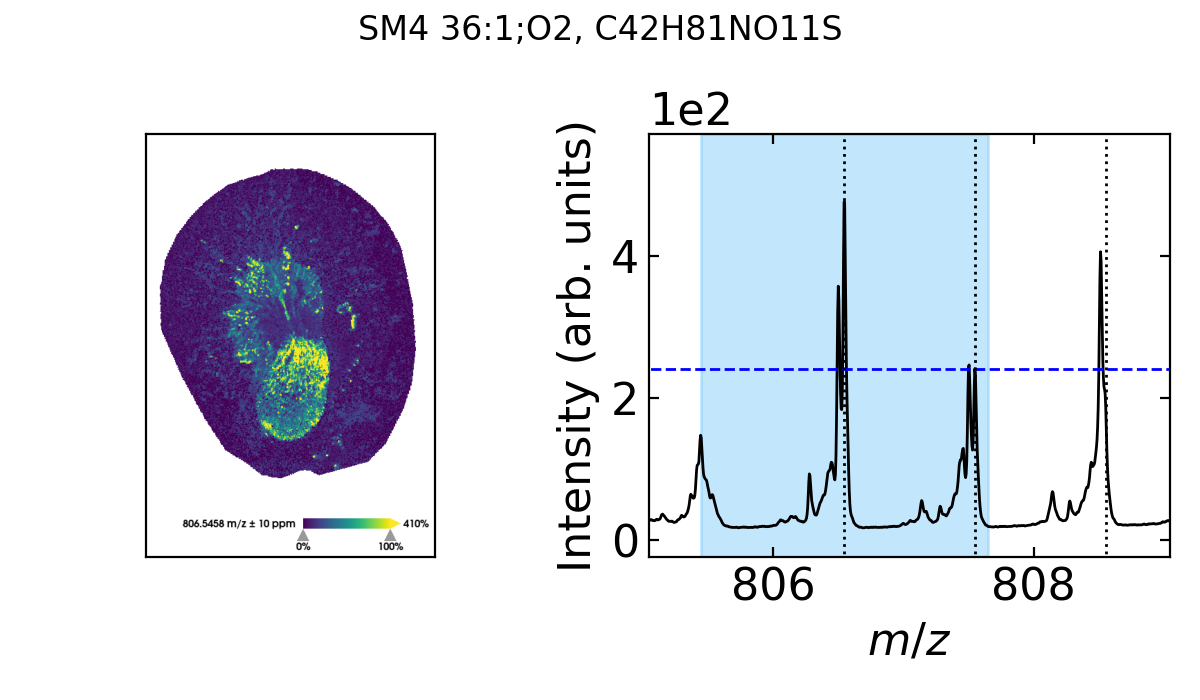

Supplement: Supplementary file 3 — Supplementary Data 1 [file 41467_2025_59839_MOESM3_ESM.zip › Suppl_Dataset_1_REV/qTOF_data1_slide1_python/806.545757_qTOF_60w_1.png]

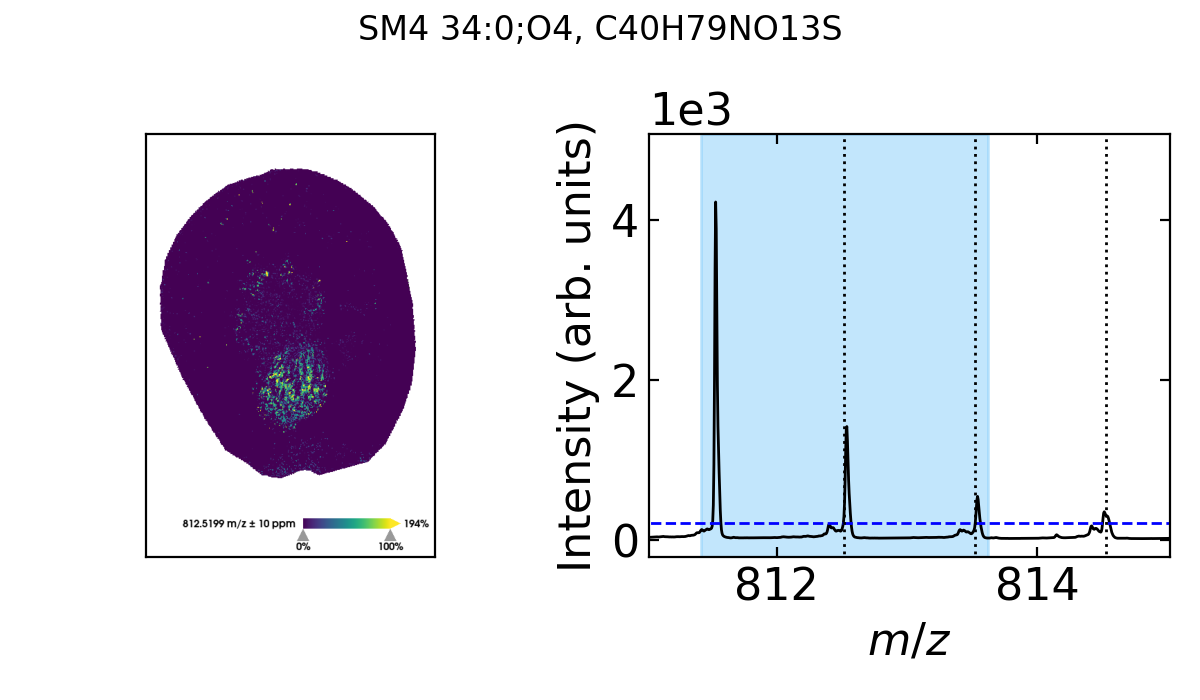

Supplement: Supplementary file 3 — Supplementary Data 1 [file 41467_2025_59839_MOESM3_ESM.zip › Suppl_Dataset_1_REV/qTOF_data1_slide1_python/812.519936_qTOF_60w_1.png]

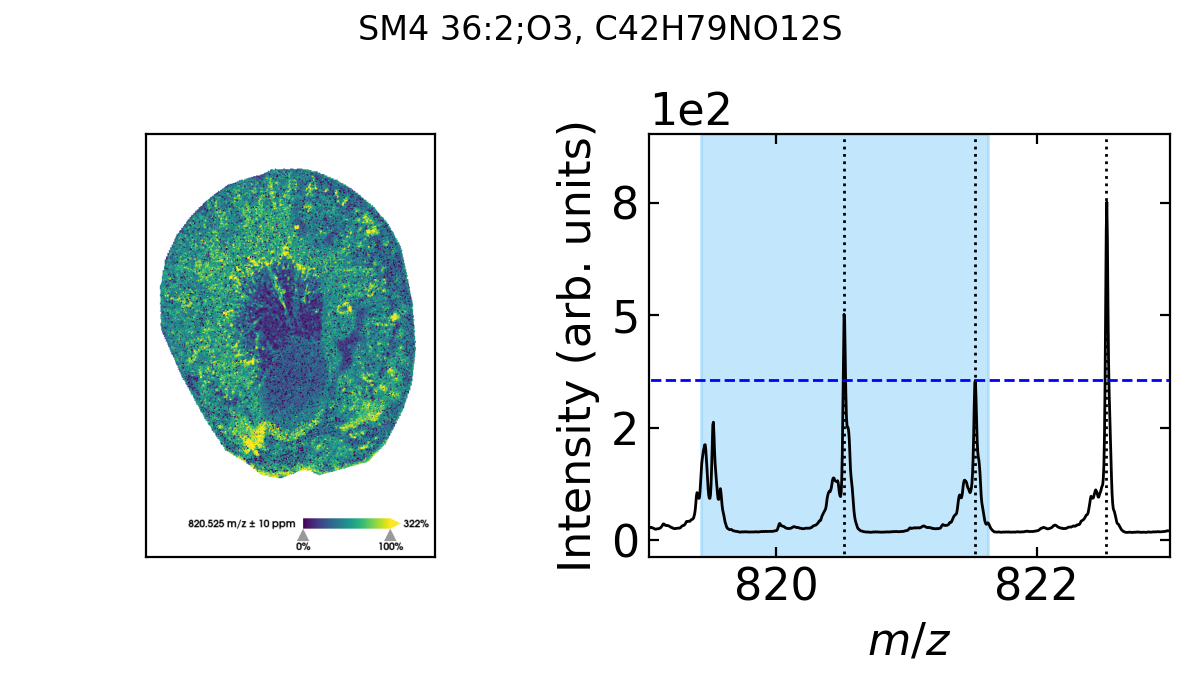

Supplement: Supplementary file 3 — Supplementary Data 1 [file 41467_2025_59839_MOESM3_ESM.zip › Suppl_Dataset_1_REV/qTOF_data1_slide1_python/820.525022_qTOF_60w_1.png]

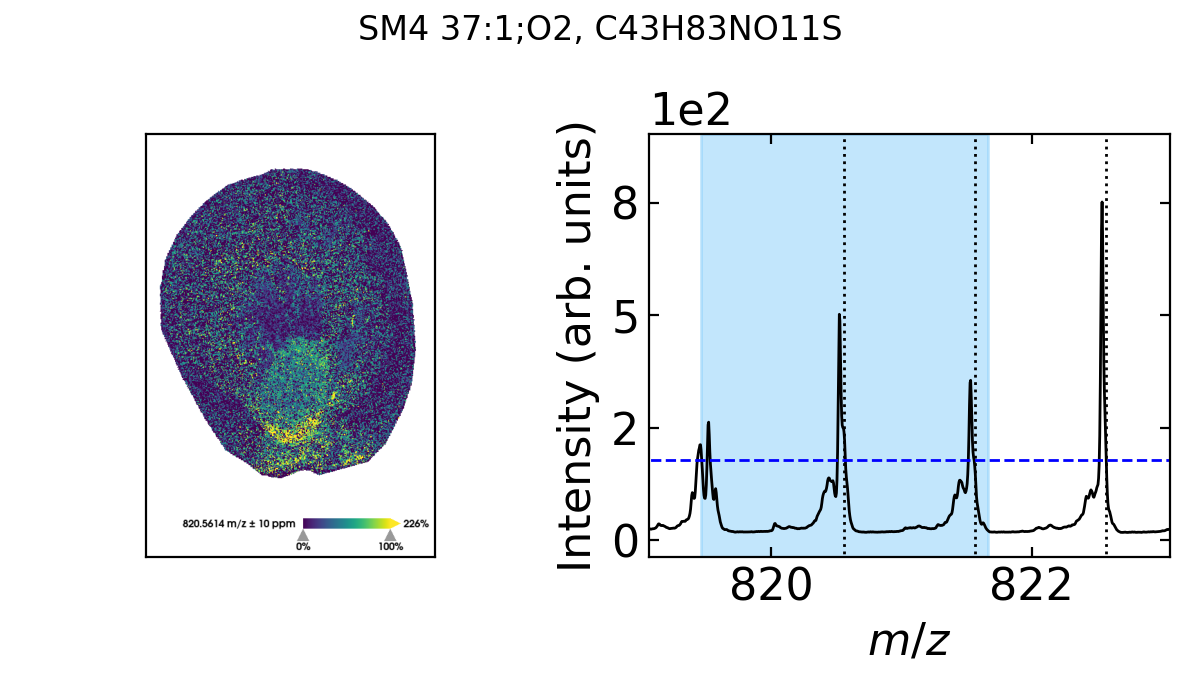

Supplement: Supplementary file 3 — Supplementary Data 1 [file 41467_2025_59839_MOESM3_ESM.zip › Suppl_Dataset_1_REV/qTOF_data1_slide1_python/820.561407_qTOF_60w_1.png]

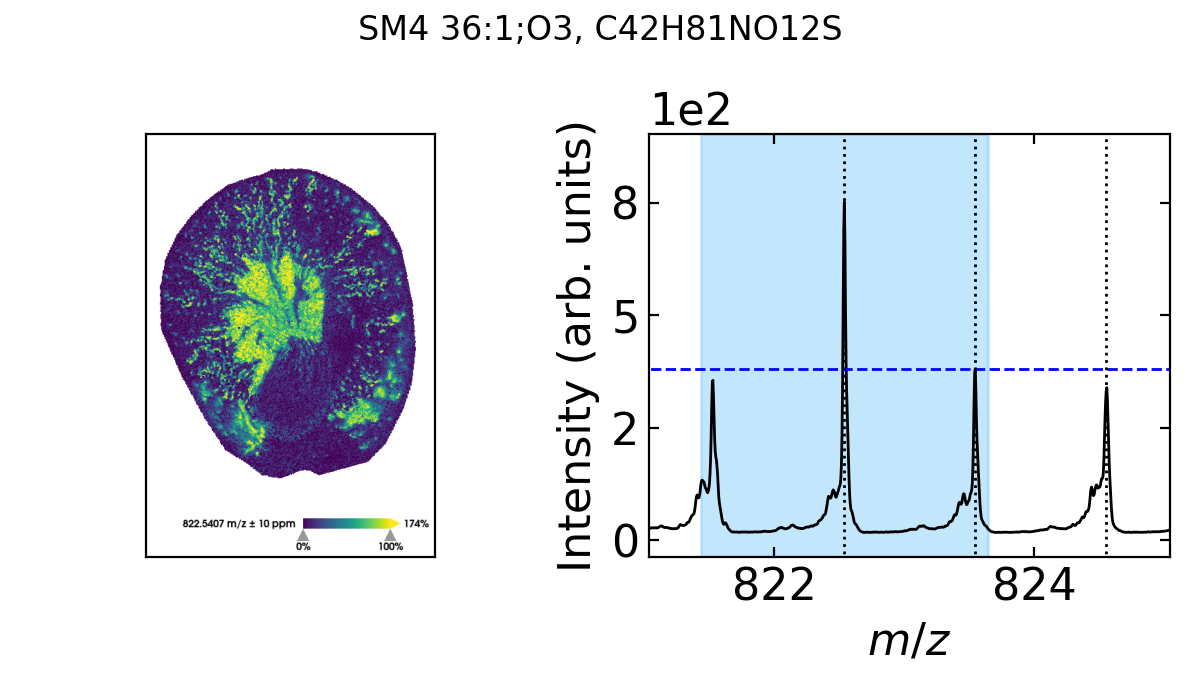

Supplement: Supplementary file 3 — Supplementary Data 1 [file 41467_2025_59839_MOESM3_ESM.zip › Suppl_Dataset_1_REV/qTOF_data1_slide1_python/822.540672_qTOF_60w_1.png]

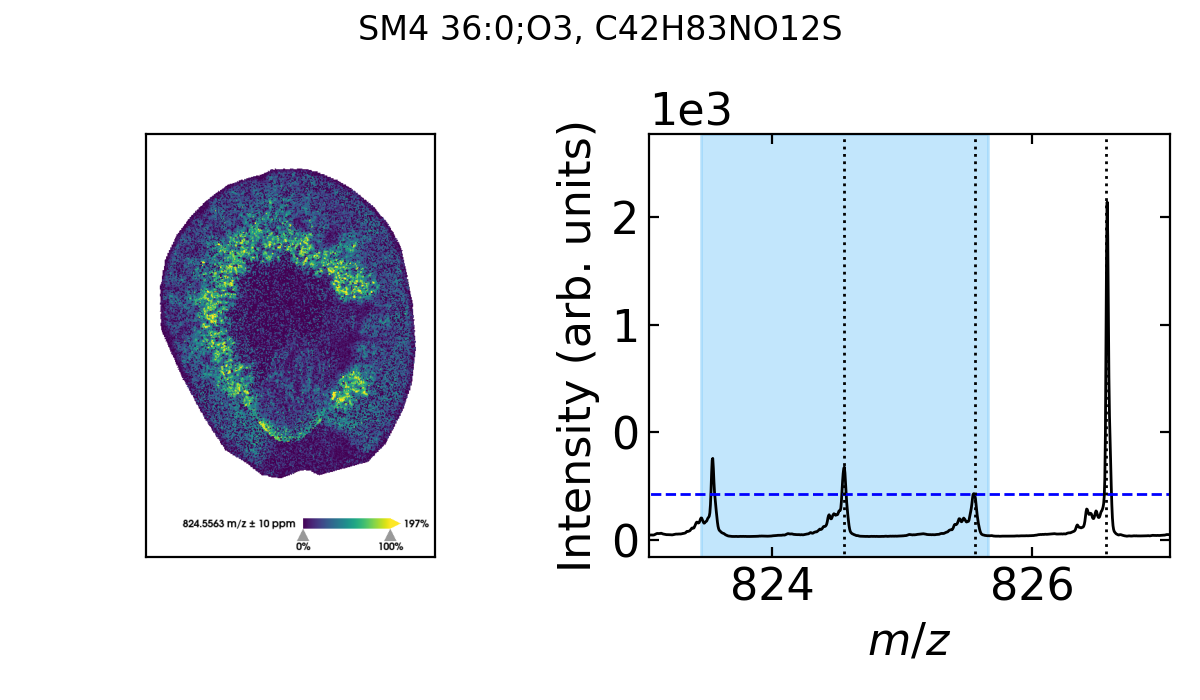

Supplement: Supplementary file 3 — Supplementary Data 1 [file 41467_2025_59839_MOESM3_ESM.zip › Suppl_Dataset_1_REV/qTOF_data1_slide1_python/824.556322_qTOF_60w_1.png]

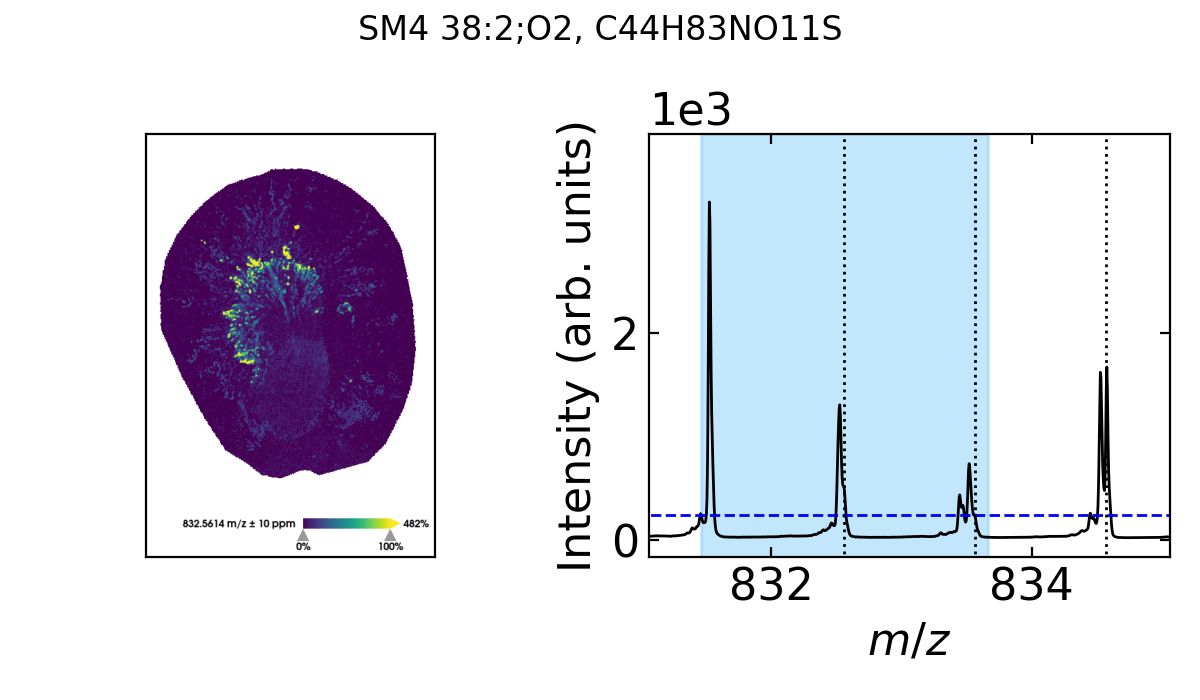

Supplement: Supplementary file 3 — Supplementary Data 1 [file 41467_2025_59839_MOESM3_ESM.zip › Suppl_Dataset_1_REV/qTOF_data1_slide1_python/832.561407_qTOF_60w_1.png]

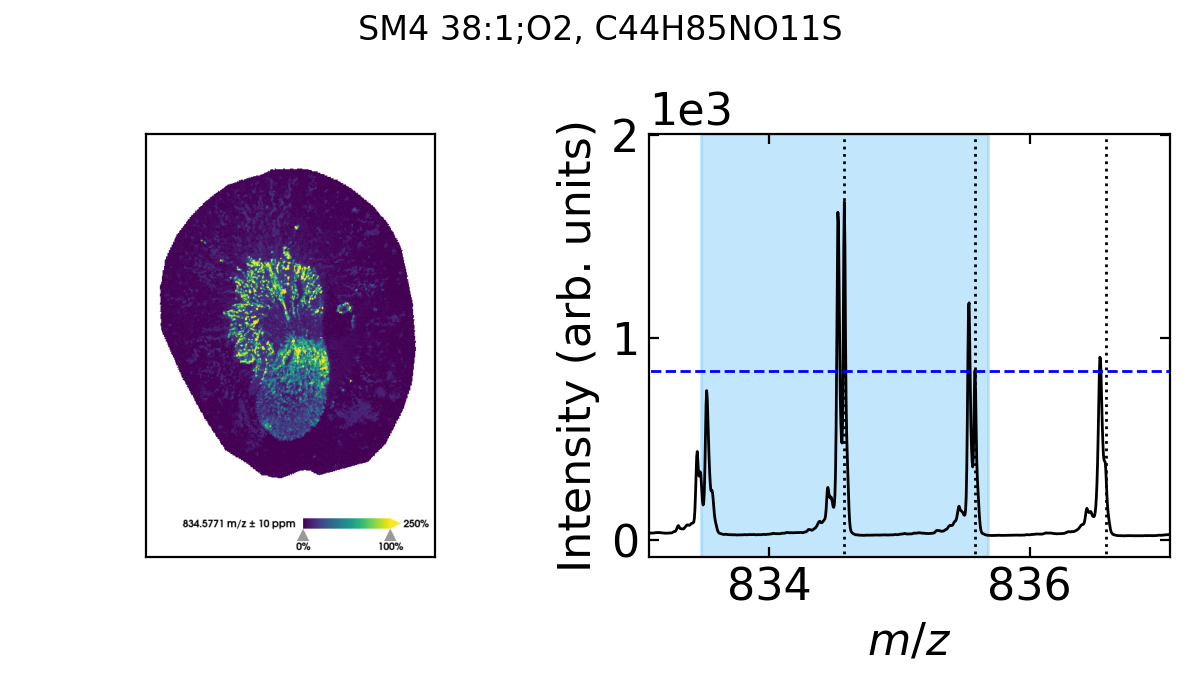

Supplement: Supplementary file 3 — Supplementary Data 1 [file 41467_2025_59839_MOESM3_ESM.zip › Suppl_Dataset_1_REV/qTOF_data1_slide1_python/834.577057_qTOF_60w_1.png]

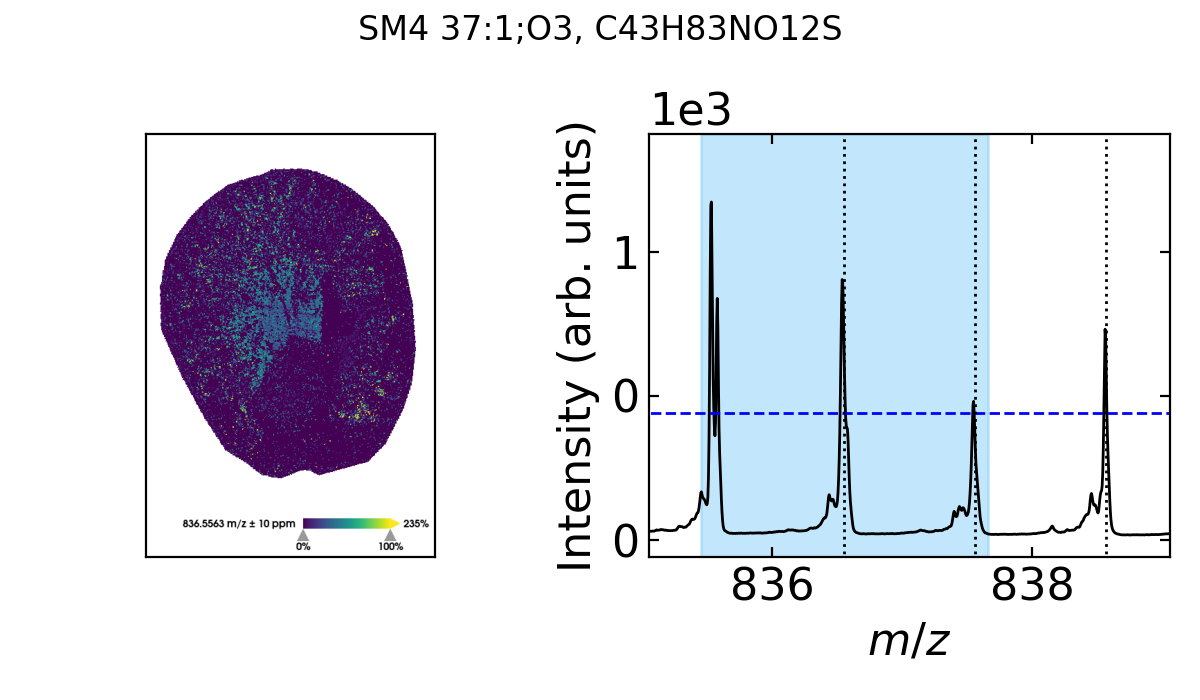

Supplement: Supplementary file 3 — Supplementary Data 1 [file 41467_2025_59839_MOESM3_ESM.zip › Suppl_Dataset_1_REV/qTOF_data1_slide1_python/836.556322_qTOF_60w_1.png]

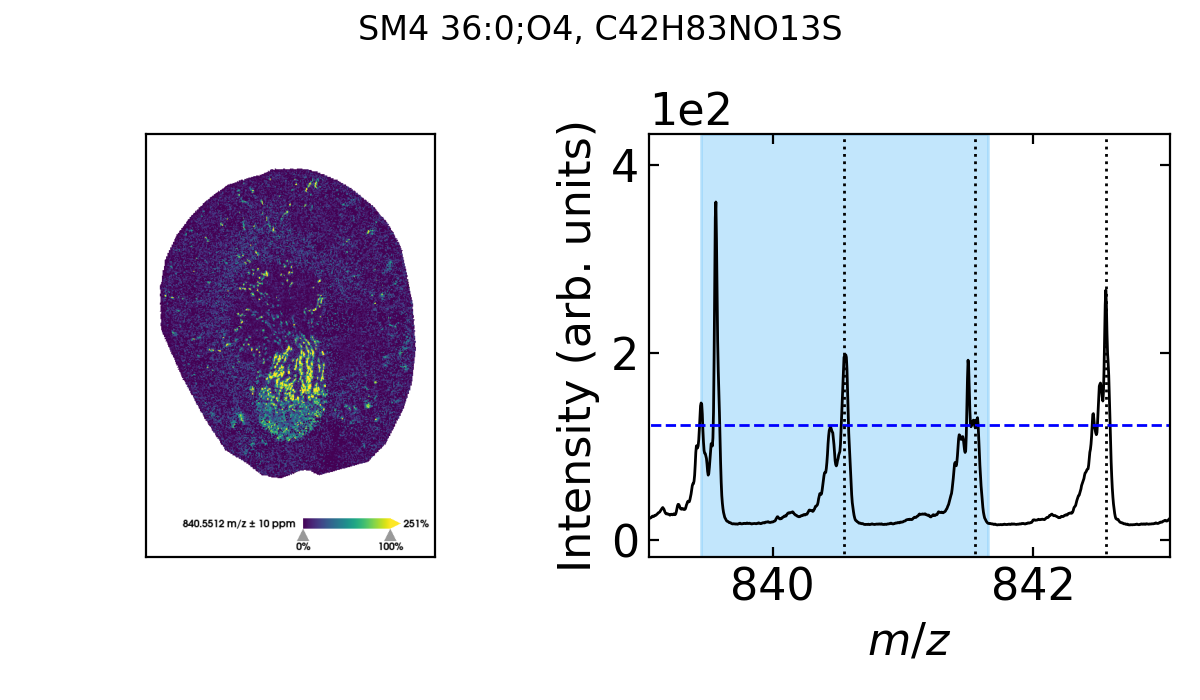

Supplement: Supplementary file 3 — Supplementary Data 1 [file 41467_2025_59839_MOESM3_ESM.zip › Suppl_Dataset_1_REV/qTOF_data1_slide1_python/840.551237_qTOF_60w_1.png]

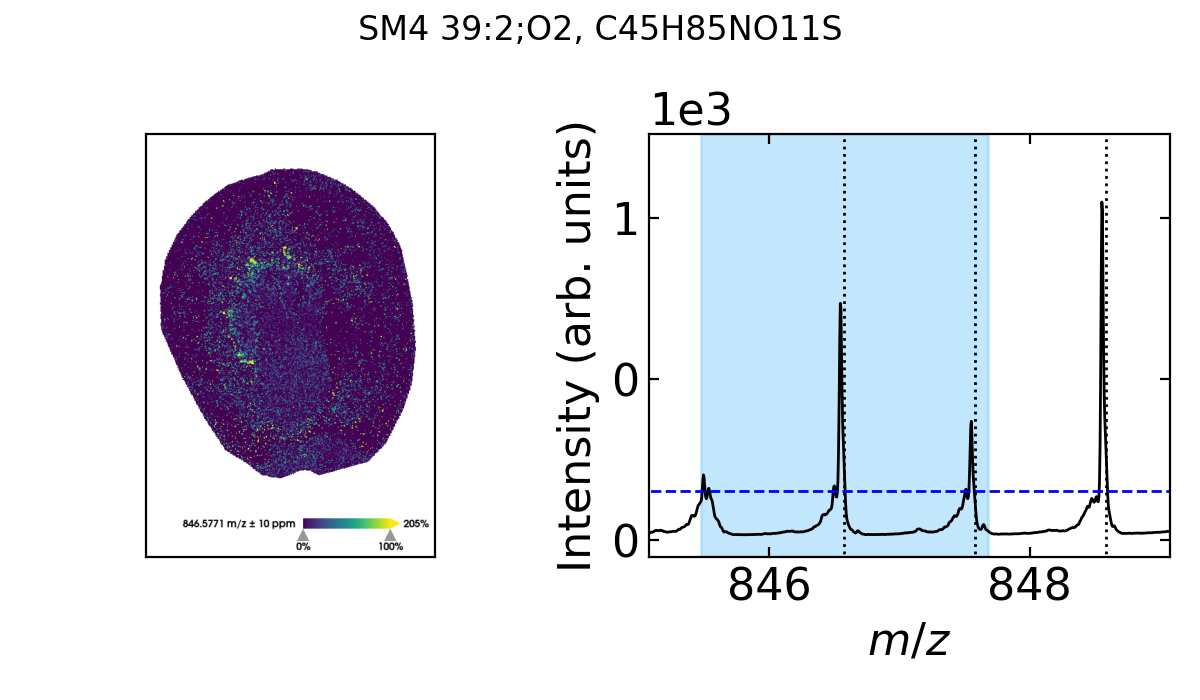

Supplement: Supplementary file 3 — Supplementary Data 1 [file 41467_2025_59839_MOESM3_ESM.zip › Suppl_Dataset_1_REV/qTOF_data1_slide1_python/846.577057_qTOF_60w_1.png]

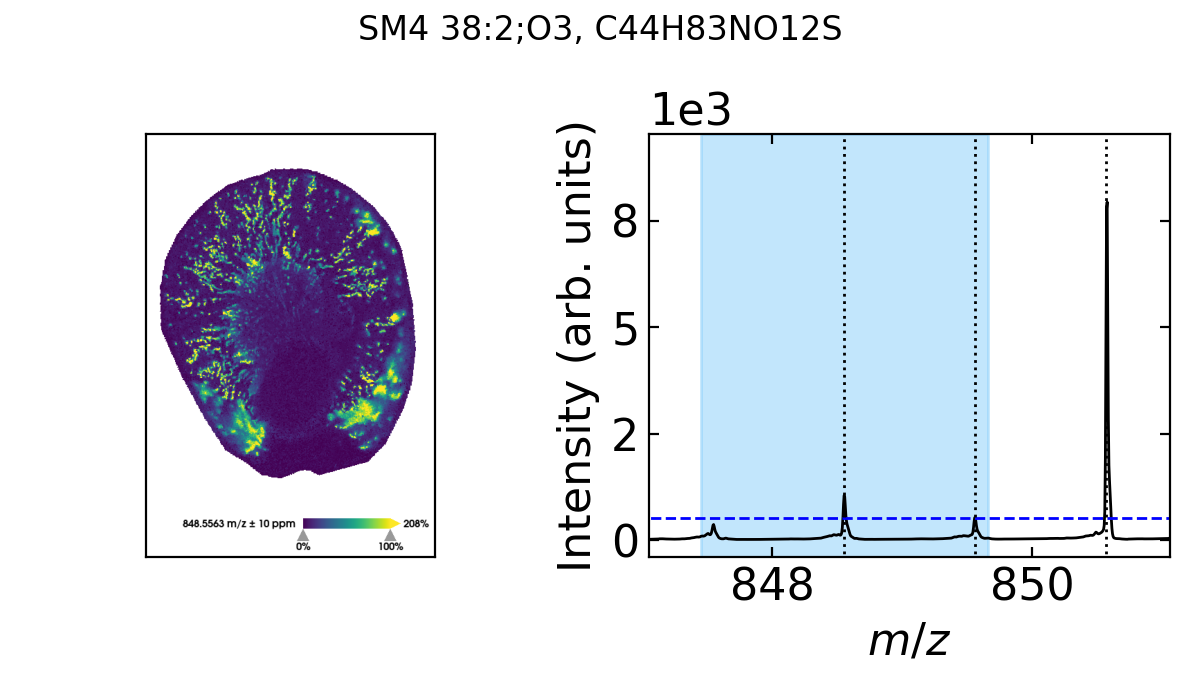

Supplement: Supplementary file 3 — Supplementary Data 1 [file 41467_2025_59839_MOESM3_ESM.zip › Suppl_Dataset_1_REV/qTOF_data1_slide1_python/848.556322_qTOF_60w_1.png]

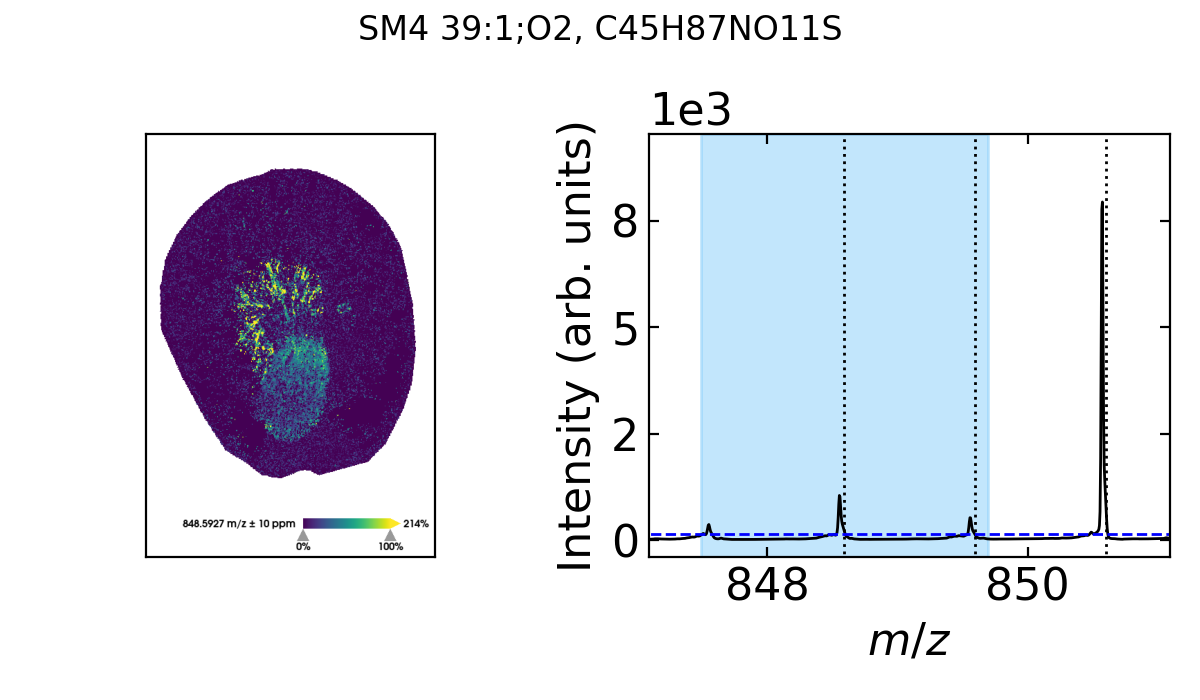

Supplement: Supplementary file 3 — Supplementary Data 1 [file 41467_2025_59839_MOESM3_ESM.zip › Suppl_Dataset_1_REV/qTOF_data1_slide1_python/848.592707_qTOF_60w_1.png]

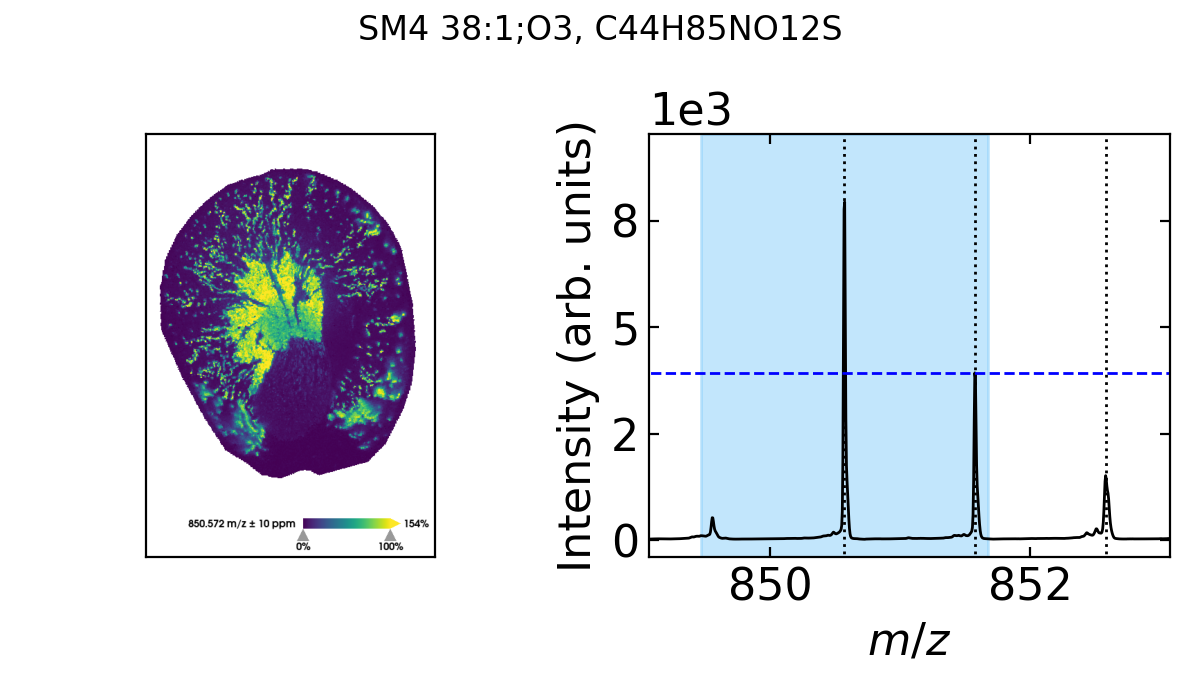

Supplement: Supplementary file 3 — Supplementary Data 1 [file 41467_2025_59839_MOESM3_ESM.zip › Suppl_Dataset_1_REV/qTOF_data1_slide1_python/850.571972_qTOF_60w_1.png]

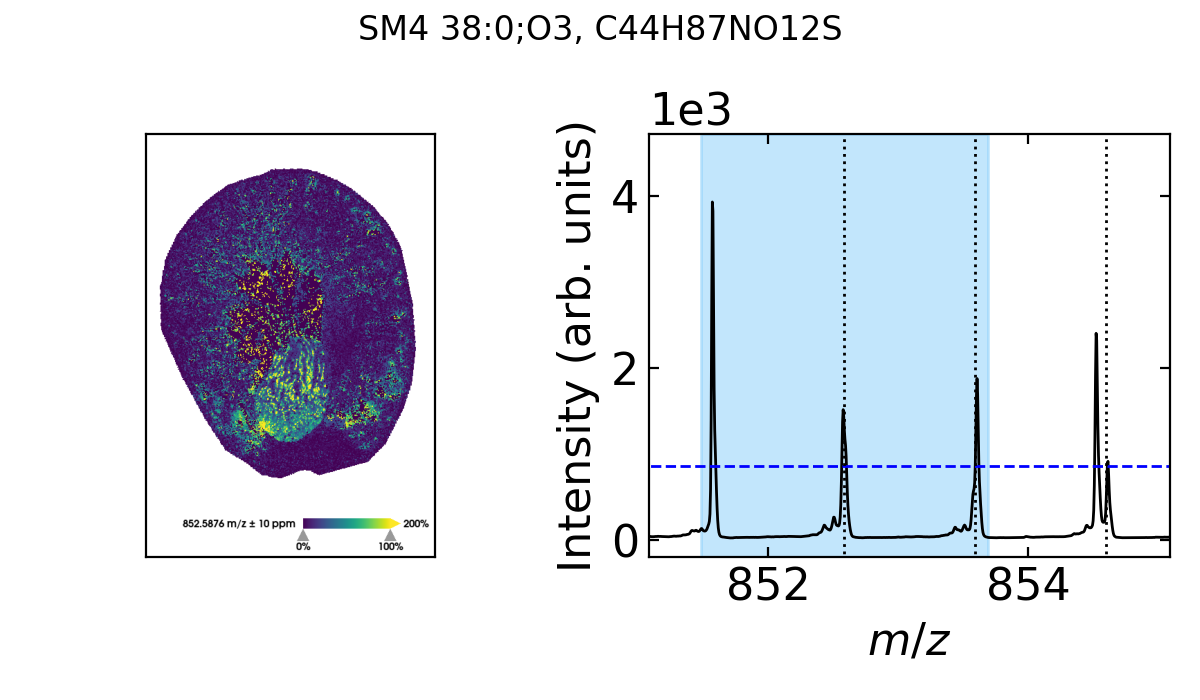

Supplement: Supplementary file 3 — Supplementary Data 1 [file 41467_2025_59839_MOESM3_ESM.zip › Suppl_Dataset_1_REV/qTOF_data1_slide1_python/852.587622_qTOF_60w_1.png]

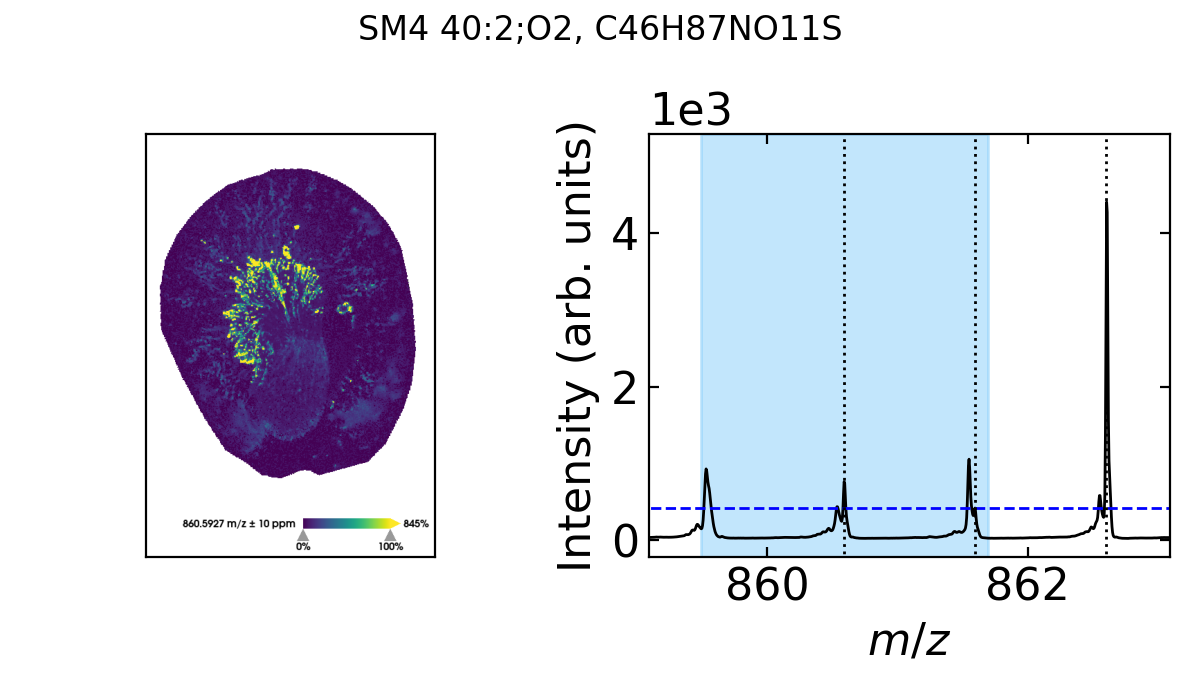

Supplement: Supplementary file 3 — Supplementary Data 1 [file 41467_2025_59839_MOESM3_ESM.zip › Suppl_Dataset_1_REV/qTOF_data1_slide1_python/860.592707_qTOF_60w_1.png]

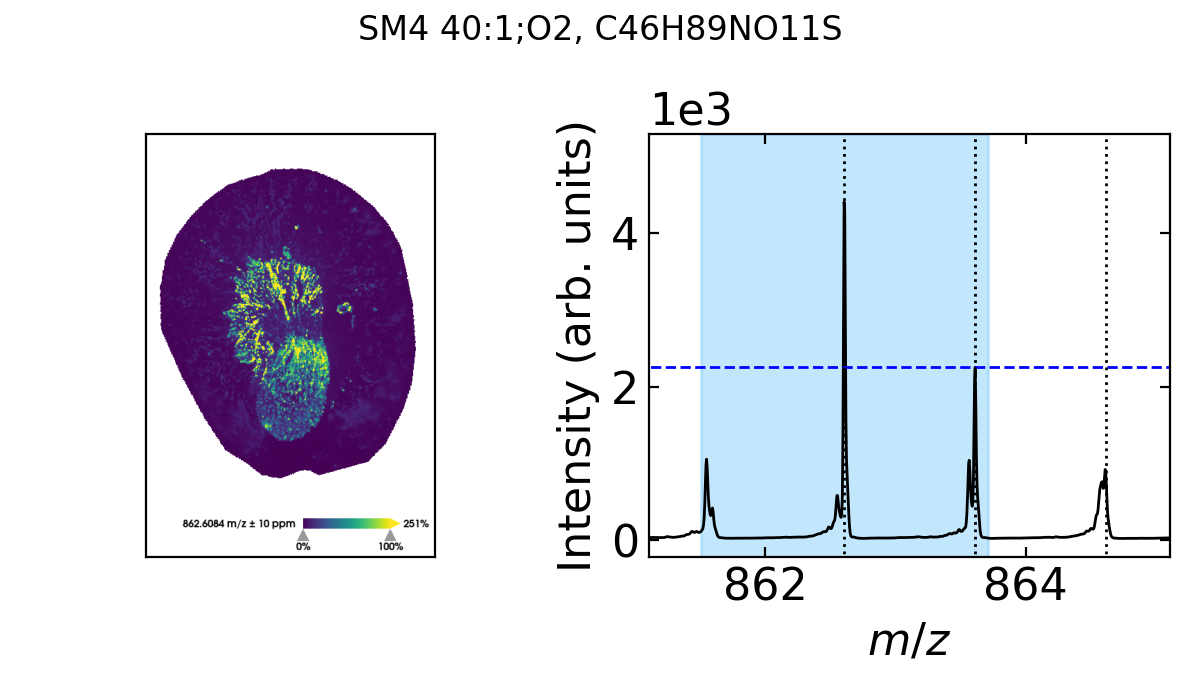

Supplement: Supplementary file 3 — Supplementary Data 1 [file 41467_2025_59839_MOESM3_ESM.zip › Suppl_Dataset_1_REV/qTOF_data1_slide1_python/862.608357_qTOF_60w_1.png]

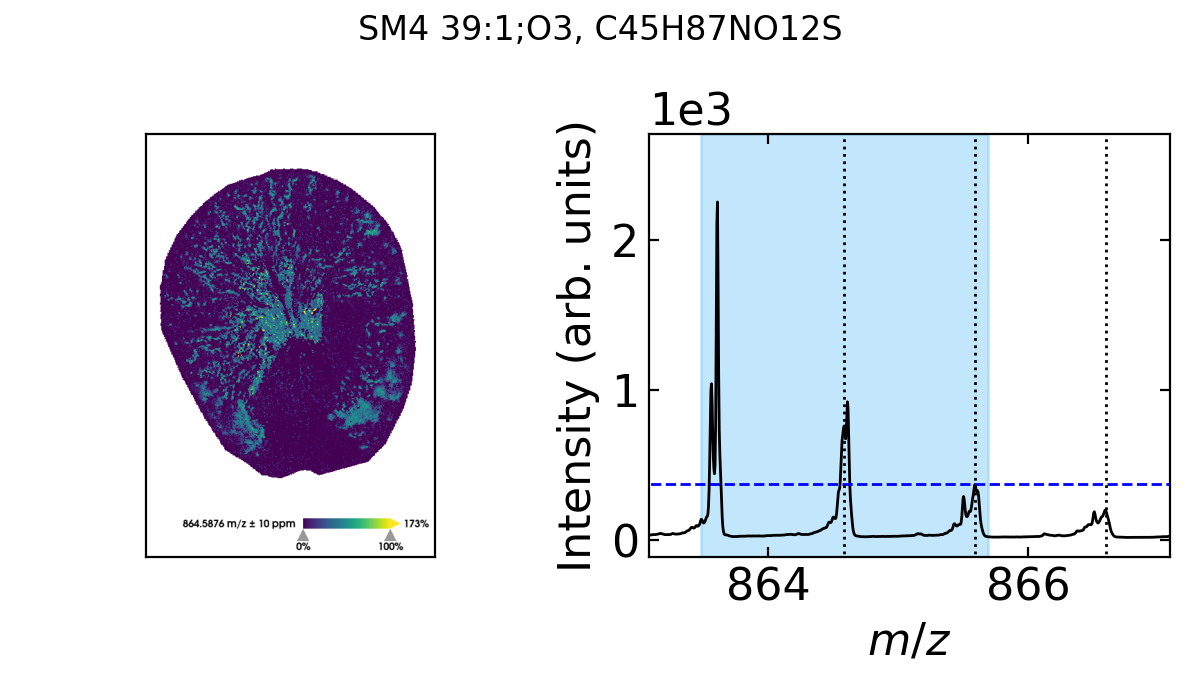

Supplement: Supplementary file 3 — Supplementary Data 1 [file 41467_2025_59839_MOESM3_ESM.zip › Suppl_Dataset_1_REV/qTOF_data1_slide1_python/864.587622_qTOF_60w_1.png]

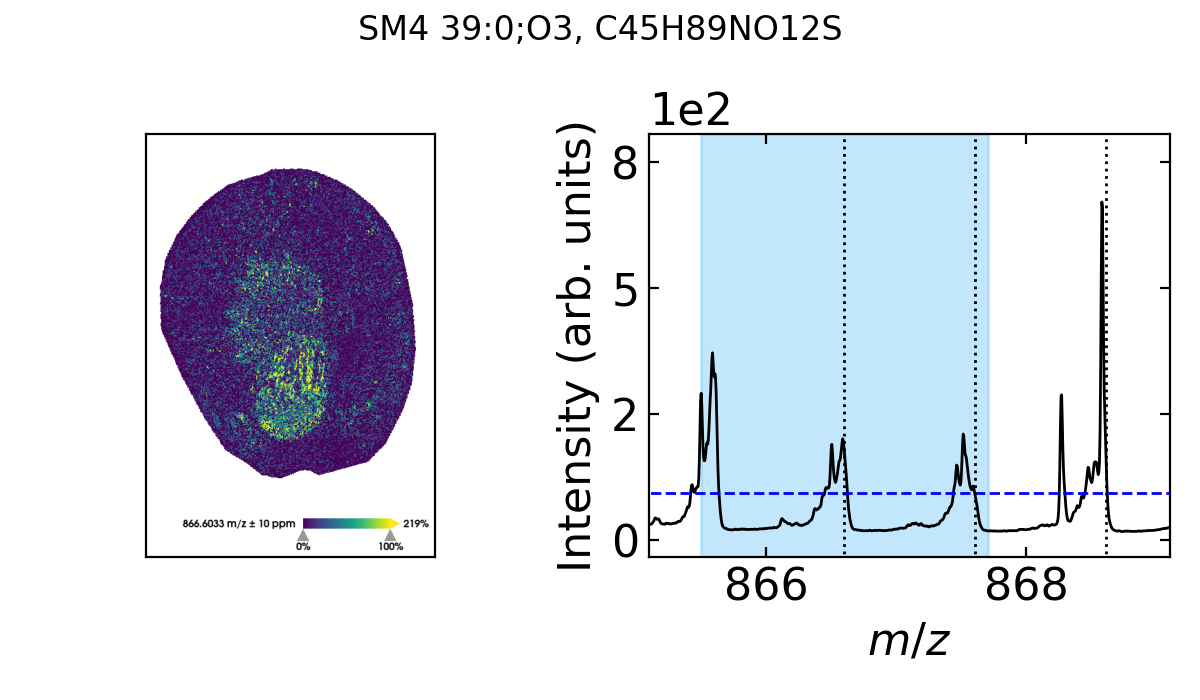

Supplement: Supplementary file 3 — Supplementary Data 1 [file 41467_2025_59839_MOESM3_ESM.zip › Suppl_Dataset_1_REV/qTOF_data1_slide1_python/866.603272_qTOF_60w_1.png]

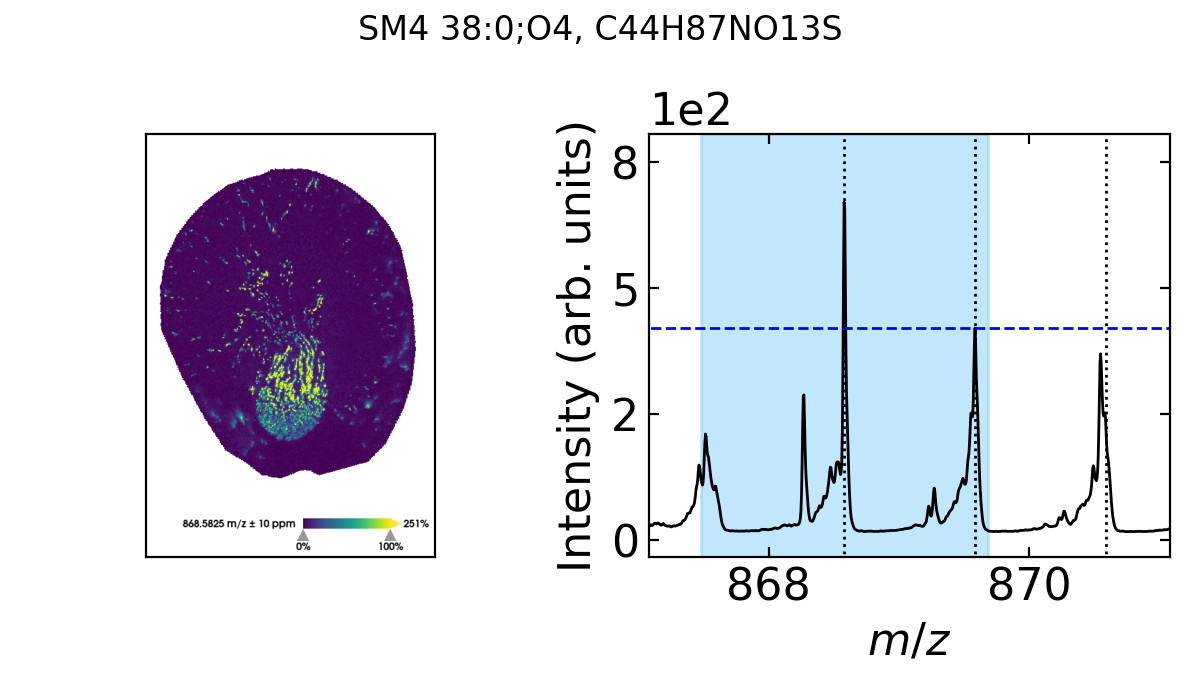

Supplement: Supplementary file 3 — Supplementary Data 1 [file 41467_2025_59839_MOESM3_ESM.zip › Suppl_Dataset_1_REV/qTOF_data1_slide1_python/868.582536_qTOF_60w_1.png]

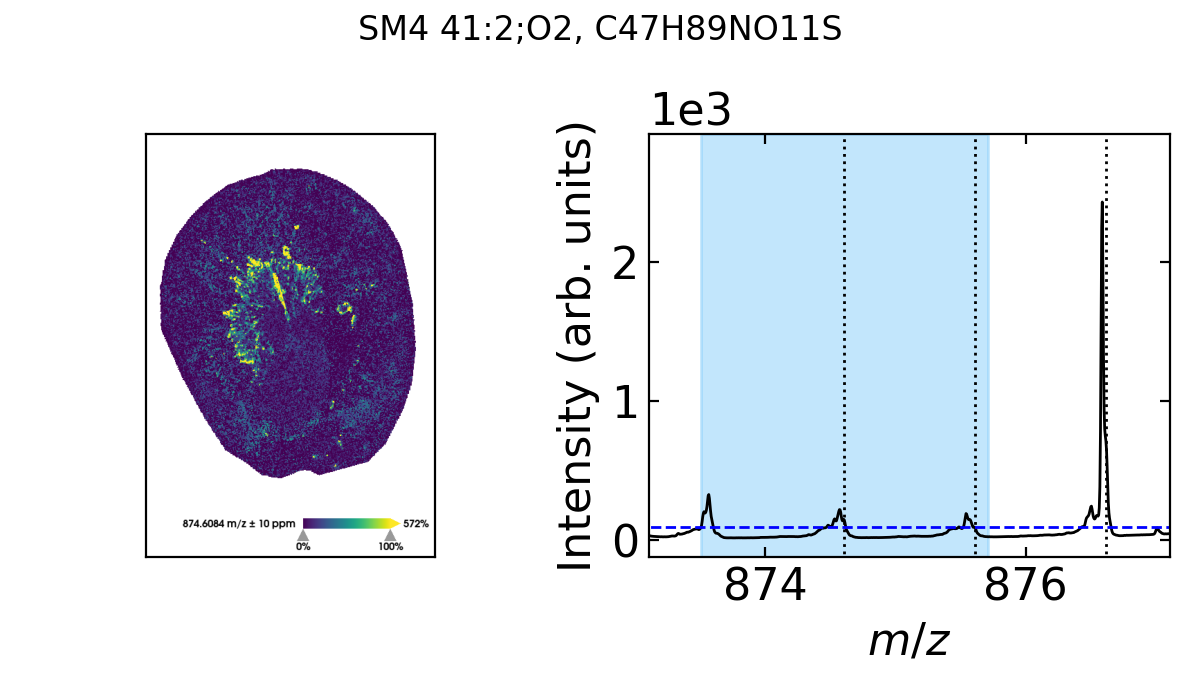

Supplement: Supplementary file 3 — Supplementary Data 1 [file 41467_2025_59839_MOESM3_ESM.zip › Suppl_Dataset_1_REV/qTOF_data1_slide1_python/874.608357_qTOF_60w_1.png]

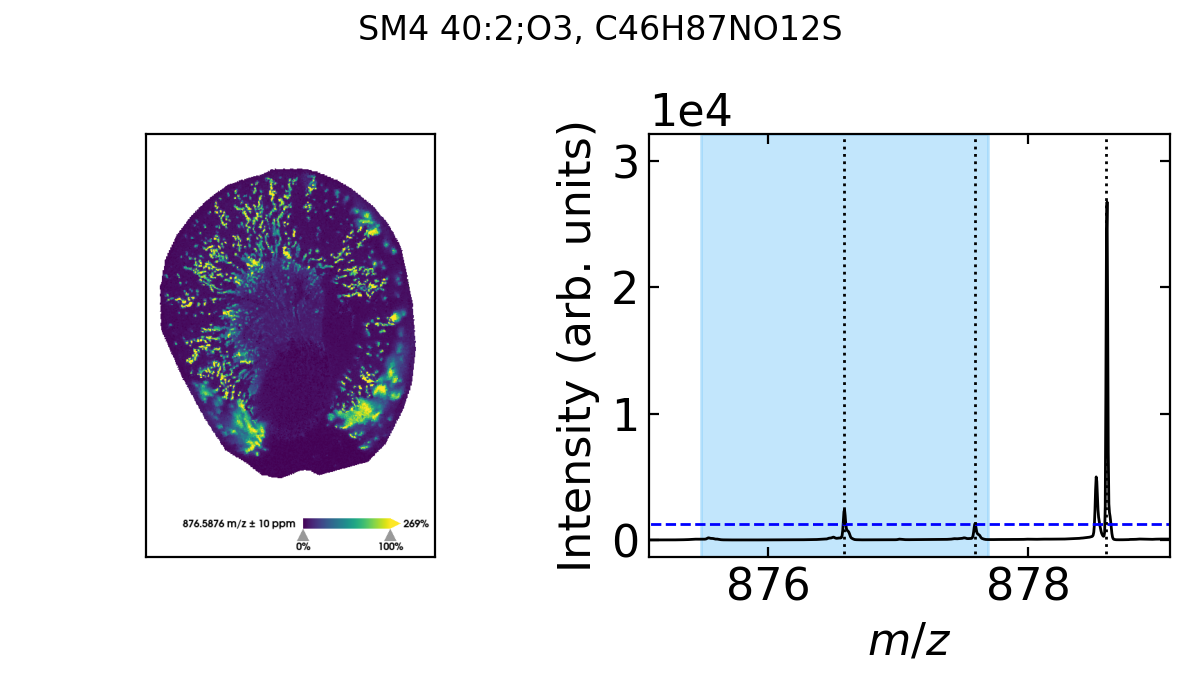

Supplement: Supplementary file 3 — Supplementary Data 1 [file 41467_2025_59839_MOESM3_ESM.zip › Suppl_Dataset_1_REV/qTOF_data1_slide1_python/876.587622_qTOF_60w_1.png]

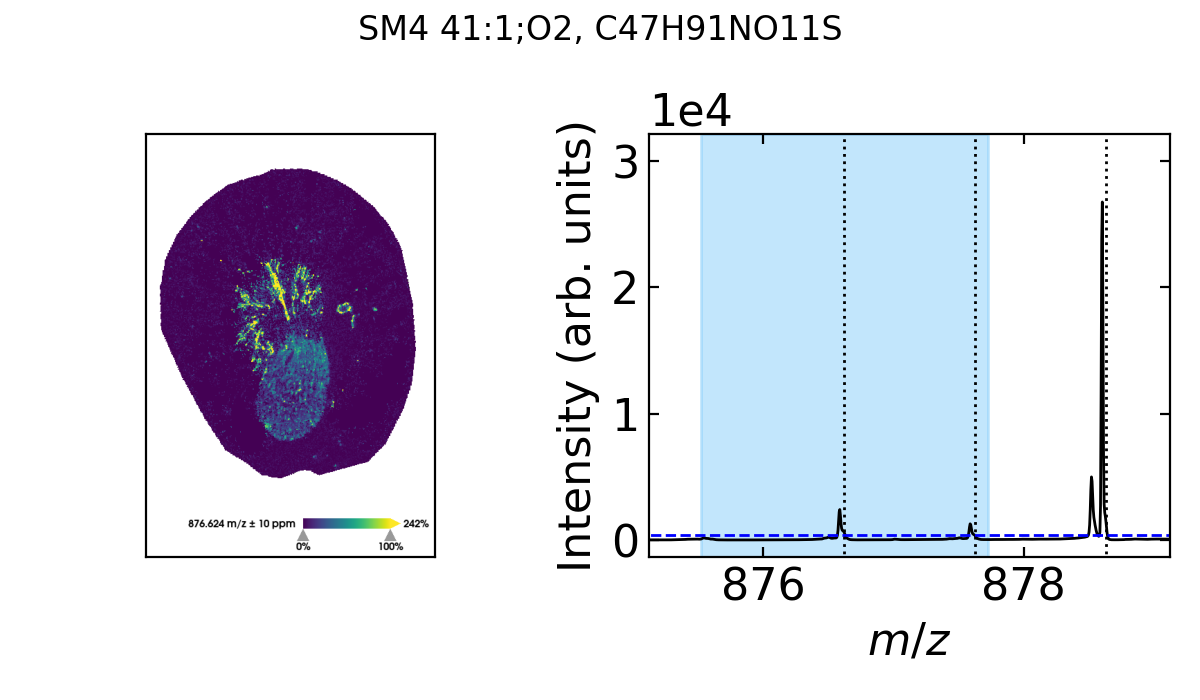

Supplement: Supplementary file 3 — Supplementary Data 1 [file 41467_2025_59839_MOESM3_ESM.zip › Suppl_Dataset_1_REV/qTOF_data1_slide1_python/876.624007_qTOF_60w_1.png]

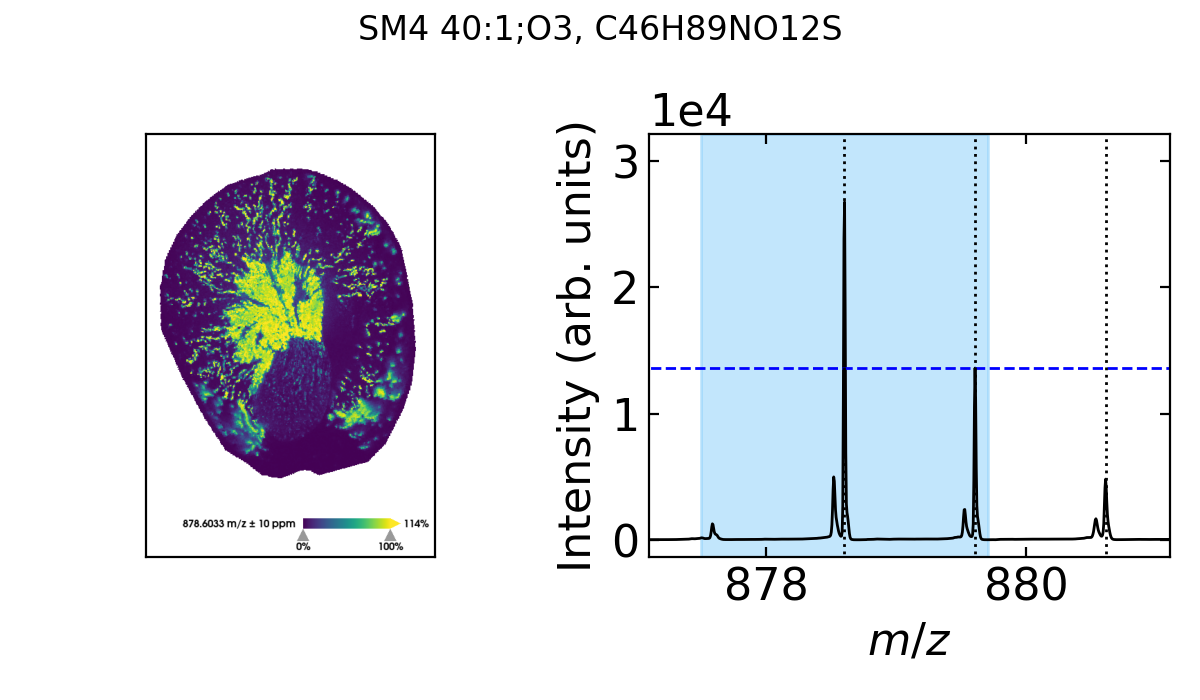

Supplement: Supplementary file 3 — Supplementary Data 1 [file 41467_2025_59839_MOESM3_ESM.zip › Suppl_Dataset_1_REV/qTOF_data1_slide1_python/878.603272_qTOF_60w_1.png]

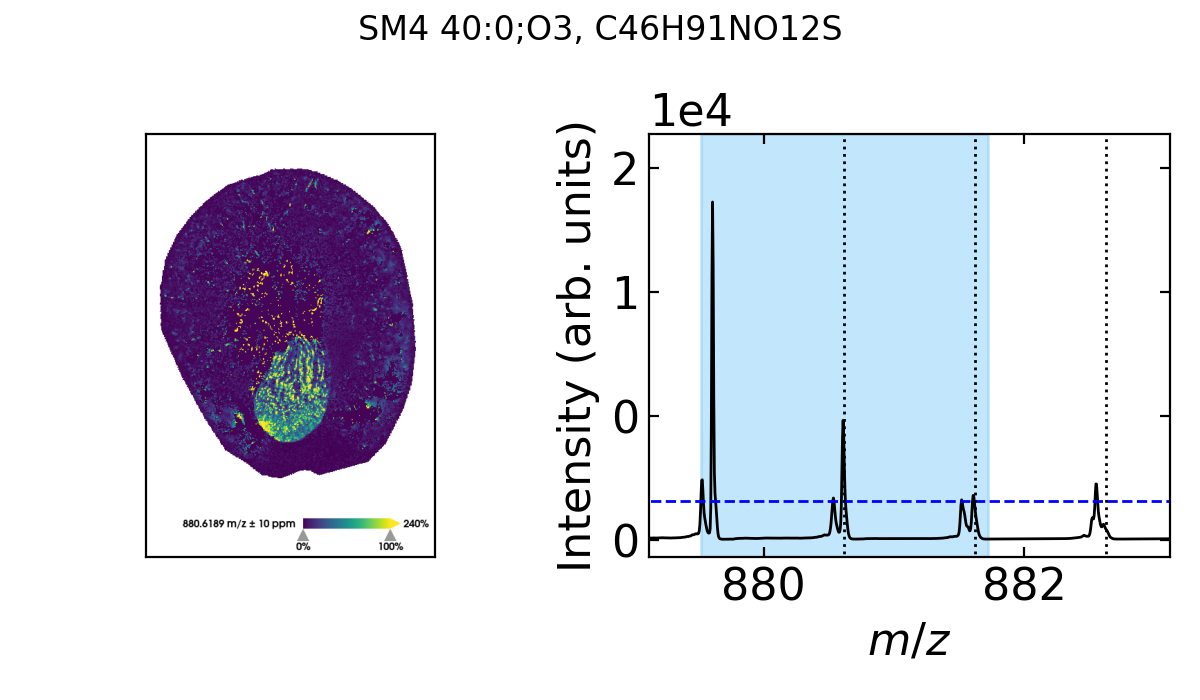

Supplement: Supplementary file 3 — Supplementary Data 1 [file 41467_2025_59839_MOESM3_ESM.zip › Suppl_Dataset_1_REV/qTOF_data1_slide1_python/880.618922_qTOF_60w_1.png]

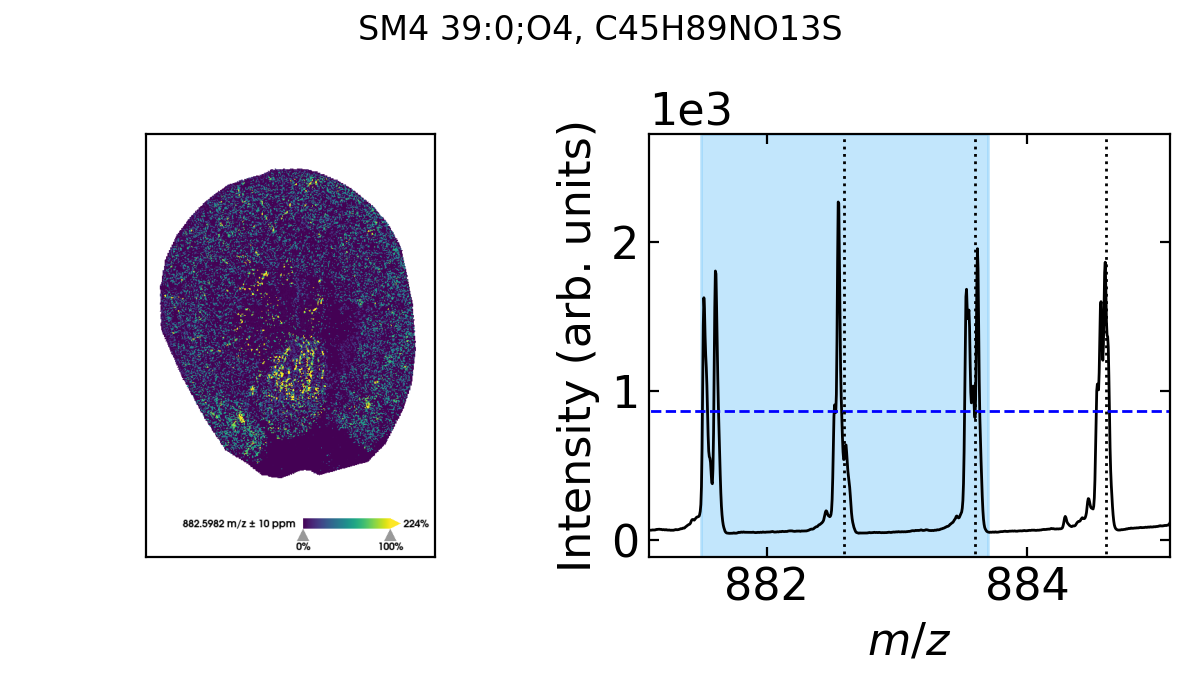

Supplement: Supplementary file 3 — Supplementary Data 1 [file 41467_2025_59839_MOESM3_ESM.zip › Suppl_Dataset_1_REV/qTOF_data1_slide1_python/882.598187_qTOF_60w_1.png]

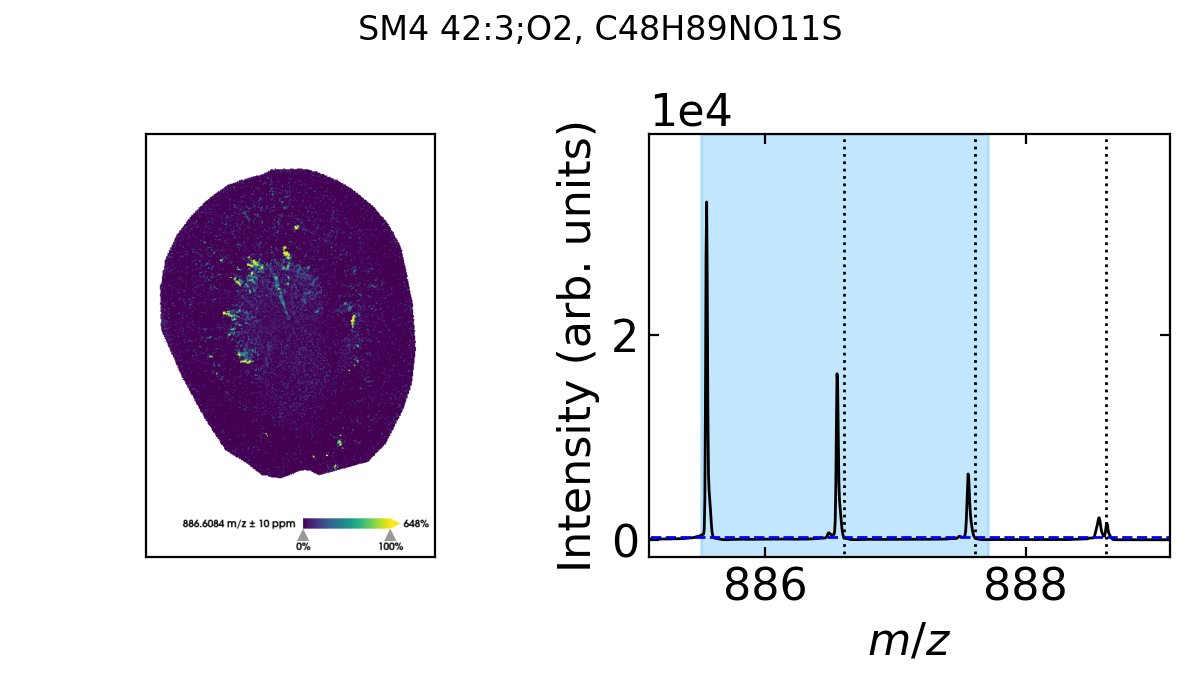

Supplement: Supplementary file 3 — Supplementary Data 1 [file 41467_2025_59839_MOESM3_ESM.zip › Suppl_Dataset_1_REV/qTOF_data1_slide1_python/886.608357_qTOF_60w_1.png]

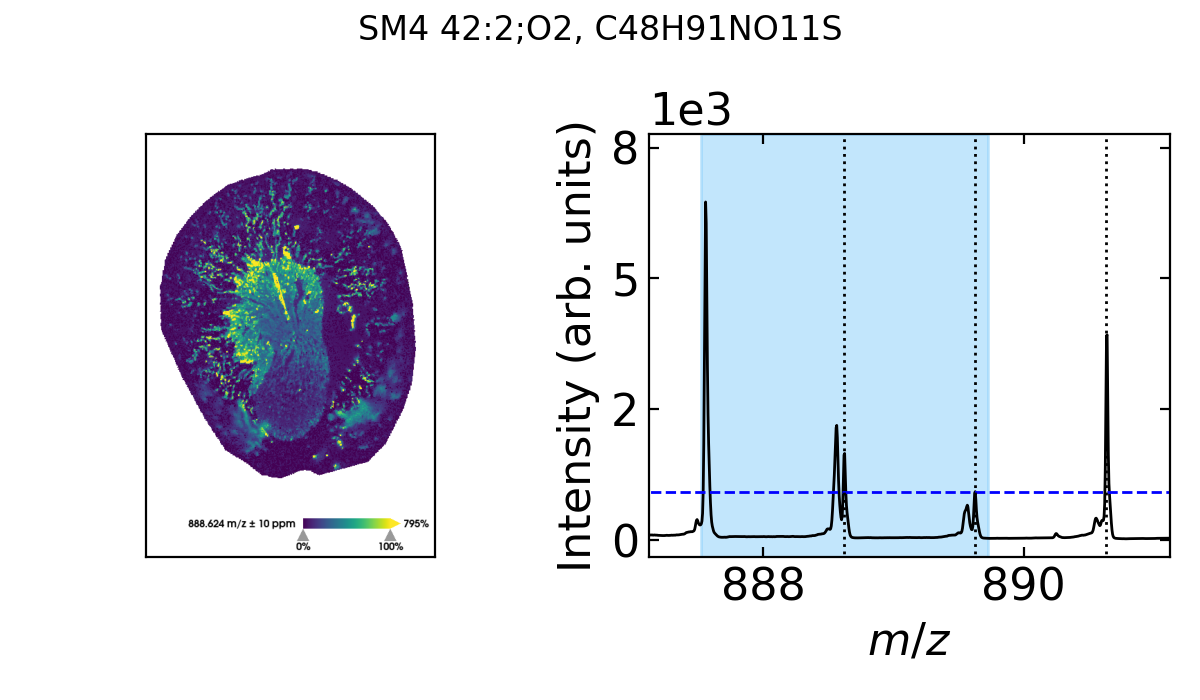

Supplement: Supplementary file 3 — Supplementary Data 1 [file 41467_2025_59839_MOESM3_ESM.zip › Suppl_Dataset_1_REV/qTOF_data1_slide1_python/888.624007_qTOF_60w_1.png]

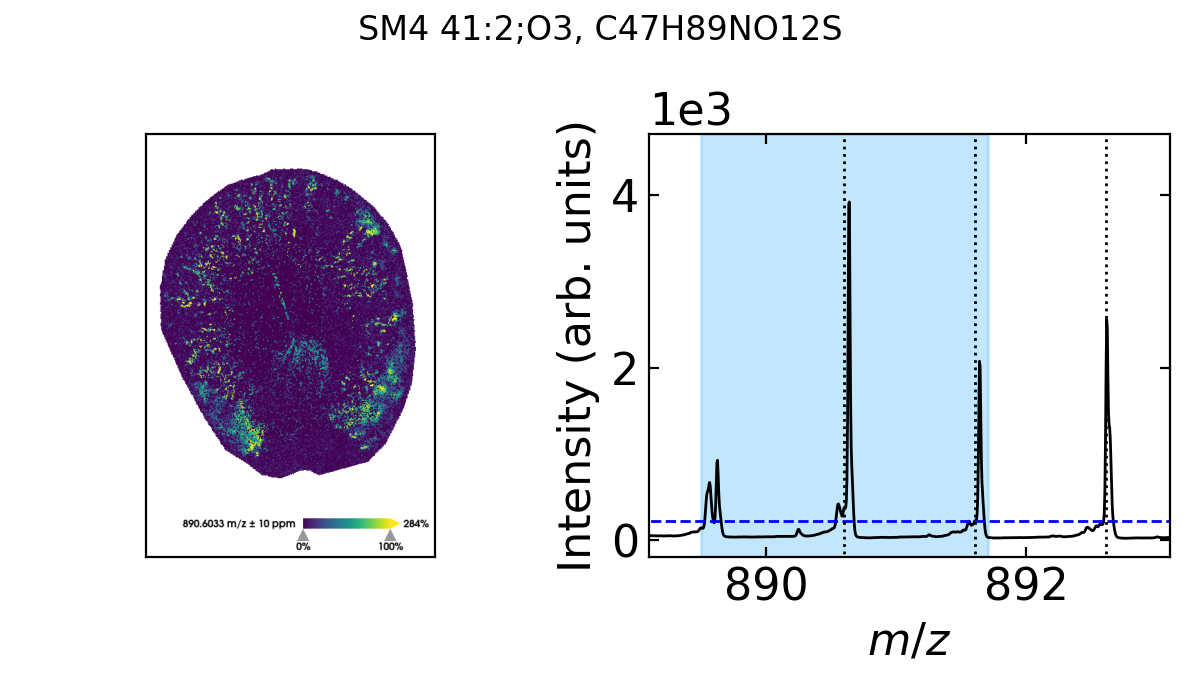

Supplement: Supplementary file 3 — Supplementary Data 1 [file 41467_2025_59839_MOESM3_ESM.zip › Suppl_Dataset_1_REV/qTOF_data1_slide1_python/890.603272_qTOF_60w_1.png]

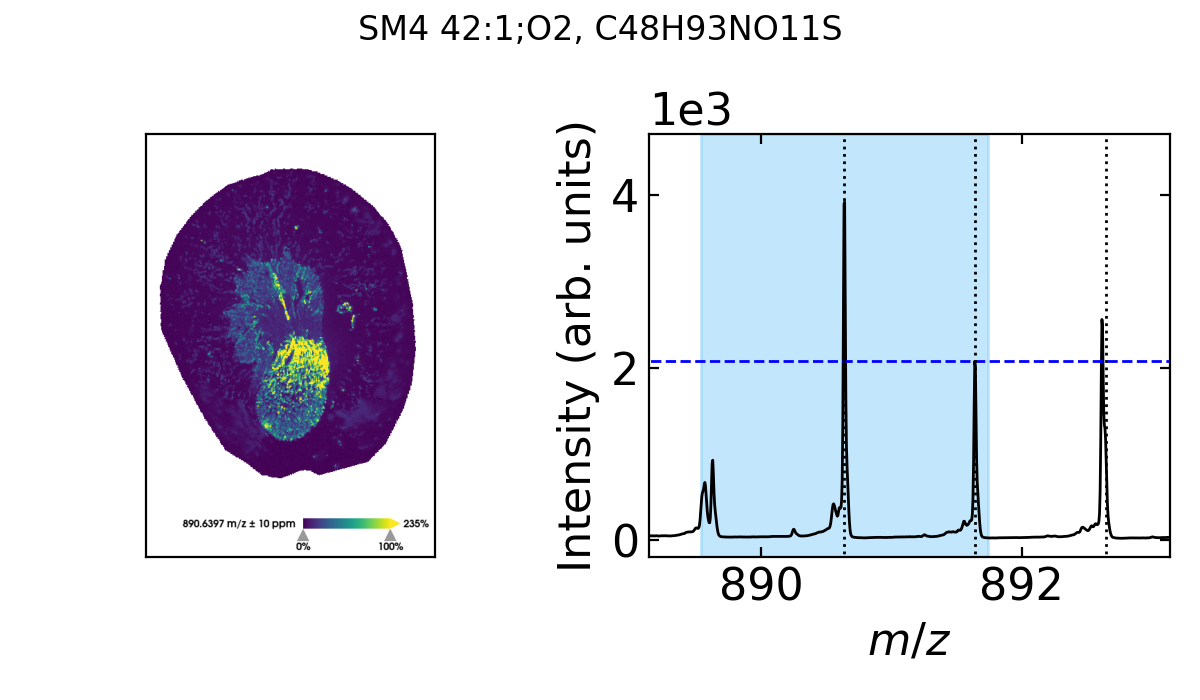

Supplement: Supplementary file 3 — Supplementary Data 1 [file 41467_2025_59839_MOESM3_ESM.zip › Suppl_Dataset_1_REV/qTOF_data1_slide1_python/890.639657_qTOF_60w_1.png]
